# A chromosome-level genome assembly and intestinal transcriptome of *Trypoxylus dichotomus* (Coleoptera: Scarabaeidae) to understand its lignocellulose digestion ability

**DOI:** 10.1093/gigascience/giac059

**Published:** 2022-06-28

**Authors:** Qingyun Wang, Liwei Liu, Sujiong Zhang, Hong Wu, Junhao Huang

**Affiliations:** National Joint Local Engineering Laboratory for High-Efficient Preparation of Biopesticide, Zhejiang A&F University, 666 Wusu Street, Lin'an, Hangzhou, Zhejiang 311300, China; National Joint Local Engineering Laboratory for High-Efficient Preparation of Biopesticide, Zhejiang A&F University, 666 Wusu Street, Lin'an, Hangzhou, Zhejiang 311300, China; Zhejiang Museum of Natural History, No. 6 West Lake Cultural Square, Hangzhou, Zhejiang 310014, China; Dapanshan Insect Institute of Zhejiang, Pan'an, Zhejiang 322300, China; National Joint Local Engineering Laboratory for High-Efficient Preparation of Biopesticide, Zhejiang A&F University, 666 Wusu Street, Lin'an, Hangzhou, Zhejiang 311300, China; National Joint Local Engineering Laboratory for High-Efficient Preparation of Biopesticide, Zhejiang A&F University, 666 Wusu Street, Lin'an, Hangzhou, Zhejiang 311300, China

**Keywords:** chromosome rearrangement, gene family, intestinal transcriptome, lignocellulose digestion, rhinoceros beetle

## Abstract

Lignocellulose, as the key structural component of plant biomass, is a recalcitrant structure, difficult to degrade. The traditional management of plant waste, including landfill and incineration, usually causes serious environmental pollution and health problems. Interestingly, the xylophagous beetle, *Trypoxylus dichotomus*, can decompose lignocellulosic biomass. However, the genomics around the digestion mechanism of this beetle remain to be elucidated. Here, we assembled the genome of *T. dichotomus*, showing that the draft genome size of *T. dichotomus* is 636.27 Mb, with 95.37% scaffolds anchored onto 10 chromosomes. Phylogenetic results indicated that a divergent evolution between the ancestors of *T. dichotomus* and the closely related scarabaeid species *Onthophagus taurus* occurred in the early Cretaceous (120 million years ago). Through gene family evolution analysis, we found 67 rapidly evolving gene families, within which there were 2 digestive gene families (encoding Trypsin and Enoyl-(Acyl carrier protein) reductase) that have experienced significant expansion, indicating that they may contribute to the high degradation efficiency of lignocellulose in *T. dichotomus*. Additionally, events of chromosome breakage and rearrangement were observed by synteny analysis during the evolution of *T. dichotomus* due to chromosomes 6 and 8 of *T. dichotomus* being intersected with chromosomes 2 and 10 of *Tribolium castaneum*, respectively. Furthermore, the comparative transcriptome analyses of larval guts showed that the digestion-related genes were more commonly expressed in the midgut or mushroom residue group than the hindgut or sawdust group. This study reports the well-assembled and annotated genome of *T. dichotomus*, providing genomic and transcriptomic bases for further understanding the functional and evolutionary mechanisms of lignocellulose digestion in *T. dichotomus*.

## Introduction

As a key structural component of plant biomass and an important route of carbon fixation, lignocellulosic biomass is found in all kinds of living and dead plants. This biomass is principally composed of celluloses, hemicelluloses, pectins, and lignins [1], which form a complex cross-linked and recalcitrant structure that protects carbohydrates from decomposition by microorganisms or enzymes [2, 3]. The traditional management of plant waste is usually done via landfill or incineration, which causes serious environmental pollution and health problems [4]. Thus, recycling plant wastes produced by human production is a noteworthy environmental issue [5]. Currently, chemical and biological pretreatment of lignocellulose degradation—especially biotransformation, an environmentally friendly and sustainable strategy for biofuels and biomaterial production—has catalyzed a great interest [6–8].

Due to the complex structural and chemical mechanisms of lignocellulose, lignocellulose decomposition is not common among animals [9, 10] except for wood-feeding insects such as termites, wood-feeding cockroaches, beetles, and wood wasps [7, 8, 10–12]. These insects are involved in the degradation of lignocellulose and other types of biomass by consuming plant cell walls, thereby contributing to lignocellulose bioconversion and energy utilization [13]. Among them, xylophagous termites are the most well known of efficient lignocellulose digesters, having been studied in detail, including in terms of functional genomics and symbiotic intestinal microorganisms [14–16]. Many studies have focused on the chemical degradation and microbiological deterioration of lignocellulose [17], but limited attention has been paid to the biodegradation ability and genetic traits of other xylophagous insects, including the well-known ornamental scarabaeid beetle, *Trypoxylus dichotomus* (Linnaeus, 1771; NCBI:txid273928), which has a similar diet to xylophagous termites.

The rhinoceros beetle, *T. dichotomus* (Coleoptera: Scarabaeidae), is an ecologically important xylophagous and saprophagous insect widely distributed in China and neighboring countries [18]. In the larval stage, it can decompose recalcitrant wood material and humus efficiently in the wild [19–21], and this has been harnessed industrially to biotransform the waste substrate from mushroom production [22]. *T. dichotomus* can secrete various digestive enzymes comprising cellulase, glycanase, and glycosidase to degrade lignocellulose-rich plant polymers [19], greatly promoting the formation of soil organic matter, the major pool of organic carbons [23, 24]. To date, several studies have focused on the digestive enzymes and mutualistic associations with microbial symbionts in larval guts [19, 21, 25–27]. However, without the genome data of *T. dichotomus*, the underlying mechanisms enabling the digestion of lignocellulose will not be revealed.

It is generally believed that different diets significantly affect the digestive enzymatic activities of beetles [28]. Regional differentiation of the digestive tract and adaptations to divergent feeding habits mediate efficient digestion of food and protect insects against hazardous substances therein [29]. Although larvae of the rhinoceros beetle could degrade decaying wood and mushroom residue efficiently, no work has studied their digestive ability in terms of different food eating habits. To understand the gut segment–specific function and molecular pattern of the digestive tract in larval *T. dichotomus*, it is necessary to identify the digestion-related genes and characterize their expression patterns associated with different food eating habits.

In the current study, we drafted the genome sequence of *T. dichotomus* and investigated its genomic characteristics through comparative genomic analysis with available data sets from other related insects. We also clarified the evolutionary history of gene families, highlighting the rapid expansion of 2 digestion-related gene families and possible chromosome evolution events in *T. dichotomus*. Furthermore, we conducted an intestinal transcriptome comparative analysis of the third instar larvae feeding on sawdust or mushroom residue and reveal that the expression of digestive enzyme genes was significantly higher in the midgut or mushroom residue group than in the hindgut or sawdust group. Finally, we illustrate the effects of different food habits on *T. dichotomus* larval intestinal segments and digestive ability.

## Materials and Methods

### Sampling and sequencing

The male and female adult samples and living larvae of *T. dichotomus* were obtained from the artificial breeding base in Pan'an County (28.94°N, 120.55°E), Zhejiang Province, China, in May 2020 and transported to the laboratory. The adult samples were washed 3 times with distilled water and then transferred to a clean bench for dissection. In view of the following 2 factors, (i) the sex determination system of *T. dichotomus* is XY, in which the Y chromosome is much smaller than the X chromosome [30], and (ii) there was only 1 pair of newly emerged beetles of different sexes obtained for genome sequencing; thus, to meet the sequencing requirements, muscle of a female thorax was prepared for Illumina and Nanopore sequencing, and then a male thorax was dissected for Hi-C and RNA sequencing (Supplementary Table S1). Prior to the extraction of genomic DNA and RNA, the samples were transferred to liquid nitrogen for preservation. The similar-sized larvae were divided into 2 groups and reared with high-temperature sterilized sawdust and mushroom residue (composed of wood fiber and fungal mycelia) at 20–25°C and 50–60% humidity for 2 months, separately.

**Figure 1:**
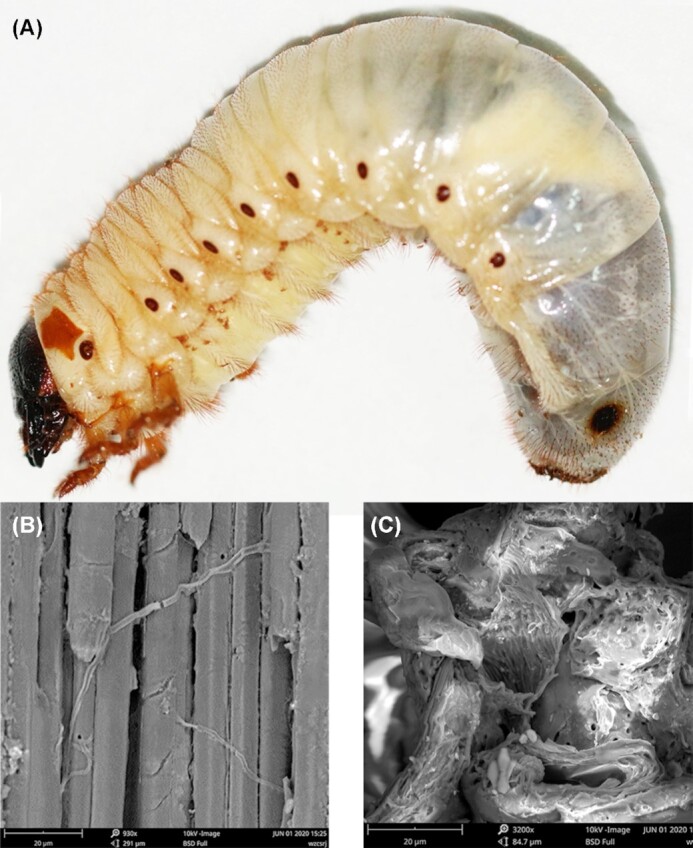
Wood fiber degradation by larvae of *Trypoxylus dichotomus*. (A) Third instar larva. (B) Wood fiber structure in sawdust. (C) Wood fiber structure after digestion of sawdust in larval excrement.

After intake and digestion by the third instar larvae (Fig. 1A, taken by a digital single-lens reflex camera), 6 larval excrement samples were randomly selected and photographed by an environment scanning electron microscope (ESEM). Compared to the intact wood fiber of sawdust before digestion (Fig. 1B, taken by ESEM), the wood fiber structures of sawdust were degraded into similar fragments after digestion by the larvae of *T. dichotomus* (Fig. 1C). Then, 6 third instar individuals were selected from each group and quickly rinsed twice using 75% alcohol. Because the foregut is small and short with weak digestion, and digestive activity mainly occurs in the midgut and hindgut [19], only the midgut and hindgut were separated and quickly rinsed twice with diethyl pyrocarbonate and then phosphate-buffered saline (1×) on a clean bench for dissection. After drying the surface liquid, 24 midgut and hindgut samples were preserved in liquid nitrogen, separately (Supplementary Table S2). All of the samples were divided into 4 groups: (i) midgut from sawdust (SM, midgut of larva feeding sawdust), (ii) hindgut from sawdust (SH, hindgut of larva feeding sawdust), (iii) midgut from mushroom residue (MM, midgut of larva feeding mushroom residue), and (iv) hindgut from mushroom residue (MH, hindgut of larva feeding mushroom residue). Each group consisted of 6 replicates.

Genomic DNA was extracted using a QIAGEN (Germany) genomic kit for short-insert (350 bp) and large-insert (>20 kb) library construction according to the manufacturer's instructions. Libraries were quantified using Qubit 3.0 fluorometry (Invitrogen, USA). Prior to genome sequencing, a *k*-mer distribution analysis was performed using genome survey sequences (GSS; Illumina (USA) DNA data) to estimate genome size and heterozygosity. The raw reads were filtered using the fastp (v.0.20.0) preprocessor [31] (set to default parameters) to remove low-quality reads, adapters, and reads containing poly-N. Briefly, quality-filtered reads were subjected to 17-mer frequency distribution analysis using the Jellyfish program [32]. By analyzing the 17-mer depth distribution from the 350-bp library cleaned sequencing reads, genome size and heterozygosity were estimated with FindGSE (skew normal distribution model) [33] and GenomeScope (negative binomial model) [34], separately. After genome estimation, a certain concentration (50 fmol) and volume (24 µL) of DNA library was transferred to a flow cell of PromethION (ONT, Oxford Nanopore Technologies (UK), FLO-PRO002 chip; RRID:SCR_017987) for whole-genome sequencing. Total RNA was extracted using the QIAGEN RNeasy Plus Universal Mini Kit, and ribosomal RNA (rRNA) was removed with QIAseq FastSelect RNA Remove Kits. It was qualified and quantified as follows: (i) RNA purity and concentration were examined using NanoDrop 2000, and (ii) RNA integrity and quantity were measured using the Agilent (UK) 2100 system. Sequencing libraries were generated using TruSeq RNA Library Preparation Kit v2 (Illumina) following the manufacturer's recommendations. The library preparations (350-bp target insert size) were sequenced on an Illumina Novaseq 6000 platform (Illumina; RRID:SCR_016387) to generate 150-bp paired-end reads, according to the manufacturer's instructions. All of the raw reads containing adapters and low-quality bases (“N” >10%, Q-value ≤20) were removed using fastp (fastp, RRID:SCR_016962).

### Genome assembly

The quality of reads was controlled using ONT Guppy (v3.2.2), referring to the value of mean_qscore_template ≥7, with the other parameters left at the defaults. Passed reads were assembled with NextDenovo (v2.0) (reads_cutoff: 1 k, seed_cutoff: 23 k). Raw data were aligned with the assembled genome using Minimap2 [35] (-x map-ont; RRID:SCR_018550) for sequence alignment information. Based on this information, the genome was corrected using Racon (v1.3.1; RRID:SCR_017642) in 3 iterations. The Illumina DNA data of the genome survey were filtered using fastp with default parameters. The corrected genomic data were polished with the filtered DNA data of the genome survey using Nextpolish (v1.0.5) over 4 iterations.

Possible contaminated sequences were detected using BLAST+ v2.9.1 [36] against the nt and UniVec databases and then removed. Scaffolds greater than 10 kb were retained and uploaded to NCBI for contamination detection in the final assembly. Comparing to the insecta_odb10 database in OrthoDB, a benchmarking universal single-copy ortholog (BUSCO, RRID:SCR_015008) analysis was performed to assess completeness of genome assembly using BUSCO v5.1.2 [37]. Moreover, to verify utilization of raw data and the completeness of genome assembly, the Illumina DNA data of the genome survey, Illumina RNA data of the male thorax, and ONT data were mapped to the genome assembly using Minimap2. Then, the mapping rates were calculated using SAMtools v1.9 (SAMTOOLS, RRID:SCR_002105) [38].

To anchor hybrid scaffolds onto the chromosome, genomic DNA was extracted from the thoracic muscle of the male individual. The Hi-C library was prepared, followed by a procedure [39] with improvement modifications. In brief, quick-freezing tissues of *T. dichotomus* were vacuum infiltrated in nuclei isolation buffer supplemented with 2% formaldehyde. Crosslinking was stopped by adding glycine and additional vacuum infiltration. Fixed tissue was then grounded into powders before resuspending in nuclei isolation buffer to obtain a suspension of nuclei. The purified nuclei were digested with 100 units of DpnII and marked by being incubated with biotin-14-dCTP. Biotin-14-dCTP from nonligated DNA ends was removed owing to the exonuclease activity of T4 DNA polymerase. The ligated DNA was sheared into 300- to 600-bp fragments and then blunt-end repaired and A-tailed, followed by purification through biotin-streptavidin–mediated pulldown. Finally, the Hi-C libraries were quantified and sequenced using the Illumina Novaseq platform according to the manufacturer's instructions. Quality control of Hi-C raw data and extraction of Hi-C contacts was performed using Juicer v1.6.2 (Juicer, RRID:SCR_017226) [40]. Hi-C contigs were anchored to pseu-chromosomes using 2 rounds of 3D-DNA v180922 [41] workflow. The initial assignment was manually corrected using Juicebox v1.11.08 [40] and then imported into 3D-DNA again to produce the final chromosome-anchored genome assembly, with the contigs separated by 100 Ns on the same chromosome.

### Genome annotation

A *de novo* repeat library was constructed using RepeatModeler v2.0.1 (RepeatModeler, RRID:SCR_015027) with a long terminal repeat (LTR) structural search [42] and then combined with the databases of Dfam_3.1 and RepBase-20181026 to generate a custom library. Repetitive elements (DNA/short interspersed nuclear element [SINE]/long interspersed nuclear element [LINE]/LTR) were searched by applying the program RepeatMasker v4.1.0 (RepeatMasker, RRID:SCR_012954) [43] based on the database of repeated sequences.

Protein-coding gene (PCG) structure was predicted in the pipeline of MAKER v3.01.03 (min_protein = 30, min_intron = 20) [44]. Three strategies were integrated for the prediction. (i) *Ab initio* gene structure prediction was made by applying the BRAKER v2.1.5 pipeline (BRAKER, RRID:SCR_018964) [45] together with self-training of Augustus v3.3.4 (Augustus, RRID:SCR_008417) [46] and GeneMark-ES/ET/EP 4.59_lic [47]. To improve the prediction accuracy, transcripts of thoracic muscle were optimized in the program bbduk.sh (qtrim = rl trimq = 20 minlen = 20 ecco = t maxns = 5 trimpolya = 10 trimpolyg = 10 trimpolyc = 10) in BBTools v38.82 [48]. Then, they were incorporated with protein homology-based evidence, in which transcriptome evidence in BAM alignments was produced using HISAT2 v2.2.0 (-dta) (HISAT2, RRID:SCR_015530) [49]. The arthropod protein source was mined from the OrthoDB10 v1 database [50]. (ii) With the BAM alignments inputted, transcripts of thoracic muscle were assembled using the genome-guided assembler StringTie v2.1.4 (StringTie, RRID:SCR_016323) [51]. (iii) Protein sequences for *Drosophila melanogaster* (Diptera), *Apis mellifera* (Hymenoptera), *Bombyx mori* (Lepidoptera), and beetles (*Tribolium castaneum, Onthophagus taurus, Anoplophora glabripennis*) were downloaded from NCBI and passed to MAKER as evidence of protein homology. The prepared files obtained from the above pipeline were imported into MAKER for integrated annotation.

Gene function was annotated with the following two strategies. (i) Gene functions were annotated by searching the protein sequence database UniProtKB using Diamond v0.9.24 (-more-sensitive -e 1e-5) [52]. (ii) Protein-conserved sequences and domains, Gene Ontology (GO), and pathways (KEGG, Reactome) were predicted by searching Pfam (Pfam, RRID:SCR_004726) [53], SMART (SMART, RRID:SCR_005026) [54], Gene3D [55], Superfamily [56], and CDD [57] using InterProScan 5.41–78.0 (InterProScan, RRID:SCR_005829) [58]. Simultaneously, their functions were predicted by searching the eggNOG v5.0 database [59] employing eggNOG-mapper v2.0.1 [60].

Noncoding RNAs (ncRNAs) were annotated with 2 strategies. (i) rRNAs, small nuclear RNAs (snRNAs), and microRNAs (miRNAs) were searched against the Rfam database using the program infernal v1.1.3 [61]. (ii) Transfer RNAs (tRNAs) were predicted using tRNAscan-SE v2.0.6 (tRNAscan-SE, RRID:SCR_010835) [62], with low-credibility tRNAs filtered out using the script “EukHighConfidenceFilter.” Based on the results of genome annotation, chromosome length, GC content, the density of PCGs, and repetitive elements on each pseudo-chromosome were plotted and visualized using Circos (v0.67–7; RRID:SCR_011798) [63].

### Comparative genomic and phylogenetic analysis

Gene family homology was inferred from protein sequences of 13 representative insect species downloaded from NCBI, including 8 beetles (*T. castaneum, Agrilus planipennis, Lamprigera yunnana, Nicrophorus vespilloides, O. taurus, Aethina tumida, Sitophilus oryzae*, and *A. glabripennis*) [64–69] and 5 other insect species (*D. melanogaster* [Diptera], *A. mellifera* [Hymenoptera], *B. mori* [Lepidoptera], *Coptotermes formosanus* [Blattodea], and *Rhopalosiphum maidis* [Hemiptera]) [70–74]. Gene families were identified by clustering protein sequences using OrthoFinder v2.3.8 (OrthoFinder, RRID:SCR_017118) [75] with Diamond [52] as the sequence aligner.

Phylogenetic trees were constructed with protein sequences of 1,260 single-copy orthologs, which were aligned with MAFFT v7.394 (MAFFT, RRID:SCR_011811) using the model “L-INS-I” [76]. The unreliable homologous regions were removed with BMGE v1.12 (-m BLOSUM90 -h 0.4) [77]. All of the well-aligned sequences were concatenated with FASconCAT-G v1.04 [78]. Maximum likelihood trees were constructed using IQ-TREE v2.0.7 [79] with the set of “-symtest-remove-bad -symtest-pval 0.10” for removing those genes not conforming to SRH (stationary, reversible, and homogeneous). The substitution model was constrained to LG with a heuristic partitioned search strategy “-m MFP -mset LG -msub nuclear -rclusterf 10,” and node support values were evaluated with ultrafast bootstrapping and SH-aLRT algorithms (-B 1000 -alrt 1000). The divergence time of phylogenies was estimated using r8s v1.81 [80]. Fossil calibration data were obtained from the PBDB database [81] and 2 published studies [82, 83], namely, root (Pterygota, <443.4 million years ago [mya]), Holometabola (315.2–382.7 mya), Lepidoptera + Diptera (Trichoptera, 311.4–323.2 mya), Coleoptera (307–323.2 mya), Scarabaeiformia (196.5–201.3 mya), Elateriformia (242–252 mya), and Cucujiformia (196.5–201.3 mya).

Expansions and contractions of gene families at each node of the evolutionary tree were estimated using CAFÉ v4.2.1 [84] under the stochastic gene birth–death model and default significance level (*P* = 0.01). For significantly expanded gene families, GO and KEGG functional enrichment analyses were performed using R package clusterProfiler v3.14.3 (clusterProfiler, RRID:SCR_016884) [85] with the default parameters. Forty-five rapidly expanded gene families were further selected and analyzed to understand the evolution of expanded gene families. Coding sequence (CDS) analysis of each gene family was performed using the PAML package of codeml [86] under the site models. Models applied in this step included M0 (one rate), M1a (neutral)–M2a (selection), and M7 (beta)–M8 (beta&ω) (NSsites = 0 1 2 7 8). A likelihood ratio test compared the results from the M1a–M2a and M7–M8 models (*P* = 0.05). Bayes empirical Bayes inference [87] was used for testing the positive loci in each gene family.

Chromosomal synteny was performed to investigate variation/conservation of chromosomes between *T. dichotomus* and the related beetle *T. castaneum* (Coleoptera: Tenebrionidae), whose genome was assembled at the chromosome level with 10 chromosomes (9 autosomal chromosomes and the X chromosome) [64]. Gene and protein sequences were aligned using MMseq2 v11-e1a1c [88] under the default parameters (-s 7.5 -alignment-mode 3 -num-iterations 4 -e 1e-5 -max-accept 5). Synteny analysis was performed using MCScanX [89] with the collinear block containing at least 5 homologous genes (-s 5 -e 1e-10). A chromosome synteny diagram was visualized using TBtools v1.0692 [90].

### Intestinal transcriptome analysis

Raw reads were further filtered by fastp to remove adapters and low-quality bases (“N” >10%, Q-value ≤20). The rRNA reads were found and removed by mapping short reads to the rRNA database of *T. dichotomus* with Bowtie2 (version 2.2.8; RRID:SCR_016368) [91]. The remaining clean reads were mapped to the reference genome using HISAT2 with “-rna-strandness RF” and other parameters set as the default. The mapped reads of each sample were assembled using StringTie in a reference-based approach. For each transcription region, a FPKM (fragment per kilobase of transcript per million mapped reads) value was calculated to quantify its expression abundance and variations, using RSEM software [92].

Based on FPKM, permutational multivariate analysis of variance (PERMANOVA) was performed with 999 permutations using the R package “vegan” [93]. Principal component analysis (PCA) and Pearson correlation coefficient (PCC) were performed with the R package gmodels [94]. Differential gene expression analysis was performed with DESeq2 (DESeq2, RRID:SCR_015687) [95] software, with a shrinkage estimator for dispersion between different gut tissues from the same diet, or the same gut tissues from different diets. The genes associated with a false discovery rate below 0.05 and absolute fold change ≥2 (|FC| ≥ 2) were considered differentially expressed genes. All differentially expressed digestion-related genes were further annotated with KEGG pathways and GO terms. Digestion-related genes were then filtered to exclude those with mean gene counts fewer than 5 within a group for all groups. A heatmap of differentially expressed digestion-related genes was visualized using TBtools.

## Results and Discussion

### Genome estimation

Before ONT sequencing, 25 Gb (more than 40×) of Illumina DNA data from a genome survey with GC content of 35.85% were obtained for sample quality and genome assessment (Supplementary Table S3). By analyzing the 17-mer depth distribution from the 350-bp library cleaned sequencing reads, the genome size and repeat ratio of *T. dichotomus* were estimated to be 630.93 Mb and 32.29% in FindGSE and 567.40 Mb and 22.99% in GenomeScope (Supplementary Fig. S1; Supplementary Table S4). Further combined with the simulation results, the final genome size of *T. dichotomus* was estimated to be about 599.17 Mb, with a 2.09% heterozygous ratio.

The N50 and the mean length of the long reads were 24.54 and 16.88 Kb, respectively, with the longest read of 170.57 Kb. Furthermore, 12 Gb of Illumina RNA data were obtained from thoracic muscle for genome evaluation and annotation (Supplementary Table S3).

### Genome assembly and assessment

**Figure 2:**
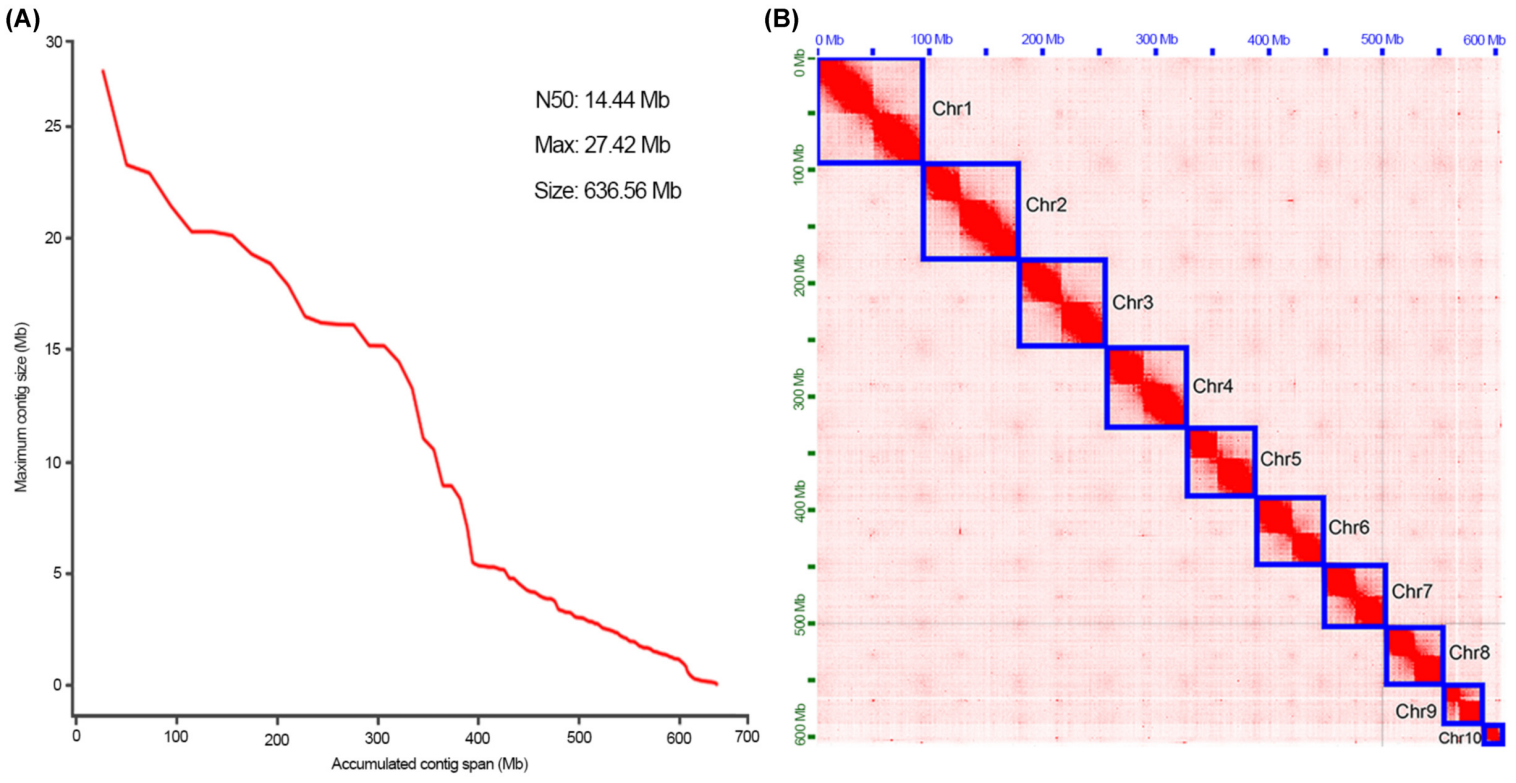
Genome assembly and assessment of *Trypoxylus dichotomus*. (A) Accumulated graph of contig length. (B) Hi-C heatmap showing 10 chromosomes (Chr1 to Chr10) arranged by length.

ONT sequencing generated 73 Gb (approximately 120×) of pass reads (Supplementary Table S3), which were then corrected by the NextCorrect module (NextDenovo) and produced 45 Gb of consensus sequences. The preliminary assembly was generated using the NextGraph module (NextDenovo), with the genome size of 634.66 Mb and N50 length of 14.42 Mb. After being corrected and polished by Racon and Nextpolish, the polished genome size was 636.56 Mb, with the scaffold N50 length of 14.44 Mb (Table 1), suggesting a good continuity of our assembled genome (Fig. 2A).

**Table 1:** Genome assembly and quality evaluation

**Assembly**	**Total length (Mb)**	**Number of scaffolds**	**N50 length (Mb)**	**Longest scaffold (Mb)**	**GC (%)**	**BUSCO (*n* = 1367) (%)**			
						**C**	**D**	**F**	**M**
NextDenovo	636.56	304	14.44	27.42	35.12	98.8	0.9	0.8	0.4
3D-DNA	636.61	496	71.04	94.63	35.12	98.7	0.8	0.9	0.4
Final	636.27	417	71.04	94.63	35.11	98.7	0.8	0.9	0.4

C, complete BUSCOs; D, complete and duplicated BUSCOs; F, fragmented BUSCOs; M, missing BUSCOs.

The genome of *T. dichotomus* was further sequenced by NovaSeq sequencing, which generated 83 Gb of Hi-C data (Supplementary Table S3) and was filtered to produce 79 Gb of clean data. Based on the clean data in the 3D-DNA analysis, the chromosome-anchored genome size was estimated to be 636.61 Mb, with 496 scaffolds and an N50 length of 71.04 Mb (Table 1). After polishing, removing redundancy and contaminants, and Hi-C scaffolding, the final genome size was determined to be 636.27 Mb, composed of 417 scaffolds, with a scaffold/contig N50 length of 71.04/12.99 Mb, GC content of 35.11%, and gaps of 0.004% (Supplementary Table S5), which was close to the earlier genome estimation by FindGSE. Furthermore, 606.8 Mb scaffolds covering 95.37% of the draft reference genome were precisely anchored onto 10 pseudo-chromosomes (Fig. 2B), indicating a high quality of the chromosome-level genome assembly.

Taking all the published genomes of Scarabaeidae into account, we found that the genomic characteristics varied significantly among the 8 retrievable genomes of scarabaeid beetles, with a genome size of 267–1,144 Mb [96–98]. A draft genome assembly of *T. dichotomus* was recently released in GenBank (Bioproject: PRJDB10500; genome size of 739.41 Mb; contig N50 length of 7.93 Mb; contig number of 2,347) without further analysis. Its BUSCO assessment (*n* = 1,367) identified 1,352 (98.9%) complete BUSCOs, comprising 1,340 (98.0%) single-copy and 12 (0.9%) duplicated BUSCOs. In comparison, the size of our genome was smaller than that of the released genome assembly, probably due to the scaffold assembly level we used. Furthermore, our genome assembly showed a longer scaffold N50 (71.04 Mb) and a smaller scaffold number (414) than that of the released one. We found that *T. dichotomus* has a relatively larger genome than most other scarabaeids, but a similar GC content close to 35% (except 25% for *Protaetia brevitarsis*, Bioproject: PRJNA477715). The clearest example of genomic difference was found in its closest relative species from the same subfamily, *O. taurus*, with a much smaller genome size of 267.08 Mb (Bioproject: PRJNA419349).

Using BUSCO assessment (*n* = 1,367), we identified 1,349 (98.7%) conserved orthologous as complete genes, with 97.9% “complete and single-copy BUSCO” and 0.8% “complete and duplicated BUSCO” genes represented (Table 1). The mapping rates of Illumina DNA data from the genome survey, Illumina RNA data of the male thorax, and ONT data onto our draft genome were as high as 99.89%, 95.39%, and 99.60%, respectively. These results indicate that the genome assembly of *T. dichotomus* in this study reached an extremely high quality in both continuity and integrity.

### Genome annotation

**Figure 3:**
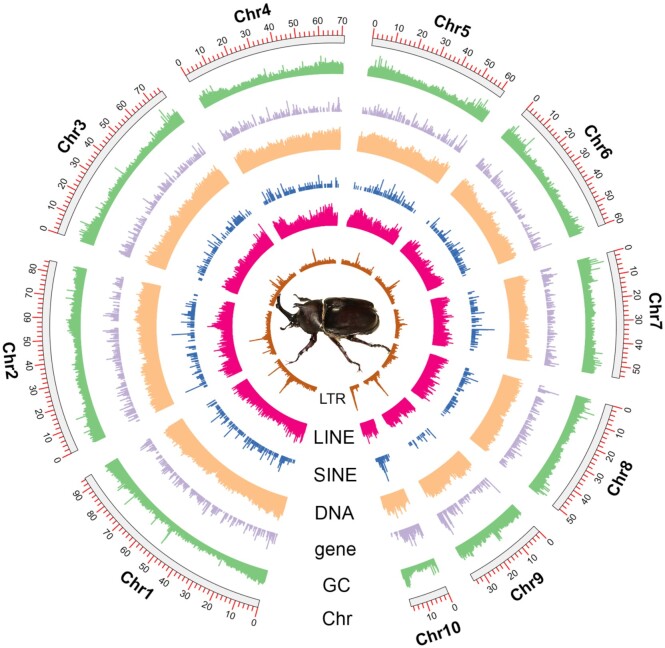
Circos graph of chromosome-level genome of *Trypoxylus dichotomus*, showing length of chromosomes, GC content, density of protein-coding genes, and repetitive elements (DNA/SINE/LINE/LTR). (Sliding window size = 100 kb)

A total of 1,369,555 repeat sequences (365,506,399 bp) were identified, accounting for 57.45% of the whole genome, with the top 6 represented as DNA elements (28.97%), unclassified (16.67%), LINEs (9.69%), LTRs (1.24%), SINEs (0.52%), and simple repeats (0.52%) (Supplementary Table S6). The density of each type (except unclassified) was shown on each chromosome, indicating that DNA elements and LINEs have the maximum densities (Fig. 3).

To predict the genes in *T. dichotomus*, we employed MAKER pipeline and generated 12,193 PCGs, among which the average length of genes, CDS, and transcripts was 15,150, 1,743, and 2,355 bp, respectively. On average, the size of the exons and introns was 339 and 1,857 bp, respectively (Supplementary Table S5), which is common in organisms with large genomes [99]. Furthermore, BUSCO assessment (*n* = 1,367) identified 95.8% (S: 85.4%, D: 10.4%) of the conserved orthologous as complete genes in the predicted PCGs, indicating that our prediction was relatively complete.

After PCG functional annotation, 11,551 (94.73%) genes were detected matching the UniprotKB records by Diamond, while 10,640 (87.26%) protein domains of PCGs were identified using InterProScan. In addition, we also identified 10,535 GO, 8,224 KEGG ko, 2,886 enzyme codes, 9,431 KEGG pathways, 10,590 reactome pathways, and 12,025 COG categories by InterProScan and eggNOG-mapper. To evaluate these data sets, we compared them with other high-quality genome annotations from 6 insects and revealed more than 10,000 hits (Table 2).

**Table 2:** Gene hits between *Trypoxylus dichotomus* and another 6 insects

**Species**	**Gene number**	**Hit number**
*Trypoxylus dichotomus*	12,193	—
*Onthophagus taurus*	15,366	11,329
*Tribolium castaneum*	12,657	11,178
*Anoplophora glabripennis*	14,698	11,144
*Apis mellifera*	12,739	10,365
*Bombyx mori*	13,683	10,381
*Drosophila melanogaster*	13,617	10,135

Based on the annotation by the Rfam database and tRNAscan-SE, we identified 668 ncRNAs in the genome, including 43 rRNAs, 57 miRNAs, 129 snRNAs, 2 long noncoding RNAs, 2 ribozymes, 361 tRNAs, and 74 other ncRNAs. Twenty-one isotypes of tRNAs were annotated in this species, but the Supres isotype was missing. We also identified 129 snRNAs, with 106 spliceosomal RNAs (U1, U2, U4, U5, U6, and U11), 5 minor spliceosomal RNAs (U4atac, U6atac, and U12), 14 C/D box snoRNAs (small nucleolar RNAs), 3 H/ACA box snoRNAs, and 1 other snRNA (SCARNA8) (Supplementary Table S7).

### Comparative genome and phylogeny

#### Gene family identification

**Figure 4:**
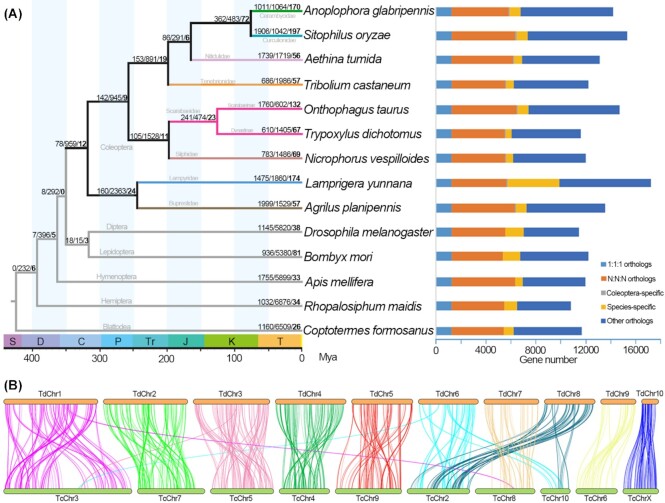
(A) Phylogenetic tree and statistics of orthologs. Left: Phylogenetic tree and divergence times of beetles based on 1,108 single-copy orthologs; branch values representing the number of expanded, contracted, and rapidly evolving gene families (bold) respectively; color value scale representing divisions of geologic time, abbreviations standing for Silurian (S), Devonian (D), Carboniferous (C), Permian (P), Triassic (Tr), Jurassic (J), Cretaceous (K), and Tertiary (T). Right: statistics of orthologous genes among the 14 insect species; “1:1:1” representing shared single-copy genes, “N:N:N” representing multicopy genes shared by all species, “Coleoptera” representing orthologs unique to Coleoptera, and “Others” representing unclassified orthologs. (B) Chromosome-level genome synteny between *Trypoxylus dichotomus* and *Tribolium castaneum*; “TdChr” representing chromosomes of *T. dichotomus*, “TcChr” representing chromosomes of *T. castaneum*.

Using homology analysis of the gene family, 181,904 (92.90%) genes were clustered into 14,467 orthogroups (gene families), in which 12,658 orthogroups were unique to beetles. Moreover, there were 1260 single-copy orthogroups and 3,120 multicopy orthogroups identified for *T. dichotomus*. Among the PCGs in the genome of this beetle, 11,614 (95.25%) genes were clustered into 8,727 orthogroups, in which 107 orthogroups/488 genes were specific to *T. dichotomus* (Table 3, Fig. 4A).

**Table 3:** Statistics of gene families among 14 insects

**Category**	**Number**
Number of species	14
Number of genes	195,765
Number of genes in orthogroups	181,904
Number of unassigned genes	13,861
Percentage of genes in orthogroups	92.9
Number of orthogroups	14,467
Number of species-specific orthogroups	3,396
Number of genes in species-specific orthogroups	15,299
Percentage of genes in species-specific orthogroups	7.8
Mean orthogroup size	12.6
Number of orthogroups with all species present	4,380
Number of single-copy orthogroups	1,260

#### Phylogeny and gene family evolution

After removing 152 single-copy orthologs using symtest, the remaining 1,108 single-copy orthologs (450,544 amino acids) were concatenated for the phylogenetic tree construction (Fig. 4A). The phylogenetic relationships of 14 insect species were well recovered [83, 100], with all the nodes being strongly supported (UFB/SH-aLRT = 100/100), showing a good resolution in the phylogram. Coinciding with the previous beetle phylogenomic study [100], our results indicated that Coleoptera originated in the Early Carboniferous (320 mya), while the split of the ancestors of *T. dichotomus* and its closely related scarabaeid species *O. taurus* occurred in the early Cretaceous (120 mya) (Supplementary Fig. S2).

**Figure 5:**
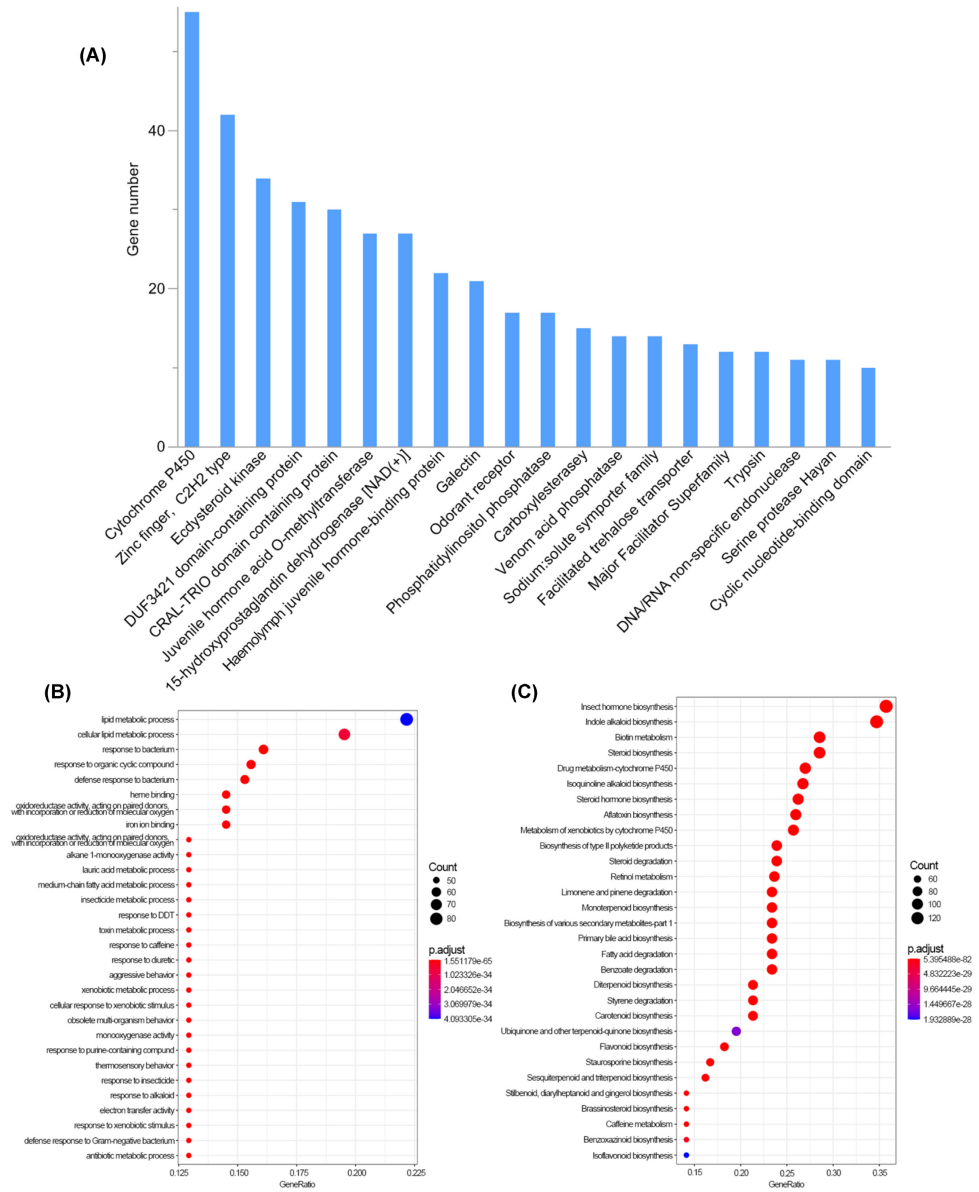
Expanded gene families and functional enrichment. (A) Top 20 significantly expanded gene families. (B) GO enrichment of rapidly expanded gene families. (C) KEGG enrichment of rapidly expanded gene families.

To investigate the rapidly evolving gene families in *T. dichotomus*, we used gene family evolution analysis and revealed that 610 and 1,405 gene families had experienced expansions and contractions, respectively, in which 67 gene families (45 expansions and 22 contractions) were recognized as rapidly evolving orthogroups (Fig. 4A). The significantly expanded gene families were primarily associated with digestion (trypsin, enoyl-[acyl carrier protein] reductase), detoxification (cytochrome P450, ecdysteroid kinase, carboxylesterase, aldo/keto reductase), chemoreception (odorant receptor, gustatory receptor), glycometabolism (facilitated trehalose transporter, neutral alpha-glucosidase), immunity (15-hydroxyprostaglandin dehydrogenase [NAD(+)], galectin, serine protease Hayan, prostaglandin reductase 1, inducible metalloproteinase inhibitor protein), development (hemolymph juvenile hormone binding protein, juvenile hormone acid O-methyltransferase, serine protease snake), and toxoprotein (venom acid phosphatase) (Fig. 5A; Supplementary Table S8).

The rapidly expanded gene families were further confirmed in the GO and KEGG enrichments (Supplementary Tables S9 and S10), with metabolic detoxification, digestion, and immunity mainly in the GO enrichment (Fig. 5B) and metabolic detoxification, digestion, juvenile hormone, and secondary metabolite synthesis mainly in the KEGG pathway (Fig. 5C). Four gene families were positively selected, including serine protease Hayan (OG0000411), phosphatidylinositol phosphatase (OG0001456), Hsp70 protein (OG0009015), and nucleoporin autopeptidase (OG0009016), which were related to immunity, cell proliferation/differentiation, heatshock proteins, and nucleo-cytoplasmic transport, respectively (Supplementary Table S11). These results indicated that digestion and detoxification were significantly reflected in the rapidly expanded gene families and functional enrichment in the *T. dichotomus* genome.

Most beetles were considered not to capitalize on their significant ability for endogenous lignocellulose digestion [101], but this is not the case for *T. dichotomus*. Our results revealed that the functional capacity of digestion was obviously reinforced by expansions of digestion-related gene families [102] in the evolution of *T. dichotomus*, which would greatly promote lignocellulose digestion. Additionally, the detoxification function was also reinforced by gene family expansion and positive selection, suggesting an adaptive evolution responding to environmental exposures [103, 104]. This was further supported by the diversification of expression patterns of *T. dichotomus* that adapted to different humus resources [105].

#### Synteny

To investigate the chromosomal evolution in *T. dichotomus*, we carried out a synteny analysis and generated 262 collinear blocks based on 4,477 collinear genes (18.69% of all genes), with 6–23 genes in each block (Supplementary Table S12). Chromosomes 1–7 and 9–10 of *T. dichotomus* (TdChr1–7 and 9–10) were mapped to chromosomes 3, 7, 5, 4, 9, 2, 8, 6, and X of *T. castaneum* (TcChr3, 7, 5, 4, 9, 2, 8, 6, and X), with strong syntenic relationships. Only chromosome 8 of *T. dichotomus* (TdChr8) showed a relatively low synteny with chromosome 10 of *T. castaneum* (TcChr10) (Fig. 4B). These results indicated a high genome synteny between *T. dichotomus* and *T. castaneum*, which clearly reveals an overall conservation of chromosomes in *T. dichotomus* [106]. Furthermore, TdChr10 was mapped to TcChrX perfectly (Fig. 4B), suggesting that TdChr10 was the X chromosome in *T. dichotomus* [107].

Collinear genes were intersected within homologous chromosomes extensively (Fig. 4B), indicating a common reshuffling of gene orders within chromosomes, that is, intrachromosomal rearrangements (inversions) [108]. In contrast, collinear genes were occasionally intersected among nonhomologous chromosomes, with only 5 pairs of interchromosomal rearrangements (translocations) (TdChr1–TcChr8, TdChr3–TcChr3, TdChr6–TcChr3, TdChr6–TcChr10, and TdChr8–TcChr2) [109]. Notably, TdChr6 and 8 were significantly intersected with TcChr2 and 10, respectively, indicating a wide variety of chromosome breakages and rearrangements [108] during the evolutionary history of *T. dichotomus*.

Although the clades of *T. dichotomus* (Scarabaeoidea) and *T. castaneum* (Tenebrionoidea) diverged in the late Permian (Fig. 4A), their chromosomes (autosomes and X chromosome) were conserved on account of the relatively limited translocations, which might indicate relative conservation of chromosomes in the evolutionary history of beetles, at least to some extent. In contrast to the autosomes, X chromosome was considered more conserved and more recalcitrant to rearrangement than that of the autosomes in insects [106, 109–111], which is consistent with our results for *T. dichotomus*. Therefore, we assume that the intrachromosomal rearrangements are possibly the main evolutionary force for beetles, and autosome rearrangements may be the most important factor. Nevertheless, despite the occasional occurrences, interchromosomal rearrangements of autosomes might also play a vital role in the evolutionary process of beetles.

### Gene expression and sample correlation

**Figure 6:**
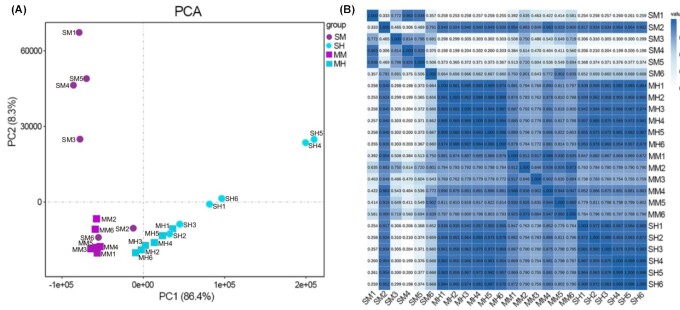
Sample correlation of intestinal gene expression patterns among 4 groups of *Trypoxylus dichotomus*. Each group consists of 6 replicates. (A) Principal component analysis (PCA) diagram; circle indicates larva feeding sawdust, square indicates larva feeding mushroom residue, green indicates midgut, and red indicates hindgut. (B) Pearson correlation coefficient (PCC) heatmap; colors and values indicate the relationship between paired samples (the darker the color and larger value mean, the closer the relationship); value ≥0.8 shows the good repeatability. MH, hindgut from mushroom residue; MM, midgut from mushroom residue; SH, hindgut from sawdust; SM, midgut from sawdust.

To further explore the intestinal gene expression patterns associated with different gut tissues and food habits, we carried out intestinal transcriptome analysis for the larvae of *T. dichotomus*. Based on the gene expression (FPKM) of all annotated genes for each sample by PERMANOVA, we found significant differences of gene expressions between the groups separated by gut tissues or food habits (Table 4). PCA and PCC (Supplementary Tables S13 and S14) were then used to calculate and plot diagrams (Fig. 6), respectively. With PCA analysis (Fig. 6A), we showed that samples from the same group were mainly aggregated together, except for 4 outliers (SM2, SM6, SH2, and SH3) in the midgut and hindgut of sawdust feeding beetles. Similarly, PCC analysis (Fig. 6B) also displayed good repeatability within most of the intragroups, but a relatively low level of repeatability in the midgut of sawdust feeding larvae was due to the abnormal values of SM2.

**Table 4:** Permutational multivariate analysis of variance (PERMANOVA) among groups separated by gut tissue and food habit

**Groups**		**Mean squares**	***df***	***R* ^2^**	***P***
Tissue	SM vs. SH	0.91	1	0.63	**
	MM vs. MH	0.56	1	0.77	**
Food	SM vs. MM	0.30	1	0.42	**
	SH vs. MH	0.23	1	0.46	**

MH, hindgut from mushroom residue; MM, midgut from mushroom residue; SH, hindgut from sawdust; SM, midgut from sawdust. **P < 0.01.

For the groups with the same food habits (SM and SH, MM and MH), more significant differences of gene expressions were observed between the midgut and hindgut in the sawdust groups than in the mushroom residue groups along PC1 and PC2. Furthermore, there were also significant differences between groups within the same gut tissue (SM vs. MM, SH vs. MH), suggesting that intestinal gene expressions could be significantly affected by food habits in *T. dichotomus*. Consistently, it was reported that different host diets could significantly affect the digestive physiology of the beetle, *Trogoderma granarium* [28].

### Differentially expressed digestion-related genes

**Figure 7:**
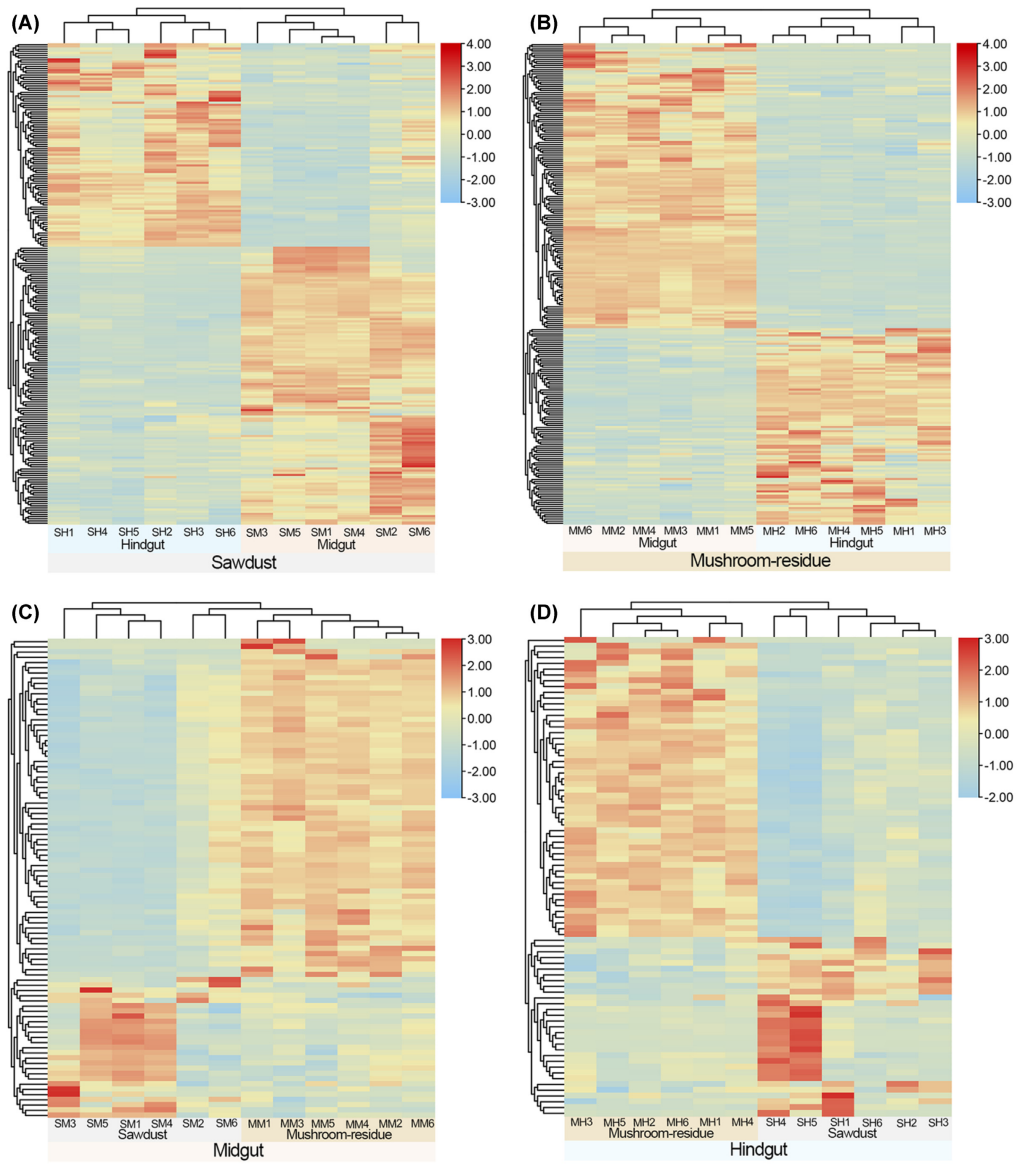
Heatmaps of differentially expressed digestion-related genes among 4 groups of the rhinoceros beetle. Each group consists of 6 replicates. Colors indicate a higher (red) or lower (blue) gene expression in each sample for every gene, identified by the FPKM value. Gene expression clustering between midgut and hindgut from sawdust group (A) and mushroom residue group (B). Gene expression clustering of midgut (C) and hindgut (D) between sawdust and mushroom residue groups. MH, hindgut from mushroom residue; MM, midgut from mushroom residue; SH, hindgut from sawdust; SM, midgut from sawdust.

To understand the digestive ability of *T. dichotomus* larvae on different gut tissues and food habits, digestion-related genes were filtered (Supplementary Table S15) and differentially expressed genes were further compared within 4 different treatment groups (i.e., SM vs. SH, MM vs. MH, SM vs. MM, and SH vs. MH) (Supplementary Table S16, Fig. 7). A total of 222 differentially expressed digestion-related genes were identified in the midgut and hindgut from the sawdust groups, in which 128 and 94 genes were highly expressed in the midgut and hindgut (SM vs. SH), respectively (Fig. 7A). Similarly, 231 differentially expressed digestion-related genes were detected in the midgut and hindgut from mushroom residue groups, among which 137 and 94 genes were highly expressed in the midgut and hindgut (MM vs. MH), respectively (Fig. 7B). These results indicate that more digestion-related genes are highly expressed in the midgut than the hindgut of larvae, regardless of food habit. Thus, the digestion of lignocellulose in larvae may require more digestive enzymes in the midgut than in the hindgut. To some extent, this is consistent with previous studies showing that polysaccharide degradation occurs mainly in the midgut of the rhinoceros beetle [19, 27].

The highly expressed digestion-related genes in the gut varied between the sawdust and mushroom residue groups. Of the 92 differentially expressed digestion-related genes in the midguts from 2 different food habits, 65 and 27 genes were highly expressed in the mushroom residue group and the sawdust group (SM vs. MM), respectively (Fig. 7C). Similarly, 83 differentially expressed digestion-related genes were detected in the hindguts, among which 52 and 31 genes were highly expressed in the mushroom residue group and the sawdust group (SH vs. MH), respectively (Fig. 7D). Taken together, more digestion-related genes were highly expressed in the mushroom residue group than in the sawdust group regardless of whether the specific location was the midgut or hindgut. These results suggest that digestion of mushroom residue might require a greater digestive ability than that of sawdust for the larvae of *T. dichotomus*, which is probably due to the complex components of mushroom residue, including not only wood fiber but also fungal mycelia.

The rhinoceros beetle may serve as an efficient decomposer in lignocellulose-enriched agro-forestry residues, including mushroom residue and decaying wood, which would provide an environmentally friendly method for sustainable development. In the forest, the larvae of *T. dichotomus* usually inhabit soil organic matter and feed on decayed wood [19, 20, 27]. This is similar to the living and feeding habitats of the white-spotted flower chafer, *Protaetia brevitarsis* (Scarabaeidae), which also efficiently digests high lignocellulosic mushroom residue [112]. Interestingly, both species were often observed coexisting in the outdoor mushroom residue, showing that these 2 scarab beetles might share an overlapping ecological niche and promote more effective lignocellulosic degradation through close cooperation.

## Conclusion

In this study, we assembled and provided the chromosome-level genome of *T. dichotomus* in the family Scarabaeidae. Combing different assembling methods, we concluded the final genome size to be 636.27 Mb with the BUSCO completeness up to 98.7%, indicating a high quality of our genome assembly. Furthermore, 95.37% scaffolds in the draft genome were anchored onto 10 chromosomes, and chromosome 10 was further identified as the X chromosome (sex chromosome) of *T. dichotomus*. In addition, the result of synteny analysis showed that chromosomes 6 and 8 of *T. dichotomus* were intersected with chromosomes 2 and 10 of *T. castaneum*, revealing that chromosome breakages and rearrangements evolutionarily occurred in *T. dichotomus*. Based on 1,108 single-copy orthologs, the phylogenetic relationships of the beetles were recovered, showing that the ancestor of *T. dichotomus* diverged in the early Cretaceous (120 mya) from that of the closely related species *O. taurus*.

Interestingly, gene families that associated with digestion and detoxification were significantly expanded in the evolutionary history of *T. dichotomus*, indicating improved adaptation to the local environment by the rhinoceros beetle. This is supported by the high degradation efficiency of lignocellulosic biomass and extensive adaptability to humus environment at the larval stage. Through a comparative analysis of intestinal transcriptome of larvae feeding on sawdust and mushroom residue, we found that intestinal gene expressions could be significantly affected by food habits in *T. dichotomus*. Digestion-related genes were more commonly expressed in the midgut or mushroom residue group than hindgut or sawdust group, despite different food treatments or gut tissue treatments. In conclusion, chromosome-level genome assembly and larval intestinal transcriptome analyses will facilitate future genetic studies on the lignocellulose degradation in *T. dichotomus*, as well as effective utilization of *T. dichotomus* in the eco-friendly biotreatment of plant biomass. Furthermore, the well-assembled and annotated genomic data in this study will provide a valuable resource for further understanding the evolutionary history of beetles and the functions of specific genes.

## Data Availability

The data sets supporting the results of this article are available in the GenBank repository. The whole-genome sequencing and assembly project has been deposited at GenBank (NCBI BioProject: PRJNA688811). The chromosome-level genome assembly of *Trypoxylus dichotomus* has been stored in the NCBI database under accession no. JAENHH000000000. All the sequencing raw data, including genome survey, Nanopore, Hi-C, and RNA sequencing, have been submitted to the BioProject PRJNA688811. All supporting data and materials are available in the *GigaScience* GigaDB database [113].

## Additional Files

**Figure S1:** K-mer distribution curve.

**Figure S2:** Phylogenetic tree and divergence times of beetles based on 1,260 single-copy orthologs. Node values representing divergence times.

**Table S1:** Sample information of *Trypoxylus dichotomus*.

**Table S2:** Transcriptome sequencing sample information.

**Table S3:** Genome sequencing data statistics.

**Table S4:** Genome estimation.

**Table S5:** Genome assembly and annotation statistics.

**Table S6:** Repeat annotation.

**Table S7:** Annotations of non-coding RNAs.

**Table S8:** Rapidly expanded gene families and functions.

**Table S9:** GO enrichment.

**Table S10:** KEGG enrichment.

**Table S11:** Ka/Ks values of forty-five rapidly expanded gene families (M0 model).

**Table S12:** Collinearity analysis between *Trypoxylus dichotomus* and *Tribolium castaneum*.

**Table S13:** PC values of all the samples based on the expression level of larval intestinal transcriptome.

**Table S14:** Pearson values of all samples based on the expression level of larval intestinal transcriptome.

**Table S15:** Expression of digestion-related genes in transcripts referring to KEGG pathways and GO terms.

**Table S16:** Comparison of differentially expressed digestion-related genes among different groups.

## List of Abbreviations

BUSCO: Benchmarking Universal Single-Copy Orthologs; CDS: coding sequence; ESEM: environment scanning electron microscope; FC: fold change; FPKM: fragment per kilobase of transcript per million mapped reads; GO: Gene Ontology; GSS: genome survey sequences; KEGG: Kyoto Encyclopedia of Genes and Genomes; LINE: long interspersed nuclear element; LTR: long terminal repeat; MH: hindgut of larva feeding mushroom residue; miRNA: microRNA; MM: midgut of larva feeding mushroom residue; ncRNA: noncoding RNA; PCA: principal component analysis; PCC: Pearson correlation coefficient; PCG: protein-coding gene; PERMANOVA: permutational multivariate analysis of variance; rRNA: ribosomal RNA; SH: hindgut of larva feeding sawdust; SINE: short interspersed nuclear element; SM: midgut of larva feeding sawdust; snRNA: small nuclear RNA; SRH: stationary, reversible, and homogeneous.

## Competing Interests

The authors declare that they have no competing interests.

## Funding

This work was supported by Cooperation Project of Zhejiang Province and Chinese Academy of Forestry (Grant No. 2020SY08).

## Authors' Contributions

Q.W.: methodology, software, validation, formal analysis, investigation, data process, writing—original draft, visualization; J.H.: conceptualization, resources, writing—review & editing, project administration, funding acquisition; L.L.: conceptualization, writing—review & editing; S.Z.: resources, writing—review & editing; H.W.: supervision.

## Supplementary Material

giac059_GIGA-D-21-00415_Original_Submission

giac059_GIGA-D-21-00415_Revision_1

giac059_GIGA-D-21-00415_Revision_2

giac059_Response_to_Reviewer_Comments_Original_Submission

giac059_Response_to_Reviewer_Comments_Revision_1

giac059_Reviewer_1_Report_Original_Submission Julian Dupuis -- 1/21/2022 Reviewed

giac059_Reviewer_1_Report_Revision_1 Julian Dupuis -- 4/12/2022 Reviewed

giac059_Reviewer_1_Report_Revision_2 Julian Dupuis -- 5/10/2022 Reviewed

giac059_Reviewer_2_Report_Original_Submission Christopher Cunningham -- 1/23/2022 Reviewed

giac059_Reviewer_2_Report_Revision_1 Christopher Cunningham -- 4/8/2022 Reviewed

giac059_Supplemental_Files
