# Supplementary material for: A chromosome-level genome assembly and intestinal transcriptome of Trypoxylus dichotomus (Coleoptera: Scarabaeidae) to understand its lignocellulose digestion ability
Source: Gigascience. 2022 Jun 28;11:giac059. doi: 10.1093/gigascience/giac059 (PMC9239855; doi:10.1093/gigascience/giac059)
Supplement: giac059_GIGA-D-21-00415_Original_Submission [file giac059_giga-d-21-00415_original_submission.pdf]

GigaScience

A chromosome-level genome assembly and intestinal transcriptome of Trypoxylus dichotomus (Coleoptera: Scarabaeidae) to understand its lignocellulose digestion ability

--Manuscript Draft--

**Manuscript Number:**	GIGA-D-21-00415
**Full Title:**	A chromosome-level genome assembly and intestinal transcriptome of Trypoxylus dichotomus (Coleoptera: Scarabaeidae) to understand its lignocellulose digestion ability
**Article Type:**	Research
**Funding Information:**	Cooperation Project of Zhejiang Province and Chinese Academy of Forestry (2020SY08) — Dr. Junhao Huang
**Abstract:**	Lignocellulose, as the key structural component of plant biomass, is of recalcitrant structure and is difficult to degrade. Meanwhile, the improper handling of plant residues is accelerating the process of global warming. Interestingly, we noticed that the xylophagous beetle, *Trypoxylus dichotomus* has a significant ability to decompose lignocellulosic biomass. However, no study has been conducted to elucidate the digestion mechanism from a genome-wide aspect for this beetle. Based on sequencing and assembling, the draft genome size of *T. dichotomus* is 636.27 Mb, with 95.37% scaffolds anchored onto 10 chromosomes. The phylogenetic results indicated that *T. dichotomus* and its closely related scarabaeid species *Onthophagus taurus* split from each other in the early Cretaceous. Furthermore, two digestive gene families (Trypsin and Enoyl-(Acyl carrier protein) reductase) have experienced significant expansion, accounting for the high degradation efficiency of lignocellulose. Additionally, the collinearity analysis revealed that chromosome breakages and rearrangements occur in the evolution of *T. dichotomus* due to chromosomes 6 and 8 of *T. dichotomus* being intersected with chromosomes 2 and 10 of *Tribolium castaneum* respectively. As suggested by the larval intestinal transcriptome comparative analyses, the digestive ability of midgut is much stronger than that of hindgut, even though susceptible to different food habits. This study reported the well-assembled and annotated genome of this rhinoceros beetle, providing genomic and transcriptomic bases for further understanding the functional mechanism and evolutionary history of lignocellulolytic digestion of the beetle.
**Corresponding Author:**	Junhao Huang<br>Zhejiang A&F University<br>Hangzhou, Zhejiang CHINA
**Corresponding Author Secondary Information:**	
**Corresponding Author's Institution:**	Zhejiang A&F University
**Corresponding Author's Secondary Institution:**	
**First Author:**	Qingyun Wang
**First Author Secondary Information:**	
**Order of Authors:**	Qingyun Wang<br>Liwei Liu<br>Sujiong Zhang<br>Hong Wu<br>Junhao Huang
**Order of Authors Secondary Information:**	
**Additional Information:**	

Powered by Editorial Manager® and ProduXion Manager® from Aries Systems Corporation

Question	Response
Are you submitting this manuscript to a special series or article collection?	No
**Experimental design and statistics**<br><br>Full details of the experimental design and statistical methods used should be given in the Methods section, as detailed in our Minimum Standards Reporting Checklist. Information essential to interpreting the data presented should be made available in the figure legends.<br><br>Have you included all the information requested in your manuscript?	Yes
**Resources**<br><br>A description of all resources used, including antibodies, cell lines, animals and software tools, with enough information to allow them to be uniquely identified, should be included in the Methods section. Authors are strongly encouraged to cite Research Resource Identifiers (RRIDs) for antibodies, model organisms and tools, where possible.<br><br>Have you included the information requested as detailed in our Minimum Standards Reporting Checklist?	Yes
**Availability of data and materials**<br><br>All datasets and code on which the conclusions of the paper rely must be either included in your submission or deposited in publicly available repositories (where available and ethically appropriate), referencing such data using a unique identifier in the references and in the "Availability of Data and Materials" section of your manuscript.	Yes

Have you have met the above requirement as detailed in our Minimum Standards Reporting Checklist?	

Powered by Editorial Manager® and ProduXion Manager® from Aries Systems Corporation

Manuscript

Click here to access/download;Manuscript;Chromosome-level genome of Trypoxylus dichotomus.docx

A chromosome-level genome assembly and intestinal transcriptome of *Trypoxylus dichotomus* (Coleoptera: Scarabaeidae) to understand its lignocellulose digestion ability

Qingyun Wang[a], Liwei Liu[a,b], Sujiong Zhang[c], Hong Wu[a], Junhao Huang[a]*

[a] National Joint Local Engineering Laboratory for High-Efficient Preparation of Biopesticide, Zhejiang A&F University, 666 Wusu Street, Lin'an, Hangzhou, Zhejiang 311300, China

[b] Zhejiang Museum of Natural History, No.6 West Lake Cultural Square, Hangzhou, Zhejiang 310014, China

[c] Dapanshan Insect Institute of Zhejiang, Pan'an, Zhejiang, China

* Corresponding author: E-mail: huangjh@zafu.edu.cn, Tel: 86-571-63732758, Fax: 86-571-63740898

Abstract

Lignocellulose, as the key structural component of plant biomass, is of recalcitrant structure and is difficult to degrade. Meanwhile, the improper handling of plant residues is accelerating the process of global warming. Interestingly, we noticed that the xylophagous beetle, *Trypoxylus dichotomus* has a significant ability to decompose lignocellulosic biomass. However, no study has been conducted to elucidate the digestion mechanism from a genome-wide aspect for this beetle. Based on sequencing and assembling, the draft genome size of *T. dichotomus* is 636.27 Mb, with 95.37% scaffolds anchored onto 10 chromosomes. The phylogenetic results indicated that *T. dichotomus* and its closely related scarabaeid species *Onthophagus taurus* split from each other in the early Cretaceous. Furthermore, two digestive gene families (Trypsin and Enoyl-(Acyl carrier protein) reductase) have experienced significant expansion, accounting for the high degradation efficiency of lignocellulose. Additionally, the collinearity analysis revealed that chromosome breakages and

rearrangements occur in the evolution of *T. dichotomus* due to chromosomes 6 and 8 of *T. dichotomus* being intersected with chromosomes 2 and 10 of *Tribolium castaneum* respectively. As suggested by the larval intestinal transcriptome comparative analyses, the digestive ability of midgut is much stronger than that of hindgut, even though susceptible to different food habits. This study reported the well-assembled and annotated genome of this rhinoceros beetle, providing genomic and transcriptomic bases for further understanding the functional mechanism and evolutionary history of lignocellulolytic digestion of the beetle.

Keywords: Chromosome rearrangement, gene family, lignocellulose digestion, rhinoceros beetle, intestinal transcriptome

1. Introduction

Plant biomass, mainly consisting of lignocellulose, is the most abundant, sustainable and widespread source of renewable carbon as well as the major sink for photosynthetically fixed carbon on earth [1-4]. In the Anthropocene, it constitutes a large component of municipal, agricultural, forestry and industrial wastes [5]. But the improper handling of plant biomass is one of the most important factors accelerating global warming [6, 7]. As the key structural component, lignocellulosic biomass consists in almost all kinds of living and dead plants serving as an important route of carbon fixation. They are principally composed of the polymers cellulose, hemicellulose, pectin and lignin [8], which form a complex cross-linked and

recalcitrant structure complex, that protecting the carbohydrates from decomposition by microorganism or enzymes [4, 9]. Thus, the enormous quantity of plant wastes from production is a noteworthy environmental issue that should be solved by recycling the waste to produce valuable organic matters [10]. Nowadays, the pretreatment of lignocellulose degradation, including chemical and biological processes, have catalyzed a great interest [6, 11, 12], especially for biotransformation, an environmental-friendly and sustainable strategy for biofuels and biomaterial production.

Lignocellulose decomposition is not a common trait among animals because of its complex structural and chemical mechanisms for resisting assault [13, 14], but wood-feeding insects, such as termites, wood-feeding cockroaches, beetles and wood wasps capitalize a high-efficient digestion ability of lignocellulosic biomass [11, 12, 14-16]. These insects were involved in the degradation of lignocellulose and other types of biomass by consuming plant cell walls, thereby contributing to lignocellulose bioconversion and energy utilization [17]. Among them, xylophagous termites were the most famous and efficient lignocellulose digesters, receiving preeminent research achievements, especially in functional genomics and symbiotic intestinal microorganism [18-20]. Many studies concerned on the chemical degradation and microbiological deterioration of lignocellulose [5], while limited attention was paid to the biodegradability and genetic traits of the other xylophagous insects, such as the well-known ornamental scarabaeid beetle, *Trypoxylus dichotomus*, who share the

similar diet with xylophagous termites.

The rhinoceros beetle, *T. dichotomus* (Linnaeus, 1771) (Coleoptera: Scarabaeidae), is an ecologically important xylophagous and saprophagous insect, widely distributed in China and its neighboring countries [21]. In the larval stage, it could decompose the recalcitrant wood material and humus efficiently [22-24], which has been applied to biotransform the waste substrate from mushroom production [25]. It usually secretes digestive enzymes comprising cellulase, glycanase and glycosidase for degrading lignocellulose-rich plant polymers [22], greatly promoting the formation of soil organic matter, which is known as the major pool of organic carbon sensitive to changes in ecological environment [26, 27]. To date, several studies have been concentrated on the digestive enzymes and mutualistic associations with microbial symbionts in larval guts [22, 24, 28-30]. However, without the genome data of the beetle, the mechanism of its strong digestive ability of lignocellulose is still not fully revealed.

It was generally suggested that different diets have significant effect on digestive enzymatic activity for beetles [31]. Regional differentiation of digestive tract and adaptations to divergent feeding habits mediate the efficient digestion of the diet and protect insect against hazardous substance from food [32]. Although larva of the rhinoceros beetle could degrade the decaying wood and mushroom-residue efficiently, no study has been done for its digestive ability on different food habits. To understand the gut segment-specific function and molecular pattern of the larval

digestive tract of *T. dichotomus*, it is essential to identify the digestion-related genes and characterize gene expression on different food habits.

In this research, we studied the genomic characteristics of rhinoceros beetle and performed the comparative genomic analysis with related insects from the released database. Furthermore, we conducted intestinal transcriptome comparative analysis of 3rd larvae feeding on sawdust and mushroom-residue, separately. We firstly presented the chromosome-level genome assembly of *T. dichotomus* and clarified the evolutionary history of gene families, with a highlight on the rapid expansion of those digestion related gene families and chromosome evolution. And then, we illustrated the effects of food habits on larval intestinal segments and digestive ability for the rhinoceros beetle.

2. Materials and methods

2.1 Sampling and sequencing

The male and female adult samples and living larvae of *T. dichotomus* were obtained from the artificial breeding base in Pan'an County (28.94°N, 120.55°E), Zhejiang Province, China, in May 2020 and transported to the laboratory. The adult samples were washed three times with distilled water and then transferred to clean bench for dissection. Muscle of a female thorax was prepared for Illumina and Nanopore sequencing, and then a male thorax was dissected for Hi-C and RNA sequencing

(Table S1). Prior to the extraction of genomic DNA and RNA, the samples were transferred to liquid nitrogen for preservation. All the larvae were divided into two groups and reared with sawdust and mushroom-residue for two months, separately. During the feeding and digestion process, wood fiber was well degraded into fragments after digestion by larvae (Fig. 1). Then, six 3rd instar individuals were selected for each group, and rinsed twice quickly using 75% alcohol. Since the foregut is small and short with weak digestion and digestive activities mainly occur in the midgut and hindgut [22], only midgut and hindgut were separated and rinsed twice quickly with diethyl pyrocarbonate (DEPC) and phosphate-buffered saline (PBS, 1X) successively in clean bench during the dissection process. After drying the surface liquid, twenty-four midgut and hindgut samples were preserved in liquid nitrogen, separately (Table S2). All the samples were divided into four groups: 1) midgut from sawdust (SM, midgut of larva feeding sawdust), 2) hindgut from sawdust (SH, hindgut of larva feeding sawdust), 3) midgut from mushroom-residue (MM, midgut of larva feeding mushroom-residue), 4) hindgut from mushroom-residue (MH, hindgut of larva feeding mushroom-residue). Each group consisted of six replicates.

Genomic DNA was extracted using QIAGEN® Genomic kit for gene library construction. Prior to genome sequencing, the *k*-mer distribution analysis was performed using genome survey sequences (GSS, Illumina DNA data) to estimate the genome size and heterozygosity. Briefly, quality-filtered reads were subjected to 17-mer frequency distribution analysis using the Jellyfish program [33]. By analyzing

the 17-mer depth distribution from the 350-bp library cleaned sequencing reads, genome size and heterozygosity were estimated with FindGSE (skew normal distribution model) [34] and GenomeScope (negative binomial model) [35], separately. After genome estimation, a certain concentration and volume of DNA library was transferred to a flow cell of PromethION (ONT, Oxford Nanopore Technologies) for whole genome sequencing. Total RNA was extracted using QIAGEN® RNeasy Plus Universal Mini Kit, and then ribosomal RNA (rRNA) was removed with QIAseq FastSelect RNA Remove Kits. Sequencing libraries were generated using TruSeq RNA Library Preparation Kit (Illumina, USA) following manufacturer's recommendations. The library preparations were sequenced on an Illumina Novaseq platform and 150 bp pair-end reads were generated.

2.2 Genome assembly

The quality of reads was controlled using ONT Guppy (v3.2.2) referring to the value of mean_qscore_template ≥ 7. Pass reads were assembled with NextDenovo (v2.0) (reads_cutoff:1k, seed_cutoff:23k). Raw data were aligned with the assembled genome using Minimap2 [36] (-x map-ont) for sequence alignment information. Based on this information, the genome was corrected using Racon (v1.3.1) in three iterations. The Next-Generation Sequencing (NGS) data were filtered by fastp (v0.20.0, -n 0) [37]. The corrected genomic data were polished with the filtered data using Nextpolish (v1.0.5) in four iterations.

Possible contaminated sequences were detected using BLAST+ v2.9.1 [38]

against the nt and UniVec databases, and then removed. Scaffolds greater than 10 kb were retained and uploaded to NCBI for double check in the final assembly. Comparing to the insecta_odb10 database in OrthoDB, a Benchmarking Universal Single-Copy Orthologs (BUSCO) analysis was performed to assess completeness of genome assembly using BUSCO v4.0.5 [39]. As a reference genome, the assembled genome was aligned by the NGS data using BWA v0.7.12 [40], and then the alignment files were transferred to the programs Samtools v1.4 [41] and BCFtools v1.8.0 [42] to assess the sequence consistency. In order to evaluate the presence of exogenous contamination in the assembled genome, GC depth was analyzed using Minimap2 and Samtools.

To anchor hybrid scaffolds onto the chromosome, genomic DNA was extracted for the Hi-C library. Crosslinking was stopped by adding glycine and additional vacuum infiltration. Fixed tissue was then grounded to powder before re-suspending in nuclei isolation buffer to obtain a suspension of nuclei. The purified nuclei were digested with 100 units of DpnII and marked by incubating with biotin-14-dCTP. Biotin-14-dCTP from non-ligated DNA ends was removed owing to the exonuclease activity of T4 DNA polymerase. The ligated DNA was sheared into 300−600 bp fragments, and then was blunt-end repaired and A-tailed, followed by purification through biotin-streptavidin-mediated pull down. Finally, the Hi-C libraries were quantified and sequenced using the Illumina Novaseq platform. Quality controlling of Hi-C raw data and extraction of Hi-C contacts were performed using Juicer v1.6.2 [43].

Hi-C contigs were anchored to pseu-chromosomes using two rounds of 3D-DNA v180922 [44] workflow. The initial assignment was manually corrected using Juicebox v1.11.08 [43], and then imported into 3D-DNA again to produce the final chromosome-anchored genome assembly, with the contigs separated by 100 Ns on the same chromosome.

2.3 Genome annotation

De novo repeat library was constructed using RepeatModeler v2.0.1 with long terminal repeat (LTR) structural search [45], and then combined with the databases of Dfam_3.1 and RepBase-20181026 to be custom library. Repetitive elements (DNA/short interspersed nuclear element (SINE)/long interspersed nuclear elements (LINE)/LTR) were searched applying the program RepeatMasker v4.1.0 [46] based on the database of repeated sequences.

Protein-coding gene (PCG) structure was predicted in the pipeline of MAKER v3.01.03 [47]. The following three strategies were integrated for the prediction. 1) *Ab initio* gene structure prediction was made by applying the BRAKER v2.1.5 pipeline [48] together with self-training of Augustus v3.3.4 [49] and GeneMark-ES/ET/EP 4.59_lic [50]. To improve prediction accuracy, transcripts of thoracic muscle was incorporated with protein homology-based evidence, in which transcriptome evidence in BAM alignments was produced using HISAT2 v2.2.0 [51]. The arthropod protein source was mined from the OrthoDB10 v1 database [52]. 2) With the BAM alignments

inputted, transcripts of thoracic muscle were assembled using the genome-guided assembler StringTie v2.1.4 [53]. 3) Protein sequences for *Drosophila melanogaster* (Diptera), *Apis mellifera* (Hymenoptera), *Bombyx mori* (Lepidoptera), beetles (*Tribolium castaneum*, *Onthophagus taurus*, *Anoplophora glabripennis*) were downloaded from NCBI and passed to MAKER as evidence of protein homology. The prepared files obtained from the above pipeline were imported into MAKER for integrated annotation.

Gene function was annotated with the following two strategies. 1) Gene functions were annotated by searching the protein sequence database UniProtKB using Diamond v0.9.24 (--more-sensitive -e 1e-5) [54]. 2) Protein conserved sequences and domains, Gene Ontology (GO), and pathways [Kyoto Encyclopedia of Genes and Genomes (KEGG), Reactome] were predicted by searching Pfam [55], SMART [56], Gene3D [57], Superfamily [58], CDD [59] using InterProScan 5.41-78.0 [60]. Then their functions were predicted by searching in eggNOG v5.0 database [61] employing eggNOG-mapper v2.0.1 [62].

Noncoding RNAs (ncRNAs) were annotated with two strategies. 1) ribosomal RNAs (rRNAs), small nuclear RNAs (snRNAs) and micro RNAs (miRNAs) were searched against Rfam database using the program infernal v1.1.3 [63]. 2) tRNAs were predicted using tRNAscan-SE v2.0.6 [64], with low-credibility tRNAs filtered out using the script ‘EukHighConfidenceFilter’. Based on the results of genome annotation, chromosome length, GC-content, density of PCGs and repetitive

elements on each pseudo-chromosome were plotted and visualized by Circos (v0.67–7) [65].

2.4 Comparative genomic and phylogenetic analysis

Gene family homology were inferred from protein sequences of 13 representing insect species downloaded from NCBI, including nine beetles of *Tri. castaneum*, *Agrilus planipennis*, *Lamprigera yunnana*, *Nicrophorus vespilloides*, *Onthophagus taurus*, *Aethina tumida*, *Sitophilus oryzae* and *Anoplophora glabripennis*, and five other insect species of *Drosophila melanogaster* (Diptera), *Apis mellifera* (Hymenoptera), *Bombyx mori* (Lepidoptera), *Coptotermes formosanus* (Blattodea) and *Rhopalosiphum maidis* (Hemiptera). Gene families were identified by clustering protein sequences using OrthoFinder v2.3.8 [66] with Diamond [54] as sequence aligner.

Phylogenetic trees were constructed with protein sequences of 1,260 single-copy orthologs, which were aligned with MAFFT v7.394 using the model of 'L-INS-I' [67]. The unreliable homologous regions were removed by BMGE v1.12 [68]. All the well-aligned sequences were concatenated with FASconCAT-G v1.04 [69]. Maximum likelihood (ML) trees were constructed using IQ-TREE v2.0.7 [70] with the set of '--symtest-remove-bad --symtest-pval 0.10' for removing those genes not conforming to SRH (stationary, reversible, and homogeneous). The substitution model was constrained to LG with heuristic partitioned search strategy '-m MFP

--mset LG --msub nuclear --rclusterf 10’, node support values were evaluated with ultrafast bootstrap and SH-aLRT algorithms (-B 1000 --alrt 1000). Divergence time on phylogenies was estimated using r8s v1.81 [71]. Fossil calibration data were obtained from PBDB database [72], and another two published literatures [73, 74], including root (Pterygota, <443.4 Mya), Holometabola (315.2–382.7 Mya), Lepidoptera+Diptera (Trichoptera, 311.4–323.2 Mya), Coleoptera (307–323.2 Mya), Scarabaeiformia (196.5–201.3 Mya), Elateriformia (242–252 Mya) and Cucujiformia (196.5–201.3 Mya).

Expansions and contractions of gene families at each node of the evolutionary tree were estimated using CAFÉ v4.2.1 [75] under the stochastic gene birth-death model and default significance level (p=0.01). For significantly expanded gene families, GO and KEGG functional enrichment analyses were performed using R package clusterProfiler v3.14.3 [76] with the default parameters. Natural selection was analyzed among the 45 rapidly expanded gene families for further understanding the evolution of expanded gene families. Coding sequence (CDS) analysis of each gene family was performed using the PAML package of codeml [77] under the site models. Models applied in this step concludes M0 (one rate), M1a (neutral)–M2a (selection) and M7 (beta)–M8 (beta&ω) (NSsites = 0 1 2 7 8). Likelihood ratio test was compared between the results from M1a–M2a and M7–M8 models (p=0.05), respectively. Bayes Empirical Bayes (BEB) inference [78] was performed for testing the positive loci in each gene family.

Chromosomal collinearity was performed to investigate variation/conservation of chromosomes between *T. dichotomus* and related beetle *Tri. castaneum* (Coleoptera: Tenebrionidae), whose genome was assembled in chromosome-level with 10 chromosomes (9 autosomal chromosomes and the X) identified [79]. Gene and protein sequences were aligned using MMseq2 v11-e1a1c [80] under the default parameters (-s 7.5 --alignment-mode 3 --num-iterations 4 -e 1e-5 --max-accept 5). Collinearity analysis was performed using MCScanX [81] with the collinear block containing at least five homologous genes (-s 5 -e 1e-10). Chromosome collinearity diagram was finally visualized using TBtools v1.0692 [82].

2.5 Intestinal transcriptome analysis

Raw reads were further filtered by fastp to remove adapters and low quality bases. The rRNA reads were found and removed by mapping short reads to rRNA database with Bowtie2 (version 2.2.8) [83]. The remaining clean reads were mapped to the reference genome using HISAT2 with “-rna-strandness RF” and other parameters set as a default.

For each transcription region, a FPKM (fragment per kilobase of transcript per million mapped reads) value was calculated to quantify its expression abundance and variations, using RSEM software [84]. Based on FPKM, principal component analysis (PCA) and Pearson correlation coefficient (PCC) were performed with R package gmodels [85]. RNAs differential expression analysis was performed by DESeq2 [86]

software between two different groups (and by edgeR [87] between two samples). The genes with the parameter of false discovery rate (FDR) below 0.05 and absolute fold change ≥ 2 (|FC|≥2) were considered differentially expressed genes. All differentially expressed digestion-related genes were filtered by KEGG pathways and GO terms. Heatmaps of digestion-related gene differential expression were visualized using TBtools.

3. Results and Discussion

3.1 Genome estimation

Before ONT sequencing, 25 Gb (more than 40×) NGS DNA data with GC-content of 35.85% was obtained for sample quality and genome assessment (Table S3). By analyzing the 17-mer depth distribution from the 350-bp library cleaned sequencing reads, genome size and repeat ratio was then estimated to be 630.93 Mb and 32.29% in FindGSE, 567.40 Mb and 22.99% in GenomeScope (Fig. S1; Table S4), respectively. Further combining the simulation results, the final genome size of *T. dichotomus* was estimated to be about 599.17 Mb, with 2.09% heterozygous ratio, suggesting the good quality of the test sample. The N50 and the mean length of the long reads were 24.54 Kb and 16.88 Kb, respectively. The longest read was 170.57 Kb. Furthermore, 12 Gb NGS RNA data was obtained for genome evaluation and

annotation (Table S3).

Table 1. Genome assembly and quality evaluation

Assembly	Total length (Mb)	Number of scaffolds	N50 length (Mb)	Longest scaffold (Mb)	GC (%)	BUSCO (n = 1,367) (%)			
						C	D	F	M
NextNedovo	636.56	304	14.44	27.42	35.12	99.7	1.0	0.0	0.3
3D-DNA	636.61	496	71.04	94.63	35.12	99.3	0.7	0.2	0.5
Final	636.27	417	71.04	94.63	35.11	99.2	0.7	0.3	0.5

Note: C, complete BUSCOs; D, complete and duplicated BUSCOs; F, fragmented BUSCOs; M, missing BUSCOs.

3.2 Genome assembly and assessment

ONT sequencing generated 73 Gb (approximately 120×) pass reads (Table S3), which were then corrected by the NextCorrect module (NextDenovo) for further producing 45 Gb consensus sequences. The preliminary assembly was generated using the NextGraph module (NextDenovo), with the genome size of 634.66 Mb and N50 length of 14.42 Mb. After being corrected and polished by Racon and Nextpolish, the polish genome size was 636.56 Mb, with the scaffold N50 length of 14.44 Mb (Table 1), suggesting the good continuity of our assembled genome (Fig. 2a).

NovaSeq sequencing generated 83 Gb Hi-C data (Table S3), which was filtered to produce 79 Gb clean data. Based on the clean data in the 3D-DNA analysis, the chromosome-anchored genome size was estimated to be 636.61 Mb, with 496 scaffolds and N50 length of 71.04 Mb (Table 1). After polishing, removing redundancy and contaminants, and Hi-C scaffolding, the final genome size was determined to be 636.27 Mb, composed of 417 scaffolds, with scaffold/contig N50 length of 71.04/12.99 Mb, GC-content of 35.11%, and gaps of 0.004% (Table S5), which was

pretty close to the earlier genome estimation by FindGSE. Furthermore, 606.8 Mb scaffolds covering 95.37% of the draft reference genome were precisely anchored onto 10 pseudo-chromosomes (Fig. 2b), indicating the high-quality of the chromosome-level genome assembly.

Taken all the known genomes of Scarabaeidae into account, the genomic characteristics vary significantly among the eight retrievable genomes of scarabaeid beetles, with a genome size of 267–1144 Mb [88-90]. A draft genome assembly of *T. dichotomus* is recently released in GenBank (Bioproject: PRJDB10500; genome size of 739.41 Mb; scaffold N50 length of 7.93 Mb; scaffold number of 2,347) without further analysis. *Trypoxylus dichotomus* has a relatively larger genome than most other scarabaeids, but has a similar GC-content close to 35% (except 25% for *Protaetia brevitarsis*, Bioproject: PRJNA477715). The clearest example is found in its closest relative species from the same subfamily, *Onthophagus taurus*, which has a much smaller genome size of 267.08 Mb (Bioproject: PRJNA419349).

BUSCO assessment (n = 1,367) identified 1,356 (99.2%) conserved orthologous as complete genes. The “complete and single-copy BUSCOs” genes accounted for 98.5% of the total genes, and the “complete and duplicated BUSCOs” genes represented 0.7% (Table 1). The mapping rates of NGS reads (GSS, RNA-seq) and ONT reads onto the draft reference genome were high as 99.89%, 95.39% and 99.60%, respectively. All of the above assessment results indicated that the genome assembly of *T. dichotomus* reaches an extremely high quality in both continuity and

integrity in this study.

3.3 Genome annotation

A total of 1,369,555 repeat sequences (365,506,399 bp) were identified, accounting for 57.45% of the whole genome, in which the top five types with the highest proportions included DNA elements (28.97%), unclassified (16.67%), LINEs (9.69%), LTRs (1.24%), SINEs (0.52%) and simple repeats (0.52%) (Table S6). The density of each type (except unclassified) was shown on each chromosome, indicating that DNA elements and LINEs having the maximum densities (Fig. 3).

MAKER pipeline predicted 12,193 PCGs, in which average lengths of genes, CDS, peptide chain and transcript were 15,150 bp, 1,743 bp, 580 and 2,355 bp, respectively. Each gene has a size of 339 bp and 8 exons in average. It's worth noting that the average size of introns reaches up to 1,857 bp (much longer than exons) in rhinoceros beetles (Table S5), which was common in organisms with large genomes [91]. Furthermore, BUSCO assessment (n = 1,367) identified 95.8% conserved orthologous as complete genes in the predicted PCGs, indicating that our prediction was relatively complete.

After PCG functional annotation, 11,551 (94.73%) genes were detected matching the UniprotKB records by Diamond, while 10,640 (87.26%) protein domains of PCGs were identified by InterProScan. In further, 10,535 GO, 8,224 KEGG ko, 2,886 Enzyme Codes, 9,431 KEGG pathways, 10,590 Reactome pathways and

12,025 COG categories were identified by InterProScan and eggNOG-mapper.

Based on the annotation by Rfam database and tRNAscan-SE, 668 ncRNAs were identified in the genome, including 43 rRNAs, 57 miRNAs, 129 snRNAs, two long noncoding RNAs (lncRNAs), two ribozymes, 361 tRNAs, and 74 other ncRNAs. The snRNAs were classified as 106 spliceosomal RNAs (U1, U2, U4, U5, U6 and U11), five minor spliceosomal RNAs (U4atac, U6atac and U12), 14 C/D box snoRNAs, three H/ACA box snoRNA and one another snRNA (SCARNA8). There were 21 isotypes of tRNAs annotated in this species, but with the Supres isotype missing (Table S7).

3.4 Comparative genome and phylogeny

Gene family identification

A total of 181,904 (92.90%) genes were clustered into 14,467 orthogroups (gene families), including 1,260 single-copy orthogroups and 3,120 multi-copy orthogroups. Of all the PCGs in the genome of this beetle, 11,614 (95.25%) genes were clustered into 8,727 orthogroups, in which 107 orthogroups/488 genes were specific to *T. dichotomus* (Table 2, Fig. 4a).

Table 2. Statistics of gene families among fourteen insects

Category	Number
Number of species	14
Number of genes	195,765
Number of genes in orthogroups	181,904
Number of unassigned genes	13,861
Percentage of genes in orthogroups	92.9

Number of orthogroups	14,467
Number of species-specific orthogroups	3,396
Number of genes in species-specific orthogroups	15,299
Percentage of genes in species-specific orthogroups	7.8
Mean orthogroup size	12.6
Number of orthogroups with all species present	4,380
Number of single-copy orthogroups	1,260

Phylogeny and gene family evolution

Removing 152 single-copy orthologs using symtest, the remaining 1,108 single-copy orthologs (450,544 amino acids) were concatenated for the phylogenetic tree reconstruction (Fig. 4a). The phylogenetic relationships of these 14 insect species were commendably recovered [74, 92], with all the nodes being strongly supported (UFB/SH-aLRT = 100/100), showing the good resolution in the phylogram. Coinciding with the preceding beetle phylogenomic study [92], the present results indicated that Coleoptera originated in the Early Carboniferous (320 Mya), while the splits of *T. dichotomus* and its closely related scarabaeid species *O. taurus* occurred in the early Cretaceous (120 Mya) (Fig. S2).

Gene family evolution analysis revealed that 610 and 1,405 gene families experienced expansions and contractions, respectively, in which 67 gene families (45 expansions and 22 contractions) were recognized as rapidly evolving orthogroups (Fig. 4a). The significantly expanded families were primarily associated with digestion (trypsin, enoyl-(acyl carrier protein) reductase), detoxification (cytochrome P450, ecdysteroid kinase, carboxylesterase, aldo/keto reductase), chemoreception (odorant receptor, gustatory receptor), glycometabolism (facilitated trehalose transporter,

neutral alpha-glucosidase), immunity (15-hydroxyprostaglandin dehydrogenase [NAD(+)], galectin, serine protease Hayan, prostaglandin reductase 1, inducible metalloproteinase inhibitor protein), development (haemolymph juvenile hormone binding protein (JHBP), juvenile hormone acid O-methyltransferase, serine protease snake) and toxoprotein (venom acid phosphatase) (Fig. 5a; Table S8). The rapidly expanded gene families were confirmed in the GO and KEGG enrichments, which mainly reflect metabolic detoxification, digestion and immunity in the GO enrichment (Fig. 5b), and metabolic detoxification, digestion, juvenile hormone and secondary metabolite synthesis in the KEGG pathway (Fig. 5c). Four gene families were positively selected, including serine protease Hayan (OG0000411), phosphatidylinositol phosphatase (OG0001456), Hsp70 protein (OG0009015) and nucleoporin autopeptidase (OG0009016), which were related to immunity, cell proliferation/differentiation, heat shock protein and nucleo-cytoplasmic transport, respectively (Table S9). Accordingly, digestion, detoxification and immunity were significantly reflected in the rapidly expanded gene families and functional enrichment, meanwhile gene family concerning immunity was affected by positive selection as well.

Most beetles were considered not capitalize the significant ability of endogenous lignocellulose digestion [93], but it was clearly not the case for *T. dichotomus*. The present results revealed that the functional capacity of digestion was obviously reinforced by expansions of gene families [94] in the evolutionary process of *T.*

dichotomus, which would promote lignocellulose digestion greatly. Additionally, detoxification and immunity function were also reinforced by gene family expansion and positive selection, suggesting the adaptive evolution responding to environmental exposures [95, 96]. This corresponds to the diversification of expression patterns of *T. dichotomus* adapted to different humus resources [97].

Collinearity

In the collinearity analysis, 262 collinear blocks were constructed based on 4,477 collinear genes (18.69% of all genes), with 6–23 genes in each block (Table S10). Chromosomes 1–7 and 9–10 of *T. dichotomus* (TdChr1–7 and 9–10) mapped to chromosomes 3, 7, 5, 4, 9, 2, 8, 6 and X of *Tri. castaneum* (TcChr3, 7, 5, 4, 9, 2, 8, 6 and X) with strong syntenic relationships respectively, while only chromosome 8 of *T. dichotomus* (TdChr8) showed the relatively low synteny with chromosome 10 of *Tri. castaneum* (TcChr10) (Fig. 4b). This indicates the high genome synteny between *T. dichotomus* and *Tri. castaneum*, which clearly reveals the overall conservation of chromosomes in *T. dichotomus* [98]. Sharing all the collinear genes, TdChr10 mapped perfectly to TcChrX (Fig. 4b), one of the sex chromosomes of *Tri. castaneum* considering its XY karyotype [99]. Thus, TdChr10 was suggested to be X chromosome in *T. dichotomus* here.

Collinear genes were intersected within homologous chromosomes extensively (Fig. 4b), indicating the common reshuffling of gene order within chromosomes, i.e.

intrachromosomal rearrangements (inversions) [100]. In contrast, collinear genes were occasionally intersected among nonhomologous chromosomes, with only five pairs of chromosome intersections (TdChr1-TcChr8, TdChr3-TcChr3, TdChr6-TcChr3, TdChr6-TcChr10 and TdChr8-TcChr2), meaning interchromosomal rearrangements (translocations) [101]. Most notably, TdChr6 and 8 were significantly intersected with TcChr2 and 10 respectively, indicating a wide variety of chromosome breakages and rearrangements [100] in the evolutionary history of rhinoceros beetle.

Although the clades of *T. dichotomus* (Scarabaeoidea) and *Tri. castaneum* (Tenebrionoidea) have been diverged in the late Permian (Fig. 4a), their chromosomes (autosomes and X chromosome) are conserved in account of the relatively limited translocations, which might indicate the relative conservation of chromosomes in the evolutionary history of beetles to some extent. In contrast to the autosomes, X chromosome was considered to be more conserved and more recalcitrant to rearrangement with the autosomes for insects [98, 101-103], which was obviously true for beetles according to the current research. Accordingly, we assume that intrachromosomal rearrangements possibly are the main evolutionary force for beetles, among which autosome rearrangements might be the most important factor. Nevertheless, in spite of the occasional occurrences, interchromosomal rearrangements of autosomes might play a vital role in the evolutionary process of beetles as well.

3.5 Gene expression and sample correlation

Based on the gene expression (FPKM) of all annotated genes for each sample, PCA and PCC (Table S11-12) were calculated and plotted with diagrams (Fig. 6), separately. PCA analysis (Fig. 6a) indicates that samples from the same group are well aggregated together, except two outliers (SM2 and SM6) in the midgut of sawdust feeding beetles. Similarly, PCC analysis (Fig. 6b) shows good repeatability within the most of intra-groups, but low in the midgut of sawdust feeding beetles for the reason of abnormal values of SM2 and SM6. Although correlations exist between groups, each group is separated from the others (Fig. 6a), which suggests that intestinal gene expressions are significantly affected by food habits for the rhinoceros beetle. Similarly, it was reported that different host diets could significantly affect digestive physiology of the beetle, *Trogoderma granarium* [31], which is the same case for *T. dichotomus*.

The gene expression patterns of hindguts from sawdust and mushroom-residue overlap, while their expression patterns of midguts separate (Fig. 6a). This result indicates that the hindguts from different food habits share similar pattern of gene expression, while gene expression patterns of midguts differ by food habits (Fig. 6a). Larval midgut appears more susceptible to food habits than hindgut for the rhinoceros beetle, which is line with the digestive enzyme activities of this beetle, which suggested the midgut plays a major role in degradation, digestion and absorption [30].

3.6 Differentially expressed digestion-related genes

Digestion-related genes were filtered excluding those with mean gene count less than 5 within group for all the groups (Table S13), in which differentially expressed genes were further compared within four different treatment groups (Table S14, Fig. 7). Total 222 differentially expressed digestion-related genes are filtered in the midgut and hindgut from sawdust group, in which 128 and 94 genes are highly expressed in the midgut and hindgut, respectively (Fig. 7a). Likewise, 231 differentially expressed digestion-related genes are detected in the midgut and hindgut from mushroom-residue group, among which 137 and 94 genes are highly expressed in the midgut and hindgut, respectively (Fig. 7b). These results indicate that the highly expressed digestion-related genes significantly vary between midgut and hindgut, with much more genes in the midgut despite of different food habits. The larval digestive ability of midgut might be stronger than that of hindgut for this beetle, which is consistent with the previous studies that polysaccharide degradation occurs mainly in the midgut for the rhinoceros beetle [22, 30].

In addition, the variation of the highly expressed digestion-related genes in guts are found between sawdust group and mushroom-residue group. In the 92 differentially expressed digestion-related genes of midguts from the two different food habits, there are 65 and 27 genes highly expressed in the mushroom-residue group and the sawdust group, respectively (Fig. 7c). Similarly, 83 differentially expressed digestion-related genes are detected in hindguts, among which 52 and 31 genes are

highly expressed in the mushroom-residue group and the sawdust group, respectively (Fig. 7d). Taken together, more digestion-related genes are highly expressed in the mushroom-residue group than the sawdust group regardless of midgut or hindgut. The result suggests a much stronger digestive ability of the rhinoceros beetle that feeding on mushroom-residue, which indicates that the species is suitable for digesting the lignocellulose after previous degradation by fungi [104].

The rhinoceros beetle might serve as an efficient decomposer in lignocellulose-enriched agro-forestry residues including mushroom-residue and decaying wood, which would provide an environmental-friendly method for sustainable development. In the forest, the larvae of *T. dichotomus* usually inhabit the soil organic matter and feed on decayed wood [22, 23, 30]. This is similar to the living and feeding habitats of white-spotted flower chafer, *Protaetia brevitarsis* (Scarabaeidae), which was found to be efficient in digesting high lignocellulosic mushroom-residue as well [105]. Interestingly, both species were often observed coexisting in the outdoor mushroom-residue, showing that these two scarab beetles might share an overlapped ecological niche and promote more effective lignocellulosic degradation through close cooperation.

4. Conclusion

In this study, we assembled and provided the first chromosome-level genome of *T. dichotomus* in the family Scarabaeidae. Combing different assembling methods, we

identified its final genome size to be 636.27 Mb with the BUSCO completeness up to 99.2%, indicating the high quality of this genome assembly. Furthermore, there were 95.37% scaffolds of the draft reference genome anchored onto 10 chromosomes, in which chromosome 10 was identified as X chromosome (sex chromosome) of *T. dichotomus*. In addition, the result of collinearity analysis showed that chromosome 6 and 8 of *T. dichotomus* are intersected with chromosome 2 and 10 of *Tri. castaneum*, revealing the events of chromosome breakages and rearrangements evolutionarily occurred for *T. dichotomus*. Based on 1,108 single-copy orthologs, the phylogenetic relationships of the involved beetles were recovered, which confirmed that *T. dichotomus* have been diverged in the early Cretaceous (120 Mya) from its closely related species *O. taurus*. Interestingly, gene families associated with digestion, immunity and detoxification have been significantly expanded in the evolutionary history of *T. dichotomus*, indicating the enhancement of adaptive capacity to environmental for the rhinoceros beetle. This would well account for its high degradation efficiency of lignocellulosic biomass and extensive adaptability to humus environment in the larval stage. Comparative analysis of intestinal transcriptome of larvae that feeding on sawdust and mushroom-residue suggested that a stronger digestive ability in midgut than hindgut, even though susceptible to different food habits. In brief, the chromosome-level genome assembly and larval intestinal transcriptome analyses will facilitate future genetic studies on the lignocellulose degradation for effective utilization of *T. dichotomus* in the eco-friendly biotreatment of

plant biomass. In further, the well-assembled and annotated genomic data will provide the valuable resource for further understanding of beetle evolutionary history and new insights into the mechanism of functional genes in future.

Acknowledgements

We express our special thanks to Dr. Feng Zhang (Nanjing Agricultural University, China) for the help in data analyses, and Dr. Pu Tang (Zhejiang University, China) for the helpful advice during the initial stage of this research. We also thank Dr. Shouke Zhang (Zhejiang A&F University, China) and Dr. Huaijun Xue (Nankai University, China) for their kind suggestions in larval experimental design, and Jinliang Bao (Shanzhizhou Ecological Agriculture Company Limited, Zhejiang, China) who supplied the adults and larvae of the rhinoceros beetle for this study. We are particularly thankful to the anonymous reviewers for their valuable comments and suggestions on the manuscript. This work was supported by Cooperation Project of Zhejiang Province and Chinese Academy of Forestry (Grant No. 2020SY08).

Data accessibility

The data sets supporting the results of this article are available in the GenBank repository. The whole genome sequencing and assembly project has been deposited at GenBank (NCBI BioProject: PRJNA688811). The chromosome-level genome assembly of *Trypoxylus dichotomus* has been stored in the NCBI database under Accession no. JAENHH000000000. All the sequencing raw data, including genome survey, Nanopore, Hi-C and RNA sequencing, have been submitted to the BioProject PRJNA688811.

Author contributions

Qingyun Wang: Methodology, Software, Validation, Formal analysis, Investigation, Data process, Writing - original draft, Visualization. **Junhao Huang:**

Conceptualization, Resources, Writing - review & editing, Project administration, Funding acquisition. **Liwei Liu:** Conceptualization, Writing – review & editing. **Sujiong Zhang:** Resources, Writing - review & editing. **Hong Wu:** Supervision.

References

1. Alessi AM, Bird SM, Oates NC, Li Y, Dowle AA, Novotny EH, et al. Defining functional diversity for lignocellulose degradation in a microbial community using multi-omics studies. Biotechnology for Biofuels. 2018;11 1:1-16. doi:https://doi.org/10.1186/s13068-018-1164-2.
2. Bredon M, Dittmer J, Noël C, Moumen B and Bouchon D. Lignocellulose degradation at the holobiont level: teamwork in a keystone soil invertebrate. Microbiome. 2018;6 1:1-19. doi:https://doi.org/10.1186/s40168-018-0536-y.
3. Bredon M, Herran B, Lheraud B, Bertaux J, Grève P, Moumen B, et al. Lignocellulose degradation in isopods: new insights into the adaptation to terrestrial life. BMC Genomics. 2019;20 1:1-14. doi:https://doi.org/10.1186/s12864-019-5825-8.
4. Tan J, Li Y, Tan X, Wu H, Li H and Yang S. Advances in pretreatment of straw biomass for sugar production. Frontiers in Chemistry. 2021;9 doi:https://doi.org/10.3389/fchem.2021.696030.
5. Sethi A and Scharf ME. Biofuels: fungal, bacterial and insect degraders of lignocellulose. eLS. 2013; doi:https://doi.org/10.1002/9780470015902.a0020374.
6. Sun J, Peng H, Chen J, Wang X, Wei M, Li W, et al. An estimation of CO2 emission via agricultural crop residue open field burning in China from 1996 to 2013. Journal of Cleaner Production. 2016;112:2625-31. doi:https://doi.org/10.1016/j.jclepro.2015.09.112.
7. Ravindra K, Singh T and Mor S. Emissions of air pollutants from primary crop residue burning in India and their mitigation strategies for cleaner emissions. Journal of Cleaner Production. 2019;208:261-73. doi:https://doi.org/10.1016/j.jclepro.2018.10.031.
8. Cragg SM, Beckham GT, Bruce NC, Bugg TD, Distel DL, Dupree P, et al. Lignocellulose degradation mechanisms across the Tree of Life. Curr Opin Chem Biol. 2015;29:108-19. doi:https://doi.org/10.1016/j.cbpa.2015.10.018.
9. Sanderson K. Lignocellulose: a chewy problem. Nature. 2011;474 7352:S12-S4. doi:https://doi.org/10.1038/474S012a.
10. Chen J, Fan X, Jiang B, Mu L, Yao P, Yin H, et al. Pyrolysis of oil-plant wastes in a TGA and a fixed-bed reactor: thermochemical behaviors, kinetics, and products characterization. Bioresour Technol. 2015;192:592-602. doi:https://doi.org/10.1016/j.biortech.2015.05.108.
11. Scully ED, Geib SM, Hoover K, Tien M, Tringe SG, Barry KW, et al. Metagenomic profiling reveals lignocellulose degrading system in a microbial community associated with a wood-feeding beetle. PLoS One. 2013;8 9:e73827. doi:https://doi.org/10.1371/journal.pone.0073827.
12. Gales A, Chatellard L, Abadie M, Bonnafous A, Auer L, Carrère H, et al. Screening of phytophagous and xylophagous insects guts microbiota abilities to degrade lignocellulose in bioreactor. Front Microbiol. 2018;9:2222. doi:https://doi.org/10.3389/fmicb.2018.02222.

13. Himmel ME, Ding S-Y, Johnson DK, Adney WS, Nimlos MR, Brady JW, et al. Biomass recalcitrance: engineering plants and enzymes for biofuels production. Science. 2007;315 5813:804-7. doi:https://doi.org/10.1126/science.1137016.
14. Sun J and Zhou XJ. Utilization of lignocellulose-feeding insects for viable biofuels: an emerging and promising area of entomological science. In: Liu T and Kang L, editors. Recent Advances in Entomological Research. Berlin Heidelberg: Higher Education Press, Beijing and Springer-Verlag; 2011. p. 434-500.
15. Geib SM, Filley TR, Hatcher PG, Hoover K, Carlson JE, del Mar Jimenez-Gasco M, et al. Lignin degradation in wood-feeding insects. Proceedings of the National Academy of Sciences. 2008;105 35:12932-7. doi:https://doi.org/10.1073/pnas.0805257105.
16. Bayané A and Guiot SR. Animal digestive strategies versus anaerobic digestion bioprocesses for biogas production from lignocellulosic biomass. Reviews in Environmental Science and Bio/Technology. 2011;10 1:43-62. doi:https://doi.org/10.1007/s11157-010-9209-4.
17. Luo C, Li Y, Chen Y, Fu C, Long W, Xiao X, et al. Bamboo lignocellulose degradation by gut symbiotic microbiota of the bamboo snout beetle *Cyrtotrachelus buqueti*. Biotechnology for Biofuels. 2019;12 1:1-16. doi:https://doi.org/10.1186/s13068-019-1411-1.
18. Warnecke F, Luginbühl P, Ivanova N, Ghassemian M, Richardson TH, Stege JT, et al. Metagenomic and functional analysis of hindgut microbiota of a wood-feeding higher termite. Nature. 2007;450 7169:560-5. doi:https://doi.org/10.1038/nature06269.
19. Brune A. Symbiotic digestion of lignocellulose in termite guts. Nature Reviews Microbiology. 2014;12 3:168-80. doi:https://doi.org/10.1038/nrmicro3182.
20. Li H, Yelle DJ, Li C, Yang M, Ke J, Zhang R, et al. Lignocellulose pretreatment in a fungus-cultivating termite. Proceedings of the National Academy of Sciences. 2017;114 18:4709-14. doi:https://doi.org/10.1073/pnas.1618360114.
21. Yang H, You CJ, Tsui CK, Tembrock LR, Wu ZQ and Yang DP. Phylogeny and biogeography of the Japanese rhinoceros beetle, *Trypoxylus dichotomus* (Coleoptera: Scarabaeidae) based on SNP markers. Ecol Evol. 2021;11 1:153-73. doi:https://doi.org/10.1002/ece3.6982.
22. Wada N, Sunairi M, Anzai H, Iwata R, Yamane A and Nakajima M. Glycolytic activities in the larval digestive tract of *Trypoxylus dichotomus* (Coleoptera: Scarabaeidae). Insects. 2014;5 2:351-63. doi:https://doi.org/10.3390/insects5020351.
23. Kojima W. Attraction to carbon dioxide from feeding resources and conspecific neighbours in larvae of the rhinoceros beetle *Trypoxylus dichotomus*. PLoS One. 2015;10 11:e0141733. doi:https://doi.org/10.1371/journal.pone.0141733.
24. Eo J, Na Y-E and Kim M-H. Influence of rhinoceros beetle (*Trypoxylus dichotomus septentrionalis*) larvae and temperature on the soil bacterial community composition under laboratory conditions. Soil Biol Biochem. 2017;108:27-35. doi:https://doi.org/10.1016/j.soilbio.2016.12.005.
25. Bao JI, Wang P, Zhang SJ and Chen ZL. *Forest-fungus-insect circular ecological breeding method*. Patent CN109964723 (A), China, 2019.
26. Schmidt MW, Torn MS, Abiven S, Dittmar T, Guggenberger G, Janssens IA, et al. Persistence of soil organic matter as an ecosystem property. Nature. 2011;478 7367:49-56. doi:https://doi.org/10.1038/nature10386.
27. Cotrufo MF, Soong JL, Horton AJ, Campbell EE, Haddix ML, Wall DH, et al. Formation of soil

organic matter via biochemical and physical pathways of litter mass loss. Nature Geoscience. 2015;8 10:776-9. doi:https://doi.org/10.1038/NGEO2520.

28. Takeishi H, Anzai H, Urai M, Aizawa T, Wada N, Iwabuchi N, et al. Xylanolytic and alkaliphilic Dietzia sp. isolated from larvae of the Japanese horned beetle, *Trypoxylus dichotomus*. Actinomycetologica. 2006;20 2:49-54. doi:https://doi.org/10.3209/saj.20.49.

29. Aizawa T, Urai M, Iwabuchi N, Nakajima M and Sunairi M. *Bacillus trypoxylicola* sp. nov., xylanase-producing alkaliphilic bacteria isolated from the guts of Japanese horned beetle larvae (*Trypoxylus dichotomus septentrionalis*). Int J Syst Evol Microbiol. 2010;60 1:61-6. doi:https://doi.org/10.1099/ijs.0.005843-0.

30. Wada N, Iwabuchi N, Sunairi M, Nakajima M, Iwata R and Anzai H. Site‐specific profiles of biochemical properties in the larval digestive tract of Japanese rhinoceros beetle, *Trypoxylus dichotomus* (Coleoptera: Scarabaeidae). Entomological Science. 2020;23 1:33-43. doi:https://doi.org/10.1111/ens.12394.

31. Borzou E, Naseri B and Namin FR. Different diets affecting biology and digestive physiology of the Khapra beetle, *Trogoderma granarium* Everts (Coleoptera: Dermestidae). Journal of Stored Products Research. 2015;62:1-7. doi:http://dx.doi.org/10.1016/j.jspr.2015.03.003.

32. Holtof M, Lenaerts C, Cullen D and Broeck JV. Extracellular nutrient digestion and absorption in the insect gut. Cell Tissue Res. 2019;377:397–414. doi:https://doi.org/10.1007/s00441-019-03031-9.

33. Marçais G and Kingsford C. A fast, lock-free approach for efficient parallel counting of occurrences of k-mers. Bioinformatics. 2011;27 6:764-70. doi:https://doi.org/10.1093/bioinformatics/btr011.

34. Sun H, Ding J, Piednoël M and Schneeberger K. findGSE: estimating genome size variation within human and Arabidopsis using k-mer frequencies. Bioinformatics. 2018;34 4:550-7. doi:https://doi.org/10.1093/bioinformatics/btx637.

35. Vurture GW, Sedlazeck FJ, Nattestad M, Underwood CJ, Fang H, Gurtowski J, et al. GenomeScope: fast reference-free genome profiling from short reads. Bioinformatics. 2017;33 14:2202-4. doi:https://doi.org/10.1093/bioinformatics/btx153.

36. Li H. Minimap2: pairwise alignment for nucleotide sequences. Bioinformatics. 2018;34 18:3094-100. doi:https://doi.org/10.1093/bioinformatics/bty191.

37. Chen S, Zhou Y, Chen Y and Gu J. fastp: an ultra-fast all-in-one FASTQ preprocessor. Bioinformatics. 2018;34 17:i884-i90. doi:https://doi.org/10.1093/bioinformatics/bty560.

38. Camacho C, Coulouris G, Avagyan V, Ma N, Papadopoulos J, Bealer K, et al. BLAST+: architecture and applications. BMC Bioinformatics. 2009;10 1:1-9. doi:https://doi.org/10.1186/1471-2105-10-421.

39. Simão FA, Waterhouse RM, Ioannidis P, Kriventseva EV and Zdobnov EM. BUSCO: assessing genome assembly and annotation completeness with single-copy orthologs. Bioinformatics. 2015;31 19:3210-2. doi:https://doi.org/10.1093/bioinformatics/btv351.

40. Li H. Aligning sequence reads, clone sequences and assembly contigs with BWA-MEM. arXiv: Genomics. 2013; doi:https://doi.org/10.6084/M9.FIGSHARE.963153.V1.

41. Li H, Handsaker B, Wysoker A, Fennell T, Ruan J, Homer N, et al. The sequence alignment/map format and SAMtools. Bioinformatics. 2009;25 16:2078-9. doi:https://doi.org/10.1093/bioinformatics/btp352.

42. Danecek P and McCarthy SA. BCFtools/csq: haplotype-aware variant consequences. Bioinformatics. 2017;33 13:2037-9. doi:https://doi.org/10.1093/bioinformatics/btx100.

43. Durand NC, Shamim MS, Machol I, Rao SS, Huntley MH, Lander ES, et al. Juicer provides a one-click system for analyzing loop-resolution Hi-C experiments. Cell Systems. 2016;3 1:95-8. doi:https://doi.org/10.1016/j.cels.2016.07.002.

44. Dudchenko O, Batra SS, Omer AD, Nyquist SK, Hoeger M, Durand NC, et al. De novo assembly of the Aedes aegypti genome using Hi-C yields chromosome-length scaffolds. Science. 2017;356 6333:92-5. doi:https://doi.org/10.1126/science.aal3327.

45. Flynn JM, Hubley R, Goubert C, Rosen J, Clark AG, Feschotte C, et al. RepeatModeler2 for automated genomic discovery of transposable element families. Proceedings of the National Academy of Sciences. 2020;117 17:9451-7. doi:https://doi.org/10.1073/pnas.1921046117.

46. Chen N. Using Repeat Masker to identify repetitive elements in genomic sequences. Current Protocols in Bioinformatics. 2004;5 1:4.10.1-4..4. doi:https://doi.org/10.1002/0471250953.bi0410s05.

47. Holt C and Yandell M. MAKER2: an annotation pipeline and genome-database management tool for second-generation genome projects. BMC Bioinformatics. 2011;12 1:1-14. doi:https://doi.org/10.1186/1471-2105-12-491.

48. Brůna T, Hoff KJ, Lomsadze A, Stanke M and Borodovsky M. BRAKER2: Automatic eukaryotic genome annotation with GeneMark-EP+ and AUGUSTUS supported by a protein database. NAR Genomics and Bioinformatics. 2021;3 1:lqaa108. doi:https://doi.org/10.1093/nargab/lqaa108.

49. Stanke M, Steinkamp R, Waack S and Morgenstern B. AUGUSTUS: a web server for gene finding in eukaryotes. Nucleic Acids Res. 2004;32 suppl_2:W309-W12. doi:https://doi.org/10.1093/nar/gkh379.

50. Brůna T, Lomsadze A and Borodovsky M. GeneMark-EP+: eukaryotic gene prediction with self-training in the space of genes and proteins. NAR Genomics and Bioinformatics. 2020;2 2:lqaa026. doi:https://doi.org/10.1093/nargab/lqaa026.

51. Kim D, Paggi JM, Park C, Bennett C and Salzberg SL. Graph-based genome alignment and genotyping with HISAT2 and HISAT-genotype. Nat Biotechnol. 2019;37 8:907-15. doi:https://doi.org/10.1038/s41587-019-0201-4.

52. Kriventseva EV, Kuznetsov D, Tegenfeldt F, Manni M, Dias R, Simão FA, et al. OrthoDB v10: sampling the diversity of animal, plant, fungal, protist, bacterial and viral genomes for evolutionary and functional annotations of orthologs. Nucleic Acids Res. 2019;47 D1:D807-D11. doi:https://doi.org/10.1093/nar/gky1053.

53. Kovaka S, Zimin AV, Pertea GM, Razaghi R, Salzberg SL and Pertea M. Transcriptome assembly from long-read RNA-seq alignments with StringTie2. Genome Biol. 2019;20 1:1-13. doi:https://doi.org/10.1186/s13059-019-1910-1.

54. Buchfink B, Xie C and Huson DH. Fast and sensitive protein alignment using DIAMOND. Nature Methods. 2015;12 1:59-60. doi:https://doi.org/10.1038/nmeth.3176.

55. El-Gebali S, Mistry J, Bateman A, Eddy SR, Luciani A, Potter SC, et al. The Pfam protein families database in 2019. Nucleic Acids Res. 2019;47 D1:D427-D32. doi:https://doi.org/10.1093/nar/gky995.

56. Letunic I and Bork P. 20 years of the SMART protein domain annotation resource. Nucleic

Acids Res. 2018;46 D1:D493-D6. doi:https://doi.org/10.1093/nar/gkx922.

57. Lewis TE, Sillitoe I, Dawson N, Lam SD, Clarke T, Lee D, et al. Gene3D: extensive prediction of globular domains in proteins. Nucleic Acids Res. 2018;46 D1:D435-D9. doi:https://doi.org/10.1093/nar/gkx1187.

58. Wilson D, Pethica R, Zhou Y, Talbot C, Vogel C, Madera M, et al. SUPERFAMILY—sophisticated comparative genomics, data mining, visualization and phylogeny. Nucleic Acids Res. 2009;37 suppl_1:D380-D6. doi:https://doi.org/10.1093/nar/gkn762.

59. Marchler-Bauer A, Bo Y, Han L, He J, Lanczycki CJ, Lu S, et al. CDD/SPARCLE: functional classification of proteins via subfamily domain architectures. Nucleic Acids Res. 2017;45 D1:D200-D3. doi:https://doi.org/10.1093/nar/gkw1129.

60. Finn RD, Attwood TK, Babbitt PC, Bateman A, Bork P, Bridge AJ, et al. InterPro in 2017—beyond protein family and domain annotations. Nucleic Acids Res. 2017;45 D1:D190-D9. doi:https://doi.org/10.1093/nar/gkw1107.

61. Huerta-Cepas J, Szklarczyk D, Heller D, Hernández-Plaza A, Forslund SK, Cook H, et al. eggNOG 5.0: a hierarchical, functionally and phylogenetically annotated orthology resource based on 5090 organisms and 2502 viruses. Nucleic Acids Res. 2019;47 D1:D309-D14. doi:https://doi.org/10.1093/molbev/msx148.

62. Huerta-Cepas J, Forslund K, Coelho LP, Szklarczyk D, Jensen LJ, Von Mering C, et al. Fast genome-wide functional annotation through orthology assignment by eggNOG-mapper. Mol Biol Evol. 2017;34 8:2115-22. doi:https://doi.org/10.1093/molbev/msx148.

63. Nawrocki EP and Eddy SR. Infernal 1.1: 100-fold faster RNA homology searches. Bioinformatics. 2013;29 22:2933-5. doi:https://doi.org/10.1093/bioinformatics/btt509.

64. Chan PP and Lowe TM. tRNAscan-SE: searching for tRNA genes in genomic sequences. Methods Mol Biol. 2019;1962:1-14. doi:https://doi.org/10.1007/978-1-4939-9173-0_1.

65. Krzywinski M, Schein J, Birol I, Connors J, Gascoyne R, Horsman D, et al. Circos: an information aesthetic for comparative genomics. Genome Res. 2009;19 9:1639-45. doi:https://doi.org/10.1101/gr.092759.109.

66. Emms DM and Kelly S. OrthoFinder: phylogenetic orthology inference for comparative genomics. Genome Biol. 2019;20 1:1-14. doi:https://doi.org/10.1186/s13059-019-1832-y.

67. Katoh K and Standley DM. MAFFT multiple sequence alignment software version 7: improvements in performance and usability. Mol Biol Evol. 2013;30 4:772-80. doi:https://doi.org/10.1093/molbev/mst010.

68. Criscuolo A and Gribaldo S. BMGE (Block Mapping and Gathering with Entropy): a new software for selection of phylogenetic informative regions from multiple sequence alignments. BMC Evol Biol. 2010;10 1:1-21. doi:https://doi.org/10.1186/1471-2148-10-210.

69. Kück P and Longo GC. FASconCAT-G: extensive functions for multiple sequence alignment preparations concerning phylogenetic studies. Frontiers in Zoology. 2014;11 1:1-8. doi:https://doi.org/10.1186/s12983-014-0081-x.

70. Minh BQ, Schmidt HA, Chernomor O, Schrempf D, Woodhams MD, Von Haeseler A, et al. IQ-TREE 2: new models and efficient methods for phylogenetic inference in the genomic era. Mol Biol Evol. 2020;37 5:1530-4. doi:https://doi.org/10.1093/molbev/msaa015.

71. Sanderson MJ. r8s: inferring absolute rates of molecular evolution and divergence times in the

absence of a molecular clock. Bioinformatics. 2003;19 2:301-2. doi:https://doi.org/10.1093/bioinformatics/19.2.301.

72. https://paleobiodb.org/. Accessed 15 Nov 2021.

73. Nel A, Roques P, Nel P, Prokin AA, Bourgoin T, Prokop J, et al. The earliest known holometabolous insects. Nature. 2013;503 7475:257-61. doi:https://doi.org/10.1038/nature1262.

74. Misof B, Liu S, Meusemann K, Peters RS, Donath A, Mayer C, et al. Phylogenomics resolves the timing and pattern of insect evolution. Science. 2014;346 6210:763-7. doi:https://doi.org/10.1126/science.1257570.

75. Han MV, Thomas GW, Lugo-Martinez J and Hahn MW. Estimating gene gain and loss rates in the presence of error in genome assembly and annotation using CAFE 3. Mol Biol Evol. 2013;30 8:1987-97. doi:https://doi.org/10.1093/molbev/mst100.

76. Yu G, Wang L-G, Han Y and He Q-Y. clusterProfiler: an R package for comparing biological themes among gene clusters. OMICS. 2012;16 5:284-7. doi:https://doi.org/10.1089/omi.2011.0118.

77. Yang Z. PAML 4: phylogenetic analysis by maximum likelihood. Mol Biol Evol. 2007;24 8:1586-91. doi:https://doi.org/10.1093/molbev/msm088.

78. Yang Z, Wong WS and Nielsen R. Bayes empirical Bayes inference of amino acid sites under positive selection. Mol Biol Evol. 2005;22 4:1107-18. doi:https://doi.org/10.1093/molbev/msi097.

79. Wang S, Lorenzen MD, Beeman RW and Brown SJ. Analysis of repetitive DNA distribution patterns in the *Tribolium castaneum* genome. Genome Biol. 2008;9 3:1-14. doi:https://doi.org/10.1186/gb-2008-9-3-r61.

80. Steinegger M and Söding J. MMseqs2: sensitive protein sequence searching for the analysis of massive data sets. Nat Biotechnol. 2017;35:1026-8. doi:https://doi.org/10.1038/nbt.3988.

81. Wang Y, Tang H, DeBarry JD, Tan X, Li J, Wang X, et al. MCScanX: a toolkit for detection and evolutionary analysis of gene synteny and collinearity. Nucleic Acids Res. 2012;40 7:e49-e. doi:https://doi.org/10.1093/nar/gkr1293.

82. Chen C, Chen H, Zhang Y, Thomas HR, Frank MH, He Y, et al. TBtools: an integrative toolkit developed for interactive analyses of big biological data. Molecular Plant. 2020;13 8:1194-202. doi:https://doi.org/10.1016/j.molp.2020.06.009.

83. Langmead B and Salzberg SL. Fast gapped read alignment with Bowtie 2. Nature Methods. 2012;9 4:357-9. doi:https://doi.org/10.1038/nmeth.1923.

84. Li B and Dewey CN. RSEM: accurate transcript quantification from RNA-Seq data with or without a reference genome. BMC Bioinformatics. 2011;12 1:1-16.

85. Gregory R. Warnes BB, Thomas Lumley, Randall C Johnson. . gmodels: Various R Programming Tools for Model Fitting. 2.18.1 ed. 2018.

86. Love MI, Huber W and Anders S. Moderated estimation of fold change and disper sion for RNA seq data with DESeq2. Genome Biol. 2014;15 12:550. doi:https://doi.org/10.1186/s13059-014-0550-8.

87. Robinson MD, McCarthy DJ and Smyth GK. edgeR: a Bioconductor package for differential expression analysis of digital gene expression data. Bioinformatics. 2010;26 1:139-40. doi:https://doi.org/10.1093/bioinformatics/btp616.

88. Meyer JM, Markov GV, Baskaran P, Herrmann M, Sommer RJ and Rödelsperger C. Draft genome of the scarab beetle Oryctes borbonicus on La Réunion Island. Genome Biol Evol. 2016;8 7:2093-105. doi:https://doi.org/10.1093/gbe/evw133.

89. McKenna DD. Beetle genomes in the 21st century: prospects, progress and priorities. Current Opinion in Insect Science. 2018;25:76-82. doi:https://doi.org/10.1016/j.cois.2017.12.002.

90. Lee JH, Jung M, Shin Y, Kim I-W, Seo M, Kim M, et al. Draft Genome of the Edible Oriental Insect *Protaetia brevitarsis seulensis*. Frontiers in Genetics. 2020;11:1741. doi:https://doi.org/10.3389/fgene.2020.593994.

91. Charlesworth B and Barton N. Genome size: does bigger mean worse? Curr Biol. 2004;14 6:R233-R5. doi:https://doi.org/10.1016/j.cub.2004.02.054.

92. McKenna DD, Shin S, Ahrens D, Balke M, Beza-Beza C, Clarke DJ, et al. The evolution and genomic basis of beetle diversity. Proceedings of the National Academy of Sciences. 2019;116 49:24729-37. doi:https://doi.org/10.1073/pnas.1909655116.

93. Calderón-Cortés N, Quesada M, Watanabe H, Cano-Camacho H and Oyama K. Endogenous plant cell wall digestion: a key mechanism in insect evolution. Annu Rev Ecol Evol Syst. 2012;43:45-71. doi:https://doi.org/10.1146/annurev-ecolsys-110411-160312.

94. Dunn MJ, Kinney GM, Washington PM, Berman J and Anderson MZ. Functional diversification accompanies gene family expansion of MED2 homologs in Candida albicans. PLoS Genet. 2018;14 4:e1007326. doi:https://doi.org/10.1371/journal.pgen.1007326.

95. MacGillivray DM and Kollmann TR. The role of environmental factors in modulating immune responses in early life. Front Immunol. 2014;5:434. doi:https://doi.org/10.3389/fimmu.2014.00434.

96. Booker TR, Jackson BC and Keightley PD. Detecting positive selection in the genome. BMC Biol. 2017;15 1:1-10. doi:https://doi.org/10.1186/s12915-017-0434-y.

97. Pearce SL, Clarke DF, East PD, Elfekih S, Gordon K, Jermiin LS, et al. Genomic innovations, transcriptional plasticity and gene loss underlying the evolution and divergence of two highly polyphagous and invasive *Helicoverpa* pest species. BMC Biol. 2017;15 1:1-30. doi:https://doi.org/10.1186/s12915-017-0402-6.

98. Eichler EE and Sankoff D. Structural dynamics of eukaryotic chromosome evolution. Science. 2003;301 5634:793-7. doi:https://doi.org/10.1126/science.1086132.

99. Lorenzen MD, Doyungan Z, Savard J, Snow K, Crumly LR, Shippy TD, et al. Genetic linkage maps of the red flour beetle, *Tribolium castaneum*, based on bacterial artificial chromosomes and expressed sequence tags. Genetics. 2005;170 2:741-7. doi:https://doi.org/10.1534/genetics.104.032227.

100. d'Alencon E, Sezutsu H, Legeai F, Permal E, Bernard-Samain S, Gimenez S, et al. Extensive synteny conservation of holocentric chromosomes in Lepidoptera despite high rates of local genome rearrangements. Proceedings of the National Academy of Sciences. 2010;107 17:7680-5. doi:https://doi.org/10.1073/pnas.0910413107.

101. Pal A and Vicoso B. The X chromosome of hemipteran insects: conservation, dosage compensation and sex-biased expression. Genome Biol Evol. 2015;7 12:3259-68. doi:https://doi.org/10.1093/gbe/evv215.

102. Li Y, Zhang B and Moran NA. The aphid X chromosome is a dangerous place for functionally important genes: diverse evolution of hemipteran genomes based on chromosome-level

assemblies. Mol Biol Evol. 2020;37 8:2357-68. doi:https://doi.org/10.1093/molbev/msaa095.
103. Mathers TC, Wouters RH, Mugford ST, Swarbreck D, Van Oosterhout C and Hogenhout SA. Chromosome-scale genome assemblies of aphids reveal extensively rearranged autosomes and long-term conservation of the X chromosome. Mol Biol Evol. 2021;38 3:856-75. doi:https://doi.org/10.1093/molbev/msaa246.
104. Lebreton A, Zeng Q, Miyauchi S, Kohler A, Dai Y-C and Martin FM. Evolution of the mode of nutrition in symbiotic and saprotrophic fungi in forest ecosystems. Annu Rev Ecol Evol Syst. 2021;52:385-404.
105. Wei P, Li Y, Lai D, Geng L, Liu C, Zhang J, et al. *Protaetia brevitarsis* larvae can feed on and convert spent mushroom substrate from *Auricularia auricula* and *Lentinula edodes* cultivation. Waste Management. 2020;114:234–9. doi:https://doi.org/10.1016/j.wasman.2020.07.009.

Figure legends

Figure 1. Wood fiber degradation by larva of *Trypoxylus dichotomus*. **a.** 3rd instar larva. **b.** Wood fiber before degradation. **c.** Wood fiber after degradation.

Figure 2. Genome assembly and assessment of *Trypoxylus dichotomus*. **a.** Accumulated graph of contig length. **b.** Hi-C heatmap showing 10 chromosomes (Chr1 to Chr10) arranged by length.

Figure 3. Circos graph of chromosome-level genome of *Trypoxylus dichotomus*, showing length of chromosomes, GC-content, density of protein-coding genes and repetitive elements (DNA/SINE/LINE/LTR). (Sliding window size = 100 kb)

Figure 4. a. Phylogenetic tree and statistics of orthologs. Left: Phylogenetic tree and divergence times of beetles based on 1,108 single-copy orthologs; branch values representing the number of expanded, contracted and rapidly evolving gene families (bold) respectively; color value scale representing divisions of geologic time, abbreviations standing for Silurian (S), Devonian (D), Carboniferous (C), Permian (P), Triassic (Tr), Jurassic (J), Cretaceous (K) and Tertiary (T). Right: statistics of orthologous genes among the 14 insect species; '1:1:1' representing shared single-copy genes, 'N:N:N' representing multi-copy genes shared by all species, 'Coleoptera' representing orthologs unique to Coleoptera, and 'Others' representing unclassified orthologs. **b.** Chromosome-level genome collinearity between *Trypoxylus dichotomus* and *Tribolium castaneum*; 'TdChr' representing chromosomes of *T. dichotomus*, 'TcChr' representing chromosomes of *Tri. castaneum*.

Figure 5. Expanded gene families and functional enrichment. **a.** Top twenty significantly expanded gene families. **b.** GO enrichment of rapidly expanded gene families. **c.** KEGG enrichment of rapidly expanded gene families.

Figure 6. Sample correlation of intestinal gene expression patterns among four groups of *Trypoxylus dichotomus*. Each group consists of six replicates. **a.** Principal component analysis (PCA) diagram; circle indicates larva feeding sawdust, square indicates larva feeding mushroom-residue, green indicates midgut, red indicates hindgut. **b.** Pearson correlation coefficient (PCC) heatmap; colors and values indicate the relationship between paired samples (the darker the color and larger value mean the closer the relationship), value ≥ 0.8 shows the good repeatability. SM, midgut from sawdust; SH, hindgut from sawdust; MM, midgut from mushroom-residue; MH, hindgut from mushroom-residue.

Figure 7. Heatmaps of differentially expressed digestion-related genes among four groups of the rhinoceros beetle. Each group consists of six replicates. Colors indicate a higher (red) or lower (blue) gene expression in each sample for every gene, identified by the FPKM value. Gene expression clustering between midgut and hindgut from sawdust group (**a**) and mushroom-residue group (**b**). Gene expression clustering of midgut (**c**) and hindgut (**d**) between sawdust and mushroom-residue groups. SM, midgut from sawdust; SH, hindgut from sawdust; MM, midgut from mushroom-residue; MH, hindgut from mushroom-residue.

Figure 1

Click here to access/download;Figure;Figure 1.tif

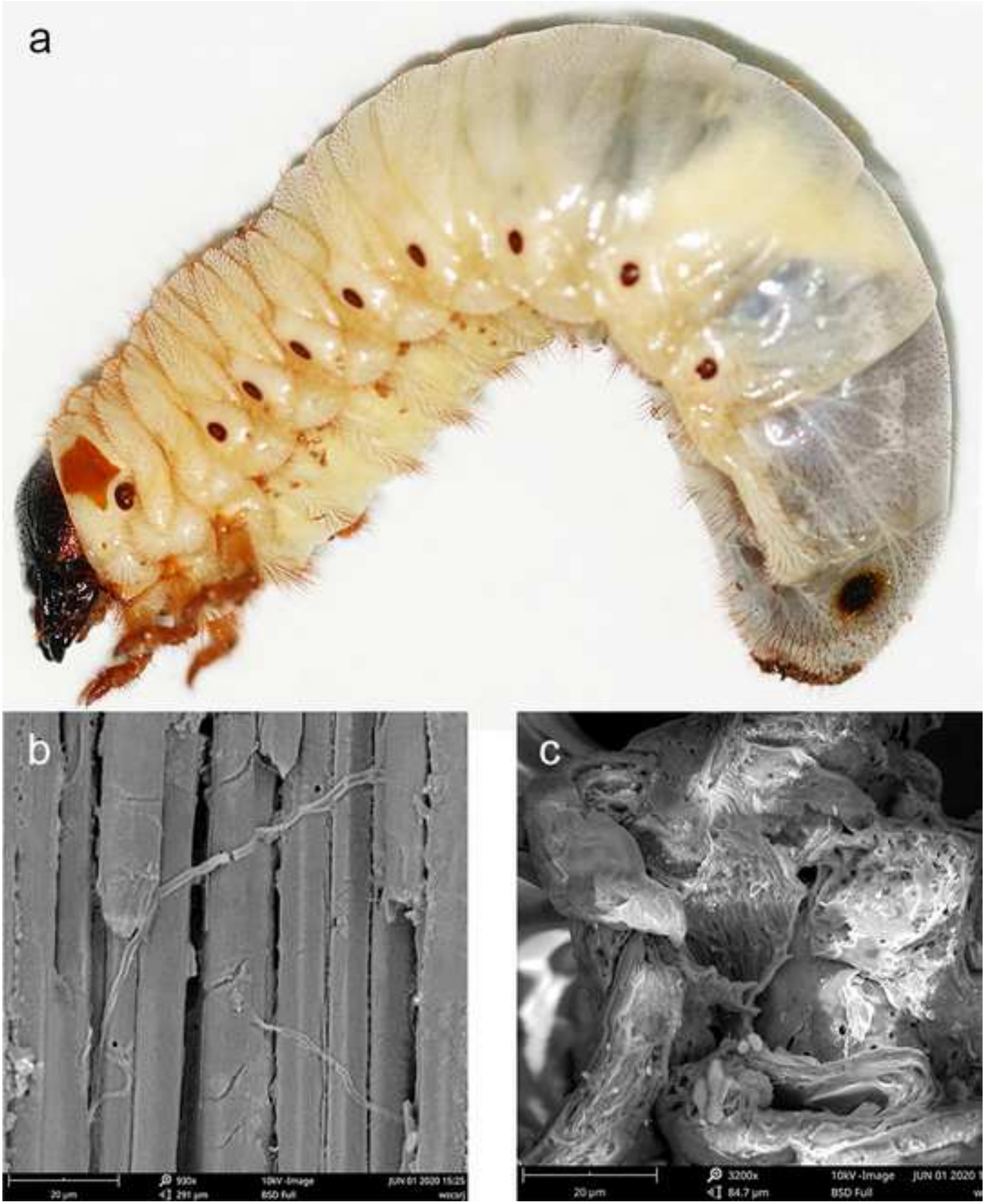

Figure 2

Click here to access/download;Figure;Figure 2.tif

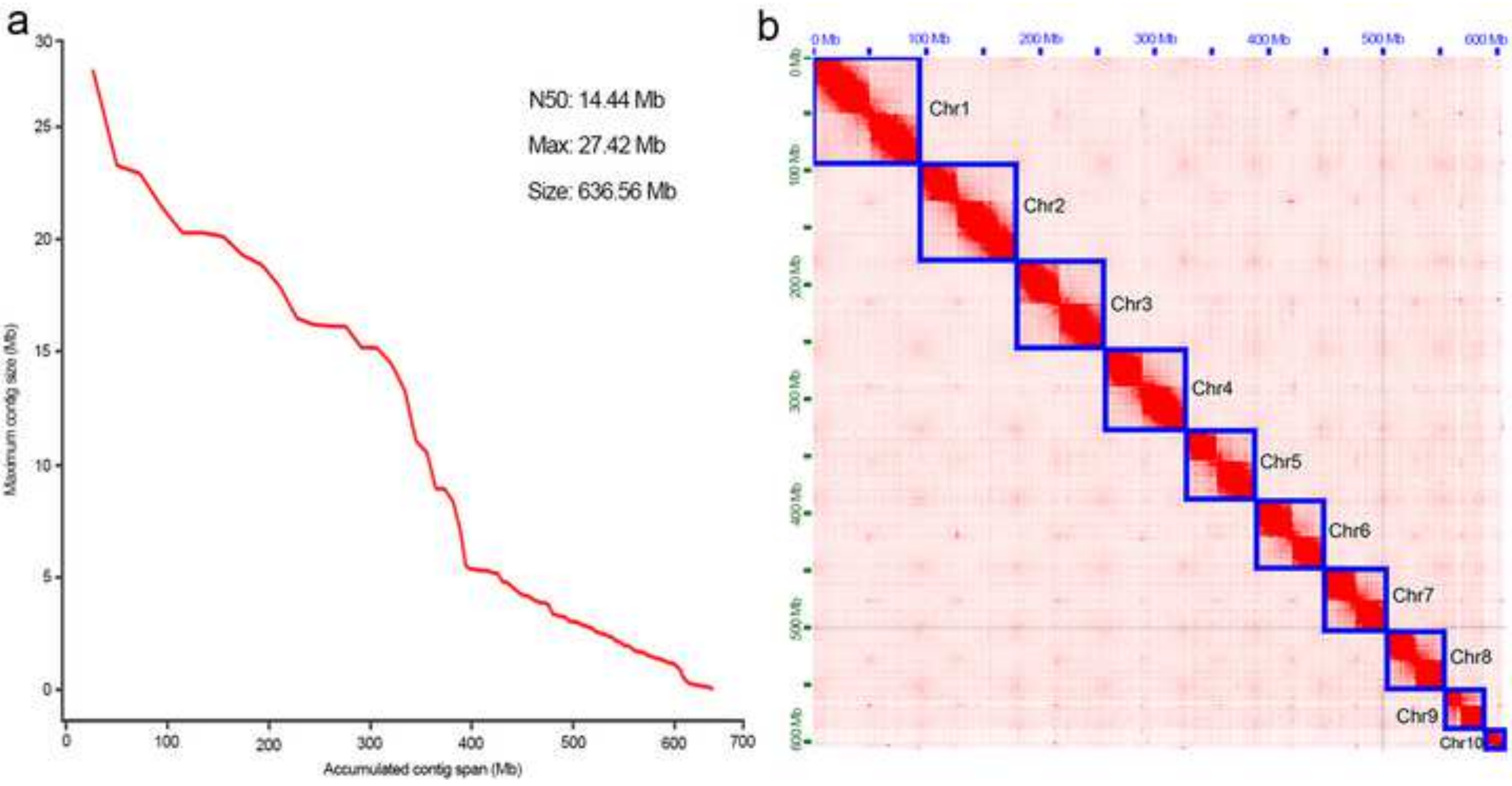

Figure 3

Click here to access/download;Figure;Figure 3.tif

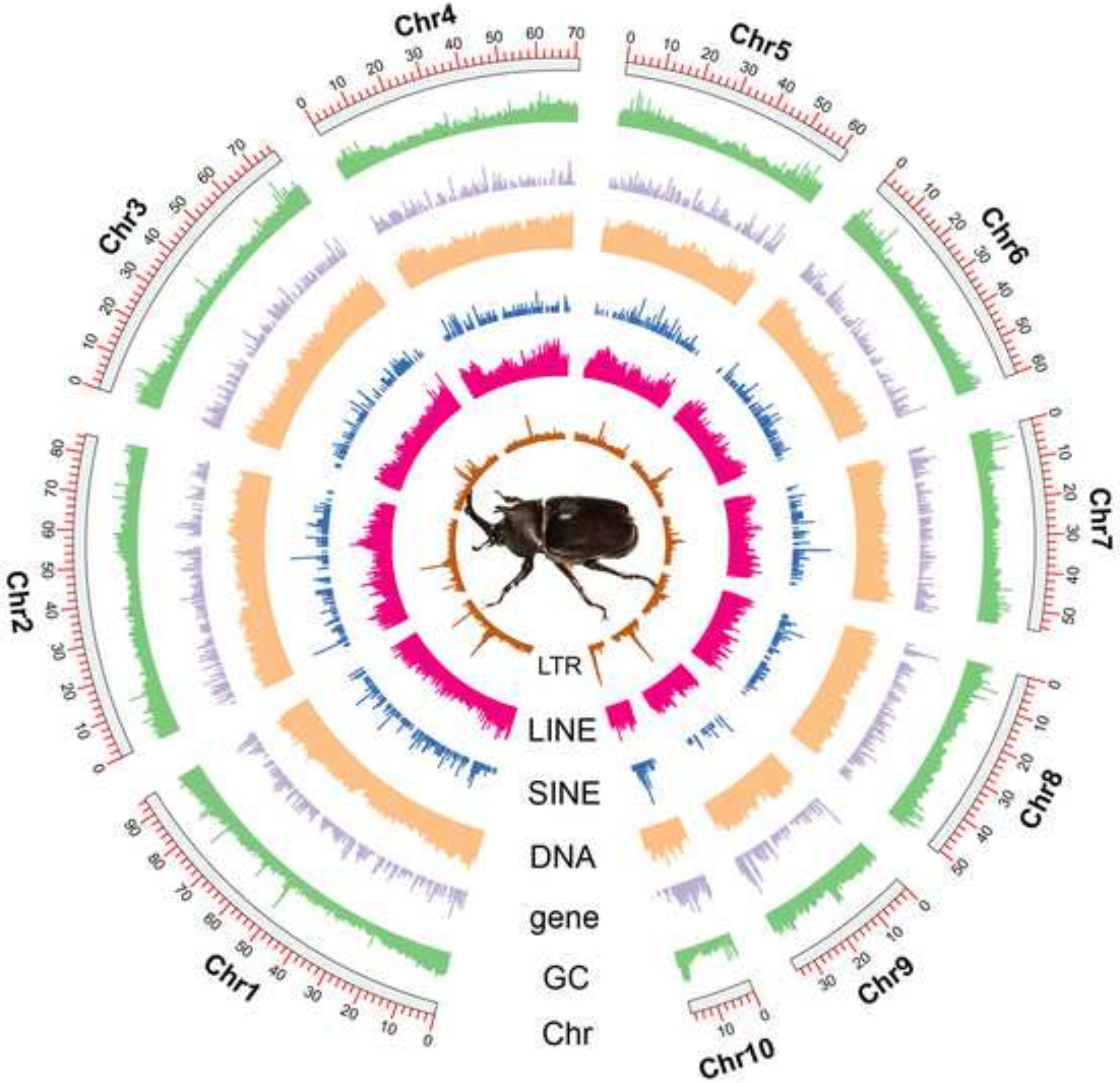

Figure 4

Click here to access/download;Figure;Figure 4.tif

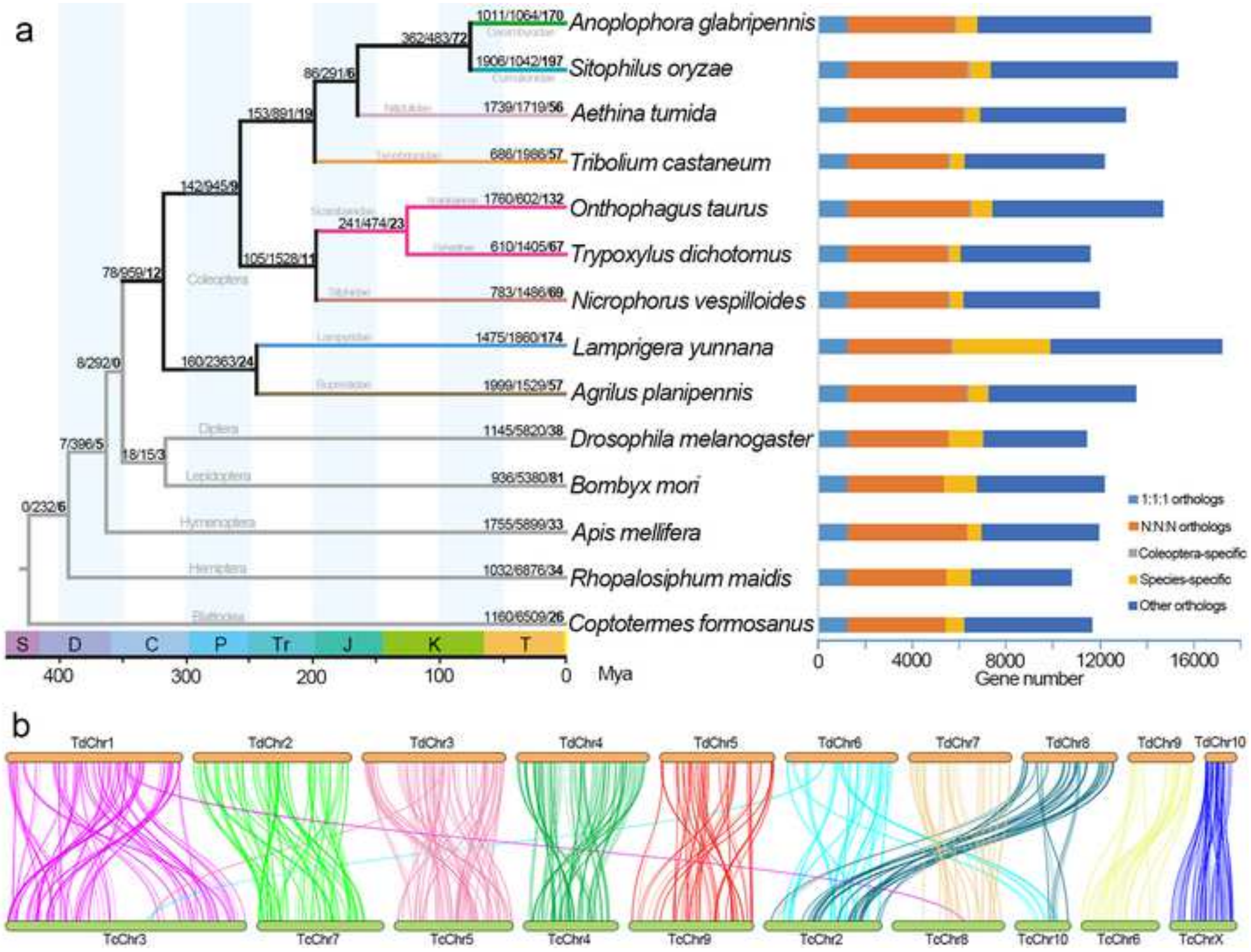

Figure 5

Click here to access/download;Figure;Figure 5.tif

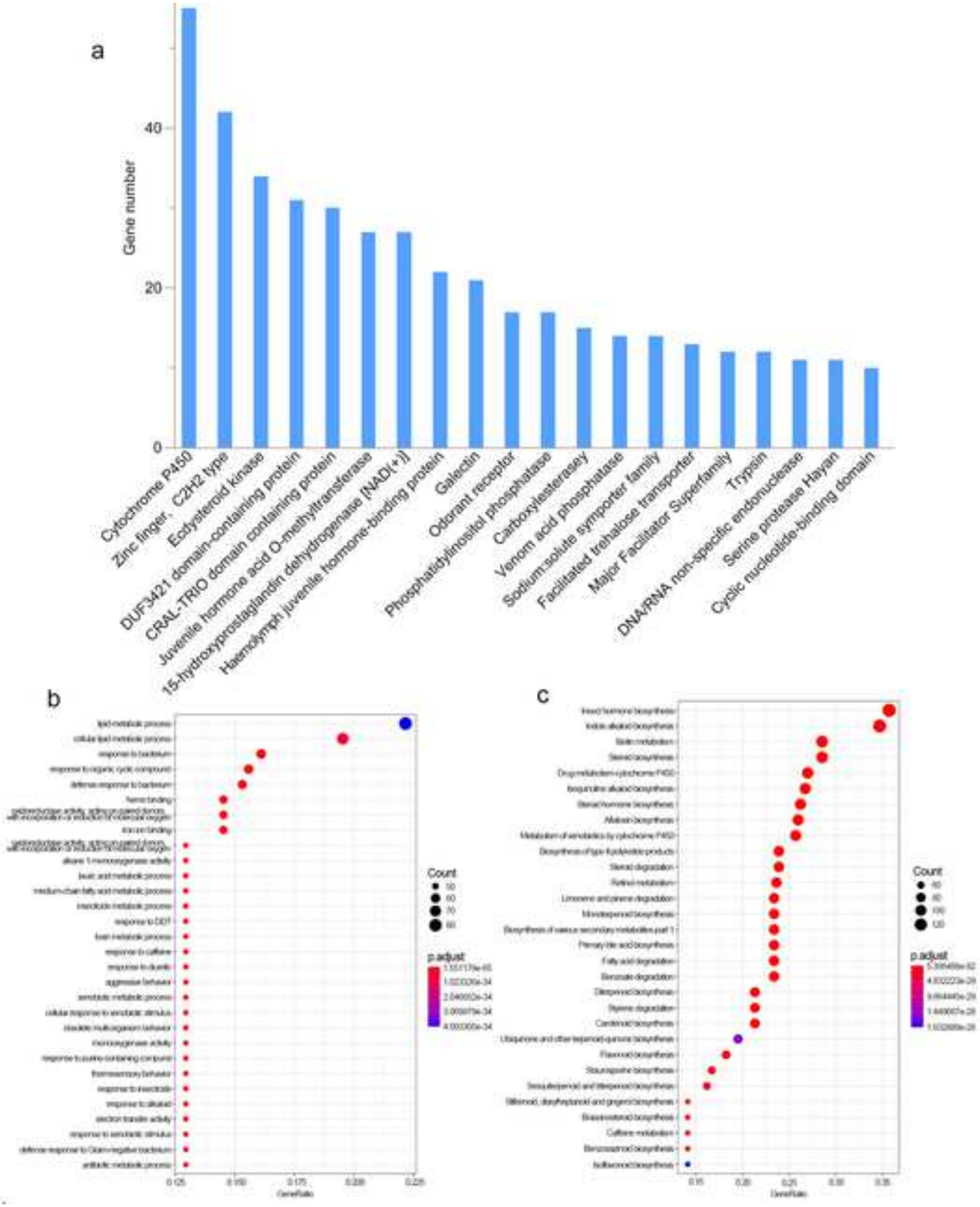

Figure 6

Click here to access/download;Figure;Figure 6.tif

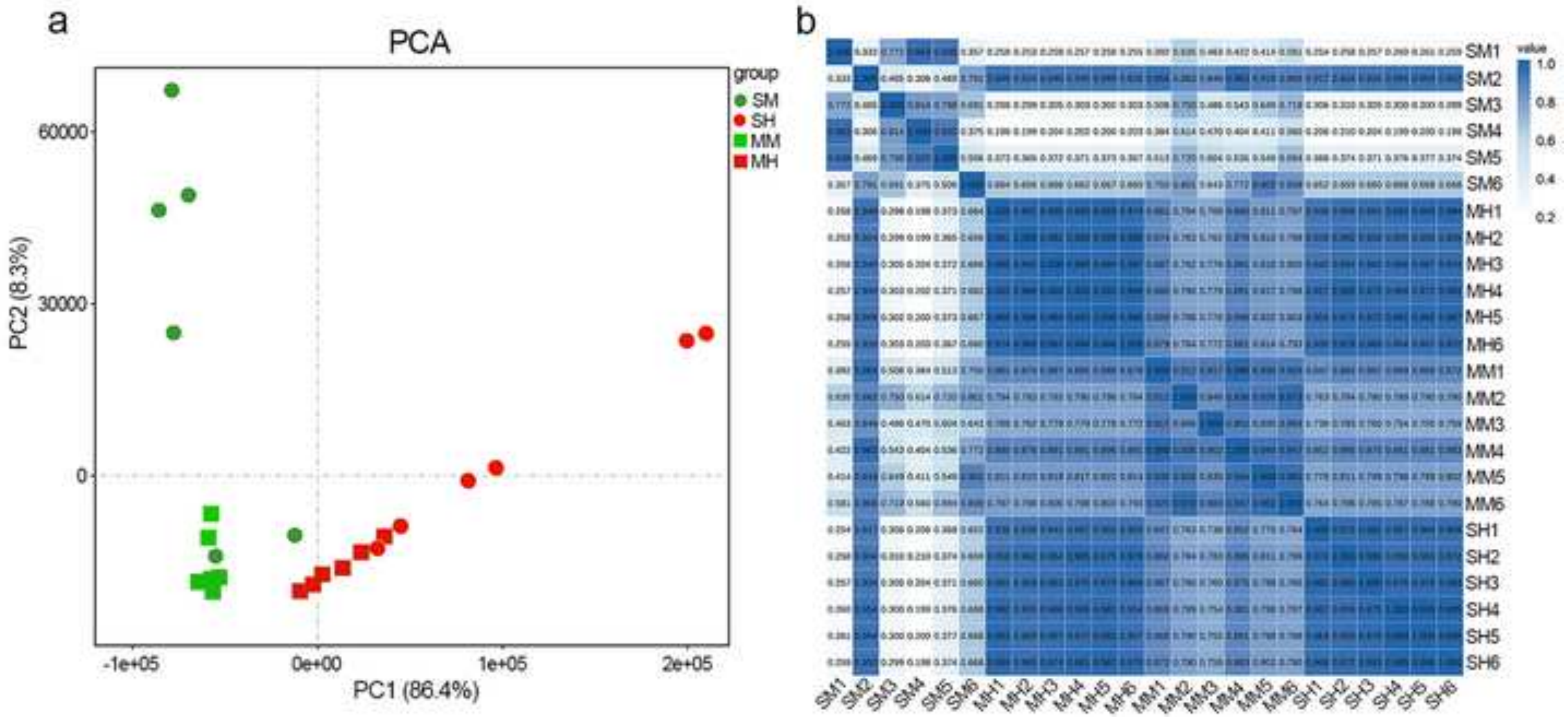

Figure 7

Click here to access/download;Figure;Figure 7.tif

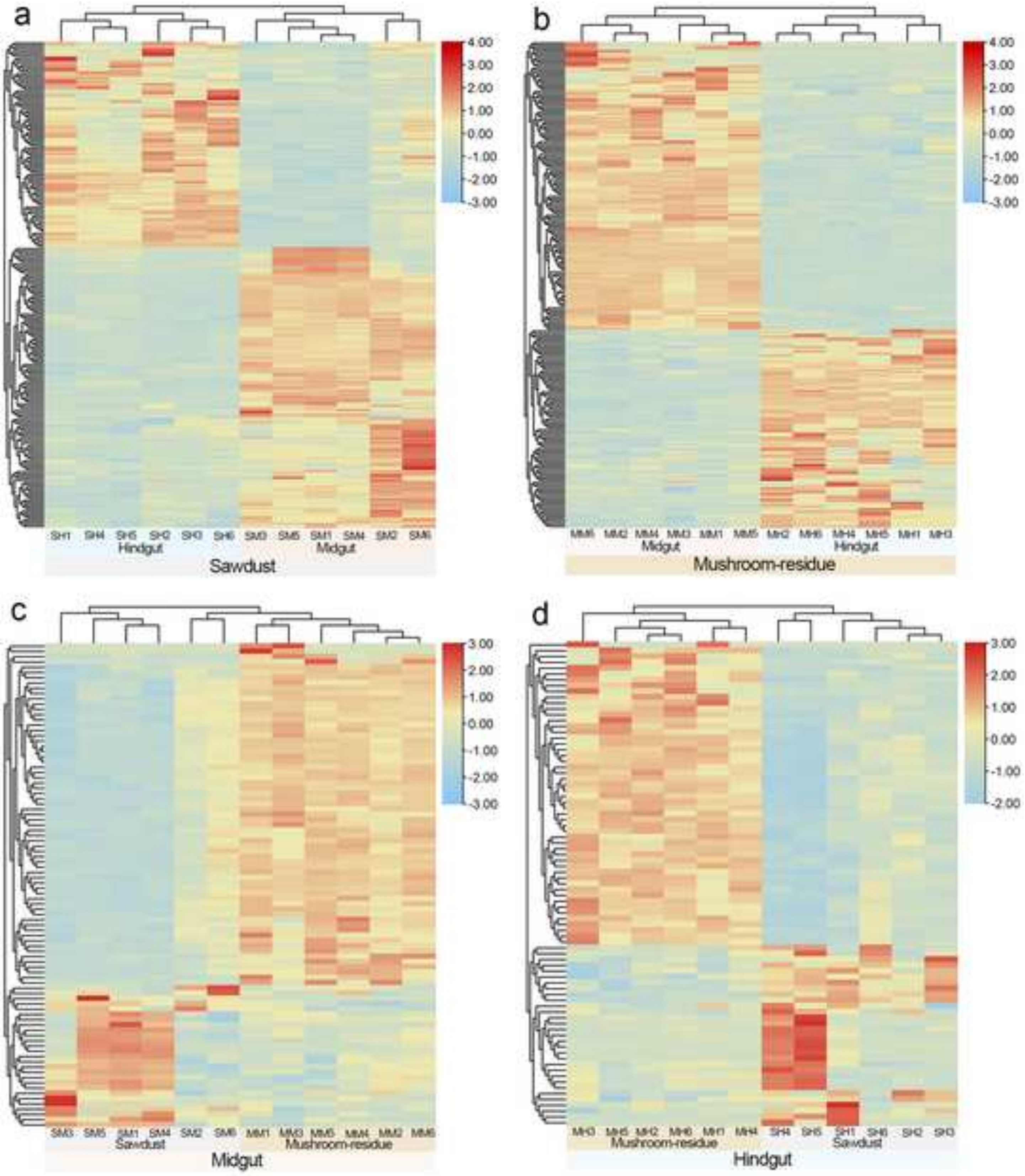

Click here to access/download
Supplementary Material
Figures S1-2.docx

Click here to access/download
Supplementary Material
Table S2.xlsx

Click here to access/download
Supplementary Material
Table S6.xls

Click here to access/download
Supplementary Material
Table S7.xls

Click here to access/download
Supplementary Material
Table S10.xlsx

Click here to access/download
Supplementary Material
Table S11.xlsx

Click here to access/download
Supplementary Material
Table S12.xlsx

Click here to access/download
Supplementary Material
Table S13.xlsx

Click here to access/download
Supplementary Material
Table S14.xlsx

Click here to access/download
Supplementary Material
Table S1, 3-5&8-9.docx

Click here to access/download
Supplementary Material
Supporting information.docx
