# Supplementary material for: A chromosome-level genome assembly and intestinal transcriptome of Trypoxylus dichotomus (Coleoptera: Scarabaeidae) to understand its lignocellulose digestion ability
Source: Gigascience. 2022 Jun 28;11:giac059. doi: 10.1093/gigascience/giac059 (PMC9239855; doi:10.1093/gigascience/giac059)
Supplement: giac059_GIGA-D-21-00415_Revision_2 [file giac059_giga-d-21-00415_revision_2.pdf]

GigaScience

A chromosome-level genome assembly and intestinal transcriptome of Trypoxylus dichotomus (Coleoptera: Scarabaeidae) to understand its lignocellulose digestion ability

--Manuscript Draft--

**Manuscript Number:**	GIGA-D-21-00415R2
**Full Title:**	A chromosome-level genome assembly and intestinal transcriptome of Trypoxylus dichotomus (Coleoptera: Scarabaeidae) to understand its lignocellulose digestion ability
**Article Type:**	Research
**Funding Information:**	Cooperation Project of Zhejiang Province and Chinese Academy of Forestry (2020SY08) — Dr. Junhao Huang
**Abstract:**	Lignocellulose, as the key structural component of plant biomass, is a recalcitrant structure, difficult to degrade. The traditional management of plant waste including landfill and incineration usually causes serious environmental pollution and health problems. Interestingly, the xylophagous beetle, Trypoxylus dichotomus can decompose lignocellulosic biomass. However, the genomics around the digestion mechanism of this beetle remain to be elucidated. Here, we assembled the genome of T. dichotomus , showing that the draft genome size of T. dichotomus is 636.27 Mb, with 95.37% scaffolds anchored onto 10 chromosomes. Phylogenetic results indicated that a divergent evolution between the ancestors of T. dichotomus and the closely related scarabaeid species Onthophagus taurus occurred in the early Cretaceous (120 Mya). Through gene family evolution analysis, we found 67 rapidly evolving gene families, within which there were two digestive gene families (encoding Trypsin and Enoyl-(Acyl carrier protein) reductase) that have experienced significant expansion, indicating that they may contribute to the high degradation efficiency of lignocellulose in T. dichotomus . Additionally, events of chromosome breakage and rearrangement were observed by synteny analysis during the evolution of T. dichotomus due to chromosomes 6 and 8 of T. dichotomus being intersected with chromosomes 2 and 10 of Tribolium castaneum , respectively. Furthermore, the comparative transcriptome analyses of larval guts showed that the digestion-related genes were more commonly expressed in the midgut or mushroom-residue group than the hindgut or sawdust group. This study reports the well-assembled and annotated genome of T. dichotomus , providing genomic and transcriptomic bases for further understanding the functional and evolutionary mechanisms of lignocellulose digestion in T. dichotomus .
**Corresponding Author:**	Junhao Huang<br>Zhejiang A&F University<br>Hangzhou, Zhejiang CHINA
**Corresponding Author Secondary Information:**	
**Corresponding Author's Institution:**	Zhejiang A&F University
**Corresponding Author's Secondary Institution:**	
**First Author:**	Qingyun Wang
**First Author Secondary Information:**	
**Order of Authors:**	Qingyun Wang
	Liwei Liu
	Sujiong Zhang
	Hong Wu
	Junhao Huang

Powered by Editorial Manager® and ProduXion Manager® from Aries Systems Corporation

Order of Authors Secondary Information:	
Response to Reviewers:	Dear editor and reviewers,<br><br>Many thanks to you for the valuable comments. All of the suggestions and problems you raised have been reconsidered and responded in the following two responses (Responses 1 and 2), in which the "Response 1" has been rewritten and attached below. The corresponding changes have been made in the revised manuscript as well. Moreover, the second revised manuscript has been edited by the paid-for-service editing company (Charlesworth). We hope that the revised manuscript will meet the requirements of this journal. Thanks again for your suggestions on the improvement of this manuscript.<br><br><br>Response 1 to the manuscript GIGA-D-21-00415<br><br>Response to Reviewer 1:<br>(1) Given the recent release of another assembly of this species (mentioned lines 328-329) which was generated using PacBio reads, the authors are missing out on an interesting comparison between data types (and as-is, I can't evaluate the truth of lines 526-527 that this is the first chromosome-scale assembly for the species). I think some comparative analyses would be quite interesting of these 2 raw assemblies, or at the very least more than a one-line mention that another assembly exists.<br>Authors response: According to the reviewer's recommendation, the BUSCO assessment of the recently released genome assembly has been performed. The result shows that there are 99.7% BUSCO completeness, including 98.5% single-copy and 1.2% duplicated BUSCOs, in the recently released genome of Trypoxylus dichotomus. In addition, the comparison has been performed on the important assembly information (scaffold/contig N50 length, scaffold/contig number) between these two draft genome assemblies of this beetle. All of these information has been concluded in the following sentences highlighted in the lines 360-366.<br>"Its BUSCO assessment (n = 1,367) identified 1,363 (99.7%) complete BUSCOs, comprised of 1,347 (98.5%) single-copy and 16 (1.2%) duplicated BUSCOs. In comparison, the size of our genome was smaller than that of the released genome assembly, probably due to the scaffold assembly level we used. Furthermore, our genome assembly showed a longer scaffold N50 (71.04 Mb) and a smaller scaffold number (414) than that of the released one."<br>Besides, the original incorrect description of the recently released genome assembly, i.e., "scaffold N50 length of 7.93 Mb; scaffold number of 2,347", has been amended as "contig N50 length of 7.93 Mb; contig number of 2,347" in the lines 359.<br>Response to the above-mentioned comment "I can't evaluate the truth of lines 526-527 that this is the first chromosome-scale assembly for the species": Although there has been no chromosome-level genome assembly released for Trypoxylus dichotomus so far, the reviewer questioned whether or not it is the first chromosome-level assembly for this species. To make the results more scientifically credible, we have deleted the words "first" and "firstly" in the present statement of chromosome-level genome of T. dichotomus (lines 89 and 574).<br><br>(2) Overall, I am not convinced by the mid vs. hindgut and mushroom vs. wood fiber differential expression analysis. This is both for lack of some methodological detail (pertaining the diets that larvae were reared on) and interpretation and resulting conclusion statements about particular parts of the gut having more digestive ability for wood vs. fungi. The latter could probably be addressed by toning down some of the statements of those results or at least more accurately describing patterns in the data.<br>Authors response: The larvae rearing methods have been detailed in the revised manuscript. The updated statement is "The similar-sized larvae were divided into two groups and reared with high-temperature sterilized sawdust and mushroom-residue (composed of wood fiber and fungal mycelia) at 20–25°C and 50–60% humidity for 2 months, separately" (lines 115-118).<br>Furthermore, the statements about the results and discussion of comparative transcriptome analyses of larval gut have been toned down according to the reviewer's comment. Firstly, the original statement of comparative analyses between midgut and hindgut from different diets has been amended as "These results indicate that more digestion-related genes are highly expressed in the midgut than the hindgut of larvae, regardless of food habit. Thus, the digestion of lignocellulose in larvae may require

more digestive enzymes in the midgut than in the hindgut” (lines 539-543). Secondly, the original statement of comparative analyses between sawdust and mushroom-residue groups from different gut tissues has been modified as “These results suggest that digestion of mushroom-residue might require a greater digestive ability than that of sawdust for the larvae of T. dichotomus, which is probably due to the complex components of mushroom-residue, including not only wood fiber but also fungal mycelia” (lines 555-559).

(3) Finally, I have made several comments in the first few paragraphs concerning grammatical flow, sentence structure, and English considerations, but not throughout the rest of the study (although there were many English errors in the rest of the manuscript). The entire manuscript requires editing from a native English speaker before being fit for publication, as there are many unclear and incorrect statements stemming from grammatical/English mistakes.
Authors response: The reviewer’s suggestion is followed in the revised manuscript presently. The entire manuscript has been edited by a native English speaker, in which the previous grammatical/English mistakes have been largely found out and corrected in the present version of manuscript.

SPECIFIC COMMENTS

(4) Line 45. "consists in almost all kinds" is a bit awkward. Perhaps "lignocellulosic biomass is found in all kinds"?
Authors response: The reviewer’s suggestion is accepted in the revised manuscript. The original sentence has been modified and simplified as “As a key structural component of plant biomass and an important route of carbon fixation, lignocellulosic biomass is found in all kinds of living and dead plants” (lines 42-43).

(5) Line 48. "protecting the" should be "protects".
Authors response: Yes, the original sentence has been modified as “……, which form a complex cross-linked and recalcitrant structure that that protects carbohydrates from decomposition by microorganisms or enzymes [2, 3]” (lines 45-46).

(6) Lines 49-51. The "Thus" beginning of this sentence isn't really following the point of the preceding sentence. Perhaps reordering the sentence to something like: "Recycling plant waste produced by human production is a noteworthy environmental issue."
Authors response: Yes, the original description is problematic. In order to make the statement more clear and specific, the original statement has been modified as “The traditional management of plant waste is usually done via landfill or incineration, which causes serious environmental pollution and health problems [4]. Thus, recycling plant wastes produced by human production is a noteworthy environmental issue [5]” (lines 46-50). Moreover, the corresponding reference [4] “Shah SN, Mo KH, Yap SP, Yang J and Ling T-C. Lightweight foamed concrete as a promising avenue for incorporating waste materials: A review. Resources, Conservation and Recycling. 2021;164:105103” has been added in “References” (lines 644-646).

(7) Line 56. I suggest rewording this sentence to make the subject of the latter half of the sentence clearer (the subject of the first half is "lignocellulose decomposition", but in the second half it is just "lignocellulose").
Authors response: The original description, “Lignocellulose decomposition is not a common trait among animals because of its complex structural and chemical mechanisms for resisting assault [13, 14], but wood-feeding insects, such as termites, wood-feeding cockroaches, beetles and wood wasps capitalize a high-efficient digestion ability of lignocellulosic biomass”, has been amended as “Due to the complex structural and chemical mechanisms of lignocellulose, lignocellulose decomposition is not common among animals [9, 10] except for wood-feeding insects such as termites, wood-feeding cockroaches, beetles, and wood wasps [7, 8, 10-12]” (lines 54-57).

(8) Line 59. Should be "highly efficient".
Authors response: The original statement, “……, but wood-feeding insects, such as termites, wood-feeding cockroaches, beetles and wood wasps capitalize a high-efficient digestion ability of lignocellulosic biomass”, has been modified as “…… except for wood-feeding insects such as termites, wood-feeding cockroaches, beetles, and wood wasps [7, 8, 10-12]” (lines 55-57).

Powered by Editorial Manager® and ProduXion Manager® from Aries Systems Corporation

(9) Line 60. Should be present tense. Same for the next sentence as well.
Authors response: Yes, these sentences should be in the present tense. This sentence has been amended as “These insects are involved in ……” (line 57). Moreover, the next sentence is modified as “Among them, xylophagous termites are the most well-known ……” (lines 59-60).

(10) Lines 63-64. 1) The termites did not receive research achievements. They were used as study systems. 2) Additionally, while comparison to termites makes biological sense, there is no mention of microbial-aided digestion in the manuscript. Is anything known about the microbiome of this beetle species as compared to termites? Or is their digestion ability fully the beetle's doing and not their gut microbiome?
Authors response:
1) The reviewer's warning is reasonable. The original description has been modified as “Among them, xylophagous termites are the most well-known of efficient lignocellulose digesters, having been studied in detail including in terms of functional genomics and symbiotic intestinal microorganisms [14-16]” (lines 59-62).
2) Yes, comparisons have not yet been made on the microbial-aided digestion between Trypoxylus dichotomus and termites so far. Although the roles what larval gut microbiome of T. dichotomus play on the digestion ability has not been reported, the recent unpublished research shows that intestinal microorganisms of T. dichotomus larvae also play a role in degradation of lignocellulose.

(11) Line 67. I don't think "biodegradability" really fits here if the authors are referring to the ability to break down the substances from the point of view of the insects.
Authors response: The word “biodegradability” has been replaced with the phrase “bio-degradation ability” (line 64), which refers to the lignocellulose degradation ability of the xylophagous insects (except for termites).

(12) Line 73. It "can", not "could".
Authors response: Yes, it refers to the larval ability of Trypoxylus dichotomus, so the word “could” has been replaced by “can” (line 70).

(13) Lines 109-110. Was there rationale for choosing female tissue for some data and male tissue for others?
Authors response: Yes, the rationale for choosing female tissue for some data and male tissue for others are as follows:
1) The sex determination system of Trypoxylus dichotomus is XY, in which the Y chromosome is much smaller than X chromosome;
2) There were only a pair of newly emerged beetles of different genders obtained for genome sequencing.
Thus, to meet the sequencing requirements, the muscle of female thoracic muscle was prepared for Illumina and Nanopore sequencing, while the male thoracic muscle was then chosen for Hi-C and RNA sequencing.
The original statement has been modified as “In view of the following two factors: 1) the sex determination system of T. dichotomus is XY, in which the Y chromosome is much smaller than the X chromosome [30]; 2) there was only one pair of newly emerged beetles of different sexes obtained for genome sequencing; thus, to meet the sequencing requirements, muscle of a female thorax was prepared for Illumina and Nanopore sequencing, and then a male thorax was dissected for Hi-C and RNA sequencing (Table S1)” (lines 108-113).

(14) Line 113. 1) What is the methodology for these rearing medias? Is there control to make sure they have similar microbial contents despite their different composition? 2) Is the mushroom residue just pure fungus? Or does that include wood in the media that has been decomposed by mycelia? 3) More description of these media would greatly improve what conclusions could be made for these samples.
4) Additionally, would these beetles be found feeding solely in fungi in the wild? Or would they always be consuming a mix of wood and fungi (along with other decomposing matter)? Detailed description of the biology of this species' feeding would help for readers who are not familiar with this beetle in the wild (such as myself, as a North American).
Authors response:
1) Yes, all of the rearing medias were sterilized by high temperature steam.

Powered by Editorial Manager® and ProduXion Manager® from Aries Systems Corporation

2) No, it is not just fungus. The mushroom residue is composed of wood fiber and fungal mycelia.
3) What the reviewer suggested is meaningful. According to the above mentioned comments, the original description has been modified as "The similar-sized larvae were divided into two groups and reared with high-temperature sterilized sawdust and mushroom-residue (composed of wood fiber and fungal mycelia) at 20–25°C and 50–60% humidity for 2 months, separately" (lines 115-118).
4) Yes, the detailed description of the biology of this species' feeding is necessary for this manuscript. The feeding biology of Trypoxylus dichotomus in the wild is included in the third paragraph of "Introduction" and third paragraph of "3.6 Differentially expressed digestion-related genes", i.e., "In the larval stage, it can decompose recalcitrant wood material and humus efficiently in the wild [19-21], ……" (lines 70-71), and "In the forest, the larvae of T. dichotomus usually inhabit soil organic matter and feed on decayed wood [19, 20, 27]. This is similar to the living and feeding habitats of the white-spotted flower chafer, Protaetia brevitarsis (Scarabaeidae), which also efficiently digests high lignocellulosic mushroom-residue [112]. Interestingly, both species were often observed coexisting in the outdoor mushroom-residue, ……" (lines 563-568).

(15) Line 115/Figure 1. 1) While it's nice to see that the wood fiber was digested, what's the rationale for providing these images as in-text figures as opposed to supplementary figures (as they don't seem terribly important to me). 2) Also, what was the methodology of taking these photos? Is this wood fiber from the guts of the larvae, or extra-orally digested fiber? What type of image are these (I assume scanning electron microscopy)? 3) Were there strategies employed to make sure there was no bias in selecting what tissue was imaged? (i.e., are these images fully representative of the entire amount of diet tissue, and not just exemplars representing that some of the wood fiber was digested but not necessarily all of it?) 4) No details are provided.
Authors response:
1) Figure 1 shows the 3rd instar larva of Trypoxylus dichotomus (a), the wood fiber structures of sawdust before (b) and after (c) degradation, which could reveal the digestion ability of lignocellulose by T. dichotomus visually. Thus, the preliminary research result of figure 1 is important and meaningful for the following researches in this manuscript, and should be kept as in-text figure.
2) Figure 1a refers to the 3rd instar larva of T. dichotomus, which was taken by a digital single lens reflex camera. Figures 1b and 1c were taken by an environment scanning electron microscope (ESEM). The wood fiber before degradation (Figure 1b) was taken from sawdust, while the wood fiber after degradation was taken from the larval excrement of T. dichotomus after the digestion of sawdust.
3) Yes, figure 1c can represent the wood fiber structure of sawdust after digestion by the 3rd instar larvae of T. dichotomus. In this study, six larval excrements samples were randomly selected and photographed by ESEM, the wood fiber structures of sawdust were degraded into the similar fragments after digestion by the T. dichotomus larvae.
4) The original statement has been be supplemented with the above-mentioned information. The updated statement is "After intake and digestion by the 3rd instar larvae (Fig. 1a, taken by a digital single lens reflex camera), six larval excrement samples were randomly selected and photographed by an environment scanning electron microscope (ESEM). Compared to the intact wood fiber of sawdust before digestion (Fig. 1b, taken by ESEM), the wood fiber structures of sawdust were degraded into similar fragments after digestion by the larvae of T. dichotomus (Fig. 1c)." (lines 119-124).

(16) Line 127. What does "gene library construction" refer to?
Authors response: It refers to the short-insert (350 bp) and large-insert (>20 kb) libraries for genome survey and sequencing, respectively. Thus, the relevant information has been added in the original statement, i.e., "Genomic DNA was extracted using a QIAGEN® genomic kit for short-insert (350 bp) and large-insert (> 20 kb) library construction according to the manufacturer's instructions" (lines 136-138).

(17) Line 135. What was this "certain concentration and volume" used?
Authors response: The concentration and volume were 50 fmol and volume 24 μL, respectively. The original description has been amended as "After genome estimation, a certain concentration (50 fmol) and volume (24 μL) of DNA library ……" (lines 147-148).

Powered by Editorial Manager® and ProduXion Manager® from Aries Systems Corporation

(18) Line 155. What is meant by "double check"? Do you mean using NCBI's EGAP? Or just their standard quality control?
Authors response: After checking, we find out that the phrase “double check” is an inaccurate statement in the original description, so we have modified this sentence as “Scaffolds greater than 10 kb were retained and uploaded to NCBI for contamination detection in the final assembly” (lines 173-175).

(19) Line 150-163. I am unclear on what the authors mean by "NGS data" here. The genome assembly of the ONT data seem appropriate, but then some additional NGS data was aligned to the reference around like 158? I am not following these methods. I assume this is just the TruSeq libraries used for polishing the ONT assembly, but this is never specified (or is it just used for the kmer genome size estimation but not for anything else?).
Authors response:
1) In the line 150 of the first manuscript, the phrase “Next-Generation Sequencing (NGS) data” refers to the Illumina DNA data of genome survey, which was used for polishing the ONT assembly. The original statement is easy to be misunderstood, so the original description has been amended as “The Illumina DNA data of the genome survey were filtered using fastp with default parameters. The corrected genomic data were polished with the filtered DNA data of the genome survey using Nextpolish (v1.0.5) over four iterations” (lines 168-171). Similarly, the phrase “NGS DNA data” has been replaced by “Illumina DNA data of the genome survey” in the lines 178-179 or “Illumina DNA data from a genome survey” in the lines 319-320. While, the phrase “NGS RNA data” has been replaced by “Illumina RNA data” in the lines 328-329.
2) The original statement “As a reference genome, ……GC depth was analyzed using Minimap2 and Samtools.” is verified to be incorrect, which has been removed. The correct statement, i.e., “Moreover, to verify utilization of raw data and the completeness of genome assembly, the Illumina DNA data of the genome survey, Illumina RNA data of the male thorax, and ONT data were mapped to the genome assembly using Minimap2. Then, the mapping rates were calculated using SAMtools v1.9 [38]”, has been added in the revised manuscript (lines 177-181). Furthermore, the original corresponding results of this analysis “The mapping rates of NGS (GSS, RNA-seq) and ONT reads onto our draft genome were high as 99.89, 95.39 and 99.60%, respectively” has been modified as “The mapping rates of Illumina DNA data from the genome survey, Illumina RNA data of the male thorax and ONT data onto our draft genome were as high as 99.89, 95.39, and 99.60%, respectively” (lines 373-376).

(20) Line 173. How were the libraries quantified?
Authors response: The statement of the libraries quantification, i.e., “Libraries were quantified using Qubit 3.0 fluorometry (Invitrogen)”, has been added after the sentence “Genomic DNA was extracted using a QIAGEN® genomic kit for short-insert (350 bp) and large-insert (> 20 kb) library construction according to the manufacturer's instructions” (lines 136-138).

(21) Line 232. "unreliable" is not a term used by BMGE. What exact BMGE commands were used?
Authors response: Yes, the word “unreliable” is not enough to describe the concrete usage of the program BMGE. The detailed parameter “(-m BLOSUM90 -h 0.4)” has been added for this program, so the present description is “The unreliable homologous regions were removed with BMGE v1.12 (-m BLOSUM90 -h 0.4) [77]” (lines 255-256).

(22) Table 1. Should read "NextDenovo".
Authors response: Yes, it was misspelled. The proper name “NextDenovo” has replaced the incorrect one in the Table 1 (line 332).

(23) Line 387. What does "commendably recovered" mean?
Authors response: It means the phylogenetic relationships of these 14 insect species are same as the previous phylogenomic studies [83, 100] mentioned earlier in the manuscript. The original statement has been amended as “The phylogenetic relationships of 14 insect species were well recovered [83, 100], ……” (lines 426-427).

(24) Line 417. 1) This sentence does not make sense grammatically. 2) Additionally, based on the beetle genomes available, I would argue that this analysis doesn't

address how "most beetles" operate (since you only sample 9 beetle species).
Authors response:
1) The original sentence has been modified as " Most beetles were considered not to capitalize on their significant ability for endogenous lignocellulose digestion [101], but this is not the case for T. dichotomus " (lines 457-458).
2) This statement was cited from the 101th article in the references, i.e., "Calderón-Cortés N, Quesada M, Watanabe H, Cano-Camacho H and Oyama K. Endogenous plant cell wall digestion: a key mechanism in insect evolution. Annu Rev Ecol Evol Syst. 2012;43:45-71" (lines 918-920), which indicated that most beetles lack the capacity for significant endogenous lignocellulose digestion.

(25) Figure 6a. Which samples in the PCA are SM2 and SM6? Additionally, I am red-green colorblind and have a somewhat tough time seeing the different colors in this plot.
Authors response: The original colors "green" and "red" in "Figure 6a" have been replaced by "purple" and "blue", respectively. Furthermore, the symbols "SM1–6", "MM1–6", "MH1–6" and "SH1–6" have been marked in the updated figure "Figure 6a", separately.

(26) Line 480. 1) What is the rationale for this statement? I can see that perhaps there is more separation in ordination space of SM vs MM samples (excluding the outliers), but that separation is along PC2 (8%) compared to separation of arguably similar numbers of SH vs MH along PC1 (86%), so I would almost put more stock into slight differentiation along PC1 vs. broad differentiation along PC2. 2) Overall, I am not convinced by statements throughout the study about midgut having stronger digestion ability than hindgut and the comparisons of wood vs. fungi as food material.
Authors response:
1) The original statement is verified to be incorrect. The section 3.5 has been reworded according to the reviewer's comment. Furthermore, to show the significant differences among groups, the PERMANOVA analysis has been added in the revised manuscript. The updated content of "section 3.5" is as follows (lines 503-522):
To further explore the intestinal gene expression patterns associated with different gut tissues and food habits, we carried out intestinal transcriptome analysis for the larvae of T. dichotomus. Based on the gene expression (FPKM) of all annotated genes for each sample by PERMANOVA, we found significant differences of gene expressions between the groups separated by gut tissues or food habits (Table 4). PCA and PCC (Tables S13 and S14) were then used to calculate and plot diagrams (Fig. 6), respectively. With PCA analysis (Fig. 6a), we showed that samples from the same group were mainly aggregated together, except for four outliers (SM2, SM6, SH2 and SH3) in the midgut and hindgut of sawdust feeding beetles. Similarly, PCC analysis (Fig. 6b) also displayed good repeatability within most of the intra-groups, but a relatively low level of repeatability in the midgut of sawdust feeding larvae was due to the abnormal values of SM2.
For the groups with the same food habits (SM and SH, MM and MH), more significant differences of gene expressions were observed between the midgut and hindgut in the sawdust groups than in the mushroom-residue groups along PC1 and PC2. Furthermore, there were also significant differences between groups within the same gut tissue (SM vs MM, SH vs MH), suggesting that intestinal gene expressions could be significantly affected by food habits in T. dichotomus. Consistently, it was reported that different host diets could significantly affect the digestive physiology of the beetle, Trogoderma granarium [28].

2) The original statements about midgut having stronger digestion ability than hindgut and the comparisons of wood vs. fungi as food material are indeed unconvincing. The original description has been modified as "These results indicate that more digestion-related genes are highly expressed in the midgut than the hindgut of larvae, regardless of food habit. Thus, the digestion of lignocellulose in larvae may require more digestive enzymes in the midgut than in the hindgut" (lines 539-543), and "These results suggest that digestion of mushroom-residue might require a greater digestive ability than that of sawdust for the larvae of T. dichotomus, which is probably due to the complex components of mushroom-residue, including not only wood fiber but also fungal mycelia" (lines 555-559).

(27) Lines 507-511. I am not following the logic here 100%. Presumably mushroom

Powered by Editorial Manager® and ProduXion Manager® from Aries Systems Corporation

residue would be already broken down compared to wood fiber itself and require less digestion on the part of the beetle, but these results indicate more expression of digestion-related genes in the mushroom treatment. Perhaps the digestion of mushroom components themselves require the extra digestive ability, not just the lignocellulose?
Authors response: What the reviewer proposed is reasonable. The original statement has been modified as “Taken together, more digestion-related genes were highly expressed in the mushroom-residue group than in the sawdust group regardless of whether the specific location was the midgut or hindgut. These results suggest that digestion of mushroom-residue might require a greater digestive ability than that of sawdust for the larvae of T. dichotomus, which is probably due to the complex components of mushroom-residue, including not only wood fiber but also fungal mycelia” (lines 553-559).

(28) Lines 536-538. Not technically true since those are not sister taxa. Their ancestors diverged from each other at that time.
Authors response: Yes, the reviewer’s perspective is rational. The original statement has been modified as “……, showing that the ancestor of T. dichotomus diverged in the early Cretaceous (120 Mya) from that of the closely related species O. taurus” (lines 584-585). In addition, the other similar statements have been modified with the phrase “the ancestors of” added as well (lines 22 and 430).

Response to Reviewer 2:
Major Comments
(1) Title. The genome is estimated here as 599 MB, but the assembly is 636 MB. That needs to be resolved before claiming a chromosome level assembly.
Authors response: The consensus estimated genome size (599 Mb) was inferred from the genome estimation results of FindGSE (630.93 Mb) and GenomeScope (567.4 Mb) (line 323), which could not represent the actual genome size of this beetle. Furthermore, our final genome assembly size (636 Mb) was close to the previous estimated size by FindGSE, indicating that the final genome size was reliable. Not only that, there were 606.8 Mb scaffolds covering 95.37% of the draft reference genome anchored onto 10 pseudo-chromosomes in the chromosome-level assembly, which showed the chromosome-level genome assembly of high quality. As mentioned above, the tile “A chromosome-level genome assembly and intestinal transcriptome of Trypoxylus dichotomus (Coleoptera: Scarabaeidae) to understand its lignocellulose digestion ability” is applicable for this manuscript.

(2) Abstract. How is the timing of the split between Td and O taurus is needed in the abstract.
Authors response: The divergence time between the ancestors of Trypoxylus dichotomus and Onthophagus taurus was inferred to be 120 Mya. This information has been added in the revised abstract, i.e., “…… in the early Cretaceous (120 Mya)” (lines 23-24).

(3) L39. Beetles are not mentioned in the first paragraph. I realize that lignocellulose digestion might be an important aspect to this work, but the paper reports a beetle genome assembly. That should be the main focus. The start of the first paragraph is much better for a biological, not geological, audience.
Authors response: What the reviewer suggested is reasonable to some extent. However, the first paragraph highlighted the significance of this study, and explained why we paid great attention to the lignocellulose digestion related genes of this beetle, which is important for this manuscript. Thus, the main content of this paragraph is considered to be kept, but the overall content should be simplified and improved with the first three sentences removed. Given this, the original description at the beginning of first paragraph, i.e., “Plant biomass, mainly consisting of lignocellulose, …… But the improper handling of plant biomass is one of the most important factors accelerating global warming [6, 7]”, has been deleted. Thus, the updated paragraph starts with the content “As a key structural component of plant biomass and an important route of carbon fixation, ……” (line 42).

(4) L222 - I was going to put this in the minor comments, but then I realized that it is actually a serious comment. All of the used genomic resources need citations. I know

off the top of my head at least nine do. If we as a field do not cite reference for the resources that we use, how can we expect people in other fields to cite them. I assume the authors here want people to cite this work when using the resource here, so they should do the same for others.
Authors response: Yes, the reviewer's advice is reasonable. The citation of genomic resources of the other insects is necessary for this manuscript. Thus, the genomes of all the involved insects have been appended with the bibliographic citation (lines 247 and 250), and the corresponding references [64-74] have been added in the updated manuscript as well, i.e., "……, including eight beetles (Tri. castaneum, Agrilus planipennis, Lamprigera yunnana, Nicrophorus vespilloides, Onthophagus taurus, Aethina tumida, Sitophilus oryzae and Anoplophora glabripennis) [64-69] and five other insect species (Drosophila melanogaster (Diptera), Apis mellifera (Hymenoptera), Bombyx mori (Lepidoptera), Coptotermes formosanus (Blattodea) and Rhopalosiphum maidis (Hemiptera)) [70-74]" (lines 245-250).

(5) L127. I assume this was done according to the manufacturer's instructions, but state that so the reader does not have to wonder.
Authors response: Yes, this was done according to the manufacturer's instructions. The original description has been amended as "Genomic DNA was extracted using a QIAGEN® genomic kit for short-insert (350 bp) and large-insert (> 20 kb) library construction according to the manufacturer's instructions" (lines 136-138).

(6) L163. What any type of haplotype reduction used or tried?
Authors response: Haplotype reduction was not used or tried in this manuscript.

(7) L194. 1) HISAT2 - what paramters were used? 2) What any QC done of the RNA-seq reads or the alignments? Again, more details are needed in this section to make it reasonably reproducible.
Authors response:
1) The parameter "--dta" has been added after the software "HISAT2 v2.2.0" (line 218).
2) Quality control of RNA-seq reads and alignments was performed with the program bbduk.sh (qtrim=rl trimq=20 minlen=20 ecco=t maxns=5 trimpolya=10 trimpolyg=10 trimpolyc=10) in the BBTools v38.82. The details of quality control have been added in the following sentence, i.e., "To improve the prediction accuracy, transcripts of thoracic muscle were optimized in the program bbduk.sh (qtrim=rl trimq=20 minlen=20 ecco=t maxns=5 trimpolya=10 trimpolyg=10 trimpolyc=10) in BBTools v38.82 [48]" (lines 213-216).

(8) L200. What QC of the gene models from MAKER was done?
Authors response: Using MAKER, gene prediction was performed with three strategies, Ab initio, transcriptome and protein, respectively. In the integrated results of MAKER, the AED values (range from 0 and 1, with 0 denoting perfect agreement of the annotation) can be referenced to the quality of gene prediction. Furthermore, the parameters "min_protein=30, min_intron=20" were set for quality control (line 210).

(9) L209. Is eggNOG not a third method to assign gene functional information. How was the information QC and integrated?
Authors response: Gene function was annotated with two strategies: 1) Gene function was predicted by searching the specialized database "UniProtKB"; 2) Protein conserved sequences and domains, Gene Ontology (GO), and pathways [Kyoto Encyclopedia of Genes and Genomes (KEGG), Reactome] were predicted by searching the integrated databases "InterProScan and eggNOG". Although InterProScan and eggNOG are both integrated databases, they are usually different in KEGG pathways and GO terms. Thus, it's necessary to search these two databases at the same time. Applying these two strategies, we could get as much information of gene function annotation as possible. Furthermore, quality control are not needed for the above gene function information. The results of above two strategies were finally presented in the 3rd paragraph of section 3.3 (lines 394-400).

(10) L262. That is definitely not the proper citation for the Tc genome. That paper does not report a single nucleotide sequence.
Authors response: Yes, the original citation for the genome of Tribolium castaneum is improper. Thus, it has been replaced by the proper reference [64] in the updated manuscript, i.e., "Richards S, Gibbs RA, Weinstock GM, Brown SJ, Denell R, Beeman

	RW, et al. The genome of the model beetle and pest Tribolium castaneum. Nature. 2008;452 7190:949-55. doi:10.1038/natur e06784" (lines 817-819).<br><br>(11) L298. How does this suggest good or bad sample quality? Heterozygosity is a biological property, as is genome size.<br>Authors response: Yes, heterozygosity is a biological property for the genome of Trypoxylus dichotomus. The incorrect statement has been corrected with the original phrase "suggesting the good quality of the test sample" removed (line 326).<br><br>(12) L298. Sequencing results need to be separated from genome size estimate results.<br>Authors response: The sequencing results have been separated from genome size estimate results, and placed in the next paragraph (lines 327-330).<br><br>(13) L321. But 599 Mb was the consensus estimate you gave.<br>Authors response: Despite using the same Illumina DNA data of the genome survey, the results of genome estimation were different between the methods of FindGSE (630.93 Mb) and GenomeScope (567.4 Mb). For that reason, we provided the consensus estimated genome size (599 Mb) before Oxford Nanopore Technologies (ONT) sequencing. However, our final genome assembly size was 636 Mb after analyzing ONT sequencing data, which was apparently close to the genome estimation by FindGSE. The original statement might be inappropriate, so we deleted the word "pretty" in the revised manuscript, and the revised sentence is "……, which was close to the earlier genome estimation by FindGSE" (line 351).<br><br>(14) L356. 4% of the BUSCO genes are not represented in the gene set? What number is duplicated or fragmented? All of those should have been in each proteome used to annotate. Odd to lose so much in what seems such a complete assembly.<br>Authors response: The fragmented genes account for 1% among all the genes in the BUSCO assessment, thus there were actually 96.8% genes predicted in the MAKER process.<br>The software MAKER predicted genes based on the pathway of "Ab initio", with another two pathways "transcriptome" and "protein" for the correction. Thus, the insufficient transcriptomic information might lead to the weak support for predicted genes. Furthermore, the larger size the genome, the worse the gene prediction in MAKER in general. Actually, this BUSCO completeness of gene prediction (95.8%) is relatively high among the other published insect genomes.<br><br>(15) L359. How many of the hits were to other scarabs? To other beetles? To other insects? The reader needs more information here better assess the quality of the annotation.<br>Authors response: The table 2 "Gene hits between Trypoxylus dichotomus and another six insects" has been added in the revised manuscript, which shows the gene hit number to the other involved insects (line 410). Furthermore, the description of gene hits have also been added in the end of third paragraph of "Genome annotation", i.e., "To evaluate these datasets, we compared them with other high-quality genome annotations from six insects and revealed more than 10,000 hits (Table 2)" (lines 398-400).<br><br>(16) L377. How many beetle only orthogroups were there?<br>Authors response: There are 12,658 orthogroups only to beetle. The original description has been amended as "Using homology analysis of the gene family, 181,904 (92.90%) genes were clustered into 14,467 orthogroups (gene families), in which 12,658 orthogroups were unique to beetles. Moreover, there were 1,260 single-copy orthogroups and 3,120 multi-copy orthogroups identified for T. dichotomus" (lines 414-417).<br><br>(17) L413. Why is immunity being seen with the GO analysis for the gene families important? What were the contracted families and their enriched GO terms, if any?<br>Authors response: The reviewer's comment reminds us that the immunity is not important to the manuscript. Although there are two gene families involved in immunity (Galectin and Serine protease Hayan) ranked in the first 20 rapidly expanded gene families, this manuscript mainly focuses on the digestion of Trypoxylus dichotomus larvae. Thus, the discussion of immunity has been removed in the revised manuscript.

Powered by Editorial Manager® and ProduXion Manager® from Aries Systems Corporation

The original statement "……; meanwhile gene family concerning immunity was also affected by positive selection during evolution" has been deleted (lines 545-456).
In addition, GO and KEGG enrichments has been appended as supplementary files (Tables S9 and S10) in the revised manuscript (line 447), which provides the detailed information for enriched gene families. The updated statement is "The rapidly expanded gene families were further confirmed in the GO and KEGG enrichments (Tables S9 and S10), with metabolic detoxification, digestion and immunity mainly in the GO enrichment (Fig. 5b), and metabolic detoxification, digestion, juvenile hormone and secondary metabolite synthesis mainly in the KEGG pathway (Fig. 5c)" (lines 446-450).

(18) L466. I fundamentally disagree with that statement. There a large scatter in hind and mid-gut samples along the two axes. Hind and mid definitely separate, but little consistent pattern after that.
Authors response: Yes, the original statement failed to clarify the result of PCA analysis. The original sentence has been modified as "With PCA analysis (Fig. 6a), we showed that samples from the same group were mainly aggregated together, except for four outliers (SM2, SM6, SH2 and SH3) in the midgut and hindgut of sawdust feeding beetles" (lines 509-511). Furthermore, the permutational multivariate analysis of variance (PERMANOVA) is newly added in the revised manuscript. The result of PERMANOVA indicates that there are significant differences among the pairwise comparisons of groups separated by gut tissues and food habits (SM vs SH, MM vs MH, SM vs MM and SH vs MH) (Table 4). This information has been added in the second paragraph of section "2.5 Intestinal transcriptome analysis" and the first paragraph of section "3.5 Gene expression and sample correlation", i.e., "Based on FPKM, permutational multivariate analysis of variance (PERMANOVA) was performed with 999 permutations using the R package 'vegan' [93]" (line 301-302) and "Based on the gene expression (FPKM) of all annotated genes for each sample by PERMANOVA, we found significant differences of gene expressions between the groups separated by gut tissues or food habits (Table 4)" (lines 505-507).

(19) Section 3.6. I have great difficulty understanding this section. 1) What was the comparison treatment? 2) So there are 423 DEGs in total? That seems like very few genes given the complete shift of diet, esp from native to a manipulated diet. That from two separate tissues too. 3) These sections are very, very difficult to get through and interpret.
Authors response:
1) The comparison treatment refers to midgut and hindgut treatments from the same food habit (SM vs SH and MM vs MH), and sawdust and mushroom-residue treatments from the same gut tissue (SM vs MM and SH vs MH) which are listed in the "Table S16". To clarify the comparison treatment, the detailed information has been added in the revised manuscript. The updated statement is "To understand the digestive ability of T. dichotomus larvae on different gut tissues and food habits, digestion-related genes were filtered (Table S15) and differentially expressed genes were further compared within four different treatment groups, i.e., SM vs SH, MM vs MH, SM vs MM and SH vs MH (Table S16, Fig. 7)" (lines 530-533). In which, the specific group information is mentioned earlier in the first paragraph of "2.1 Sampling and sequencing", i.e., "All of the samples were divided into four groups: 1) midgut from sawdust (SM, midgut of larva feeding sawdust), 2) hindgut from sawdust (SH, hindgut of larva feeding sawdust), 3) midgut from mushroom-residue (MM, midgut of larva feeding mushroom-residue), 4) hindgut from mushroom-residue (MH, hindgut of larva feeding mushroom-residue). Each group consisted of six replicates" (lines 130-135).
2) The differentially expressed genes (DEGs) are not 423 in total. As mentioned in this manuscript, there are 222, 231, 92 and 83 DEGs in the groups of "SM vs SH", "MM vs MH", "SM vs MM" and "SH vs MH", respectively. Mushroom-residue usually contains fungal mycelia, but it is mainly composed of partially decayed wood. Thus, it shares a high proportion of similar component with sawdust, which makes the differences between these two rearing foods are not as big as they look. For this reason, there were not too many DEGs between the groups of different rearing foods from the same gut tissue.
3) The original statement did have some inaccuracies, which makes certain content in the above-mentioned section confusing. According to the first reviewer's comments, these problems and mistakes have been corrected, with the statements being improved in the revised manuscript (see the detailed responses to the comments 2, 26

and 27 of Reviewer 1).

(20) L500. 1) Much of this is discussion, not results. 2) However, DEGs being different between sawdust and mushrooms is not direct evidence the beetles are better at digesting mushrooms than sawdust.
Authors response: This comment is considered to be part of the former one.
1) This paragraph is in the third part “Results and Discussion”, the contents of results are closely followed by the contents of discussion, so it’s not a problem for this manuscript (lines 546-559).
2) Yes, the differences of DEGs between sawdust and mushrooms is not a direct evidence that Trypoxylus dichotomus is better at digesting mushrooms than sawdust. As mentioned in the above comment, we have modified the original statement as “These results suggest that digestion of mushroom-residue might require a greater digestive ability than that of sawdust for the larvae of T. dichotomus, which is probably due to the complex components of mushroom-residue, including not only wood fiber but also fungal mycelia” (line 555-559).

(21) L512-522. This is not a result.
Authors response: Yes, this is a discussion. The results are closely followed by the content of discussion in the “Results and Discussion” section (lines 560-570).

Minor Comments
(22) L25. There is no evidence provided that supports statement directly. I think it is likely, but the language needs to be moderated.
Authors response: Yes, the original statement is not proper. Thus, it has been modified as “Through gene family evolution analysis, we found 67 rapidly evolving gene families, within which there were two digestive gene families (encoding Trypsin and Enoyl-(Acyl carrier protein) reductase) that have experienced significant expansion, indicating that they may contribute to the high degradation efficiency of lignocellulose in T. dichotomus” (lines 24-28).

(23) L29. Need a comma before respectively.
Authors response: The comma has been added before “respectively”, i.e., “…… being intersected with chromosomes 2 and 10 of Tribolium castaneum, respectively” (line 31).

(24) Keywords. Put in alphabetical order.
Authors response: These keywords have been rearranged in alphabetical order, i.e., “Chromosome rearrangement, gene family, intestinal transcriptome, lignocellulose digestion, rhinoceros beetle” (lines 38-39).

(25) L63. "receiving preeminent research achievements" is very odd phrasing.
Authors response: Yes, the original statement is improper. The original sentence has been amended as “Among them, xylophagous termites are the most well-known of efficient lignocellulose digesters, having been studied in detail including in terms of functional genomics and symbiotic intestinal microorganisms [14-16]” (lines 59-62).

(26) L73. Can, not could. It still has this ability as a species.
Authors response: Yes, it refers to the larval ability of Trypoxylus dichotomus. The word “could” has been replaced by “can” (line 70).

(27) L113. Where did the sawdust and mushrooms come from? What was the temperature? Far more detail is needed here for repeatability.
Authors response: Yes, the rearing details of T. dichotomus larvae are needed for repeatability. The original description has been added with the information on larvae rearing, and thus modified as "The similar-sized larvae were divided into two groups and reared with high-temperature sterilized sawdust and mushroom-residue (composed of wood fiber and fungal mycelia) at 20–25°C and 50–60% humidity for 2 months, separately" (lines 115-118).

(28) L114. Is this needed? Was this in doubt?
Authors response: Yes, the figure 1 reflects the wood fiber structure after digestion by this beetle, which provides the visual evidence for its digestion ability of lignocellulosic biomass. To make the statement more accurate and detailed, the original statement

	has been amended as “After intake and digestion by the 3rd instar larvae (Fig. 1a, taken by a digital single lens reflex camera), six larval excrement samples were randomly selected and photographed by an environment scanning electron microscope (ESEM). Compared to the intact wood fiber of sawdust before digestion (Fig. 1b, taken by ESEM), the wood fiber structures of sawdust were degraded into similar fragments after digestion by the larvae of T. dichotomus (Fig. 1c)” (lines 119-124).<br><br>(29) L130. What quality filer was used? What program? What parameters? Or were these just the QC of the Illumina machine. What machine was used?<br>Authors response: Quality control of raw reads was performed using fastp (v.0.20.0) preprocessor. The detailed description of quality control has been added in the revised manuscript, i.e., “The raw reads were filtered using the fastp (v.0.20.0) preprocessor [31] (set to default parameters) to remove low quality reads, adapters, and reads containing poly-N” (lines 141-143).<br><br>(30) L136. What chemistry and chip?<br>Authors response: The chemistry and chip of PromethION is “FLO-PRO002”, which has been added in the sentence “After genome estimation, a certain concentration (50 fmol) and volume (24 μL) of DNA library was transferred to a flow cell of PromethION (ONT, Oxford Nanopore Technologies, FLO-PRO002 chip) for whole genome sequencing” (lines 147-150).<br><br>(31) L137-142. 1) How were the sample homogenized? 2) What version of the kits for TruSeq? 3) What version of chemisty for the Novaseq? 4) Where? What was the target insert size? 5) How did you QC the RNA and the gDNA? Many needed details are missing to make the work reasonably reproducible.<br>Nothing is mentioned out the Hi-C sequencing mentioned in the previous paragraph.<br>Authors response:<br>1) Total RNA was extracted from the male thoracic muscle, “……, and then a male thorax was dissected for Hi-C and RNA sequencing (Table S1)” (line 113), and then qualified and quantified using NanoDrop 2000 and Agilent 2100 system. The sentence “It was qualified and quantified as follows: 1) RNA purity and concentration were examined using NanoDrop 2000; 2) RNA integrity and quantity were measured using the Agilent 2100 system” has been added in the revised manuscript (lines 152-154).<br>2) The version of the kits for TruSeq is TruSeq RNA Library Preparation Kit v2 (line ...
**Additional Information:**	
**Question**	**Response**
Are you submitting this manuscript to a special series or article collection?	No
**Experimental design and statistics**<br><br>Full details of the experimental design and statistical methods used should be given in the Methods section, as detailed in our Minimum Standards Reporting Checklist. Information essential to interpreting the data presented should be made available in the figure legends.<br><br>Have you included all the information requested in your manuscript?	Yes
**Resources**	Yes

Powered by Editorial Manager® and ProduXion Manager® from Aries Systems Corporation

A description of all resources used, including antibodies, cell lines, animals and software tools, with enough information to allow them to be uniquely identified, should be included in the Methods section. Authors are strongly encouraged to cite Research Resource Identifiers (RRIDs) for antibodies, model organisms and tools, where possible.<br><br>Have you included the information requested as detailed in our Minimum Standards Reporting Checklist?	
**Availability of data and materials**<br><br>All datasets and code on which the conclusions of the paper rely must be either included in your submission or deposited in publicly available repositories (where available and ethically appropriate), referencing such data using a unique identifier in the references and in the "Availability of Data and Materials" section of your manuscript.<br><br>Have you have met the above requirement as detailed in our Minimum Standards Reporting Checklist?	Yes

Powered by Editorial Manager® and ProduXion Manager® from Aries Systems Corporation

Manuscript
Click here to access/download;Manuscript;Chromosome-level genome ofTrypoxylus dichotomus.docx

A chromosome-level genome assembly and intestinal transcriptome of *Trypoxylus dichotomus* (Coleoptera: Scarabaeidae) to understand its lignocellulose digestion ability

Qingyun Wang[a], Liwei Liu[a,b], Sujiong Zhang[c], Hong Wu[a], Junhao Huang[a*]
[a] National Joint Local Engineering Laboratory for High-Efficient Preparation of Biopesticide, Zhejiang A&F University, 666 Wusu Street, Lin'an, Hangzhou, Zhejiang 311300, China
[b] Zhejiang Museum of Natural History, No.6 West Lake Cultural Square, Hangzhou, Zhejiang 310014, China
[c] Dapanshan Insect Institute of Zhejiang, Pan'an, Zhejiang, China
* Corresponding author: E-mail: huangjh@zafu.edu.cn, Tel: 86-571-63732758, Fax: 86-571-63740898

ORCID iDs:
Qingyun Wang [0000-0001-8756-4383]; Liwei Liu [0000-0002-5758-0638]; Sujiong Zhang [0000-0002-0809-6727];Hong Wu [0000-0001-5542-5370]; Junhao Huang [0000-0002-5038-3535].

Abstract

Lignocellulose, as the key structural component of plant biomass, is a recalcitrant structure, difficult to degrade. The traditional management of plant waste including landfill and incineration usually causes serious environmental pollution and health problems. Interestingly, the xylophagous beetle, *Trypoxylus dichotomus* can decompose lignocellulosic biomass. However, the genomics around the digestion mechanism of this beetle remain to be elucidated. Here, we assembled the genome of *T. dichotomus*, showing that the draft genome size of *T. dichotomus* is 636.27 Mb, with 95.37% scaffolds anchored onto 10 chromosomes. Phylogenetic results indicated that a divergent evolution between the ancestors of *T. dichotomus* and the closely related scarabaeid species *Onthophagus taurus* occurred in the early Cretaceous (120 Mya). Through gene family evolution analysis, we found 67 rapidly evolving gene families, within which there were two digestive gene families (encoding

Trypsin and Enoyl-(Acyl carrier protein) reductase) that have experienced significant expansion, indicating that they may contribute to the high degradation efficiency of lignocellulose in *T. dichotomus*. Additionally, events of chromosome breakage and rearrangement were observed by synteny analysis during the evolution of *T. dichotomus* due to chromosomes 6 and 8 of *T. dichotomus* being intersected with chromosomes 2 and 10 of *Tribolium castaneum*, respectively. Furthermore, the comparative transcriptome analyses of larval guts showed that the digestion-related genes were more commonly expressed in the midgut or mushroom-residue group than the hindgut or sawdust group. This study reports the well-assembled and annotated genome of *T. dichotomus*, providing genomic and transcriptomic bases for further understanding the functional and evolutionary mechanisms of lignocellulose digestion in *T. dichotomus*.

Keywords: Chromosome rearrangement, gene family, intestinal transcriptome, lignocellulose digestion, rhinoceros beetle

1. Introduction

As a key structural component of plant biomass and an important route of carbon fixation, lignocellulosic biomass is found in all kinds of living and dead plants. This biomass is principally composed of celluloses, hemicelluloses, pectins, and lignins [1], which form a complex cross-linked and recalcitrant structure that protects carbohydrates from decomposition by microorganisms or enzymes [2, 3]. The

traditional management of plant waste is usually done via landfill or incineration, which causes serious environmental pollution and health problems [4]. Thus, recycling plant wastes produced by human production is a noteworthy environmental issue [5]. Currently, chemical and biological pretreatment of lignocellulose degradation—especially biotransformation, an environmental-friendly and sustainable strategy for biofuels and biomaterial production—has catalyzed a great interest [6-8].

Due to the complex structural and chemical mechanisms of lignocellulose, lignocellulose decomposition is not common among animals [9, 10] except for wood-feeding insects such as termites, wood-feeding cockroaches, beetles, and wood wasps [7, 8, 10-12]. These insects are involved in the degradation of lignocellulose and other types of biomass by consuming plant cell walls, thereby contributing to lignocellulose bioconversion and energy utilization [13]. Among them, xylophagous termites are the most well-known of efficient lignocellulose digesters, having been studied in detail including in terms of functional genomics and symbiotic intestinal microorganisms [14-16]. Many studies have focused on the chemical degradation and microbiological deterioration of lignocellulose [17], however limited attention has been paid to the bio-degradation ability and genetic traits of other xylophagous insects, including the well-known ornamental scarabaeid beetle, *Trypoxylus dichotomus* (Linnaeus, 1771; NCBI:txid273928), which has a similar diet to xylophagous termites.

The rhinoceros beetle, *T. dichotomus* (Coleoptera: Scarabaeidae), is an ecologically important xylophagous and saprophagous insect widely distributed in China and neighboring countries [18]. In the larval stage, it can decompose recalcitrant wood material and humus efficiently in the wild [19-21], and this has been harnessed industrially to bio transform the waste substrate from mushroom production [22]. *T. dichotomus* can secrete various digestive enzymes comprising cellulase, glycanase, and glycosidase to degrade lignocellulose-rich plant polymers [19], greatly promoting the formation of soil organic matter, the major pool of organic carbons [23, 24]. To date, several studies have focused on the digestive enzymes and mutualistic associations with microbial symbionts in larval guts [19, 21, 25-27]. However, without the genome data of *T. dichotomus*, the underlying mechanisms enabling the digestion of lignocellulose will not be revealed.

It is generally believed that different diets significantly effect the digestive enzymatic activities of beetles [28]. Regional differentiation of the digestive tract and adaptations to divergent feeding habits mediate efficient digestion of food and protect insects against hazardous substances therein [29]. Although larvae of the rhinoceros beetle could degrade decaying wood and mushroom-residue efficiently, no work has studied their digestive ability in terms of different food eating habits. To understand the gut segment-specific function and molecular pattern of the digestive tract in larval *T. dichotomus*, it is necessary to identify the digestion-related genes and characterize their expression patterns associated with different food eating habits.

In the current study, we drafted the genome sequence of *T. dichotomus* and investigated its genomic characteristics through comparative genomic analysis with available datasets from other related insects. We also clarified the evolutionary history of gene families, highlighting the rapid expansion of two digestion related gene families and possible chromosome evolution events in *T. dichotomus*. Furthermore, we conducted an intestinal transcriptome comparative analysis of the 3rd instar larvae feeding on sawdust or mushroom-residue, and reveal that the expression of digestive enzyme genes was significantly higher in the midgut or mushroom-residue group than in the hindgut or sawdust group. Finally, we illustrate the effects of different food habits on *T. dichotomus* larval intestinal segments and digestive ability.

2. Materials and methods

2.1 Sampling and sequencing

The male and female adult samples and living larvae of *T. dichotomus* were obtained from the artificial breeding base in Pan'an County (28.94°N, 120.55°E), Zhejiang Province, China, in May 2020 and transported to the laboratory. The adult samples were washed three times with distilled water and then transferred to a clean bench for dissection. In view of the following two factors: 1) the sex determination system of *T. dichotomus* is XY, in which the Y chromosome is much smaller than the X

chromosome [30]; 2) there was only one pair of newly emerged beetles of different sexes obtained for genome sequencing; thus, to meet the sequencing requirements, muscle of a female thorax was prepared for Illumina and Nanopore sequencing, and then a male thorax was dissected for Hi-C and RNA sequencing (Table S1). Prior to the extraction of genomic DNA and RNA, the samples were transferred to liquid nitrogen for preservation. The similar-sized larvae were divided into two groups and reared with high-temperature sterilized sawdust and mushroom-residue (composed of wood fiber and fungal mycelia) at 20–25°C and 50–60% humidity for 2 months, separately.

After intake and digestion by the 3rd instar larvae (Fig. 1a, taken by a digital single lens reflex camera), six larval excrement samples were randomly selected and photographed by an environment scanning electron microscope (ESEM). Compared to the intact wood fiber of sawdust before digestion (Fig. 1b, taken by ESEM), the wood fiber structures of sawdust were degraded into similar fragments after digestion by the larvae of *T. dichotomus* (Fig. 1c). Then, six 3rd instar individuals were selected from each group, and quickly rinsed twice using 75% alcohol. Because the foregut is small and short with weak digestion, and digestive activity mainly occurs in the midgut and hindgut [19], only the midgut and hindgut were separated and quickly rinsed twice, with diethyl pyrocarbonate (DEPC) and then phosphate-buffered saline (PBS, 1X) on a clean bench for dissection. After drying the surface liquid, 24 midgut and hindgut samples were preserved in liquid nitrogen, separately (Table S2). All of the

samples were divided into four groups: 1) midgut from sawdust (SM, midgut of larva feeding sawdust), 2) hindgut from sawdust (SH, hindgut of larva feeding sawdust), 3) midgut from mushroom-residue (MM, midgut of larva feeding mushroom-residue), 4) hindgut from mushroom-residue (MH, hindgut of larva feeding mushroom-residue). Each group consisted of six replicates.

Genomic DNA was extracted using a QIAGEN® genomic kit for short-insert (350 bp) and large-insert (> 20 kb) library construction according to the manufacturer's instructions. Libraries were quantified using Qubit 3.0 fluorometry (Invitrogen). Prior to genome sequencing, a *k*-mer distribution analysis was performed using genome survey sequences (GSS, Illumina DNA data) to estimate genome size and heterozygosity. The raw reads were filtered using the fastp (v.0.20.0) preprocessor [31] (set to default parameters) to remove low quality reads, adapters, and reads containing poly-N. Briefly, quality-filtered reads were subjected to 17-mer frequency distribution analysis using the Jellyfish program [32]. By analyzing the 17-mer depth distribution from the 350-bp library cleaned sequencing reads, genome size and heterozygosity were estimated with FindGSE (skew normal distribution model) [33] and GenomeScope (negative binomial model) [34], separately. After genome estimation, a certain concentration (50 fmol) and volume (24 µL) of DNA library was transferred to a flow cell of PromethION (ONT, Oxford Nanopore Technologies, FLO-PRO002 chip; RRID:SCR_017987) for whole genome sequencing. Total RNA was extracted using QIAGEN® RNeasy Plus Universal Mini Kit, and ribosomal RNA

(rRNA) was removed with QIAseq FastSelect RNA Remove Kits. It was qualified and quantified as follows: 1) RNA purity and concentration were examined using NanoDrop 2000; 2) RNA integrity and quantity were measured using the Agilent 2100 system. Sequencing libraries were generated using TruSeq RNA Library Preparation Kit v2 (Illumina, USA) following the manufacturer’s recommendations. The library preparations (350 bp target insert size) were sequenced on an Illumina Novaseq 6000 platform (Illumina; RRID:SCR_016387) to generate 150 bp paired-end reads, according to the manufacturer’s instructions. All of the raw reads containing adapters and low quality bases (“N” >10%, Q-value≤20) were removed using fastp (fastp, RRID:SCR_016962).

2.2 Genome assembly

The quality of reads was controlled using ONT Guppy (v3.2.2) referring to the value of mean_qscore_template ≥ 7, with the other parameters left at the defaults. Passed reads were assembled with NextDenovo (v2.0) (reads_cutoff: 1 k, seed_cutoff: 23 k). Raw data were aligned with the assembled genome using Minimap2 [35] (-x map-ont; RRID:SCR_018550) for sequence alignment information. Based on this information, the genome was corrected using Racon (v1.3.1; RRID:SCR_017642) in three iterations. The Illumina DNA data of the genome survey were filtered using fastp with default parameters. The corrected genomic data were polished with the filtered DNA data of the genome survey using Nextpolish (v1.0.5) over four iterations.

Possible contaminated sequences were detected using BLAST+ v2.9.1 [36]

against the nt and UniVec databases, and then removed. Scaffolds greater than 10 kb were retained and uploaded to NCBI for contamination detection in the final assembly. Comparing to the insecta_odb10 database in OrthoDB, a benchmarking universal single-copy orthologs (BUSCO, RRID:SCR_015008) analysis was performed to assess completeness of genome assembly using BUSCO v5.1.2 [37]. Moreover, to verify utilization of raw data and the completeness of genome assembly, the Illumina DNA data of the genome survey, Illumina RNA data of the male thorax, and ONT data were mapped to the genome assembly using Minimap2. Then, the mapping rates were calculated using SAMtools v1.9 (SAMTOOLS, RRID:SCR_002105) [38].

To anchor hybrid scaffolds onto the chromosome, genomic DNA was extracted from the thoracic muscle of the male individual. The Hi-C library was prepared followed by a procedure [39] with improvement modifications. In brief, quick-freezing tissues of *T. dichotomus* were vacuum infiltrated in nuclei isolation buffer supplemented with 2% formaldehyde. Crosslinking was stopped by adding glycine and additional vacuum infiltration. Fixed tissue was then grounded into powders before re-suspending in nuclei isolation buffer to obtain a suspension of nuclei. The purified nuclei were digested with 100 units of DpnII and marked by being incubated with biotin-14-dCTP. Biotin-14-dCTP from non-ligated DNA ends was removed owing to the exonuclease activity of T4 DNA polymerase. The ligated DNA was sheared into 300−600 bp fragments, and then blunt-end repaired and A-tailed, followed by

purification through biotin-streptavidin-mediated pull down. Finally, the Hi-C libraries were quantified and sequenced using the Illumina Novaseq platform according to the manufacturer's instructions. Quality control of Hi-C raw data and extraction of Hi-C contacts was performed using Juicer v1.6.2 (Juicer, RRID:SCR_017226) [40]. Hi-C contigs were anchored to pseu-chromosomes using two rounds of 3D-DNA v180922 [41] workflow. The initial assignment was manually corrected using Juicebox v1.11.08 [40], and then imported into 3D-DNA again to produce the final chromosome-anchored genome assembly, with the contigs separated by 100 Ns on the same chromosome.

2.3 Genome annotation

A de novo repeat library was constructed using RepeatModeler v2.0.1 (RepeatModeler, RRID:SCR_015027) with a long terminal repeat (LTR) structural search [42], and then combined with the databases of Dfam_3.1 and RepBase-20181026 to generate a custom library. Repetitive elements (DNA/short interspersed nuclear element (SINE)/long interspersed nuclear element (LINE)/LTR) were searched by applying the program RepeatMasker v4.1.0 (RepeatMasker, RRID:SCR_012954) [43] based on the database of repeated sequences.

Protein-coding gene (PCG) structure was predicted in the pipeline of MAKER v3.01.03 (min_protein=30, min_intron=20) [44]. Three strategies were integrated for the prediction. 1) *Ab initio* gene structure prediction was made by applying the

BRAKER v2.1.5 pipeline (BRAKER, RRID:SCR_018964) [45] together with self-training of Augustus v3.3.4 (Augustus, RRID:SCR_008417) [46] and GeneMark-ES/ET/EP 4.59_lic [47]. To improve the prediction accuracy, transcripts of thoracic muscle were optimized in the program bbduk.sh (qtrim=rl trimq=20 minlen=20 ecco=t maxns=5 trimpolya=10 trimpolyg=10 trimpolyc=10) in BBTools v38.82 [48]. Then, they were incorporated with protein homology-based evidence, in which transcriptome evidence in BAM alignments was produced using HISAT2 v2.2.0 (--dta) (HISAT2, RRID:SCR_015530) [49]. The arthropod protein source was mined from the OrthoDB10 v1 database [50]. 2) With the BAM alignments inputted, transcripts of thoracic muscle were assembled using the genome-guided assembler StringTie v2.1.4 (StringTie, RRID:SCR_016323) [51]. 3) Protein sequences for *Drosophila melanogaster* (Diptera), *Apis mellifera* (Hymenoptera), *Bombyx mori* (Lepidoptera), and beetles (*Tribolium castaneum*, *Onthophagus taurus*, *Anoplophora glabripennis*) were downloaded from NCBI and passed to MAKER as evidence of protein homology. The prepared files obtained from the above pipeline were imported into MAKER for integrated annotation.

Gene function was annotated with the following two strategies. 1) Gene functions were annotated by searching the protein sequence database UniProtKB using Diamond v0.9.24 (--more-sensitive -e 1e-5) [52]. 2) protein conserved sequences and domains, gene ontology (GO), and pathways [Kyoto Encyclopedia of Genes and Genomes (KEGG), Reactome] were predicted by searching Pfam (Pfam,

RRID:SCR_004726) [53], SMART (SMART, RRID:SCR_005026) [54], Gene3D [55], Superfamily [56], and CDD [57] using InterProScan 5.41-78.0 (InterProScan, RRID:SCR_005829) [58]. Simultaneously, their functions were predicted by searching the eggNOG v5.0 database [59] employing eggNOG-mapper v2.0.1 [60].

Noncoding RNAs (ncRNAs) were annotated with two strategies. 1) Ribosomal RNAs (rRNAs), small nuclear RNAs (snRNAs), and micro RNAs (miRNAs) were searched against the Rfam database using the program infernal v1.1.3 [61]. 2) tRNAs were predicted using tRNAscan-SE v2.0.6 (tRNAscan-SE, RRID:SCR_010835) [62], with low-credibility tRNAs filtered out using the script 'EukHighConfidenceFilter'. Based on the results of genome annotation, chromosome length, GC-content, the density of PCGs and repetitive elements on each pseudo-chromosome were plotted and visualized using Circos (v0.67–7; RRID:SCR_011798) [63].

2.4 Comparative genomic and phylogenetic analysis

Gene family homology was inferred from protein sequences of 13 representative insect species downloaded from NCBI, including eight beetles (*Tri. castaneum*, *Agrilus planipennis*, *Lamprigera yunnana*, *Nicrophorus vespilloides*, *Onthophagus taurus*, *Aethina tumida*, *Sitophilus oryzae* and *Anoplophora glabripennis*) [64-69] and five other insect species (*Drosophila melanogaster* (Diptera), *Apis mellifera* (Hymenoptera), *Bombyx mori* (Lepidoptera), *Coptotermes formosanus* (Blattodea) and *Rhopalosiphum maidis* (Hemiptera)) [70-74]. Gene families were identified by

clustering protein sequences using OrthoFinder v2.3.8 (OrthoFinder, RRID:SCR_017118) [75] with Diamond [52] as the sequence aligner.

Phylogenetic trees were constructed with protein sequences of 1,260 single-copy orthologs, which were aligned with MAFFT v7.394 (MAFFT, RRID:SCR_011811) using the model 'L-INS-I' [76]. The unreliable homologous regions were removed with BMGE v1.12 (-m BLOSUM90 -h 0.4) [77]. All of the well-aligned sequences were concatenated with FASconCAT-G v1.04 [78]. Maximum likelihood (ML) trees were constructed using IQ-TREE v2.0.7 [79] with the set of '--symtest-remove-bad --symtest-pval 0.10' for removing those genes not conforming to SRH (stationary, reversible, and homogeneous). The substitution model was constrained to LG with a heuristic partitioned search strategy '-m MFP --mset LG --msub nuclear --rclusterf 10', and node support values were evaluated with ultrafast bootstrapping and SH-aLRT algorithms (-B 1000 --alrt 1000). The divergence time of phylogenies was estimated using r8s v1.81 [80]. Fossil calibration data were obtained from the PBDB database [81] and two published studies [82, 83], namely root (Pterygota, <443.4 Mya), Holometabola (315.2–382.7 Mya), Lepidoptera+Diptera (Trichoptera, 311.4–323.2 Mya), Coleoptera (307–323.2 Mya), Scarabaeiformia (196.5–201.3 Mya), Elateriformia (242–252 Mya) and Cucujiformia (196.5–201.3 Mya).

Expansions and contractions of gene families at each node of the evolutionary tree were estimated using CAFÉ v4.2.1 [84] under the stochastic gene birth-death

model and default significance level (p=0.01). For significantly expanded gene families, GO and KEGG functional enrichment analyses were performed using R package clusterProfiler v3.14.3 (clusterProfiler, RRID:SCR_016884) [85] with the default parameters. Forty-five rapidly expanded gene families were further selected and analyzed to understand the evolution of expanded gene families. Coding sequence (CDS) analysis of each gene family was performed using the PAML package of codeml [86] under the site models. Models applied in this step included M0 (one rate), M1a (neutral)–M2a (selection) and M7 (beta)–M8 (beta&ω) (NSsites = 0 1 2 7 8). A likelihood ratio test compared the results from the M1a–M2a and M7–M8 models (p=0.05). Bayes Empirical Bayes (BEB) inference [87] was used for testing the positive loci in each gene family.

Chromosomal synteny was performed to investigate variation/conservation of chromosomes between *T. dichotomus* and the related beetle *Tri. castaneum* (Coleoptera: Tenebrionidae), whose genome was assembled at the chromosome level with 10 chromosomes (nine autosomal chromosomes and the X chromosome) [64]. Gene and protein sequences were aligned using MMseq2 v11-e1a1c [88] under the default parameters (-s 7.5 --alignment-mode 3 --num-iterations 4 -e 1e-5 --max-accept 5). Synteny analysis was performed using MCScanX [89] with the collinear block containing at least five homologous genes (-s 5 -e 1e-10). A chromosome synteny diagram was visualized using TBtools v1.0692 [90].

2.5 Intestinal transcriptome analysis

Raw reads were further filtered by fastp to remove adapters and low quality bases ("N">10%, Q-value⩽20). The rRNA reads were found and removed by mapping short reads to the rRNA database of *T. dichotomus* with Bowtie2 (version 2.2.8; RRID:SCR_016368) [91]. The remaining clean reads were mapped to the reference genome using HISAT2 with "-rna-strandness RF" and other parameters set as the default. The mapped reads of each sample were assembled using StringTie in a reference-based approach. For each transcription region, a FPKM (fragment per kilobase of transcript per million mapped reads) value was calculated to quantify its expression abundance and variations, using RSEM software [92].

Based on FPKM, permutational multivariate analysis of variance (PERMANOVA) was performed with 999 permutations using the R package 'vegan' [93]. Principal component analysis (PCA) and Pearson correlation coefficient (PCC) were performed with the R package gmodels [94]. Differential gene expression analysis was performed with DESeq2 (DESeq2, RRID:SCR_015687) [95] software, with a shrinkage estimator for dispersion between different gut tissues from the same diet, or the same gut tissues from different diets. The genes associated with a false discovery rate (FDR) below 0.05 and absolute fold change ≥ 2 (|FC|≥2) were considered as differentially expressed genes. All differentially expressed digestion-related genes were further annotated with KEGG pathways and GO terms. Digestion-related genes were then filtered to exclude those with mean gene counts

less than five within a group for all groups. A heatmap of differentially expressed digestion-related genes was visualized using TBtools.

3. Results and Discussion

3.1 Genome estimation

Before ONT sequencing, 25 Gb (more than 40×) of Illumina DNA data from a genome survey with GC-content of 35.85% were obtained for sample quality and genome assessment (Table S3). By analyzing the 17-mer depth distribution from the 350-bp library cleaned sequencing reads, the genome size and repeat ratio of *T. dichotomus* were estimated to be 630.93 Mb and 32.29% in FindGSE, and 567.40 Mb and 22.99% in GenomeScope (Fig. S1; Table S4). Further combined with the simulation results, the final genome size of *T. dichotomus* was estimated to be about 599.17 Mb, with a 2.09% heterozygous ratio.

The N50 and the mean length of the long reads were 24.54 and 16.88 Kb, respectively; with the longest read of 170.57 Kb. Furthermore, 12 Gb of Illumina RNA data were obtained from thoracic muscle for genome evaluation and annotation (Table S3).

Table 1. Genome assembly and quality evaluation

Assembly	Total length	Number of	N50 length	Longest scaffold	GC (%)	BUSCO (n = 1,367) (%)			
						C	D	F	M

	(Mb)	scaffolds	(Mb)	(Mb)					
NextDenovo	636.56	304	14.44	27.42	35.12	98.8	0.9	0.8	0.4
3D-DNA	636.61	496	71.04	94.63	35.12	98.7	0.8	0.9	0.4
Final	636.27	417	71.04	94.63	35.11	98.7	0.8	0.9	0.4

Note: C, complete BUSCOs; D, complete and duplicated BUSCOs; F, fragmented BUSCOs; M, missing BUSCOs.

3.2 Genome assembly and assessment

ONT sequencing generated 73 Gb (approximately 120×) of pass reads (Table S3), which were then corrected by the NextCorrect module (NextDenovo) and produced 45 Gb of consensus sequences. The preliminary assembly was generated using the NextGraph module (NextDenovo), with the genome size of 634.66 Mb and N50 length of 14.42 Mb. After being corrected and polished by Racon and Nextpolish, the polished genome size was 636.56 Mb, with the scaffold N50 length of 14.44 Mb (Table 1), suggesting a good continuity of our assembled genome (Fig. 2a).

The genome of *T. dichotomus* was further sequenced by NovaSeq sequencing, which generated 83 Gb of Hi-C data (Table S3), and was filtered to produce 79 Gb of clean data. Based on the clean data in the 3D-DNA analysis, the chromosome-anchored genome size was estimated to be 636.61 Mb, with 496 scaffolds and an N50 length of 71.04 Mb (Table 1). After polishing, removing redundancy and contaminants, and Hi-C scaffolding, the final genome size was determined to be 636.27 Mb, composed of 417 scaffolds, with a scaffold/contig N50 length of 71.04/12.99 Mb, GC-content of 35.11%, and gaps of 0.004% (Table S5), which was close to the earlier genome estimation by FindGSE. Furthermore, 606.8 Mb scaffolds covering 95.37% of the draft reference genome were precisely

anchored onto 10 pseudo-chromosomes (Fig. 2b), indicating a high-quality of the chromosome-level genome assembly.

Taking all the published genomes of Scarabaeidae into account, we found that the genomic characteristics varied significantly among the eight retrievable genomes of scarabaeid beetles, with a genome size of 267–1144 Mb [96-98]. A draft genome assembly of *T. dichotomus* was recently released in GenBank (Bioproject: PRJDB10500; genome size of 739.41 Mb; contig N50 length of 7.93 Mb; contig number of 2,347) without further analysis. Its BUSCO assessment (n = 1,367) identified 1,352 (98.9%) complete BUSCOs, comprised of 1,340 (98.0%) single-copy and 12 (0.9%) duplicated BUSCOs. In comparison, the size of our genome was smaller than that of the released genome assembly, probably due to the scaffold assembly level we used. Furthermore, our genome assembly showed a longer scaffold N50 (71.04 Mb) and a smaller scaffold number (414) than that of the released one. We found that *T. dichotomus* has a relatively larger genome than most other scarabaeids, but a similar GC-content close to 35% (except 25% for *Protaetia brevitarsis*, Bioproject: PRJNA477715). The clearest example of genomic difference was found in its closest relative species from the same subfamily, *Onthophagus taurus*, with a much smaller genome size of 267.08 Mb (Bioproject: PRJNA419349).

Using BUSCO assessment (n = 1,367), we identified 1,349 (98.7%) conserved orthologous as complete genes, with 97.9% “complete and single-copy BUSCOs” and 0.8% “complete and duplicated BUSCOs” genes represented (Table 1). The

mapping rates of Illumina DNA data from the genome survey, Illumina RNA data of the male thorax and ONT data onto our draft genome were as high as 99.89, 95.39, and 99.60%, respectively. These results indicate that the genome assembly of *T. dichotomus* in this study reached an extremely high quality in both continuity and integrity.

3.3 Genome annotation

A total of 1,369,555 repeat sequences (365,506,399 bp) were identified, accounting for 57.45% of the whole genome, with the top six represented as DNA elements (28.97%), unclassified (16.67%), LINEs (9.69%), LTRs (1.24%), SINEs (0.52%) and simple repeats (0.52%) (Table S6). The density of each type (except unclassified) was shown on each chromosome, indicating that DNA elements and LINEs have the maximum densities (Fig. 3).

To predict the genes in *T. dichotomus*, we employed MAKER pipeline and generated 12,193 PCGs, among which the average length of genes, CDS and transcripts was 15,150, 1,743, and 2,355 bp, respectively. On average, the size of the exons and introns was 339 and 1,857 bp, respectively (Table S5), which is common in organisms with large genomes [99]. Furthermore, BUSCO assessment (n = 1,367) identified 95.8% (S: 85.4%, D: 10.4%) of the conserved orthologous as complete genes in the predicted PCGs, indicating that our prediction was relatively complete.

After PCG functional annotation, 11,551 (94.73%) genes were detected

matching the UniprotKB records by Diamond, while 10,640 (87.26%) protein domains of PCGs were identified using InterProScan. In addition, we also identified 10,535 GO, 8,224 KEGG ko, 2,886 enzyme codes, 9,431 KEGG pathways, 10,590 reactome pathways and 12,025 COG categories by InterProScan and eggNOG-mapper. To evaluate these datasets, we compared them with other high-quality genome annotations from six insects and revealed more than 10,000 hits (Table 2).

Based on the annotation by Rfam database and tRNAscan-SE, we identified 668 ncRNAs in the genome, including 43 rRNAs, 57 miRNAs, 129 snRNAs, two long noncoding RNAs (lncRNAs), two ribozymes, 361 tRNAs, and 74 other ncRNAs. Twenty-one isotypes of tRNAs were annotated in this species, however, the Supres isotype was missing. We also identified 129 snRNAs, with 106 spliceosomal RNAs (U1, U2, U4, U5, U6 and U11), five minor spliceosomal RNAs (U4atac, U6atac and U12), 14 C/D box snoRNAs, three H/ACA box snoRNAs and one other snRNA (SCARNA8) (Table S7).

Table 2. Gene hits between *Trypoxylus dichotomus* and another six insects

Species	Gene number	Hit number
Trypoxylus dichotomus	12,193	-
Onthophagus taurus	15,366	11,329
Tribolium castaneum	12,657	11,178
Anoplophora glabripennis	14,698	11,144
Apis mellifera	12,739	10,365
Bombyx mori	13,683	10,381
Drosophila melanogaster	13,617	10,135

3.4 Comparative genome and phylogeny

Gene family identification

Using homology analysis of the gene family, 181,904 (92.90%) genes were clustered into 14,467 orthogroups (gene families), in which 12,658 orthogroups were unique to beetles. Moreover, there were 1,260 single-copy orthogroups and 3,120 multi-copy orthogroups identified for *T. dichotomus*. Among the PCGs in the genome of this beetle, 11,614 (95.25%) genes were clustered into 8,727 orthogroups, in which 107 orthogroups/488 genes were specific to *T. dichotomus* (Table 3, Fig. 4a).

Table 3. Statistics of gene families among fourteen insects

Category	Number
Number of species	14
Number of genes	195,765
Number of genes in orthogroups	181,904
Number of unassigned genes	13,861
Percentage of genes in orthogroups	92.9
Number of orthogroups	14,467
Number of species-specific orthogroups	3,396
Number of genes in species-specific orthogroups	15,299
Percentage of genes in species-specific orthogroups	7.8
Mean orthogroup size	12.6
Number of orthogroups with all species present	4,380
Number of single-copy orthogroups	1,260

Phylogeny and gene family evolution

After removing 152 single-copy orthologs using symtest, the remaining 1,108 single-copy orthologs (450,544 amino acids) were concatenated for the phylogenetic tree construction (Fig. 4a). The phylogenetic relationships of 14 insect species were well recovered [83, 100], with all the nodes being strongly supported (UFB/SH-aLRT = 100/100), showing a good resolution in the phylogram. Coinciding with the previous

beetle phylogenomic study [100], our results indicated that Coleoptera originated in the Early Carboniferous (320 Mya), while the split of the ancestors of *T. dichotomus* and its closely related scarabaeid species *O. taurus* occurred in the early Cretaceous (120 Mya) (Fig. S2).

To investigate the rapidly evolving gene families in *T. dichotomus*, we used gene family evolution analysis and revealed that 610 and 1,405 gene families had experienced expansions and contractions, respectively, in which 67 gene families (45 expansions and 22 contractions) were recognized as rapidly evolving orthogroups (Fig. 4a). The significantly expanded gene families were primarily associated with digestion (trypsin, enoyl-(acyl carrier protein) reductase), detoxification (cytochrome P450, ecdysteroid kinase, carboxylesterase, aldo/keto reductase), chemoreception (odorant receptor, gustatory receptor), glycometabolism (facilitated trehalose transporter, neutral alpha-glucosidase), immunity (15-hydroxyprostaglandin dehydrogenase [NAD(+)], galectin, serine protease Hayan, prostaglandin reductase 1, inducible metalloproteinase inhibitor protein), development (haemolymph juvenile hormone binding protein (JHBP), juvenile hormone acid O-methyltransferase, serine protease snake) and toxoprotein (venom acid phosphatase) (Fig. 5a; Table S8).

The rapidly expanded gene families were further confirmed in the GO and KEGG enrichments (Tables S9 and S10), with metabolic detoxification, digestion and immunity mainly in the GO enrichment (Fig. 5b), and metabolic detoxification, digestion, juvenile hormone and secondary metabolite synthesis mainly in the KEGG

pathway (Fig. 5c). Four gene families were positively selected, including serine protease Hayan (OG0000411), phosphatidylinositol phosphatase (OG0001456), Hsp70 protein (OG0009015), and nucleoporin autopeptidase (OG0009016), which were related to immunity, cell proliferation/differentiation, heat shock proteins and nucleo-cytoplasmic transport, respectively (Table S11). These results indicated that digestion and detoxification were significantly reflected in the rapidly expanded gene families and functional enrichment in the *T. dichotomus* genome.

Most beetles were considered not to capitalize on their significant ability for endogenous lignocellulose digestion [101], but this is not the case for *T. dichotomus*. Our results revealed that the functional capacity of digestion was obviously reinforced by expansions of digestion-related gene families [102] in the evolution of *T. dichotomus*, which would greatly promote lignocellulose digestion. Additionally, the detoxification function was also reinforced by gene family expansion and positive selection, suggesting an adaptive evolution responding to environmental exposures [103, 104]. This was further supported by the diversification of expression patterns of *T. dichotomus* that adapted to different humus resources [105].

Synteny

To investigate the chromosomal evolution in *T. dichotomus*, we carried out a synteny analysis and generated 262 collinear blocks based on 4,477 collinear genes (18.69% of all genes), with 6–23 genes in each block (Table S12). Chromosomes 1–7 and 9–

10 of *T. dichotomus* (TdChr1–7 and 9–10) were mapped to chromosomes 3, 7, 5, 4, 9, 2, 8, 6 and X of *Tri. castaneum* (TcChr3, 7, 5, 4, 9, 2, 8, 6 and X), with strong syntenic relationships. Only chromosome 8 of *T. dichotomus* (TdChr8) showed a relatively low synteny with chromosome 10 of *Tri. castaneum* (TcChr10) (Fig. 4b). These results indicated a high genome synteny between *T. dichotomus* and *Tri. castaneum*, which clearly reveals an overall conservation of chromosomes in *T. dichotomus* [106]. Furthermore, TdChr10 was mapped to TcChrX perfectly (Fig. 4b), suggesting that TdChr10 was the X chromosome in *T. dichotomus* [107].

Collinear genes were intersected within homologous chromosomes extensively (Fig. 4b), indicating a common reshuffling of gene orders within chromosomes, i.e., intrachromosomal rearrangements (inversions) [108]. In contrast, collinear genes were occasionally intersected among nonhomologous chromosomes, with only five pairs of interchromosomal rearrangements (translocations) (TdChr1-TcChr8, TdChr3-TcChr3, TdChr6-TcChr3, TdChr6-TcChr10 and TdChr8-TcChr2) [109]. Notably, TdChr6 and 8 were significantly intersected with TcChr2 and 10, respectively, indicating a wide variety of chromosome breakages and rearrangements [108] during the evolutionary history of *T. dichotomus*.

Although the clades of *T. dichotomus* (Scarabaeoidea) and *Tri. castaneum* (Tenebrionoidea) diverged in the late Permian (Fig. 4a), their chromosomes (autosomes and X chromosome) were conserved on account of the relatively limited translocations, which might indicate relative conservation of chromosomes in the

evolutionary history of beetles, at least to some extent. In contrast to the autosomes, X chromosome was considered to be more conserved and more recalcitrant to rearrangement than that of the autosomes in insects [106, 109-111], which is consistent with our results for *T. dichotomus*. Therefore, we assume that the intrachromosomal rearrangements are possibly the main evolutionary force for beetles, and autosome rearrangements may be the most important factor. Nevertheless, in spite of the occasional occurrences, interchromosomal rearrangements of autosomes might also play a vital role in the evolutionary process of beetles.

3.5 Gene expression and sample correlation

To further explore the intestinal gene expression patterns associated with different gut tissues and food habits, we carried out intestinal transcriptome analysis for the larvae of *T. dichotomus*. Based on the gene expression (FPKM) of all annotated genes for each sample by PERMANOVA, we found significant differences of gene expressions between the groups separated by gut tissues or food habits (Table 4). PCA and PCC (Tables S13 and S14) were then used to calculate and plot diagrams (Fig. 6), respectively. With PCA analysis (Fig. 6a), we showed that samples from the same group were mainly aggregated together, except for four outliers (SM2, SM6, SH2 and SH3) in the midgut and hindgut of sawdust feeding beetles. Similarly, PCC analysis (Fig. 6b) also displayed good repeatability within most of the intra-groups, but a

relatively low level of repeatability in the midgut of sawdust feeding larvae was due to the abnormal values of SM2.

For the groups with the same food habits (SM and SH, MM and MH), more significant differences of gene expressions were observed between the midgut and hindgut in the sawdust groups than in the mushroom-residue groups along PC1 and PC2. Furthermore, there were also significant differences between groups within the same gut tissue (SM vs MM, SH vs MH), suggesting that intestinal gene expressions could be significantly affected by food habits in *T. dichotomus*. Consistently, it was reported that different host diets could significantly affect the digestive physiology of the beetle, *Trogoderma granarium* [28].

Table 4. Permutational multivariate analysis of variance (PERMANOVA) among groups separated by gut tissue and food habit

**Groups**		**Mean squares**	***df***	R^2	***p***
Tissue	SM vs SH	0.91	1	0.63	**
	MM vs MH	0.56	1	0.77	**
Food	SM vs MM	0.30	1	0.42	**
	SH vs MH	0.23	1	0.46	**

Note: **, $p < 0.01$; SM, midgut from sawdust; SH, hindgut from sawdust; MM, midgut from mushroom-residue; MH, hindgut from mushroom-residue.

3.6 Differentially expressed digestion-related genes

To understand the digestive ability of *T. dichotomus* larvae on different gut tissues and food habits, digestion-related genes were filtered (Table S15) and differentially expressed genes were further compared within four different treatment groups, i.e., SM vs SH, MM vs MH, SM vs MM and SH vs MH (Table S16, Fig. 7). A total of 222

differentially expressed digestion-related genes were identified in the midgut and hindgut from the sawdust groups, in which 128 and 94 genes were highly expressed in the midgut and hindgut (SM vs SH), respectively (Fig. 7a). Similarly, 231 differentially expressed digestion-related genes were detected in the midgut and hindgut from mushroom-residue groups, among which 137 and 94 genes were highly expressed in the midgut and hindgut (MM vs MH), respectively (Fig. 7b). These results indicate that more digestion-related genes are highly expressed in the midgut than the hindgut of larvae, regardless of food habit. Thus, the digestion of lignocellulose in larvae may require more digestive enzymes in the midgut than in the hindgut. To some extent, this is consistent with previous studies showing that polysaccharide degradation occurs mainly in the midgut of the rhinoceros beetle [19, 27].

The highly expressed digestion-related genes in the gut varied between the sawdust and mushroom-residue groups. Of the 92 differentially expressed digestion-related genes in the midguts from two different food habits, 65 and 27 genes were highly expressed in the mushroom-residue group and the sawdust group (SM vs MM), respectively (Fig. 7c). Similarly, 83 differentially expressed digestion-related genes were detected in the hindguts, among which 52 and 31 genes were highly expressed in the mushroom-residue group and the sawdust group (SH vs MH), respectively (Fig. 7d). Taken together, more digestion-related genes were highly expressed in the mushroom-residue group than in the sawdust group

regardless of whether the specific location was the midgut or hindgut. These results suggest that digestion of mushroom-residue might require a greater digestive ability than that of sawdust for the larvae of *T. dichotomus*, which is probably due to the complex components of mushroom-residue, including not only wood fiber but also fungal mycelia.

The rhinoceros beetle may serve as an efficient decomposer in lignocellulose-enriched agro-forestry residues including mushroom-residue and decaying wood, which would provide an environmentally-friendly method for sustainable development. In the forest, the larvae of *T. dichotomus* usually inhabit soil organic matter and feed on decayed wood [19, 20, 27]. This is similar to the living and feeding habitats of the white-spotted flower chafer, *Protaetia brevitarsis* (Scarabaeidae), which also efficiently digests high lignocellulosic mushroom-residue [112]. Interestingly, both species were often observed coexisting in the outdoor mushroom-residue, showing that these two scarab beetles might share an overlapping ecological niche and promote more effective lignocellulosic degradation through close cooperation.

4. Conclusion

In this study, we assembled and provided the chromosome-level genome of *T. dichotomus* in the family Scarabaeidae. Combing different assembling methods, we concluded the final genome size to be 636.27 Mb with the BUSCO completeness up

to 98.7%, indicating a high quality of our genome assembly. Furthermore, 95.37% scaffolds in the draft genome were anchored onto 10 chromosomes, and chromosome 10 was further identified as the X chromosome (sex chromosome) of *T. dichotomus*. In addition, the result of synteny analysis showed that chromosomes 6 and 8 of *T. dichotomus* were intersected with chromosome 2 and 10 of *Tri. castaneum*, revealing that chromosome breakages and rearrangements evolutionarily occurred in *T. dichotomus*. Based on 1,108 single-copy orthologs, the phylogenetic relationships of the beetles were recovered, showing that the ancestor of *T. dichotomus* diverged in the early Cretaceous (120 Mya) from that of the closely related species *O. taurus*.

Interestingly, gene families that associated with digestion and detoxification were significantly expanded in the evolutionary history of *T. dichotomus*, indicating improved adaptation to the local environment by the rhinoceros beetle. This is supported by the high degradation efficiency of lignocellulosic biomass and extensive adaptability to humus environment at the larval stage. Through a comparative analysis of intestinal transcriptome of larvae feeding on sawdust and mushroom-residue, we found that intestinal gene expressions could be significantly affected by food habits in *T. dichotomus*. Digestion-related genes were more commonly expressed in the midgut or mushroom-residue group than hindgut or sawdust group, despite different food treatments or gut tissue treatments. In conclusion, chromosome-level genome assembly and larval intestinal transcriptome analyses will facilitate future genetic studies on the lignocellulose degradation in *T.*

dichotomus, as well as effective utilization of *T. dichotomus* in the eco-friendly biotreatment of plant biomass. Furthermore, the well-assembled and annotated genomic data in this study will provide a valuable resource for further understanding the evolutionary history of beetles and the functions of specific genes.

Data Availability

The data sets supporting the results of this article are available in the GenBank repository. The whole genome sequencing and assembly project has been deposited at GenBank (NCBI BioProject: PRJNA688811). The chromosome-level genome assembly of *Trypoxylus dichotomus* has been stored in the NCBI database under Accession no. JAENHH000000000. All the sequencing raw data, including genome survey, Nanopore, Hi-C and RNA sequencing, have been submitted to the BioProject PRJNA688811. All supporting data and materials are available in the *GigaScience* GigaDB database [113].

List of abbreviations

BEB: Bayes Empirical Bayes; BUSCO: benchmarking universal single-copy orthologs; CDS: Coding sequence; DEPC: diethyl pyrocarbonate; ESEM: environment scanning electron microscope; FC: fold change; FDR: false discovery rate; FPKM: fragment per kilobase of transcript per million mapped reads; GO: gene ontology; GSS: genome survey sequences; JHBP: haemolymph juvenile hormone binding protein; KEGG: Kyoto Encyclopedia of Genes and Genomes; LINE: long interspersed nuclear element; lncRNA: long noncoding RNA; LTR: long terminal repeat; MH: hindgut of larva feeding mushroom-residue; miRNA: micro RNA; ML: Maximum likelihood; MM: midgut of larva feeding mushroom-residue; ncRNAs: noncoding RNAs; PBS:

phosphate-buffered saline; PCA: principal component analysis; PCC: Pearson correlation coefficient; PCG: Protein-coding gene; PERMANOVA: permutational multivariate analysis of variance; rRNA: ribosomal RNA; SH: hindgut of larva feeding sawdust; SINE: short interspersed nuclear element; SM: midgut of larva feeding sawdust; snRNA: small nuclear RNA; SRH: stationary, reversible, and homogeneous.

Competing interests

The authors declare that they have no competing interests.

Author contributions

Qingyun Wang: Methodology, Software, Validation, Formal analysis, Investigation, Data process, Writing - original draft, Visualization. **Junhao Huang:** Conceptualization, Resources, Writing - review & editing, Project administration, Funding acquisition. **Liwei Liu:** Conceptualization, Writing – review & editing. **Sujiong Zhang:** Resources, Writing - review & editing. **Hong Wu:** Supervision.

Acknowledgements

We express our special thanks to Dr. Feng Zhang (Nanjing Agricultural University, China) for the help in data analyses, and Dr. Pu Tang (Zhejiang University, China) for the helpful advice during the initial stage of this research. We also thank Dr. Shouke Zhang (Zhejiang A&F University, China) and Dr. Huaijun Xue (Nankai University, China) for their kind suggestions in larval experimental design, Jinliang Bao (Shanzhizhou Ecological Agriculture Company Limited, Zhejiang, China) who supplied the adults and larvae of the rhinoceros beetle for this study, and Mr. Kui Long (Zhejiang A&F University, China) for his help in the PERMANOVA analysis. We are particularly thankful to the reviewers for their valuable comments and suggestions on the manuscript. This work was supported by Cooperation Project of Zhejiang Province and Chinese Academy of Forestry (Grant No. 2020SY08).

References

1. Cragg SM, Beckham GT, Bruce NC, Bugg TD, Distel DL, Dupree P, et al. Lignocellulose degradation

mechanisms across the Tree of Life. Curr Opin Chem Biol. 2015;29:108-19. doi:10.1016/j.cbpa.2015.10.018.

2. Tan J, Li Y, Tan X, Wu H, Li H and Yang S. Advances in pretreatment of straw biomass for sugar production. Frontiers in Chemistry. 2021;9. doi:10.3389/fchem.2021.696030.

3. Sanderson K. Lignocellulose: a chewy problem. Nature. 2011;474 7352:S12-S4. doi:10.1038/474S012a.

4. Shah SN, Mo KH, Yap SP, Yang J and Ling T-C. Lightweight foamed concrete as a promising avenue for incorporating waste materials: A review. Resources, Conservation and Recycling. 2021;164:105103. doi:10.1016/j.resconrec.2020.105103.

5. Chen J, Fan X, Jiang B, Mu L, Yao P, Yin H, et al. Pyrolysis of oil-plant wastes in a TGA and a fixed-bed reactor: thermochemical behaviors, kinetics, and products characterization. Bioresour Technol. 2015;192:592-602. doi:10.1016/j.biortech.2015.05.108.

6. Sun J, Peng H, Chen J, Wang X, Wei M, Li W, et al. An estimation of CO2 emission via agricultural crop residue open field burning in China from 1996 to 2013. Journal of Cleaner Production. 2016;112:2625-31. doi:10.1016/j.jclepro.2015.09.112.

7. Scully ED, Geib SM, Hoover K, Tien M, Tringe SG, Barry KW, et al. Metagenomic profiling reveals lignocellulose degrading system in a microbial community associated with a wood-feeding beetle. PLoS One. 2013;8 9:e73827. doi:10.1371/journal.pone.0073827.

8. Gales A, Chatellard L, Abadie M, Bonnafous A, Auer L, Carrère H, et al. Screening of phytophagous and xylophagous insects guts microbiota abilities to degrade lignocellulose in bioreactor. Front Microbiol. 2018;9:2222. doi:10.3389/fmicb.2018.02222.

9. Himmel ME, Ding S-Y, Johnson DK, Adney WS, Nimlos MR, Brady JW, et al. Biomass recalcitrance: engineering plants and enzymes for biofuels production. Science. 2007;315 5813:804-7. doi:10.1126/science.1137016.

10. Sun J and Zhou XJ. Utilization of lignocellulose-feeding insects for viable biofuels: an emerging and promising area of entomological science. In: Liu T and Kang L, editors. Recent Advances in Entomological Research. Berlin Heidelberg: Higher Education Press, Beijing and Springer-Verlag; 2011. p. 434-500.

11. Geib SM, Filley TR, Hatcher PG, Hoover K, Carlson JE, del Mar Jimenez-Gasco M, et al. Lignin degradation in wood-feeding insects. Proceedings of the National Academy of Sciences. 2008;105 35:12932-7. doi:10.1073/pnas.0805257105.

12. Bayané A and Guiot SR. Animal digestive strategies versus anaerobic digestion bioprocesses for biogas production from lignocellulosic biomass. Reviews in Environmental Science and Bio/Technology. 2011;10 1:43-62. doi:10.1007/s11157-010-9209-4.

13. Luo C, Li Y, Chen Y, Fu C, Long W, Xiao X, et al. Bamboo lignocellulose degradation by gut symbiotic microbiota of the bamboo snout beetle *Cyrtotrachelus buqueti*. Biotechnology for Biofuels. 2019;12 1:1-16. doi:10.1186/s13068-019-1411-1.

14. Warnecke F, Luginbühl P, Ivanova N, Ghassemian M, Richardson TH, Stege JT, et al. Metagenomic and functional analysis of hindgut microbiota of a wood-feeding higher termite. Nature. 2007;450 7169:560-5. doi:10.1038/nature06269.

15. Brune A. Symbiotic digestion of lignocellulose in termite guts. Nature Reviews Microbiology. 2014;12 3:168-80. doi:10.1038/nrmicro3182.

16. Li H, Yelle DJ, Li C, Yang M, Ke J, Zhang R, et al. Lignocellulose pretreatment in a fungus-cultivating

termite. Proceedings of the National Academy of Sciences. 2017;114 18:4709-14. doi:10.1073/pnas.1618360114.

17. Sethi A and Scharf ME. Biofuels: fungal, bacterial and insect degraders of lignocellulose. eLS. 2013; doi:10.1002/9780470015902.a0020374.

18. Yang H, You CJ, Tsui CK, Tembrock LR, Wu ZQ and Yang DP. Phylogeny and biogeography of the Japanese rhinoceros beetle, *Trypoxylus dichotomus* (Coleoptera: Scarabaeidae) based on SNP markers. Ecol Evol. 2021;11 1:153-73. doi:10.1002/ece3.6982.

19. Wada N, Sunairi M, Anzai H, Iwata R, Yamane A and Nakajima M. Glycolytic activities in the larval digestive tract of *Trypoxylus dichotomus* (Coleoptera: Scarabaeidae). Insects. 2014;5 2:351-63. doi:10.3390/insects5020351.

20. Kojima W. Attraction to carbon dioxide from feeding resources and conspecific neighbours in larvae of the rhinoceros beetle *Trypoxylus dichotomus*. PLoS One. 2015;10 11:e0141733. doi:10.1371/journal.pone.0141733.

21. Eo J, Na Y-E and Kim M-H. Influence of rhinoceros beetle (*Trypoxylus dichotomus septentrionalis*) larvae and temperature on the soil bacterial community composition under laboratory conditions. Soil Biol Biochem. 2017;108:27-35. doi:10.1016/j.soilbio.2016.12.005.

22. Bao Jl, Wang P, Zhang SJ and Chen ZL. *Forest-fungus-insect circular ecological breeding method*. Patent CN109964723 (A), China, 2019.

23. Schmidt MW, Torn MS, Abiven S, Dittmar T, Guggenberger G, Janssens IA, et al. Persistence of soil organic matter as an ecosystem property. Nature. 2011;478 7367:49-56. doi:10.1038/nature10386.

24. Cotrufo MF, Soong JL, Horton AJ, Campbell EE, Haddix ML, Wall DH, et al. Formation of soil organic matter via biochemical and physical pathways of litter mass loss. Nature Geoscience. 2015;8 10:776-9. doi:10.1038/NGEO2520.

25. Takeishi H, Anzai H, Urai M, Aizawa T, Wada N, Iwabuchi N, et al. Xylanolytic and alkaliphilic *Dietzia* sp. isolated from larvae of the Japanese horned beetle, *Trypoxylus dichotomus*. Actinomycetologica. 2006;20 2:49-54. doi:10.3209/saj.20.49.

26. Aizawa T, Urai M, Iwabuchi N, Nakajima M and Sunairi M. *Bacillus trypoxylicola* sp. nov., xylanase-producing alkaliphilic bacteria isolated from the guts of Japanese horned beetle larvae (*Trypoxylus dichotomus septentrionalis*). Int J Syst Evol Microbiol. 2010;60 1:61-6. doi:10.1099/ijs.0.005843-0.

27. Wada N, Iwabuchi N, Sunairi M, Nakajima M, Iwata R and Anzai H. Site-specific profiles of biochemical properties in the larval digestive tract of Japanese rhinoceros beetle, *Trypoxylus dichotomus* (Coleoptera: Scarabaeidae). Entomological Science. 2020;23 1:33-43. doi:10.1111/ens.12394.

28. Borzou E, Naseri B and Namin FR. Different diets affecting biology and digestive physiology of the Khapra beetle, *Trogoderma granarium* Everts (Coleoptera: Dermestidae). Journal of Stored Products Research. 2015;62:1-7. doi:10.1016/j.jspr.2015.03.003.

29. Holtof M, Lenaerts C, Cullen D and Broeck JV. Extracellular nutrient digestion and absorption in the insect gut. Cell Tissue Res. 2019;377:397–414. doi:10.1007/s00441-019-03031-9.

30. Dutrillaux A-M, Mamuris Z and Dutrillaux B. Chromosome analyses challenge the taxonomic position of *Augosoma centaurus* Fabricius, 1775 (Coleoptera: Scarabaeidae: Dynastinae) and the separation of Dynastini and Oryctini. Zoosystema. 2013;35 4:537-49. doi:10.5252/z2013n4a7.

31. Chen S, Zhou Y, Chen Y and Gu J. fastp: an ultra-fast all-in-one FASTQ preprocessor. Bioinformatics. 2018;34 17:i884-i90. doi:10.1093/bioinformatics/bty560.

32. Marçais G and Kingsford C. A fast, lock-free approach for efficient parallel counting of occurrences of k-mers. Bioinformatics. 2011;27 6:764-70. doi:10.1093/bioinformatics/btr011.
33. Sun H, Ding J, Piednoël M and Schneeberger K. findGSE: estimating genome size variation within human and Arabidopsis using k-mer frequencies. Bioinformatics. 2018;34 4:550-7. doi:10.1093/bioinformatics/btx637.
34. Vurture GW, Sedlazeck FJ, Nattestad M, Underwood CJ, Fang H, Gurtowski J, et al. GenomeScope: fast reference-free genome profiling from short reads. Bioinformatics. 2017;33 14:2202-4. doi:10.1093/bioinformatics/btx153.
35. Li H. Minimap2: pairwise alignment for nucleotide sequences. Bioinformatics. 2018;34 18:3094-100. doi:10.1093/bioinformatics/bty191.
36. Camacho C, Coulouris G, Avagyan V, Ma N, Papadopoulos J, Bealer K, et al. BLAST+: architecture and applications. BMC Bioinformatics. 2009;10 1:1-9. doi:10.1186/1471-2105-10-421.
37. Simão FA, Waterhouse RM, Ioannidis P, Kriventseva EV and Zdobnov EM. BUSCO: assessing genome assembly and annotation completeness with single-copy orthologs. Bioinformatics. 2015;31 19:3210-2. doi:10.1093/bioinformatics/btv351.
38. Li H, Handsaker B, Wysoker A, Fennell T, Ruan J, Homer N, et al. The sequence alignment/map format and SAMtools. Bioinformatics. 2009;25 16:2078-9. doi:10.1093/bioinformatics/btp352.
39. Belton J-M, McCord RP, Gibcus JH, Naumova N, Zhan Y and Dekker J. Hi-C: a comprehensive technique to capture the conformation of genomes. Methods. 2012;58 3:268-76.
40. Durand NC, Shamim MS, Machol I, Rao SS, Huntley MH, Lander ES, et al. Juicer provides a one-click system for analyzing loop-resolution Hi-C experiments. Cell Systems. 2016;3 1:95-8. doi:10.1016/j.cels.2016.07.002.
41. Dudchenko O, Batra SS, Omer AD, Nyquist SK, Hoeger M, Durand NC, et al. De novo assembly of the Aedes aegypti genome using Hi-C yields chromosome-length scaffolds. Science. 2017;356 6333:92-5. doi:10.1126/science.aal3327.
42. Flynn JM, Hubley R, Goubert C, Rosen J, Clark AG, Feschotte C, et al. RepeatModeler2 for automated genomic discovery of transposable element families. Proceedings of the National Academy of Sciences. 2020;117 17:9451-7. doi:10.1073/pnas.1921046117.
43. Chen N. Using Repeat Masker to identify repetitive elements in genomic sequences. Current Protocols in Bioinformatics. 2004;5 1:4.10.1-4..4. doi:10.1002/0471250953.bi0410s05.
44. Holt C and Yandell M. MAKER2: an annotation pipeline and genome-database management tool for second-generation genome projects. BMC Bioinformatics. 2011;12 1:1-14. doi:10.1186/1471-2105-12-491.
45. Brůna T, Hoff KJ, Lomsadze A, Stanke M and Borodovsky M. BRAKER2: Automatic eukaryotic genome annotation with GeneMark-EP+ and AUGUSTUS supported by a protein database. NAR Genomics and Bioinformatics. 2021;3 1:lqaa108. doi:10.1093/nargab/lqaa108.
46. Stanke M, Steinkamp R, Waack S and Morgenstern B. AUGUSTUS: a web server for gene finding in eukaryotes. Nucleic Acids Res. 2004;32 suppl_2:W309-W12. doi:10.1093/nar/gkh379.
47. Brůna T, Lomsadze A and Borodovsky M. GeneMark-EP+: eukaryotic gene prediction with self-training in the space of genes and proteins. NAR Genomics and Bioinformatics. 2020;2 2:lqaa026. doi:10.1093/nargab/lqaa026.
48. Khan MA, Bhatia P and Sadiq M. BBTool: a tool to generate the test cases. Int J Recent Technol Eng. 2012;1 2:192-7.

49. Kim D, Paggi JM, Park C, Bennett C and Salzberg SL. Graph-based genome alignment and genotyping with HISAT2 and HISAT-genotype. Nat Biotechnol. 2019;37 8:907-15. doi:10.1038/s41587-019-0201-4.
50. Kriventseva EV, Kuznetsov D, Tegenfeldt F, Manni M, Dias R, Simão FA, et al. OrthoDB v10: sampling the diversity of animal, plant, fungal, protist, bacterial and viral genomes for evolutionary and functional annotations of orthologs. Nucleic Acids Res. 2019;47 D1:D807-D11. doi:10.1093/nar/gky1053.
51. Kovaka S, Zimin AV, Pertea GM, Razaghi R, Salzberg SL and Pertea M. Transcriptome assembly from long-read RNA-seq alignments with StringTie2. Genome Biol. 2019;20 1:1-13. doi:10.1186/s13059-019-1910-1.
52. Buchfink B, Xie C and Huson DH. Fast and sensitive protein alignment using DIAMOND. Nature Methods. 2015;12 1:59-60. doi:10.1038/nmeth.3176.
53. El-Gebali S, Mistry J, Bateman A, Eddy SR, Luciani A, Potter SC, et al. The Pfam protein families database in 2019. Nucleic Acids Res. 2019;47 D1:D427-D32. doi:10.1093/nar/gky995.
54. Letunic I and Bork P. 20 years of the SMART protein domain annotation resource. Nucleic Acids Res. 2018;46 D1:D493-D6. doi:10.1093/nar/gkx922.
55. Lewis TE, Sillitoe I, Dawson N, Lam SD, Clarke T, Lee D, et al. Gene3D: extensive prediction of globular domains in proteins. Nucleic Acids Res. 2018;46 D1:D435-D9. doi:10.1093/nar/gkx1187.
56. Wilson D, Pethica R, Zhou Y, Talbot C, Vogel C, Madera M, et al. SUPERFAMILY—sophisticated comparative genomics, data mining, visualization and phylogeny. Nucleic Acids Res. 2009;37 suppl_1:D380-D6. doi:10.1093/nar/gkn762.
57. Marchler-Bauer A, Bo Y, Han L, He J, Lanczycki CJ, Lu S, et al. CDD/SPARCLE: functional classification of proteins via subfamily domain architectures. Nucleic Acids Res. 2017;45 D1:D200-D3. doi:10.1093/nar/gkw1129.
58. Finn RD, Attwood TK, Babbitt PC, Bateman A, Bork P, Bridge AJ, et al. InterPro in 2017—beyond protein family and domain annotations. Nucleic Acids Res. 2017;45 D1:D190-D9. doi:10.1093/nar/gkw1107.
59. Huerta-Cepas J, Szklarczyk D, Heller D, Hernández-Plaza A, Forslund SK, Cook H, et al. eggNOG 5.0: a hierarchical, functionally and phylogenetically annotated orthology resource based on 5090 organisms and 2502 viruses. Nucleic Acids Res. 2019;47 D1:D309-D14. doi:10.1093/molbev/msx148.
60. Huerta-Cepas J, Forslund K, Coelho LP, Szklarczyk D, Jensen LJ, Von Mering C, et al. Fast genome-wide functional annotation through orthology assignment by eggNOG-mapper. Mol Biol Evol. 2017;34 8:2115-22. doi:10.1093/molbev/msx148.
61. Nawrocki EP and Eddy SR. Infernal 1.1: 100-fold faster RNA homology searches. Bioinformatics. 2013;29 22:2933-5. doi:10.1093/bioinformatics/btt509.
62. Chan PP and Lowe TM. tRNAscan-SE: searching for tRNA genes in genomic sequences. Methods Mol Biol. 2019;1962:1-14. doi:10.1007/978-1-4939-9173-0_1.
63. Krzywinski M, Schein J, Birol I, Connors J, Gascoyne R, Horsman D, et al. Circos: an information aesthetic for comparative genomics. Genome Res. 2009;19 9:1639-45. doi:10.1101/gr.092759.109.
64. Richards S, Gibbs RA, Weinstock GM, Brown SJ, Denell R, Beeman RW, et al. The genome of the model beetle and pest *Tribolium castaneum*. Nature. 2008;452 7190:949-55. doi:10.1038/natur e06784.
65. McKenna DD, Scully ED, Pauchet Y, Hoover K, Kirsch R, Geib SM, et al. Genome of the Asian longhorned beetle (*Anoplophora glabripennis*), a globally significant invasive species, reveals key functional and evolutionary innovations at the beetle–plant interface. Genome Biol. 2016;17 1:1-18.

doi:10.1186/s13059-016-1088-8.

66. Chen X, Dong Z, Liu G, He J, Zhao R, Wang W, et al. Phylogenetic analysis provides insights into the evolution of Asian fireflies and adult bioluminescence. Mol Phylogenet Evol. 2019;140:106600. doi:10.1016/j.ympev.2019.106600.

67. Cunningham CB, Ji L, Wiberg RAW, Shelton J, McKinney EC, Parker DJ, et al. The genome and methylome of a beetle with complex social behavior, *Nicrophorus vespilloides* (Coleoptera: Silphidae). Genome Biol Evol. 2015;7 12:3383-96. doi:10.1093/gbe/evv194.

68. Evans JD, McKenna D, Scully E, Cook SC, Dainat B, Egekwu N, et al. Genome of the small hive beetle (*Aethina tumida*, Coleoptera: Nitidulidae), a worldwide parasite of social bee colonies, provides insights into detoxification and herbivory. Gigascience. 2018;7 12:giy138. doi:10.1093/gigascience/giy138.

69. Vargas-Chavez C, Parisot N, Goubert C, Baa-Puyoulet P, Balmand S, Boulesteix M, et al. Evaluating the essentiality of the primary endosymbiont of the rice weevil *Sitophilus oryzae* through genome analysis. In: *VI Meeting of the Spanish Society for Evolutionary Biology (SESBE)* 2018.

70. Adams MD, Celniker SE, Holt RA, Evans CA, Gocayne JD, Amanatides PG, et al. The genome sequence of *Drosophila melanogaster*. Science. 2000;287 5461:2185-95. doi:10.1126/science.

71. Solignac M, Zhang L, Mougel F, Li B, Vautrin D, Monnerot M, et al. The genome of *Apis mellifera*: dialog between linkage mapping and sequence assembly. Genome Biol. 2007;8 3:1-4. doi:10.1186/gb-2007-8-3-403.

72. Consortium ISG. The genome of a lepidopteran model insect, the silkworm *Bombyx mori*. Insect Biochem Mol Biol. 2008;38 12:1036-45. doi:10.1016/j.ibmb.2008.11.004.

73. Itakura S, Yoshikawa Y, Togami Y and Umezawa K. Draft genome sequence of the termite, *Coptotermes formosanus*: Genetic insights into the pyruvate dehydrogenase complex of the termite. Journal of Asia-Pacific Entomology. 2020;23 3:666-74. doi:10.1016/j.aspen.2020.05.004.

74. Chen W, Shakir S, Bigham M, Richter A, Fei Z and Jander G. Genome sequence of the corn leaf aphid (*Rhopalosiphum maidis* Fitch). Gigascience. 2019;8 4:giz033. doi:10.1093/gigascience/giz033.

75. Emms DM and Kelly S. OrthoFinder: phylogenetic orthology inference for comparative genomics. Genome Biol. 2019;20 1:1-14. doi:10.1186/s13059-019-1832-y.

76. Katoh K and Standley DM. MAFFT multiple sequence alignment software version 7: improvements in performance and usability. Mol Biol Evol. 2013;30 4:772-80. doi:10.1093/molbev/mst010.

77. Criscuolo A and Gribaldo S. BMGE (Block Mapping and Gathering with Entropy): a new software for selection of phylogenetic informative regions from multiple sequence alignments. BMC Evol Biol. 2010;10 1:1-21. doi:10.1186/1471-2148-10-210.

78. Kück P and Longo GC. FASconCAT-G: extensive functions for multiple sequence alignment preparations concerning phylogenetic studies. Frontiers in Zoology. 2014;11 1:1-8. doi:10.1186/s12983-014-0081-x.

79. Minh BQ, Schmidt HA, Chernomor O, Schrempf D, Woodhams MD, Von Haeseler A, et al. IQ-TREE 2: new models and efficient methods for phylogenetic inference in the genomic era. Mol Biol Evol. 2020;37 5:1530-4. doi:10.1093/molbev/msaa015.

80. Sanderson MJ. r8s: inferring absolute rates of molecular evolution and divergence times in the absence of a molecular clock. Bioinformatics. 2003;19 2:301-2. doi:10.1093/bioinformatics/19.2.301.

81. https://paleobiodb.org/. Accessed 15 Nov 2021.

82. Nel A, Roques P, Nel P, Prokin AA, Bourgoin T, Prokop J, et al. The earliest known holometabolous insects. Nature. 2013;503 7475:257-61. doi:10.1038/nature1262.

83. Misof B, Liu S, Meusemann K, Peters RS, Donath A, Mayer C, et al. Phylogenomics resolves the timing and pattern of insect evolution. Science. 2014;346 6210:763-7. doi:10.1126/science.1257570.
84. Han MV, Thomas GW, Lugo-Martinez J and Hahn MW. Estimating gene gain and loss rates in the presence of error in genome assembly and annotation using CAFE 3. Mol Biol Evol. 2013;30 8:1987-97. doi:10.1093/molbev/mst100.
85. Yu G, Wang L-G, Han Y and He Q-Y. clusterProfiler: an R package for comparing biological themes among gene clusters. OMICS. 2012;16 5:284-7. doi:10.1089/omi.2011.0118.
86. Yang Z. PAML 4: phylogenetic analysis by maximum likelihood. Mol Biol Evol. 2007;24 8:1586-91. doi:10.1093/molbev/msm088.
87. Yang Z, Wong WS and Nielsen R. Bayes empirical Bayes inference of amino acid sites under positive selection. Mol Biol Evol. 2005;22 4:1107-18. doi:10.1093/molbev/msi097.
88. Steinegger M and Söding J. MMseqs2: sensitive protein sequence searching for the analysis of massive data sets. Nat Biotechnol. 2017;35:1026-8. doi:10.1038/nbt.3988.
89. Wang Y, Tang H, DeBarry JD, Tan X, Li J, Wang X, et al. MCScanX: a toolkit for detection and evolutionary analysis of gene synteny and collinearity. Nucleic Acids Res. 2012;40 7:e49-e. doi:10.1093/nar/gkr1293.
90. Chen C, Chen H, Zhang Y, Thomas HR, Frank MH, He Y, et al. TBtools: an integrative toolkit developed for interactive analyses of big biological data. Molecular Plant. 2020;13 8:1194-202. doi:10.1016/j.molp.2020.06.009.
91. Langmead B and Salzberg SL. Fast gapped read alignment with Bowtie 2. Nature Methods. 2012;9 4:357-9. doi:10.1038/nmeth.1923.
92. Li B and Dewey CN. RSEM: accurate transcript quantification from RNA-Seq data with or without a reference genome. BMC Bioinformatics. 2011;12 1:1-16. doi:10.1186/1471-2105-12-323.
93. Edwards J, Johnson C, Santos-Medellín C, Lurie E, Podishetty NK, Bhatnagar S, et al. Structure, variation, and assembly of the root-associated microbiomes of rice. Proceedings of the National Academy of Sciences. 2015;112 8:E911-E20. doi:10.1073/pnas.1414592112.
94. Gregory R. Warnes BB, Thomas Lumley, Randall C Johnson. . gmodels: Various R Programming Tools for Model Fitting. 2.18.1 ed. 2018.
95. Love MI, Huber W and Anders S. Moderated estimation of fold change and disper sion for RNA seq data with DESeq2. Genome Biol. 2014;15 12:550. doi:10.1186/s13059-014-0550-8.
96. Meyer JM, Markov GV, Baskaran P, Herrmann M, Sommer RJ and Rödelsperger C. Draft genome of the scarab beetle *Oryctes borbonicus* on La Réunion Island. Genome Biol Evol. 2016;8 7:2093-105. doi:10.1093/gbe/evw133.
97. McKenna DD. Beetle genomes in the 21st century: prospects, progress and priorities. Current Opinion in Insect Science. 2018;25:76-82. doi:10.1016/j.cois.2017.12.002.
98. Lee JH, Jung M, Shin Y, Kim I-W, Seo M, Kim M, et al. Draft Genome of the Edible Oriental Insect *Protaetia brevitarsis seulensis*. Frontiers in Genetics. 2020;11:1741. doi:10.3389/fgene.2020.593994.
99. Charlesworth B and Barton N. Genome size: does bigger mean worse? Curr Biol. 2004;14 6:R233-R5. doi:10.1016/j.cub.2004.02.054.
100. McKenna DD, Shin S, Ahrens D, Balke M, Beza-Beza C, Clarke DJ, et al. The evolution and genomic basis of beetle diversity. Proceedings of the National Academy of Sciences. 2019;116 49:24729-37. doi:10.1073/pnas.1909655116.
101. Calderón-Cortés N, Quesada M, Watanabe H, Cano-Camacho H and Oyama K. Endogenous plant cell

wall digestion: a key mechanism in insect evolution. Annu Rev Ecol Evol Syst. 2012;43:45-71. doi:10.1146/annurev-ecolsys-110411-160312.

102. Dunn MJ, Kinney GM, Washington PM, Berman J and Anderson MZ. Functional diversification accompanies gene family expansion of MED2 homologs in Candida albicans. PLoS Genet. 2018;14 4:e1007326. doi:10.1371/journal.pgen.1007326.

103. MacGillivray DM and Kollmann TR. The role of environmental factors in modulating immune responses in early life. Front Immunol. 2014;5:434. doi:10.3389/fimmu.2014.00434.

104. Booker TR, Jackson BC and Keightley PD. Detecting positive selection in the genome. BMC Biol. 2017;15 1:1-10. doi:10.1186/s12915-017-0434-y.

105. Pearce SL, Clarke DF, East PD, Elfekih S, Gordon K, Jermiin LS, et al. Genomic innovations, transcriptional plasticity and gene loss underlying the evolution and divergence of two highly polyphagous and invasive *Helicoverpa* pest species. BMC Biol. 2017;15 1:1-30. doi:10.1186/s12915-017-0402-6.

106. Eichler EE and Sankoff D. Structural dynamics of eukaryotic chromosome evolution. Science. 2003;301 5634:793-7. doi:10.1126/science.1086132.

107. Lorenzen MD, Doyungan Z, Savard J, Snow K, Crumly LR, Shippy TD, et al. Genetic linkage maps of the red flour beetle, *Tribolium castaneum*, based on bacterial artificial chromosomes and expressed sequence tags. Genetics. 2005;170 2:741-7. doi:10.1534/genetics.104.032227.

108. d'Alencon E, Sezutsu H, Legeai F, Permal E, Bernard-Samain S, Gimenez S, et al. Extensive synteny conservation of holocentric chromosomes in Lepidoptera despite high rates of local genome rearrangements. Proceedings of the National Academy of Sciences. 2010;107 17:7680-5. doi:10.1073/pnas.0910413107.

109. Pal A and Vicoso B. The X chromosome of hemipteran insects: conservation, dosage compensation and sex-biased expression. Genome Biol Evol. 2015;7 12:3259-68. doi:10.1093/gbe/evv215.

110. Li Y, Zhang B and Moran NA. The aphid X chromosome is a dangerous place for functionally important genes: diverse evolution of hemipteran genomes based on chromosome-level assemblies. Mol Biol Evol. 2020;37 8:2357-68. doi:10.1093/molbev/msaa095.

111. Mathers TC, Wouters RH, Mugford ST, Swarbreck D, Van Oosterhout C and Hogenhout SA. Chromosome-scale genome assemblies of aphids reveal extensively rearranged autosomes and long-term conservation of the X chromosome. Mol Biol Evol. 2021;38 3:856-75. doi:10.1093/molbev/msaa246.

112. Wei P, Li Y, Lai D, Geng L, Liu C, Zhang J, et al. *Protaetia brevitarsis* larvae can feed on and convert spent mushroom substrate from *Auricularia auricula* and *Lentinula edodes* cultivation. Waste Management. 2020;114:234–9. doi:10.1016/j.wasman.2020.07.009.

113. Wang Q; Liu L; Zhang S; Wu H; Huang J (2022): Supporting data for "A chromosome-level genome assembly and intestinal transcriptome of Trypoxylus dichotomus (Coleoptera: Scarabaeidae) to understand its lignocellulose digestion ability" GigaScience Database. http://dx.doi.org/10.5524/102226.

Figure legends

Figure 1. Wood fiber degradation by larvae of *Trypoxylus dichotomus*. **a.** 3rd instar larva. **b.** Wood fiber structure in sawdust. **c.** Wood fiber structure after digestion of sawdust in larval excrement.

Figure 2. Genome assembly and assessment of *Trypoxylus dichotomus*. **a.** Accumulated graph of contig length. **b.** Hi-C heatmap showing 10 chromosomes (Chr1 to Chr10) arranged by length.

Figure 3. Circos graph of chromosome-level genome of *Trypoxylus dichotomus*, showing length of chromosomes, GC-content, density of protein-coding genes and repetitive elements (DNA/SINE/LINE/LTR). (Sliding window size = 100 kb)

Figure 4. a. Phylogenetic tree and statistics of orthologs. Left: Phylogenetic tree and divergence times of beetles based on 1,108 single-copy orthologs; branch values representing the number of expanded, contracted and rapidly evolving gene families (bold) respectively; color value scale representing divisions of geologic time, abbreviations standing for Silurian (S), Devonian (D), Carboniferous (C), Permian (P), Triassic (Tr), Jurassic (J), Cretaceous (K) and Tertiary (T). Right: statistics of orthologous genes among the 14 insect species; '1:1:1' representing shared single-copy genes, 'N:N:N' representing multi-copy genes shared by all species, 'Coleoptera' representing orthologs unique to Coleoptera, and 'Others' representing unclassified orthologs. **b.** Chromosome-level genome synteny between *Trypoxylus dichotomus* and *Tribolium castaneum*; 'TdChr' representing chromosomes of *T. dichotomus*, 'TcChr' representing chromosomes of *Tri. castaneum*.

Figure 5. Expanded gene families and functional enrichment. **a.** Top twenty significantly expanded gene families. **b.** GO enrichment of rapidly expanded gene families. **c.** KEGG enrichment of rapidly expanded gene families.

Figure 6. Sample correlation of intestinal gene expression patterns among four groups of *Trypoxylus dichotomus*. Each group consists of six replicates. **a.** Principal component analysis (PCA) diagram; circle indicates larva feeding sawdust, square indicates larva feeding mushroom-residue, green indicates midgut, red indicates hindgut. **b.** Pearson correlation coefficient (PCC) heatmap; colors and values indicate the relationship between paired samples (the darker the color and larger value mean the closer the relationship), value ≥ 0.8 shows the good repeatability. SM, midgut from sawdust; SH, hindgut from sawdust; MM, midgut from mushroom-residue; MH, hindgut from mushroom-residue.

Figure 7. Heatmaps of differentially expressed digestion-related genes among four groups of the rhinoceros beetle. Each group consists of six replicates. Colors indicate a higher (red) or lower (blue) gene expression in each sample for every gene, identified by the FPKM value. Gene expression clustering between midgut and hindgut from sawdust group (**a**) and mushroom-residue group (**b**). Gene expression clustering of midgut (**c**) and hindgut (**d**) between sawdust and mushroom-residue groups. SM, midgut from sawdust; SH, hindgut from sawdust; MM, midgut from mushroom-residue; MH, hindgut from mushroom-residue.

Figure1

Click here to access/download;Figure;Figure 1.tif

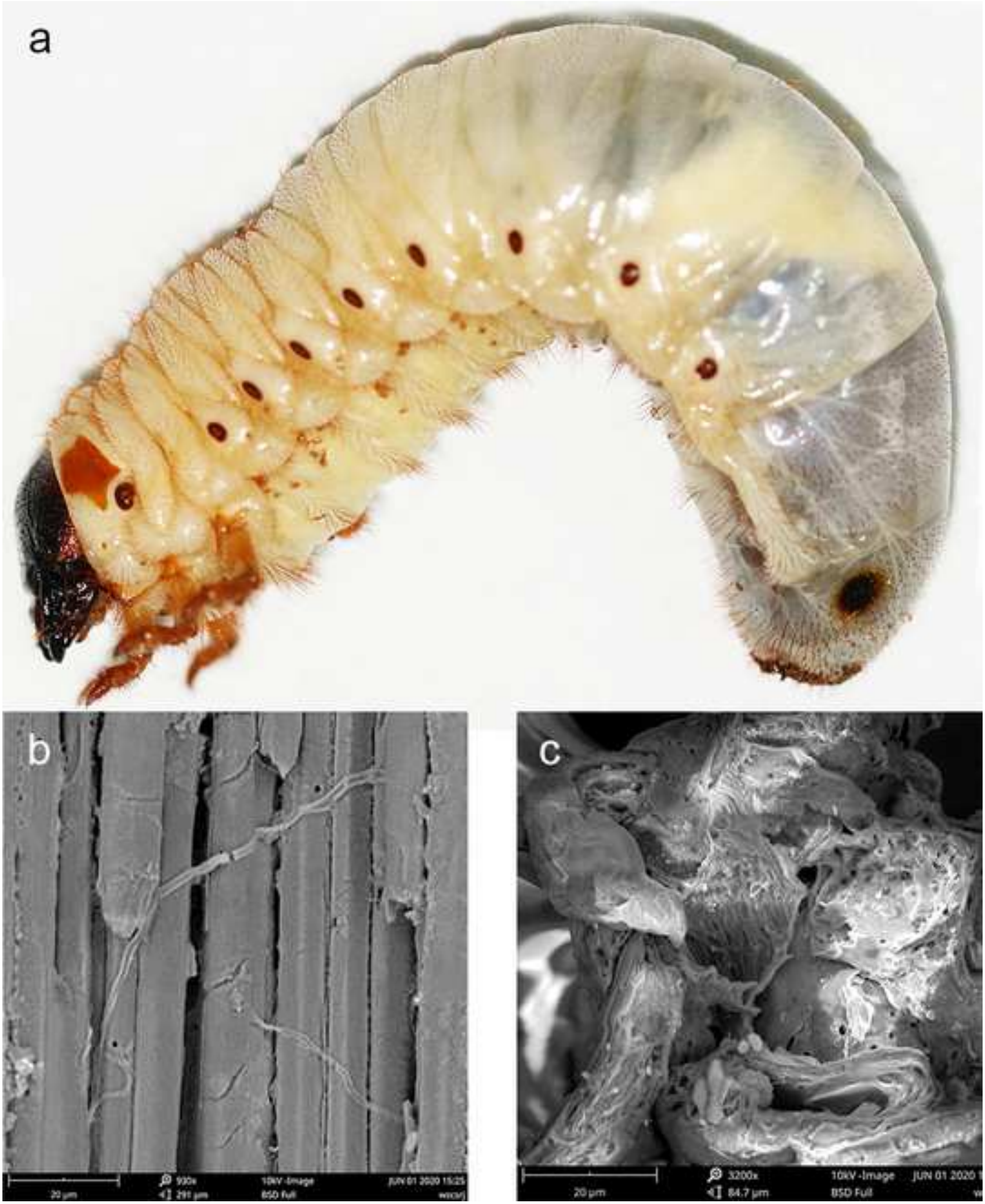

Figure2

Click here to access/download;Figure;Figure 2.tif

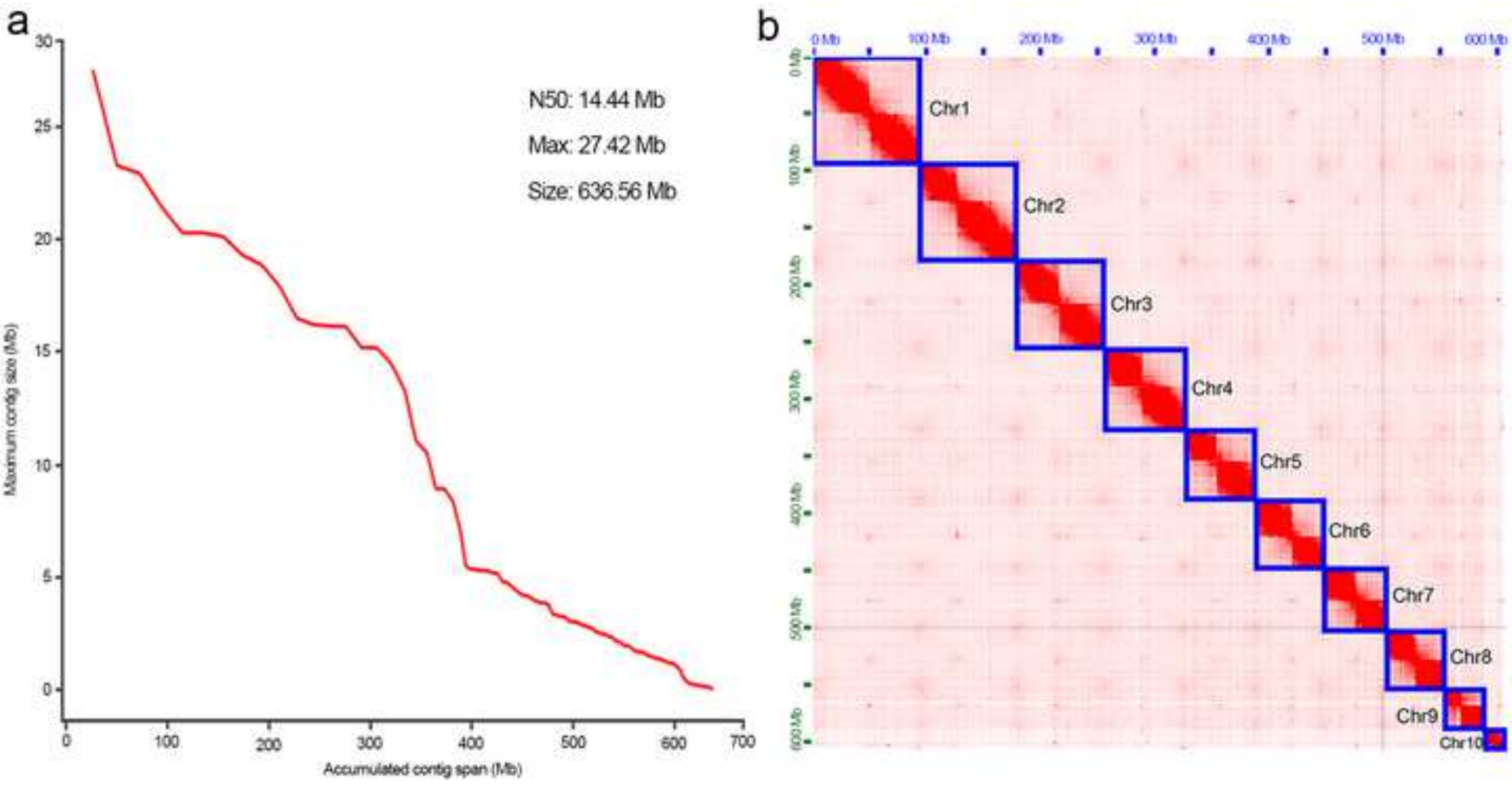

Figure3

Click here to access/download;Figure;Figure 3.tif

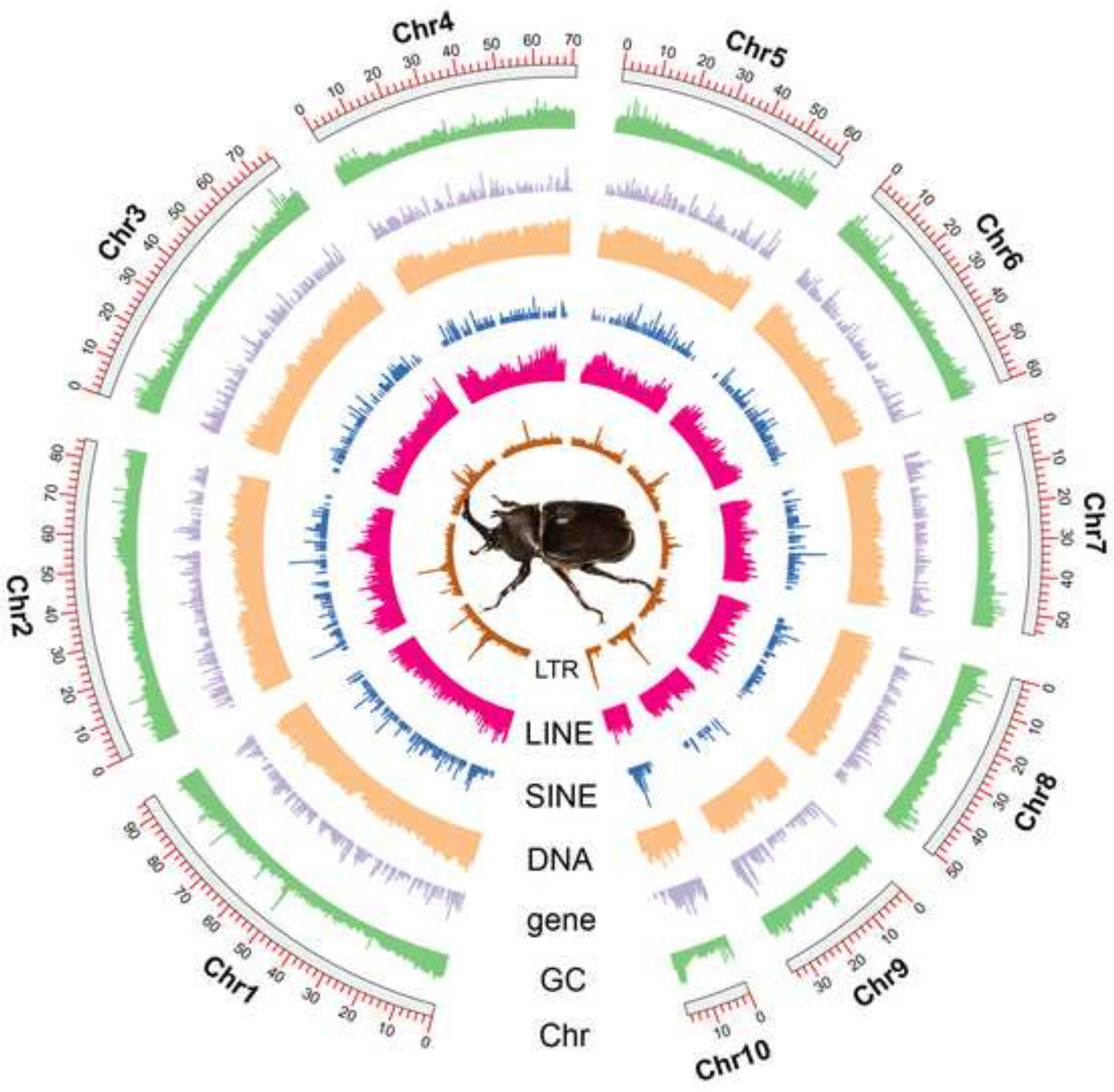

Figure4

Click here to access/download;Figure;Figure 4.tif

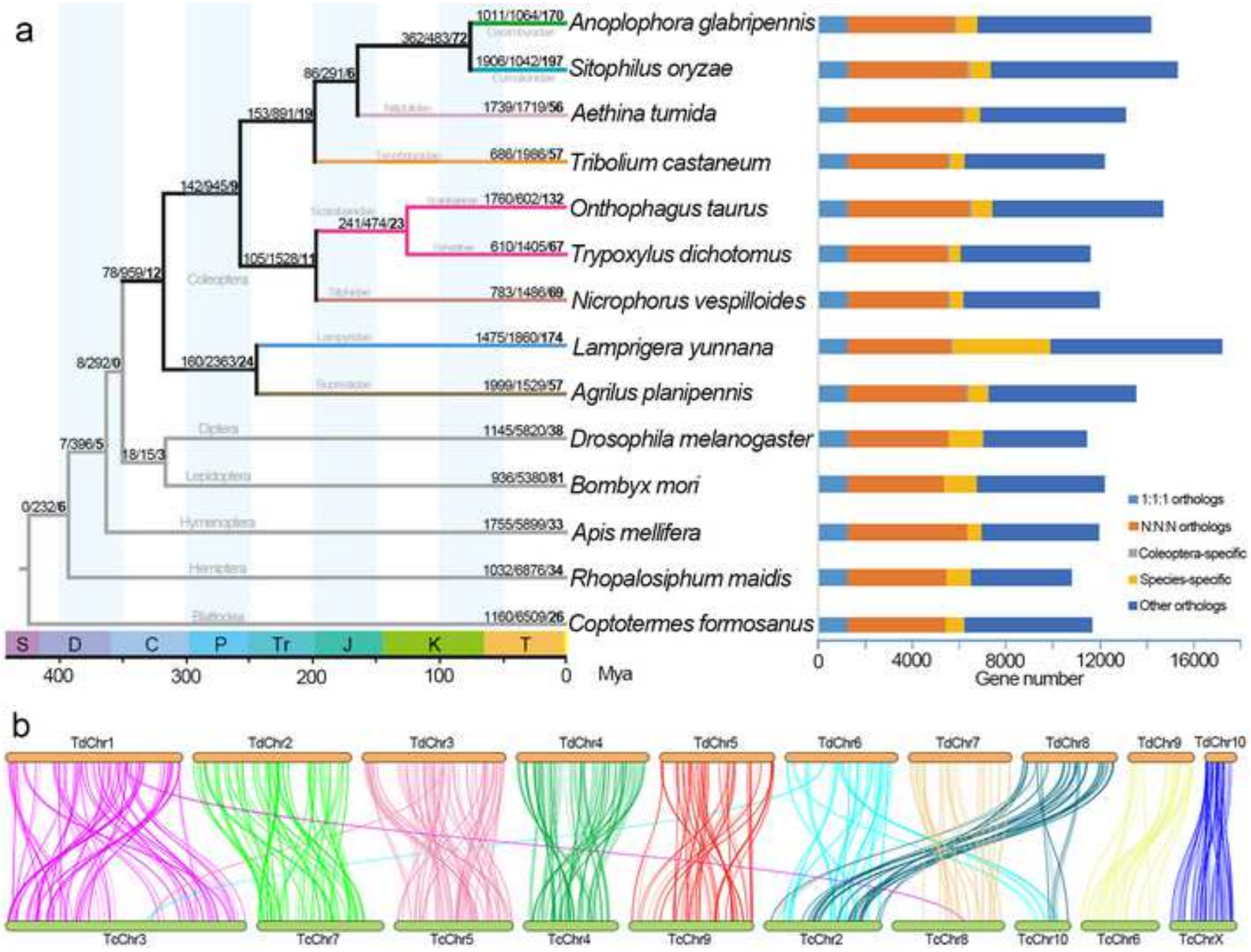

Figure5

Click here to access/download;Figure;Figure 5.tif

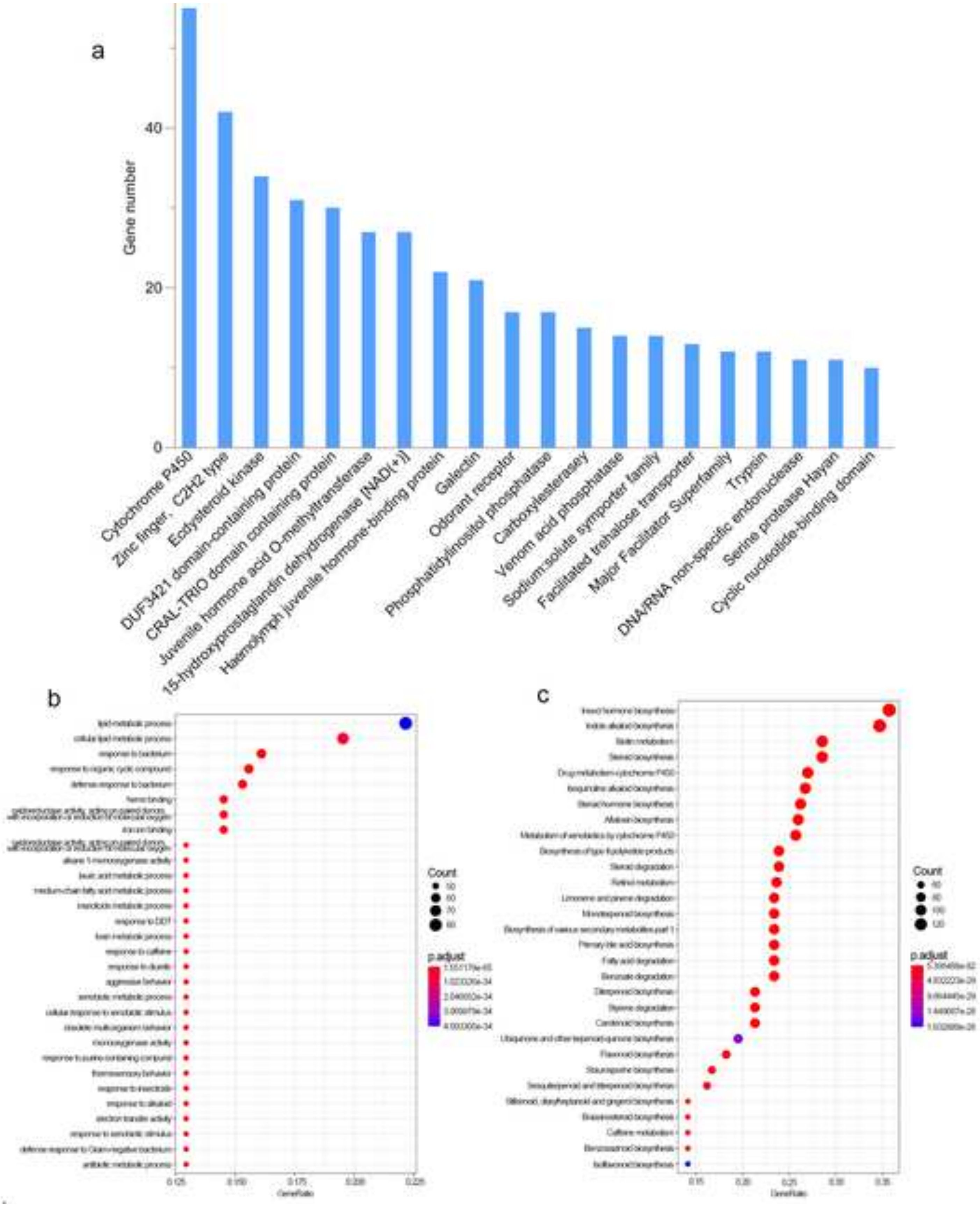

Figure6
Click here to access/download;Figure;Figure 6.tif

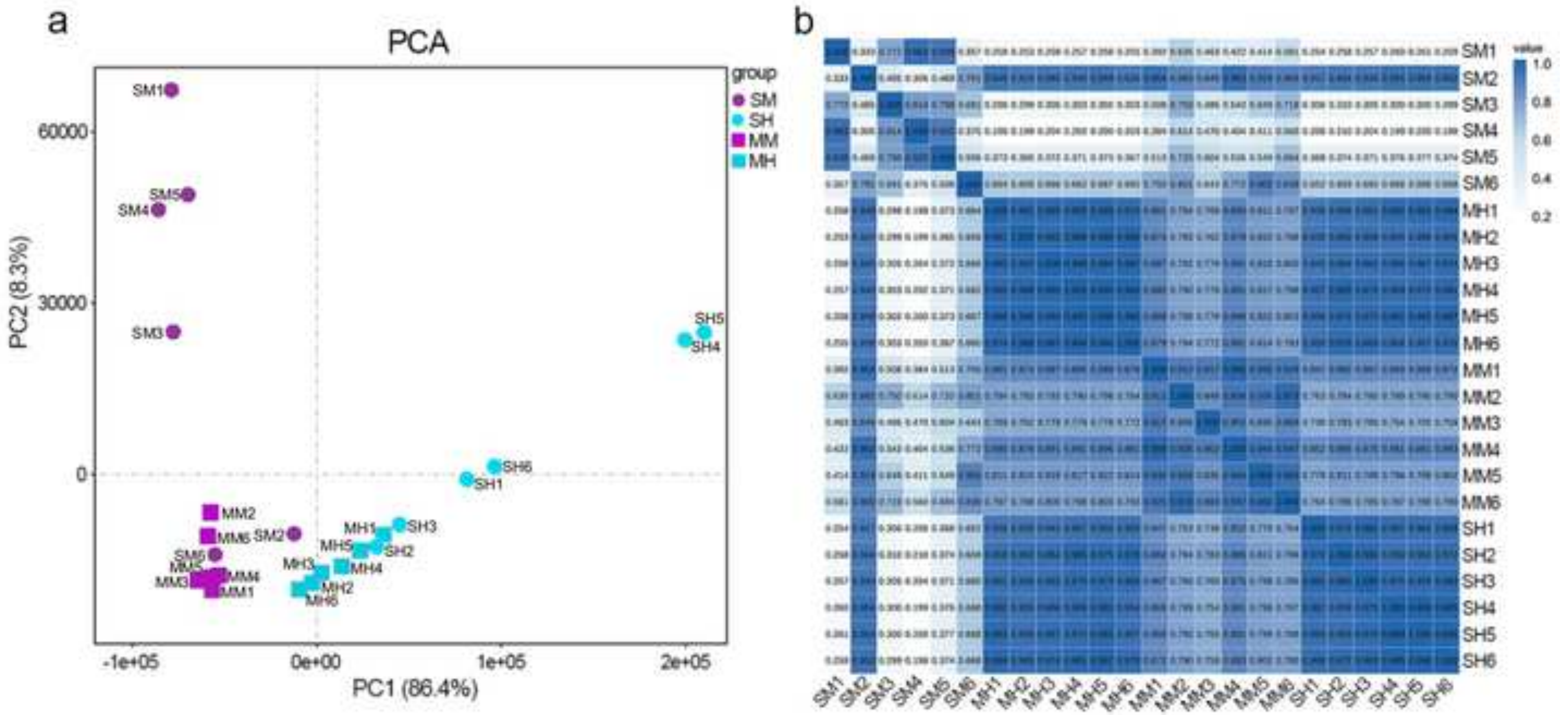

Figure7

Click here to access/download;Figure;Figure 7.tif

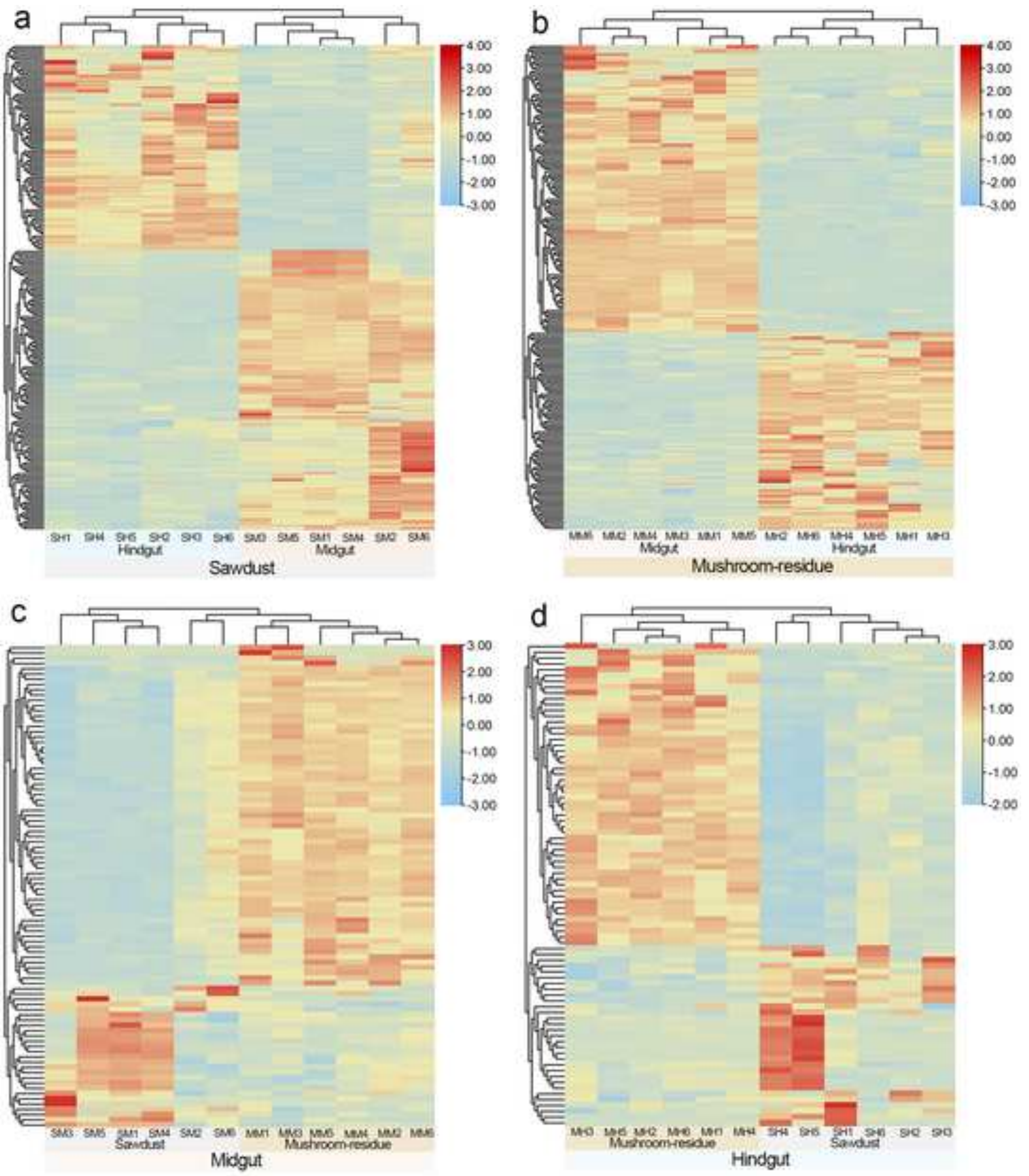

Figure S1-2

Click here to access/download
Supplementary Material
Figures S1-2.docx

Figure S legends

Click here to access/download
Supplementary Material
Supporting information.docx

Table S1,3-5,8&11

Click here to access/download
Supplementary Material
Table S1, 3-5,8&11.docx

Table S2

Click here to access/download
Supplementary Material
Table S2.xlsx

Table S6

Click here to access/download
Supplementary Material
Table S6.xls

Table S7

Click here to access/download
Supplementary Material
Table S7.xls

Table S9

Click here to access/download
Supplementary Material
Table S9.xlsx

Table S10

Click here to access/download
Supplementary Material
Table S10.xlsx

Table S12

Click here to access/download
Supplementary Material
Table S12.xlsx

Table S13

Click here to access/download
Supplementary Material
Table S13.xlsx

Table S14

Click here to access/download
Supplementary Material
Table S14.xlsx

Table S15

Click here to access/download
Supplementary Material
Table S15.xlsx

Table S16

Click here to access/download
Supplementary Material
Table S16.xlsx
