# Supplementary material for: A chromosome-level genome assembly and intestinal transcriptome of Trypoxylus dichotomus (Coleoptera: Scarabaeidae) to understand its lignocellulose digestion ability
Source: Gigascience. 2022 Jun 28;11:giac059. doi: 10.1093/gigascience/giac059 (PMC9239855; doi:10.1093/gigascience/giac059)
Supplement: giac059_Supplemental_Files [file giac059_supplemental_files.zip › Figures S1-2.docx]

**Supplementary information**

**A chromosome-level genome assembly and intestinal transcriptome of *Trypoxylus dichotomus* (Coleoptera: Scarabaeidae) to understand its lignocellulose digestion ability**

Qingyun Wang^a^, Liwei Liu^a,b^, Sujiong Zhang^c^, Hong Wu^a^, Junhao Huang^a^*

^a^ National Joint Local Engineering Laboratory for High-Efficient Preparation of Biopesticide, Zhejiang A&F University, 666 Wusu Street, Lin’an, Hangzhou, Zhejiang 311300, China

^b^ Zhejiang Museum of Natural History, No.6 West Lake Cultural Square, Hangzhou, Zhejiang 310014, China

^c^ Dapanshan Insect Institute of Zhejiang, Pan’an, Zhejiang, China

* Corresponding author: E-mail: huangjh@zafu.edu.cn, Tel: 86-571-63732758, Fax: 86-571-63740898


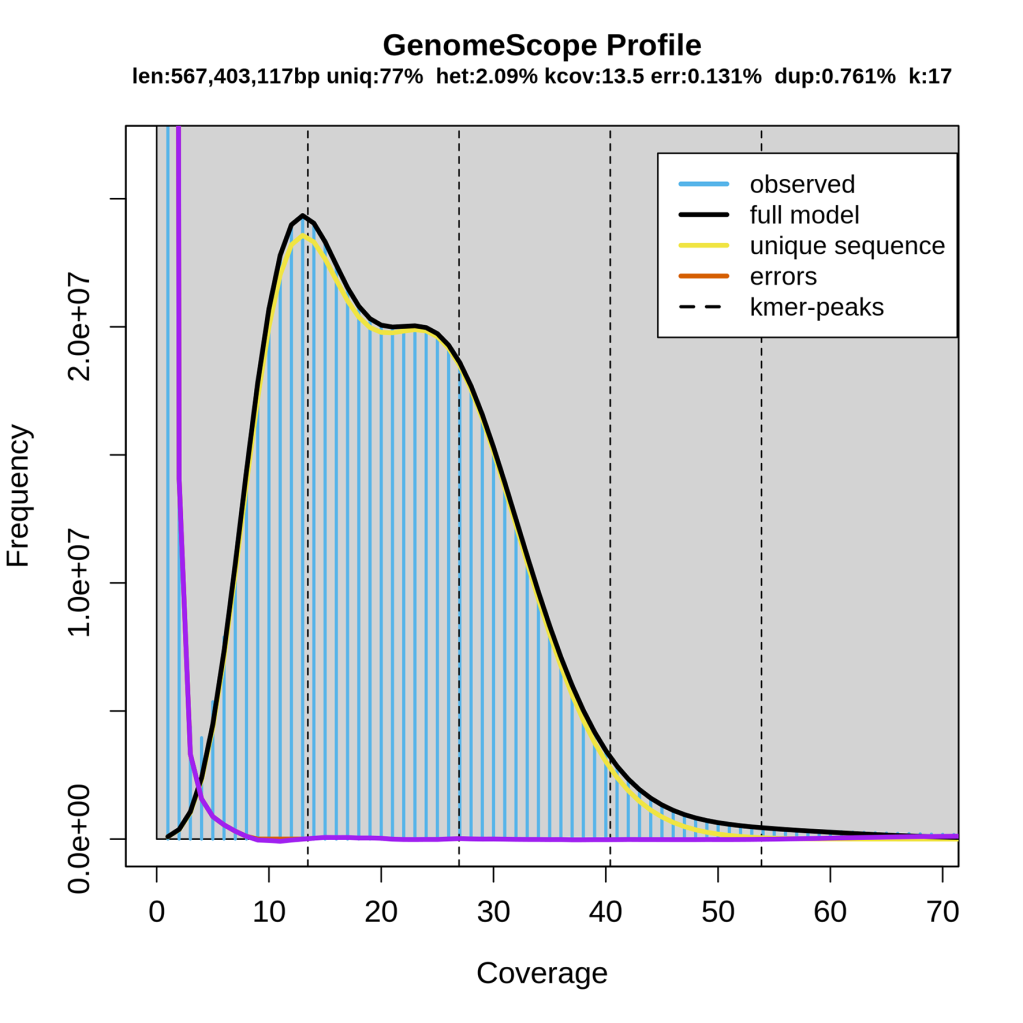


**Figure S1.** K-mer distribution curve


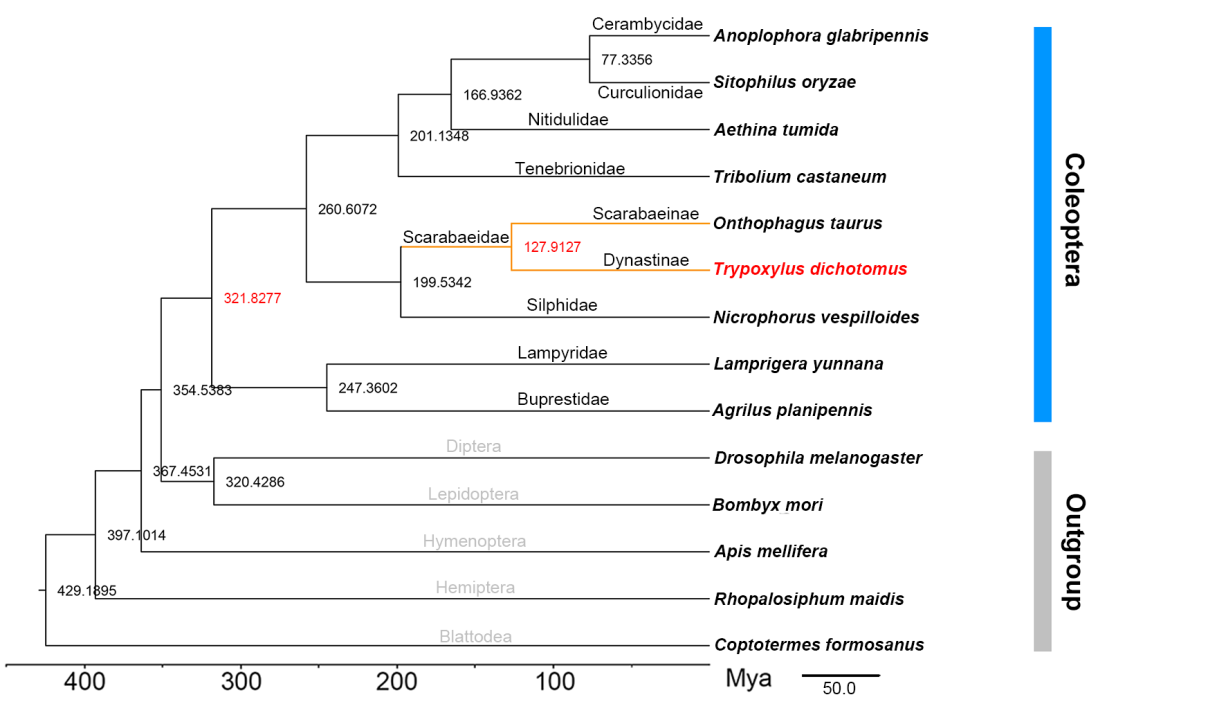


**Figure S2.** Phylogenetic tree and divergence times of beetles based on 1,260 single-copy orthologs. Node values representing divergence times.
