# Supplementary material for: A chromosome-level genome assembly and intestinal transcriptome of Trypoxylus dichotomus (Coleoptera: Scarabaeidae) to understand its lignocellulose digestion ability
Source: Gigascience. 2022 Jun 28;11:giac059. doi: 10.1093/gigascience/giac059 (PMC9239855; doi:10.1093/gigascience/giac059)
Supplement: giac059_Supplemental_Files [file giac059_supplemental_files.zip › Supporting information.docx]

**A chromosome-level genome assembly and intestinal transcriptome of *Trypoxylus dichotomus* (Coleoptera: Scarabaeidae) to understand its lignocellulose digestion ability**

Qingyun Wang^a^, Liwei Liu^a,b^, Sujiong Zhang^c^, Hong Wu^a^, Junhao Huang^a^*

^a^ National Joint Local Engineering Laboratory for High-Efficient Preparation of Biopesticide, Zhejiang A&F University, 666 Wusu Street, Lin’an, Hangzhou, Zhejiang 311300, China

^b^ Zhejiang Museum of Natural History, No.6 West Lake Cultural Square, Hangzhou, Zhejiang 310014, China

^c^ Dapanshan Insect Institute of Zhejiang, Pan’an, Zhejiang, China

* Corresponding author: E-mail: huangjh@zafu.edu.cn, Tel: 86-571-63732758, Fax: 86-571-63740898

**Supplemental information**

**Figure S1.** K-mer distribution curve.

**Figure S2.** Phylogenetic tree and divergence times of beetles based on 1,260 single-copy orthologs. Node values representing divergence times.

**Table S1.** Sample information of *Trypoxylus dichotomus*

**Table S2.** Transcriptome sequencing sample information.

**Table S3.** Genome sequencing data statistics.

**Table S4.** Genome estimation.

**Table S5.** Genome assembly and annotation statistics.

**Table S6.** Repeat annotation.

**Table S7.** Annotations of non-coding RNAs.

**Table S8.** Rapidly expanded gene families and functions.

**Table S9.** GO enrichment.

**Table S10.** KEGG enrichment.

**Table S11.** Ka/Ks values of forty-five rapidly expanded gene families (M0 model).

**Table S12.** Collinearity analysis between *Trypoxylus dichotomus* and *Tribolium castaneum*.

**Table S13.** PC values of all the samples based on the expression level of larval intestinal transcriptome.

**Table S14.** Pearson values of all samples based on the expression level of larval intestinal transcriptome.

**Table S15.** Expression of digestion-related genes in transcripts referring to KEGG pathways and GO terms.

**Table S16.** Comparison of differentially expressed digestion-related genes among different groups.
