# Supplementary material for: A chromosome-level genome assembly and intestinal transcriptome of Trypoxylus dichotomus (Coleoptera: Scarabaeidae) to understand its lignocellulose digestion ability
Source: Gigascience. 2022 Jun 28;11:giac059. doi: 10.1093/gigascience/giac059 (PMC9239855; doi:10.1093/gigascience/giac059)
Supplement: giac059_Supplemental_Files [file giac059_supplemental_files.zip › Table S1, 3-5,8&11.docx]

**Supplementary information**

**A chromosome-level genome assembly and intestinal transcriptome of *Trypoxylus dichotomus* (Coleoptera: Scarabaeidae) to understand its lignocellulose digestion ability**

Qingyun Wang^a^, Liwei Liu^a,b^, Sujiong Zhang^c^, Hong Wu^a^, Junhao Huang^a^*

^a^ National Joint Local Engineering Laboratory for High-Efficient Preparation of Biopesticide, Zhejiang A&F University, 666 Wusu Street, Lin’an, Hangzhou, Zhejiang 311300, China

^b^ Zhejiang Museum of Natural History, No.6 West Lake Cultural Square, Hangzhou, Zhejiang 310014, China

^c^ Dapanshan Insect Institute of Zhejiang, Pan’an, Zhejiang, China

* Corresponding author: E-mail: huangjh@zafu.edu.cn, Tel: 86-571-63732758, Fax: 86-571-63740898

**Table S1.** Sample information of *Trypoxylus dichotomus*

| **Sample name** | **Gender** | **Locality** | **Coordinate** | **Elevation** | **Material** | **Sequencing** | **Description** |
| --- | --- | --- | --- | --- | --- | --- | --- |
| WG1 | Female | Pan'an, Zhejiang | 28.94°N, 120.55°E | 800 m | thoracic muscle | Illumina (Survey), Nanopore | Female adult for genome sequencing and estimation |
| WG4 | Male | Pan'an, Zhejiang | 28.94°N, 120.55°E | 800 m | thoracic muscle | Illumina (RNA), Hi-C | Male adult for Hi-C sequencing and RNA-seq |

**Table S3.** Genome sequencing data statistics

| **Sample name** | **Category** | **GenBank accession** | **Total reads** | **Total bases** | **Mean length (bp)** | **N50 length (bp)** |
| --- | --- | --- | --- | --- | --- | --- |
| WG1 | Survey | SRR17320057 | 183,481,842 | 25,401,440,495 | 150 | / |
| WG1 | Nanopore | SRR17345266 | 4,305,705 | 72,671,895,372 | 16,878 | 24,539 |
| WG4 | RNA | SRR17325807 | 81,424,606 | 12,158,030,175 | 150 | / |
| WG4 | Hi-C | SRR17326714 | 552,598,450 | 82,671,820,512 | 150 | / |

**Table S4.** Genome estimation

| **Estimation** | **K-mer** | **Genome_size (bp)** | **Heterozygous ratio (%)** | **Repeat ratio (%)** |
| --- | --- | --- | --- | --- |
| FindGSE | 17 | 630,925,724 | - | 32.29 |
| GenomeScope | 17 | 567,403,117 | 2.09 | 22.99 |

**Table S5.** Genome assembly and annotation statistics

| **Category** | ***Trypoxylus dichotomus*** |
| --- | --- |
| **Genome assembly** |  |
| Assembly size (Mb) | 636.27 |
| Number of scaffolds/contigs | 417/693 |
| Longest scaffold/contig (Mb) | 94.63/24.92 |
| N50 scaffold/contig length (Mb) | 71.04/12.99 |
| GC (%) | 35.11 |
| Gaps (%) | 0.004 |
| BUSCO completeness (%) | 98.7 |
| **Gene annotation** |  |
| Protein-coding genes | 12,193 |
| Mean protein length (aa) | 579.9 |
| Mean gene length (bp) | 15150.33 |
| Exons/introns per gene | 7.91/6.71 |
| Exon (%) | 5.14 |
| Mean exon length | 339.23 |
| Intron (%) | 23.89 |
| Mean intron length | 1857.47 |
| BUSCO completeness (%) | 95.8 |

**Table S8.** Rapidly expanded gene families and functions

| **Rapidly expanded families** | **Genes** | **Functions** |
| --- | --- | --- |
| Cytochrome P450 | 55 | metabolic detoxification |
| Zinc finger, C2H2 type | 42 | unknown |
| Ecdysteroid kinase | 34 | detox |
| DUF3421 domain-containing protein | 31 | unknown |
| CRAL-TRIO domain containing protein | 30 | unknown |
| Juvenile hormone acid O-methyltransferase | 27 | juvenile hormone synthesis |
| 15-hydroxyprostaglandin dehydrogenase [NAD(+)] | 27 | inflammatory |
| Haemolymph juvenile hormone-binding protein | 22 | juvenile hormone regulation |
| Galectin | 21 | embryonic development/pathogen immunity |
| Odorant receptor | 17 | OR |
| Phosphatidylinositol phosphatase | 17 | cell proliferation/differentiation |
| Carboxylesterasey | 15 | metabolic detoxification |
| Venom acid phosphatase | 14 | toxoprotein |
| Sodium:solute symporter family | 14 | nerves/digestion |
| Facilitated trehalose transporter | 13 | glycometabolism |
| Major Facilitator Superfamily | 12 | transmembrane transport |
| Trypsin | 12 | digestion |
| DNA/RNA non-specific endonuclease | 11 | unknown |
| Serine protease Hayan | 11 | immunity |
| Cyclic nucleotide-binding domain | 10 | nerve conduction |
| Glucose dehydrogenase | 10 | unknown |
| Hsp70 protein | 10 | heat shock protein |
| Nucleoporin autopeptidase | 10 | nucleo-cytoplasmic transport |
| Arrestin | 9 | regulation of GRCR |
| Enoyl-(Acyl carrier protein) reductase | 9 | fatty acid synthesis |
| Gustatory receptor | 8 | GR |
| Aldo/keto reductase | 8 | detoxification |
| Collagen | 8 | collagen |
| Insect cuticle protein | 8 | cuticula |
| Serine protease snake | 8 | development |
| Trypsin/Low-density lipoprotein receptor domain class A | 8 | digestion |
| Prostaglandin reductase 1 | 8 | immune (anti-inflammatory) |
| Neutral alpha-glucosidase | 7 | sugar hydrolysis |
| Histone-lysine N-methyltransferase | 7 | meiosis |
| THAP domain-containing protein | 6 | unknown |
| Inducible metalloproteinase inhibitor protein | 6 | immunity (trypsin inhibitor) |
| Zonadhesin | 6 | trypsin inhibitors (reproductive related) |
| Endonuclease-reverse transcriptase | 3 | unknown |

**Table S11.** Ka/Ks values of forty-five rapidly expanded gene families (M0 model)

| **orthogroup** | **Omega (dN/dS or Ka/Ks)** |
| --- | --- |
| OG0000002 | 0.08999 |
| OG0000023 | 0.11640 |
| OG0000087 | 0.12921 |
| OG0000102 | 0.21758 |
| OG0000103 | 0.00760 |
| OG0000104 | 0.14775 |
| OG0000110 | 0.18820 |
| OG0000165 | 0.17977 |
| OG0000177 | 0.17059 |
| OG0000213 | 0.10134 |
| OG0000228 | 0.08402 |
| OG0000274 | 0.09308 |
| OG0000276 | 0.12458 |
| OG0000278 | 0.23154 |
| OG0000289 | 0.20047 |
| OG0000315 | 0.01814 |
| OG0000323 | 0.00682 |
| OG0000343 | 0.18035 |
| OG0000411** (immunity) | 0.46482 |
| OG0000444 | 0.21307 |
| OG0000515 | 0.10634 |
| OG0000618 | 0.17291 |
| OG0000625 | 0.08826 |
| OG0000686 | 0.29128 |
| OG0000742 | 0.09975 |
| OG0001117 | 0.25492 |
| OG0001265 | 0.33264 |
| OG0001456* (cell proliferation/differentiation) | 0.40511 |
| OG0001695 | 0.23023 |
| OG0002028 | 0.22158 |
| OG0002473 | 0.14621 |
| OG0004885 | 0.00776 |
| OG0007820 | 0.14887 |
| OG0008363 | 0.12741 |
| OG0008366 | 0.14462 |
| OG0008367 | 0.25987 |
| OG0008368 | 0.42622 |
| OG0008682 | 0.03566 |
| OG0008738 | 0.18878 |
| OG0009015* (heat shock protein) | 0.31409 |
| OG0009016** (nucleo-cytoplasmic transport) | 0.46641 |
| OG0009572 | 0.25896 |
| OG0009573 | 0.06430 |
| OG0009574 | 0.15127 |
| OG0009575 | 0.26693 |

Note: * and ** means positive selected gene families, ** stands for the posterior probability (PP) > 0.99, * stands for 0.99 > PP > 0.95.
