# Supplementary material for: A chromosome-level genome assembly and intestinal transcriptome of Trypoxylus dichotomus (Coleoptera: Scarabaeidae) to understand its lignocellulose digestion ability
Source: Gigascience. 2022 Jun 28;11:giac059. doi: 10.1093/gigascience/giac059 (PMC9239855; doi:10.1093/gigascience/giac059)
Supplement: giac059_Response_to_Reviewer_Comments_Original_Submission [file giac059_response_to_reviewer_comments_original_submission.pdf]

Author's Response To Reviewer Comments

Close

Dear editor,

Many thanks to you and the reviewers for your valuable comments. All of the suggestions and problems you raised have been considered and resolved seriously in the revised manuscript. In particular, the involved comparative analyses and methodological information in the original manuscript have been supplied with the detailed information as you recommended. Moreover, the reviewers' comments and questions are replied and answered in detail as follows.

Reply to Reviewer 1:
(1) Given the recent release of another assembly of this species (mentioned lines 328-329) which was generated using PacBio reads, the authors are missing out on an interesting comparison between data types (and as-is, I can't evaluate the truth of lines 526-527 that this is the first chromosome-scale assembly for the species). I think some comparative analyses would be quite interesting of these 2 raw assemblies, or at the very least more than a one-line mention that another assembly exists.
Reply: According to the reviewer's recommendation, the BUSCO assessment of the recently released genome assembly has been performed. The result shows that there are 99.7% BUSCO completeness, including 98.5% single-copy and 1.2% duplicated BUSCOs, in the recently released genome of Trypoxylus dichotomus. In addition, the comparison has been performed on the important assembly information (scaffold/contig N50 length, scaffold/contig number) between these two draft genome assemblies of this beetle.
Although there has been no chromosome-level assembly released for Trypoxylus dichotomus so far, the reviewer questioned whether it is the first chromosome-level assembly for this species, thus we have deleted the statement like "first" and "firstly" in the manuscript.
All of the above mentioned information has been updated and added in the present manuscript.

(2) Overall, I am not convinced by the mid vs. hindgut and mushroom vs. wood fiber differential expression analysis. This is both for lack of some methodological detail (pertaining the diets that larvae were reared on) and interpretation and resulting conclusion statements about particular parts of the gut having more digestive ability for wood vs. fungi. The latter could probably be addressed by toning down some of the statements of those results or at least more accurately describing patterns in the data.
Reply: The larvae rearing methods have been improved with more details added in. For instance, the statement on the food treatment and environmental temperature has been detailed, "The larvae were divided into two groups and reared with high-temperature sterilized sawdust and mushroom-residue (composed of wood fiber and fungal mycelia) at 20–25°C for two months, separately."
Furthermore, the problems on the differential expression analysis of intestinal digestion-related genes have been considered and resolved seriously. Firstly, the original comparative analysis and statement between midgut and hindgut were confirmed to be inappropriate. The proper statement should be "Thus, the digestion of lignocellulose in larvae may require more digestive enzymes in the midgut than that in the hindgut". Secondly, the proposal on the comparative analysis of food habits the reviewer raised has been adopted. The previous statement is modified as "These results suggest that digestion of mushroom-residue might require a greater digestive ability than that of sawdust for the larvae of T. dichotomus, which is probably due to the complex components of mushroom-residue, including not only wood fiber but also fungal mycelia". In addition, the statements of the other comparative analysis have also been improved according to the reviewer's suggestions.

(3) Finally, I have made several comments in the first few paragraphs concerning grammatical flow, sentence structure, and English considerations, but not throughout the rest of the study (although there were many English errors in the rest of the manuscript). The entire manuscript requires editing from a native English speaker before being fit for publication, as there are many unclear and incorrect statements stemming from grammatical/English mistakes.
Reply: The reviewer's suggestion is followed in the revised manuscript presently. The entire manuscript has been edited by a native English speaker, in which the previous grammatical/English mistakes have

been largely found out and corrected in the present version of manuscript.

SPECIFIC COMMENTS

(4) Line 45. "consists in almost all kinds" is a bit awkward. Perhaps "lignocellulosic biomass is found in all kinds"?
Reply: The reviewer's suggestion is adopted in the revised manuscript.

(5) Line 48. "protecting the" should be "protects".
Reply: This mistake has been corrected.

(6) Lines 49-51. The "Thus" beginning of this sentence isn't really following the point of the preceding sentence. Perhaps reordering the sentence to something like: "Recycling plant waste produced by human production is a noteworthy environmental issue."
Reply: Yes, it is now revised according to the reviewer's advice.

(7) Line 56. I suggest rewording this sentence to make the subject of the latter half of the sentence clearer (the subject of the first half is "lignocellulose decomposition", but in the second half it is just "lignocellulose").
Reply: This sentence has been revised as "Due to the complex structural and chemical mechanisms of lignocellulose, lignocellulose decomposition is not common among animals [8, 9], except for wood-feeding insects, such as termites, wood-feeding cockroaches, beetles and wood wasps [6, 7, 9-11]".

(8) Line 59. Should be "highly efficient".
Reply: It has been corrected.

(9) Line 60. Should be present tense. Same for the next sentence as well.
Reply: The reviewer's suggestion has been accepted in the revised version.

(10) Lines 63-64. 1) The termites did not receive research achievements. They were used as study systems. 2) Additionally, while comparison to termites makes biological sense, there is no mention of microbial-aided digestion in the manuscript. Is anything known about the microbiome of this beetle species as compared to termites? Or is their digestion ability fully the beetle's doing and not their gut microbiome?
Reply:
1) This sentence is modified as "which have achieved considerable research progresses in functional genomics and symbiotic intestinal microorganisms".
2) The reviewer's advice is followed. Comparison has not yet been made between microbiome of this beetle and termites. According to the recent microbiological experiments (not published), intestinal microorganism plays an important role in lignocellulose digestion as well.

(11) Line 67. I don't think "biodegradability" really fits here if the authors are referring to the ability to break down the substances from the point of view of the insects.
Reply: The word "biodegradability" has been replaced with the phrase "bio-degradation ability", which refers to the lignocellulose degradation ability of the xylophagous insects (except for termites).

(12) Line 73. It "can", not "could".
Reply: It has been corrected.

(13) Lines 109-110. Was there rationale for choosing female tissue for some data and male tissue for others?
Reply: We obtained a pair of newly emerged beetles of different genders before the experiments and sequencing. In order to meet the sequencing requirements, the muscle of female thoracic muscle was prepared for Illumina and Nanopore sequencing, while the male thoracic muscle was then chosen for Hi-C and RNA sequencing.

(14) Line 113. 1) What is the methodology for these rearing medias? Is there control to make sure they have similar microbial contents despite their different composition? 2) Is the mushroom residue just pure fungus? Or does that include wood in the media that has been decomposed by mycelia? 3) More description of these media would greatly improve what conclusions could be made for these samples.
4) Additionally, would these beetles be found feeding solely in fungi in the wild? Or would they always be consuming a mix of wood and fungi (along with other decomposing matter)? 5) Detailed description of

the biology of this species' feeding would help for readers who are not familiar with this beetle in the wild (such as myself, as a North American).
Reply:
1) Yes, all of the rearing medias were sterilized by high temperature steam.
2) No, it is not just fungus. The mushroom residue is composed of wood fiber and fungal mycelia.
3) The advice of “more description of these media” is followed in the revised manuscript. This sentence is modified as "The larvae were divided into two groups and reared with high-temperature sterilized sawdust and mushroom-residue (composed of wood fiber and fungal mycelia) at 20–25°C for two months, separately".
4) No, they usually feed on decaying wood and humus in the wild, which has also been mentioned in the 3rd paragraph of introduction section.
5) The reviewer’s proposal that adding detailed description of biology of this beetle's feeding is reasonable, but its feeding biology in the wild has been mentioned in the sections of “Introduction” and “Results and Discussion”.

(15) Line 115/Figure 1. 1) While it's nice to see that the wood fiber was digested, what's the rationale for providing these images as in-text figures as opposed to supplementary figures (as they don't seem terribly important to me). 2) Also, what was the methodology of taking these photos? Is this wood fiber from the guts of the larvae, or extra-orally digested fiber? What type of image are these (I assume scanning electron microscopy)? 3) Were there strategies employed to make sure there was no bias in selecting what tissue was imaged? (i.e., are these images fully representative of the entire amount of diet tissue, and not just exemplars representing that some of the wood fiber was digested but not necessarily all of it?) 4) No details are provided.
Reply:
1) Figure 1 indicates the states of wood fiber before (b) and after degradation (c), separately, which could reveal the digestion ability of lignocellulose by this beetle immediately. Hence, the preminary research result of figure 1 is important and meaningful for the following research results, and should be kept as in-text figure.
2) Figure 1a was taken by digital single lens reflex camera. Figure 1b and 1c were taken by the environment scanning electron microscope. Wood fiber before degradation (Figure 1b) was taken from sawdust, while wood fiber after degradation was taken from larval excrement after digestion of wood fiber.
3) Yes, figure 1c could represent the digestion of wood fiber by this beetle. In this study, six excrements samples were randomly selected and photographed, the wood fiber structures were all degraded into similar fragments after digestion by its larvae.
4) The sentence has been modified as "Observed by environment scanning electron microscope (ESEM), most of the wood fibers in sawdust and mushroom-residue were fully degraded into fragments after intake and digestion by the larvae".

(16) Line 127. What does "gene library construction" refer to?
Reply: It refers to the short-insert (350 bp) and large-insert (>20 kb) libraries for genome survey and sequencing, respectively. Hence, it is modified as “short-insert (350 bp) and large-insert (> 20 kb) libraries construction”.

(17) Line 135. What was this "certain concentration and volume" used?
Reply: The concentration and volume were 50 fmol and volume 24 μL, respectively.

(18) Line 155. What is meant by "double check"? Do you mean using NCBI's EGAP? Or just their standard quality control?
Reply: This was an inaccurate statement in the original manuscript, thus it is now replaced by “contamination detection”.

(19) Line 150-163. I am unclear on what the authors mean by "NGS data" here. The genome assembly of the ONT data seem appropriate, but then some additional NGS data was aligned to the reference around like 158? I am not following these methods. I assume this is just the TruSeq libraries used for polishing the ONT assembly, but this is never specified (or is it just used for the kmer genome size estimation but not for anything else?).
Reply: Yes, the “NGS data” means the second generation sequencing data from genome survey, which was used for polishing the ONT assembly here. We have added the phrase “of genome survey” after “Next-Generation Sequencing (NGS) data” in this sentence.

(20) Line 173. How were the libraries quantified?

Reply: The statement on the libraries quantification “Libraries were quantified by Qubit 3.0 fluorometry (Invitrogen).” has been added after the sentence “Genomic DNA was extracted using QIAGEN® Genomic kit for short-insert (350 bp) and large-insert (> 20 kb) libraries construction according to the manufacturer's instructions” in the revised manuscript.

(21) Line 232. "unreliable" is not a term used by BMGE. What exact BMGE commands were used?
Reply: The concrete parameter “(-m BLOSUM90 –h 0.4)” has been added behind “BMGE v1.12”.

(22) Table 1. Should read "NextDenovo".
Reply: It has been corrected.

(23) Line 387. What does "commendably recovered" mean?
Reply: It means the phylogenetic relationships of these 14 insect species are same as the previous phylogenomic studies mentioned earlier in the manuscript. Here, the phrase “commendably recovered” is revised as “well recovered” presently.

(24) Line 417. 1) This sentence does not make sense grammatically. 2) Additionally, based on the beetle genomes available, I would argue that this analysis doesn't address how "most beetles" operate (since you only sample 9 beetle species).
Reply:
1) This sentence is revised as "Most beetles were considered not to capitalize the significant ability of endogenous lignocellulose digestion, but this was not the case for T. dichotomus."
2) This view was cited from the published paper “Endogenous plant cell wall digestion: a key mechanism in insect evolution”, which indicated that most beetles lack the capacity for significant endogenous lignocellulose digestion.

(25) Figure 6a. Which samples in the PCA are SM2 and SM6? Additionally, I am red-green colorblind and have a somewhat tough time seeing the different colors in this plot.
Reply: The original colors have been changed with purple and blue. The symbols “SM2” and “SM6” have been marked in the Figure 6a, separately.

(26) Line 480. What is the rationale for this statement? I can see that perhaps there is more separation in ordination space of SM vs MM samples (excluding the outliers), but that separation is along PC2 (8%) compared to separation of arguably similar numbers of SH vs MH along PC1 (86%), so I would almost put more stock into slight differentiation along PC1 vs. broad differentiation along PC2. Overall, I am not convinced by statements throughout the study about midgut having stronger digestion ability than hindgut and the comparisons of wood vs. fungi as food material.
Reply: The reviewer’s view is accepted here. The original description was problematic, thus the section 3.5 has been reworded. The digestive ability of different gut tissues is not discussed for the weak evidence. According to the result of PCA analysis, the description and discussion focus only on the groups with same food habits (SM vs SH, MM vs MH) and groups with same gut tissue (SM vs MM, SH vs MH). For the comparisons and discussions in the other sections, we have also made modifications and improvements according to the reviewer’s suggestion.

(27) Lines 507-511. I am not following the logic here 100%. Presumably mushroom residue would be already broken down compared to wood fiber itself and require less digestion on the part of the beetle, but these results indicate more expression of digestion-related genes in the mushroom treatment. Perhaps the digestion of mushroom components themselves require the extra digestive ability, not just the lignocellulose?
Reply: What the reviewer proposed is reasonable. We have modified this sentence as “Taken together, more digestion-related genes were highly expressed in the mushroom-residue group than that in the sawdust group regardless of midgut or hindgut. These results suggest that digestion of mushroom-residue might require a greater digestive ability than that of sawdust for the larvae of T. dichotomus, which is probably due to the complex components of mushroom-residue, including not only wood fiber but also fungal mycelia”.

(28) Lines 536-538. Not technically true since those are not sister taxa. Their ancestors diverged from each other at that time.
Reply: This sentence has been revised as the reviewer suggested. In addition, the other sentences with similar expressions have also been modified with the phrase “the ancestor(s) of” added in.

Reply to Reviewer 2:
Major Comments
(1) Title. The genome is estimated here as 599 MB, but the assembly is 636 MB. That needs to be resolved before claiming a chromosome level assembly.
Reply: The consensus estimated genome size (599 Mb) was inferred from the genome estimation results of FindGSE (630.93 Mb) and GenomeScope (567.4 Mb), which could not represent the actual genome size of this beetle. Furthermore, our final genome assembly size (636 Mb) was close to the previous estimated size by FindGSE, indicating that the final genome size was reliable. Not only that, there were 606.8 Mb scaffolds covering 95.37% of the draft reference genome anchored onto 10 pseudo-chromosomes in the chromosome-level assembly, which showed the chromosome-level genome assembly of high quality. As mentioned above, this tile "A chromosome-level genome assembly and intestinal transcriptome of Trypoxylus dichotomus (Coleoptera: Scarabaeidae) to understand its lignocellulose digestion ability" is applicable for this manuscript.

(2) Abstract. How is the timing of the split between Td and O taurus is needed in the abstract.
Reply: The divergence time "120 Mya" between the ancestors of Trypoxylus dichotomus and Onthophagus taurus has been added in the abstract.

(3) L39. Beetles are not mentioned in the first paragraph. I realize that lignocellulose digestion might be an important aspect to this work, but the paper reports a beetle genome assembly. That should be the main focus. The start of the first paragraph is much better for a biological, not geological, audience.
Reply: What the reviewer suggested is reasonable to some extent. However, the first paragraph highlighted the significance of this study, and explained why we paid great attention to the lignocellulose digestion related genes of this beetle, which is important for this manuscript. Thus, the main content of this paragraph should be remained, but has been greatly simplified in the revised version according to the reviewer's comment.

(4) L222 - I was going to put this in the minor comments, but then I realized that it is actually a serious comment. All of the used genomic resources need citations. I know off the top of my head at least nine do. If we as a field do not cite reference for the resources that we use, how can we expect people in other fields to cite them. I assume the authors here want people to cite this work when using the resource here, so they should do the same for others.
Reply: According to the reviewer's advice, the genomes of all the involved insects are cited and added in the revised manuscript presently.

(5) L127. I assume this was done according to the manufacturer's instructions, but state that so the reader does not have to wonder.
Reply: Yes, this was done according to the manufacturer's instructions. The reviewer's suggestion on adding details of methods is adopted in the whole revised manuscript.

(6) L163. What any type of haplotype reduction used or tried?
Reply: Haplotype reduction was not used or tried in the GC depth analysis by using Minimap2 and Samtools.

(7) L194. HISAT2 - what paramters were used? What any QC done of the RNA-seq reads or the alignments? Again, more details are needed in this section to make it reasonably reproducible.
Reply: The parameter "--dta" is listed after the software "HISAT2 v2.2.0". Quality control of RNA-seq reads and alignments was performed with the program bbduk.sh (qtrim=rl trimq=20 minlen=20 ecco=t maxns=5 trimpolya=10 trimpolyg=10 trimpolyc=10) in the BBTools v38.82. All of the above information has been added in the new manuscript.

(8) L200. What QC of the gene models from MAKER was done?
Reply: Using MAKER, gene prediction was performed with three strategies, Ab initio, transcriptome and protein, respectively. In the integrated results of MAKER, the AED values (range from 0 and 1, with 0 denoting perfect agreement of the annotation) can be referenced to the quality of gene prediction. Furthermore, the parameters "min_protein=30, min_intron=20" were set for quality control.

(9) L209. Is eggNOG not a third method to assign gene functional information. How was the information QC and integrated?
Reply: Gene function was annotated with two strategies: 1) Gene function was predicted by searching the specialized database "UniProtKB"; 2) Protein conserved sequences and domains, Gene Ontology (GO), and pathways [Kyoto Encyclopedia of Genes and Genomes (KEGG), Reactome] were predicted by

searching the integrated databases "InterProScan and eggNOG". Although InterProScan and eggNOG are both integrated databases, they are usually different in KEGG pathways and GO terms. Thus, it's necessary to search these two databases at the same time. Applying these two strategies, we could get as much information of gene function annotation as possible. Furthermore, quality control are not needed for the above gene function information. The results of above two strategies were finally presented in the 3rd paragraph of section 3.3.

(10) L262. That is definitely not the proper citation for the Tc genome. That paper does not report a single nucleotide sequence.
Reply: The citation has been replaced with the proper one.

(11) L298. How does this suggest good or bad sample quality? Heterozygosity is a biological property, as is genome size.
Reply: This inaccurate statement has been corrected. The phrase "suggesting the good quality of the test sample" has been deleted.

(12) L298. Sequencing results need to be separated from genome size estimate results.
Reply: These results has been divided into two paragraphs as the reviewer suggested.

(13) L321. But 599 Mb was the consensus estimate you gave.
Reply: Despite using the same NGS data, the results of genome estimation were different between the methods of FindGSE (630.93 Mb) and GenomeScope (567.4 Mb). Thus, we provided the consensus estimated genome size (599 Mb) before ONT sequencing. However, our final genome assembly size was 636 Mb after analyzing ONT sequencing data, which was apparently close to the genome estimation from FindGSE. The original expression might not be appropriate, so we deleted the word "pretty" in the revised version.

(14) L356. 4% of the BUSCO genes are not represented in the gene set? What number is duplicated or fragmented? All of those should have been in each proteome used to annotate. Odd to lose so much in what seems such a complete assembly.
Reply: The fragmented genes account for 1% among all the genes in the BUSCO assessment, thus there were actually 96.8% genes predicted in the MAKER process.
The software MAKER predicted genes based on the pathway of "Ab initio", with another two pathways "transcriptome" and "protein" for the correction. Thus, the insufficient transcriptomic information might lead to the weak support for predicted genes. Furthermore, the larger size the genome, the worse the gene prediction in MAKER in general. Actually, this BUSCO completeness of gene prediction (95.8%) is relatively high among the other published insect genomes.

(15) L359. How many of the hits were to other scarabs? To other beetles? To other insects? The reader needs more information here better assess the quality of the annotation.
Reply: The table 2 "Gene hits between Trypoxylus dichotomus and another six insects" has been added in the revised manuscript, which shows the gene hit number to the other involved insects.

(16) L377. How many beetle only orthogroups were there?
Reply: There are 12,658 orthogroups only to beetle.

(17) L413. Why is immunity being seen with the GO analysis for the gene families important? What were the contracted families and their enriched GO terms, if any?
Reply: There are two gene families involved immunity (Galectin and Serine protease Hayan) ranked in the first 20 rapidly expanded gene families, thus it was in the GO analysis. GO and KEGG enrichments has been appended as supplementary files (Table S9 &10) in the revised manuscript, which provides the detailed information for enriched gene families.

(18) L466. I fundamentally disagree with that statement. There a large scatter in hind and mid-gut samples along the two axes. Hind and mid definitely separate, but little consistent pattern after that.
Reply: According to the reviewer's opinion, this sentence has been revised as "By PCA analysis (Fig. 6a), we showed that samples from the same group were mainly aggregated together, except for four outliers (SM2, SM6, SH2 and SH3) in the midgut and hindgut of sawdust feeding beetles". Furthermore, the permutational multivariate analysis of variance (PERMANOVA) has been added in this manuscript, which indicates that there are significant differences among the pairwise comparisons of groups separated by gut tissues and food habits (SM vs SH, MM vs MH, SM vs MM and SH vs MH) (Table 4).

(19) Section 3.6. I have great difficulty understanding this section. 1) What was the comparison treatment? 2) So there are 423 DEGs in total? That seems like very few genes given the complete shift of diet, esp from native to a manipulated diet. That from two separate tissues too. 3) These sections are very, very difficult to get through and interpret.
Reply:
1) There are four groups separated by gut tissues (midgut and hindgut) and food habits (sawdust and mushroom-residue) in the comparison treatment. The first two groups are midgut and hindgut treatments from the same food habit (SM vs SH and MM vs MH), separately. The other two groups are sawdust and mushroom-residue treatments from the same gut tissue (SM vs MM and SH vs MH), separately.
2) The differentially expressed genes (DEGs) are not 423 in total. As mentioned in this manuscript, there are 222, 231, 92 and 83 DEGs in the groups of "SM vs SH", "MM vs MH", "SM vs MM" and "SH vs MH", respectively.
Mushroom-residue usually contains fungal mycelia, but it is mainly composed of partially decayed wood. Thus, it shares a high proportion of similar component with sawdust, which makes the differences between these two rearing foods are not as big as they look. For this reason, there were not too many DEGs between the groups of different rearing foods from the same gut tissue.
3) The original statement did have some inaccuracies, which makes certain content in the above-mentioned section confusing. According to the first reviewer's recommendation, these problems and mistakes have been resolved and corrected, with the statements being greatly improved in the updated section.

(20) L500. 1) Much of this is discussion, not results. 2) However, DEGs being different between sawdust and mushrooms is not direct evidence the beetles are better at digesting mushrooms than sawdust.
Reply: This comment is considered to be part of the former one.
1) This paragraph is in the third part "Results and Discussion", so it's not a problem for this manuscript.
2) As mentioned in the former comment, this problem has been resolved. We have modified the statement as "These results suggest that digestion of mushroom-residue might require a greater digestive ability than that of sawdust for the larvae of T. dichotomus, which is probably due to the complex components of mushroom-residue, including not only wood fiber but also fungal mycelia".

(21) L512-522. This is not a result.
Reply: Yes, this is a discussion. All of the results and discussion are in the same section of "Results and Discussion".

Minor Comments
(22) L25. There is no evidence provided that supports statement directly. I think it is likely, but the language needs to be moderated.
Reply: This sentence has been transposed into a more appropriate statement, i.e. "By gene family evolution analysis, we found 67 rapidly evolving gene families, among which two digestive gene families (encoding Trypsin and Enoyl-(Acyl carrier protein) reductase) have experienced significant expansion, indicating that they may contribute to the high degradation efficiency of lignocellulose in T. dichotomus".

(23) L29. Need a comma before respectively.
Reply: The comma has been added before "respectively".

(24) Keywords. Put in alphabetical order.
Reply: These keywords have been rearranged in alphabetical order.

(25) L63. "receiving preeminent research achievements" is very odd phrasing.
Reply: This phrase is now modified as "which have achieved considerable research progresses in functional genomics and symbiotic intestinal microorganisms".

(26) L73. Can, not could. It still has this ability as a species.
Reply: It has been revised in the new version.

(27) L113. Where did the sawdust and mushrooms come from? What was the temperature? Far more detail is needed here for repeatability.
Reply: The details on larvae rearing materials and methods have been added in the revised manuscripts. For instance, this sentence is now modified as "The larvae were divided into two groups and reared with high-temperature sterilized sawdust and mushroom-residue (composed of wood fiber and fungal mycelia) at 20–25°C for two months, separately".

(28) L114. Is this needed? Was this in doubt?
Reply: Yes, the figure 1 reflects the wood fiber structure after digestion by this beetle, which provides the visual evidence for its digestion ability of lignocellulosic biomass. In order to make the statement more accurate and detailed, this sentence has been revised as "Observed by environment scanning electron microscope (ESEM), most of the wood fibers in sawdust and mushroom-residue were fully degraded into fragments after intake and digestion by the larvae (Fig. 1)".

(29) L130. What quality filer was used? What program? What parameters? Or were these just the QC of the Illumina machine. What machine was used?
Reply: Quality control of raw reads was performed using fastp (v.0.20.0) preprocessor (set to default parameters) to remove low quality reads, adapters, and reads containing poly-N. The low-quality reads were filtered under the following conditions: 1) reads with ≥ 10% unidentified nucleotides (N); 2) reads with > 10 nucleotides aligned to the adapter, allowing ≤ 10% mismatch; 3) reads with >50% bases having Phred quality < 5; 4) Removing putative PCR duplicates generated by PCR amplification in the library construction process (read 1 and read 2 of two paired-end reads that were completely identical).

(30) L136. What chemistry and chip?
Reply: The chip is "FLO-PRO002", which has been added in the new version.

(31) L137-142. 1) How were the sample homogenized? 2) What version of the kits for TruSeq? 3) What version of chemisty for the Novaseq? 4) Where? What was the target insert size? 5) How did you QC the RNA and the gDNA? Many needed details are missing to make the work reasonably reproducible. Nothing is mentioned out the Hi-C sequencing mentioned in the previous paragraph.
Reply:
1) Total RNA was extracted from the thoracic muscle, and then qualified and quantified as follows: (1) RNA purity and concentration were then examined using NanoDrop 2000; (2) RNA integrity and quantity were measured using the Agilent 2100 system.
2) TruSeq RNA Library Preparation Kit v2.
3) The library preparations were sequenced on an Illumina Novaseq platform to generate 150 bp paired-end reads, according to the manufacturer's instructions.
4) The target insert size was 350 bp in RNA sequencing library.
5) The quality of raw reads of RNA and gDNA were controlled by fastp to remove reads containing adapters and low quality bases ("N">10%, Q-value≤20).

(32) L145. I assume that all other parameters were default, but do state that.
Reply: Yes, the other parameters were default in this program. These information has been added in the revised manuscript.

(33) L165. 1) How was cross-linking started? 2) Using what basic protocol? Provide the citation. 3) What library construction kit was used? 4) What chemistry for the NovaSeq?
Reply:
1) The cross-linking was started by the process, "quick-freezing tissues of T. dichotomus were vacuum infiltrated in nuclei isolation buffer supplemented with 2% formaldehyde".
2) The protocol has been provided in this paragraph, which is supplied with citation in the new version.
3) The Hi-C library construction kit wasn't used in this study, but the detailed process of library preparation has been provided in the revised manuscript.
4) The Hi-C libraries were quantified and sequenced on an Illumina Novaseq platform according to the manufacturer's instructions.

(34) L230. Where all these program used with default settings where no mentioned otherwise? This paragraph has a good level of detail. That level needs to be replicated for each section in the methods.
Reply: The reviewer's recommendation is accepted here, and the other involved methods have been detailed in the revised manuscript.

(35) L250. I do not understand what "Natural selection" means here.
Reply: This sentence has been replaced by "45 rapidly expanded gene families were further selected and analyzed to understand the evolution of expanded gene families".

(36) L259. Collinearity seems an odd word to me. I have never seen it used in this context. I am more familiar with this concept as "synteny."
Reply: The reviewer's suggestion was showed to be reasonable. Compared to the word "Collinearity",

“Synteny” is more appropriate in this manuscript. Thus, “collinearity” has been replaced by “synteny” in the revised version.

(37) L270. What quality threshold were used?
Reply: The detailed parameter “”N”>10%, Q-value≤20” is placed at the end of this sentence.

(38) L271. What rRNA database? Please provide the citation.
Reply: The rRNA database refers to the rRNAs annotated in the genome of this beetle, thus this sentence is revised as “…rRNA database of T. dichotomus with Bowtie2”.

(39) L275. How is a transcription region defined? Are those supposed to be genes? What gene set was used. The one from the genome annotation above?
Reply: The transcription region refers to the gene assembled by rRNA mapped reads. For ease of understanding, the sentence “The mapped reads of each sample were assembled by StringTie in a reference-based approach" has been added at the beginning of this paragraph.

(40) L279. 1) What was the model for differential expression? Tissue by diet, I assume? Please tell the reader. 2) Why is edgeR used? 3) What what to samples? What dispersion estimator was used for DESeq2? Why were DEGs filtered for KEGG and GO terms? What does that actually mean? 4) Was GO or KEGG term enrichment analysis not done?
Reply:
1) This sentence is revised as “Differential gene expression analysis was performed by DESeq2 [95] software with shrinkage estimator for dispersion between different gut tissues from the same diet, or same gut tissues from different diets”.
2) The software “edgeR” was not used in this study, it is deleted in the new version.
3) It was performed to filter out the digestion-related genes based on KEGG pathways and GO terms.
4) The intestinal transcriptome analysis aimed to understand the influences of gut segments and diets on the digestion-related genes of this beetle, thus GO and KEGG term enrichment analysis were not necessarily for this study.

(41) L295. Ratio? Ratio to what?
Reply: As mentioned earlier, the “ratio”refers to “repeat ratio”.

(42) Table 1. Is that actually NextDeNovo in the first line?
Reply: Yes, it is actually NextDeNovo, the misspelling has been corrected.

(43) L323. What is sex determination system of this beele? XO, XY?
Reply: According to the previous chromosomal studies on this species and its closely related beetles, the sex determination system was confirmed to be XY karotype for this beetle. Furthermore, it has the same number of chromosomes with another related species Tribolium castaneum who has the same chromosome karotype. Thus, chromosome 10 (TdChr10) in the sequenced female individual was considered to be X chromosome in the later section of “Synteny”.

(44) L332. Clearest example of what?
Reply: This sentence has been revised as “The clearest example of genomic difference was found in its closest relative species from the same subfamily, Onthophagus taurus, with a much smaller genome size of 267.08 Mb (Bioproject: PRJNA419349)”.

(45) L351. MAKER produced a consensus set. Not really predicted.
Reply: The word “predicted” is replaced by “generated”.

(46) L352. What is a peptide chain?
Reply: It is not proper here, which has been deleted presently.

(47) L393. I assume the families that the GO analyses are to are the ones specific to Td?
Reply: Yes, GO analyses were targeted at these rapidly expanded gene families.

(48) L417. This paragraph is discussion, not results.
Reply: Yes, the results and discussion are put together in this manuscript. Thus, it was not a problem.

(49) L449. Discussion, not results. Same for L473, 480.
Reply: This is the similar issue as the former comment. These statements are put in the section of

“Results and Discussion” as well.

(50) L486. Methods, not results.
Reply: This problem has been resolved. The first half sentence has been moved to the section of “Materials and Methods”.

Sincerely,
Junhao

Close
