# Supplementary material for: A chromosome-level genome assembly and intestinal transcriptome of Trypoxylus dichotomus (Coleoptera: Scarabaeidae) to understand its lignocellulose digestion ability
Source: Gigascience. 2022 Jun 28;11:giac059. doi: 10.1093/gigascience/giac059 (PMC9239855; doi:10.1093/gigascience/giac059)
Supplement: giac059_Response_to_Reviewer_Comments_Revision_1 [file giac059_response_to_reviewer_comments_revision_1.pdf]

Author's Response To Reviewer Comments

Close

Dear editor and reviewers,

Many thanks to you for the valuable comments. All of the suggestions and problems you raised have been reconsidered ar
the "Response 1" has been rewritten and attached below. The corresponding changes have been made in the revised man
the paid-for-service editing company (Charlesworth). We hope that the revised manuscript will meet the requirements of t
manuscript.

Response 1 to the manuscript GIGA-D-21-00415

Response to Reviewer 1:
(1) Given the recent release of another assembly of this species (mentioned lines 328-329) which was generated using Pa
between data types (and as-is, I can't evaluate the truth of lines 526-527 that this is the first chromosome-scale assembl
interesting of these 2 raw assemblies, or at the very least more than a one-line mention that another assembly exists.
Authors response: According to the reviewer's recommendation, the BUSCO assessment of the recently released genome
BUSCO completeness, including 98.5% single-copy and 1.2% duplicated BUSCOs, in the recently released genome of Tryp
important assembly information (scaffold/contig N50 length, scaffold/contig number) between these two draft genome ass
following sentences highlighted in the lines 360-366.
"Its BUSCO assessment (n = 1,367) identified 1,363 (99.7%) complete BUSCOs, comprised of 1,347 (98.5%) single-copy
was smaller than that of the released genome assembly, probably due to the scaffold assembly level we used. Furthermor
smaller scaffold number (414) than that of the released one."
Besides, the original incorrect description of the recently released genome assembly, i.e., "scaffold N50 length of 7.93 Mb
7.93 Mb; contig number of 2,347" in the lines 359.
Response to the above-mentioned comment "I can't evaluate the truth of lines 526-527 that this is the first chromosome-
level genome assembly released for Trypoxylus dichotomus so far, the reviewer questioned whether or not it is the first ch
scientifically credible, we have deleted the words "first" and "firstly" in the present statement of chromosome-level genom

(2) Overall, I am not convinced by the mid vs. hindgut and mushroom vs. wood fiber differential expression analysis. This
larvae were reared on) and interpretation and resulting conclusion statements about particular parts of the gut having mo
addressed by toning down some of the statements of those results or at least more accurately describing patterns in the c
Authors response: The larvae rearing methods have been detailed in the revised manuscript. The updated statement is "T
temperature sterilized sawdust and mushroom-residue (composed of wood fiber and fungal mycelia) at 20–25°C and 50–6
Furthermore, the statements about the results and discussion of comparative transcriptome analyses of larval gut have be
statement of comparative analyses between midgut and hindgut from different diets has been amended as "These results
midgut than the hindgut of larvae, regardless of food habit. Thus, the digestion of lignocellulose in larvae may require mo
Secondly, the original statement of comparative analyses between sawdust and mushroom-residue groups from different
mushroom-residue might require a greater digestive ability than that of sawdust for the larvae of T. dichotomus, which is
not only wood fiber but also fungal mycelia" (lines 555-559).

(3) Finally, I have made several comments in the first few paragraphs concerning grammatical flow, sentence structure, a
(although there were many English errors in the rest of the manuscript). The entire manuscript requires editing from a na
unclear and incorrect statements stemming from grammatical/English mistakes.
Authors response: The reviewer's suggestion is followed in the revised manuscript presently. The entire manuscript has be
grammatical/English mistakes have been largely found out and corrected in the present version of manuscript.

SPECIFIC COMMENTS

(4) Line 45. "consists in almost all kinds" is a bit awkward. Perhaps "lignocellulosic biomass is found in all kinds"?
Authors response: The reviewer's suggestion is accepted in the revised manuscript. The original sentence has been modifi
important route of carbon fixation, lignocellulosic biomass is found in all kinds of living and dead plants" (lines 42-43).

(5) Line 48. "protecting the" should be "protects".
Authors response: Yes, the original sentence has been modified as "……, which form a complex cross-linked and recalcitra

microorganisms or enzymes [2, 3]" (lines 45-46).

(6) Lines 49-51. The "Thus" beginning of this sentence isn't really following the point of the preceding sentence. Perhaps r
by human production is a noteworthy environmental issue."
Authors response: Yes, the original description is problematic. In order to make the statement more clear and specific, the
plant waste is usually done via landfill or incineration, which causes serious environmental pollution and health problems [
noteworthy environmental issue [5]" (lines 46-50). Moreover, the corresponding reference [4] "Shah SN, Mo KH, Yap SP,
incorporating waste materials: A review. Resources, Conservation and Recycling. 2021;164:105103" has been added in "F

(7) Line 56. I suggest rewording this sentence to make the subject of the latter half of the sentence clearer (the subject o
just "lignocellulose").
Authors response: The original description, "Lignocellulose decomposition is not a common trait among animals because o
14], but wood-feeding insects, such as termites, wood-feeding cockroaches, beetles and wood wasps capitalize a high-effi
to the complex structural and chemical mechanisms of lignocellulose, lignocellulose decomposition is not common among
feeding cockroaches, beetles, and wood wasps [7, 8, 10-12]" (lines 54-57).

(8) Line 59. Should be "highly efficient".
Authors response: The original statement, "……, but wood-feeding insects, such as termites, wood-feeding cockroaches, be
lignocellulosic biomass", has been modified as "…… except for wood-feeding insects such as termites, wood-feeding cockro

(9) Line 60. Should be present tense. Same for the next sentence as well.
Authors response: Yes, these sentences should be in the present tense. This sentence has been amended as "These insect
"Among them, xylophagous termites are the most well-known ……" (lines 59-60).

(10) Lines 63-64. 1) The termites did not receive research achievements. They were used as study systems. 2) Additional
of microbial-aided digestion in the manuscript. Is anything known about the microbiome of this beetle species as compare
their gut microbiome?
Authors response:
1) The reviewer's warning is reasonable. The original description has been modified as "Among them, xylophagous termite
studied in detail including in terms of functional genomics and symbiotic intestinal microorganisms [14-16]" (lines 59-62).
2) Yes, comparisons have not yet been made on the microbial-aided digestion between Trypoxylus dichotomus and termit
play on the digestion ability has not been reported, the recent unpublished research shows that intestinal microorganisms

(11) Line 67. I don't think "biodegradability" really fits here if the authors are referring to the ability to break down the su
Authors response: The word "biodegradability" has been replaced with the phrase "bio-degradation ability" (line 64), whic
(except for termites).

(12) Line 73. It "can", not "could".
Authors response: Yes, it refers to the larval ability of Trypoxylus dichotomus, so the word "could" has been replaced by "

(13) Lines 109-110. Was there rationale for choosing female tissue for some data and male tissue for others?
Authors response: Yes, the rationale for choosing female tissue for some data and male tissue for others are as follows:
1) The sex determination system of Trypoxylus dichotomus is XY, in which the Y chromosome is much smaller than X chro
2) There were only a pair of newly emerged beetles of different genders obtained for genome sequencing.
Thus, to meet the sequencing requirements, the muscle of female thoracic muscle was prepared for Illumina and Nanopor
RNA sequencing.
The original statement has been modified as "In view of the following two factors: 1) the sex determination system of T. d
chromosome [30]; 2) there was only one pair of newly emerged beetles of different sexes obtained for genome sequencin
was prepared for Illumina and Nanopore sequencing, and then a male thorax was dissected for Hi-C and RNA sequencing

(14) Line 113. 1) What is the methodology for these rearing medias? Is there control to make sure they have similar micr
residue just pure fungus? Or does that include wood in the media that has been decomposed by mycelia? 3) More descript
for these samples.
4) Additionally, would these beetles be found feeding solely in fungi in the wild? Or would they always be consuming a mix
description of the biology of this species' feeding would help for readers who are not familiar with this beetle in the wild (s
Authors response:
1) Yes, all of the rearing medias were sterilized by high temperature steam.
2) No, it is not just fungus. The mushroom residue is composed of wood fiber and fungal mycelia.
3) What the reviewer suggested is meaningful. According to the above mentioned comments, the original description has
reared with high-temperature sterilized sawdust and mushroom-residue (composed of wood fiber and fungal mycelia) at 2
4) Yes, the detailed description of the biology of this species' feeding is necessary for this manuscript. The feeding biology
"Introduction" and third paragraph of "3.6 Differentially expressed digestion-related genes", i.e., "In the larval stage, it ca

[19-21], ……" (lines 70-71), and "In the forest, the larvae of T. dichotomus usually inhabit soil organic matter and feed on
of the white-spotted flower chafer, Protaetia brevitarsis (Scarabaeidae), which also efficiently digests high lignocellulosic r
coexisting in the outdoor mushroom-residue, ……" (lines 563-568).

(15) Line 115/Figure 1. 1) While it's nice to see that the wood fiber was digested, what's the rationale for providing these
don't seem terribly important to me). 2) Also, what was the methodology of taking these photos? Is this wood fiber from t
these (I assume scanning electron microscopy)? 3) Were there strategies employed to make sure there was no bias in sel
of the entire amount of diet tissue, and not just exemplars representing that some of the wood fiber was digested but not
Authors response:
1) Figure 1 shows the 3rd instar larva of Trypoxylus dichotomus (a), the wood fiber structures of sawdust before (b) and a
lignocellulose by T. dichotomus visually. Thus, the preliminary research result of figure 1 is important and meaningful for t
figure.
2) Figure 1a refers to the 3rd instar larva of T. dichotomus, which was taken by a digital single lens reflex camera. Figures
(ESEM). The wood fiber before degradation (Figure 1b) was taken from sawdust, while the wood fiber after degradation w
sawdust.
3) Yes, figure 1c can represent the wood fiber structure of sawdust after digestion by the 3rd instar larvae of T. dichotomu
photographed by ESEM, the wood fiber structures of sawdust were degraded into the similar fragments after digestion by
4) The original statement has been be supplemented with the above-mentioned information. The updated statement is "A
single lens reflex camera), six larval excrement samples were randomly selected and photographed by an environment sc
sawdust before digestion (Fig. 1b, taken by ESEM), the wood fiber structures of sawdust were degraded into similar fragm
124).

(16) Line 127. What does "gene library construction" refer to?
Authors response: It refers to the short-insert (350 bp) and large-insert (>20 kb) libraries for genome survey and sequen
original statement, i.e., "Genomic DNA was extracted using a QIAGEN® genomic kit for short-insert (350 bp) and large-in
instructions" (lines 136-138).

(17) Line 135. What was this "certain concentration and volume" used?
Authors response: The concentration and volume were 50 fmol and volume 24 μL, respectively. The original description ha
fmol) and volume (24 μL) of DNA library ……" (lines 147-148).

(18) Line 155. What is meant by "double check"? Do you mean using NCBI's EGAP? Or just their standard quality control?
Authors response: After checking, we find out that the phrase "double check" is an inaccurate statement in the original de
kb were retained and uploaded to NCBI for contamination detection in the final assembly" (lines 173-175).

(19) Line 150-163. I am unclear on what the authors mean by "NGS data" here. The genome assembly of the ONT data se
reference around like 158? I am not following these methods. I assume this is just the TruSeq libraries used for polishing
genome size estimation but not for anything else?).
Authors response:
1) In the line 150 of the first manuscript, the phrase "Next-Generation Sequencing (NGS) data" refers to the Illumina DNA
The original statement is easy to be misunderstood, so the original description has been amended as "The Illumina DNA d
The corrected genomic data were polished with the filtered DNA data of the genome survey using Nextpolish (v1.0.5) ove
been replaced by "Illumina DNA data of the genome survey" in the lines 178-179 or "Illumina DNA data from a genome su
replaced by "Illumina RNA data" in the lines 328-329.
2) The original statement "As a reference genome, ……GC depth was analyzed using Minimap2 and Samtools." is verified t
"Moreover, to verify utilization of raw data and the completeness of genome assembly, the Illumina DNA data of the geno
mapped to the genome assembly using Minimap2. Then, the mapping rates were calculated using SAMtools v1.9 [38]", ha
original corresponding results of this analysis "The mapping rates of NGS (GSS, RNA-seq) and ONT reads onto our draft g
modified as "The mapping rates of Illumina DNA data from the genome survey, Illumina RNA data of the male thorax and
99.60%, respectively" (lines 373-376).

(20) Line 173. How were the libraries quantified?
Authors response: The statement of the libraries quantification, i.e., "Libraries were quantified using Qubit 3.0 fluorometry
extracted using a QIAGEN® genomic kit for short-insert (350 bp) and large-insert (> 20 kb) library construction according

(21) Line 232. "unreliable" is not a term used by BMGE. What exact BMGE commands were used?
Authors response: Yes, the word "unreliable" is not enough to describe the concrete usage of the program BMGE. The deta
so the present description is "The unreliable homologous regions were removed with BMGE v1.12 (-m BLOSUM90 -h 0.4)

(22) Table 1. Should read "NextDenovo".
Authors response: Yes, it was misspelled. The proper name "NextDenovo" has replaced the incorrect one in the Table 1 (li

(23) Line 387. What does "commendably recovered" mean?
Authors response: It means the phylogenetic relationships of these 14 insect species are same as the previous phylogeno
statement has been amended as "The phylogenetic relationships of 14 insect species were well recovered [83, 100], ……"

(24) Line 417. 1) This sentence does not make sense grammatically. 2) Additionally, based on the beetle genomes availab
operate (since you only sample 9 beetle species).
Authors response:
1) The original sentence has been modified as " Most beetles were considered not to capitalize on their significant ability f
dichotomus " (lines 457-458).
2) This statement was cited from the 101th article in the references, i.e., "Calderón-Cortés N, Quesada M, Watanabe H, C
mechanism in insect evolution. Annu Rev Ecol Evol Syst. 2012;43:45-71" (lines 918-920), which indicated that most beet

(25) Figure 6a. Which samples in the PCA are SM2 and SM6? Additionally, I am red-green colorblind and have a somewha
Authors response: The original colors "green" and "red" in "Figure 6a" have been replaced by "purple" and "blue", respect
have been marked in the updated figure "Figure 6a", separately.

(26) Line 480. 1) What is the rationale for this statement? I can see that perhaps there is more separation in ordination sp
along PC2 (8%) compared to separation of arguably similar numbers of SH vs MH along PC1 (86%), so I would almost pu
along PC2. 2) Overall, I am not convinced by statements throughout the study about midgut having stronger digestion ab
Authors response:
1) The original statement is verified to be incorrect. The section 3.5 has been reworded according to the reviewer's comm
PERMANOVA analysis has been added in the revised manuscript. The updated content of "section 3.5" is as follows (lines 5
To further explore the intestinal gene expression patterns associated with different gut tissues and food habits, we carried
on the gene expression (FPKM) of all annotated genes for each sample by PERMANOVA, we found significant differences o
habits (Table 4). PCA and PCC (Tables S13 and S14) were then used to calculate and plot diagrams (Fig. 6), respectively.
were mainly aggregated together, except for four outliers (SM2, SM6, SH2 and SH3) in the midgut and hindgut of sawdus
repeatability within most of the intra-groups, but a relatively low level of repeatability in the midgut of sawdust feeding la
For the groups with the same food habits (SM and SH, MM and MH), more significant differences of gene expressions were
the mushroom-residue groups along PC1 and PC2. Furthermore, there were also significant differences between groups w
gene expressions could be significantly affected by food habits in T. dichotomus. Consistently, it was reported that differe
Trogoderma granarium [28].

2) The original statements about midgut having stronger digestion ability than hindgut and the comparisons of wood vs. f
been modified as "These results indicate that more digestion-related genes are highly expressed in the midgut than the hi
lignocellulose in larvae may require more digestive enzymes in the midgut than in the hindgut" (lines 539-543), and "The
greater digestive ability than that of sawdust for the larvae of T. dichotomus, which is probably due to the complex compo
mycelia" (lines 555-559).

(27) Lines 507-511. I am not following the logic here 100%. Presumably mushroom residue would be already broken dow
beetle, but these results indicate more expression of digestion-related genes in the mushroom treatment. Perhaps the dig
ability, not just the lignocellulose?
Authors response: What the reviewer proposed is reasonable. The original statement has been modified as "Taken togethe
residue group than in the sawdust group regardless of whether the specific location was the midgut or hindgut. These resu
digestive ability than that of sawdust for the larvae of T. dichotomus, which is probably due to the complex components o
(lines 553-559).

(28) Lines 536-538. Not technically true since those are not sister taxa. Their ancestors diverged from each other at that
Authors response: Yes, the reviewer's perspective is rational. The original statement has been modified as "……, showing t
Mya) from that of the closely related species O. taurus" (lines 584-585). In addition, the other similar statements have be
430).

Response to Reviewer 2:
Major Comments
(1) Title. The genome is estimated here as 599 MB, but the assembly is 636 MB. That needs to be resolved before claimin
Authors response: The consensus estimated genome size (599 Mb) was inferred from the genome estimation results of Fi
not represent the actual genome size of this beetle. Furthermore, our final genome assembly size (636 Mb) was close to t
was reliable. Not only that, there were 606.8 Mb scaffolds covering 95.37% of the draft reference genome anchored onto
the chromosome-level genome assembly of high quality. As mentioned above, the tile "A chromosome-level genome asse
Scarabaeidae) to understand its lignocellulose digestion ability" is applicable for this manuscript.

(2) Abstract. How is the timing of the split between Td and O taurus is needed in the abstract.

Authors response: The divergence time between the ancestors of Trypoxylus dichotomus and Onthophagus taurus was inf[illegible]
abstract, i.e., "…… in the early Cretaceous (120 Mya)" (lines 23-24).

(3) L39. Beetles are not mentioned in the first paragraph. I realize that lignocellulose digestion might be an important asp[illegible]
should be the main focus. The start of the first paragraph is much better for a biological, not geological, audience.
Authors response: What the reviewer suggested is reasonable to some extent. However, the first paragraph highlighted th[illegible]
the lignocellulose digestion related genes of this beetle, which is important for this manuscript. Thus, the main content of [illegible]
simplified and improved with the first three sentences removed. Given this, the original description at the beginning of firs[illegible]
the improper handling of plant biomass is one of the most important factors accelerating global warming [6, 7]", has been [illegible]
structural component of plant biomass and an important route of carbon fixation, ……" (line 42).

(4) L222 - I was going to put this in the minor comments, but then I realized that it is actually a serious comment. All of t[illegible]
least nine do. If we as a field do not cite reference for the resources that we use, how can we expect people in other fields [illegible]
using the resource here, so they should do the same for others.
Authors response: Yes, the reviewer's advice is reasonable. The citation of genomic resources of the other insects is neces[illegible]
been appended with the bibliographic citation (lines 247 and 250), and the corresponding references [64-74] have been a[illegible]
(Tri. castaneum, Agrilus planipennis, Lamprigera yunnana, Nicrophorus vespilloides, Onthophagus taurus, Aethina tumida[illegible]
insect species (Drosophila melanogaster (Diptera), Apis mellifera (Hymenoptera), Bombyx mori (Lepidoptera), Coptoterm[illegible]
(lines 245-250).

(5) L127. I assume this was done according to the manufacturer's instructions, but state that so the reader does not have[illegible]
Authors response: Yes, this was done according to the manufacturer's instructions. The original description has been ame[illegible]
short-insert (350 bp) and large-insert (> 20 kb) library construction according to the manufacturer's instructions" (lines 1[illegible]

(6) L163. What any type of haplotype reduction used or tried?
Authors response: Haplotype reduction was not used or tried in this manuscript.

(7) L194. 1) HISAT2 - what paramters were used? 2) What any QC done of the RNA-seq reads or the alignments? Again, [illegible]
Authors response:
1) The parameter "--dta" has been added after the software "HISAT2 v2.2.0" (line 218).
2) Quality control of RNA-seq reads and alignments was performed with the program bbduk.sh (qtrim=rl trimq=20 minlen[illegible]
BBTools v38.82. The details of quality control have been added in the following sentence, i.e., "To improve the prediction [illegible]
bbduk.sh (qtrim=rl trimq=20 minlen=20 ecco=t maxns=5 trimpolya=10 trimpolyg=10 trimpolyc=10) in BBTools v38.82 [[illegible]

(8) L200. What QC of the gene models from MAKER was done?
Authors response: Using MAKER, gene prediction was performed with three strategies, Ab initio, transcriptome and protei[illegible]
from 0 and 1, with 0 denoting perfect agreement of the annotation) can be referenced to the quality of gene prediction. F[illegible]
quality control (line 210).

(9) L209. Is eggNOG not a third method to assign gene functional information. How was the information QC and integrate[illegible]
Authors response: Gene function was annotated with two strategies: 1) Gene function was predicted by searching the spe[illegible]
domains, Gene Ontology (GO), and pathways [Kyoto Encyclopedia of Genes and Genomes (KEGG), Reactome] were predi[illegible]
Although InterProScan and eggNOG are both integrated databases, they are usually different in KEGG pathways and GO t[illegible]
Applying these two strategies, we could get as much information of gene function annotation as possible. Furthermore, qu[illegible]
results of above two strategies were finally presented in the 3rd paragraph of section 3.3 (lines 394-400).

(10) L262. That is definitely not the proper citation for the Tc genome. That paper does not report a single nucleotide sequ[illegible]
Authors response: Yes, the original citation for the genome of Tribolium castaneum is improper. Thus, it has been replace[illegible]
Gibbs RA, Weinstock GM, Brown SJ, Denell R, Beeman RW, et al. The genome of the model beetle and pest Tribolium cast[illegible]
817-819).

(11) L298. How does this suggest good or bad sample quality? Heterozygosity is a biological property, as is genome size.
Authors response: Yes, heterozygosity is a biological property for the genome of Trypoxylus dichotomus. The incorrect sta[illegible]
quality of the test sample" removed (line 326).

(12) L298. Sequencing results need to be separated from genome size estimate results.
Authors response: The sequencing results have been separated from genome size estimate results, and placed in the next[illegible]

(13) L321. But 599 Mb was the consensus estimate you gave.
Authors response: Despite using the same Illumina DNA data of the genome survey, the results of genome estimation we[illegible]
GenomeScope (567.4 Mb). For that reason, we provided the consensus estimated genome size (599 Mb) before Oxford Na[illegible]
assembly size was 636 Mb after analyzing ONT sequencing data, which was apparently close to the genome estimation by[illegible]

word “pretty” in the revised manuscript, and the revised sentence is “……, which was close to the earlier genome estimatio

(14) L356. 4% of the BUSCO genes are not represented in the gene set? What number is duplicated or fragmented? All of
much in what seems such a complete assembly.
Authors response: The fragmented genes account for 1% among all the genes in the BUSCO assessment, thus there were
The software MAKER predicted genes based on the pathway of “Ab initio”, with another two pathways “transcriptome” and
information might lead to the weak support for predicted genes. Furthermore, the larger size the genome, the worse the g
gene prediction (95.8%) is relatively high among the other published insect genomes.

(15) L359. How many of the hits were to other scarabs? To other beetles? To other insects? The reader needs more inform
Authors response: The table 2 “Gene hits between Trypoxylus dichotomus and another six insects” has been added in the
insects (line 410). Furthermore, the description of gene hits have also been added in the end of third paragraph of “Genom
other high-quality genome annotations from six insects and revealed more than 10,000 hits (Table 2)” (lines 398-400).

(16) L377. How many beetle only orthogroups were there?
Authors response: There are 12,658 orthogroups only to beetle. The original description has been amended as “Using hom
into 14,467 orthogroups (gene families), in which 12,658 orthogroups were unique to beetles. Moreover, there were 1,26
dichotomus” (lines 414-417).

(17) L413. Why is immunity being seen with the GO analysis for the gene families important? What were the contracted fa
Authors response: The reviewer’s comment reminds us that the immunity is not important to the manuscript. Although th
protease Hayan) ranked in the first 20 rapidly expanded gene families, this manuscript mainly focuses on the digestion of
removed in the revised manuscript. The original statement “……; meanwhile gene family concerning immunity was also af
456).
In addition, GO and KEGG enrichments has been appended as supplementary files (Tables S9 and S10) in the revised man
gene families. The updated statement is “The rapidly expanded gene families were further confirmed in the GO and KEGG
and immunity mainly in the GO enrichment (Fig. 5b), and metabolic detoxification, digestion, juvenile hormone and secon
450).

(18) L466. I fundamentally disagree with that statement. There a large scatter in hind and mid-gut samples along the two
that.
Authors response: Yes, the original statement failed to clarify the result of PCA analysis. The original sentence has been m
same group were mainly aggregated together, except for four outliers (SM2, SM6, SH2 and SH3) in the midgut and hindg
permutational multivariate analysis of variance (PERMANOVA) is newly added in the revised manuscript. The result of PER
comparisons of groups separated by gut tissues and food habits (SM vs SH, MM vs MH, SM vs MM and SH vs MH) (Table 4
Intestinal transcriptome analysis” and the first paragraph of section “3.5 Gene expression and sample correlation”, i.e., “E
(PERMANOVA) was performed with 999 permutations using the R package ‘vegan’ [93]” (line 301-302) and “Based on the
PERMANOVA, we found significant differences of gene expressions between the groups separated by gut tissues or food ha

(19) Section 3.6. I have great difficulty understanding this section. 1) What was the comparison treatment? 2) So there a
shift of diet, esp from native to a manipulated diet. That from two separate tissues too. 3) These sections are very, very d
Authors response:
1) The comparison treatment refers to midgut and hindgut treatments from the same food habit (SM vs SH and MM vs MH
(SM vs MM and SH vs MH) which are listed in the “Table S16”. To clarify the comparison treatment, the detailed informatio
understand the digestive ability of T. dichotomus larvae on different gut tissues and food habits, digestion-related genes w
compared within four different treatment groups, i.e., SM vs SH, MM vs MH, SM vs MM and SH vs MH (Table S16, Fig. 7)”
earlier in the first paragraph of “2.1 Sampling and sequencing”, i.e., “All of the samples were divided into four groups: 1)
from sawdust (SH, hindgut of larva feeding sawdust), 3) midgut from mushroom-residue (MM, midgut of larva feeding mu
feeding mushroom-residue). Each group consisted of six replicates” (lines 130-135).
2) The differentially expressed genes (DEGs) are not 423 in total. As mentioned in this manuscript, there are 222, 231, 92
“SH vs MH”, respectively. Mushroom-residue usually contains fungal mycelia, but it is mainly composed of partially decaye
sawdust, which makes the differences between these two rearing foods are not as big as they look. For this reason, there
the same gut tissue.
3) The original statement did have some inaccuracies, which makes certain content in the above-mentioned section confu
mistakes have been corrected, with the statements being improved in the revised manuscript (see the detailed responses

(20) L500. 1) Much of this is discussion, not results. 2) However, DEGs being different between sawdust and mushrooms
sawdust.
Authors response: This comment is considered to be part of the former one.
1) This paragraph is in the third part “Results and Discussion”, the contents of results are closely followed by the contents
2) Yes, the differences of DEGs between sawdust and mushrooms is not a direct evidence that Trypoxylus dichotomus is b
comment, we have modified the original statement as “These results suggest that digestion of mushroom-residue might r

dichotomus, which is probably due to the complex components of mushroom-residue, including not only wood fiber but als

(21) L512-522. This is not a result.
Authors response: Yes, this is a discussion. The results are closely followed by the content of discussion in the "Results an

Minor Comments
(22) L25. There is no evidence provided that supports statement directly. I think it is likely, but the language needs to be
Authors response: Yes, the original statement is not proper. Thus, it has been modified as "Through gene family evolution
were two digestive gene families (encoding Trypsin and Enoyl-(Acyl carrier protein) reductase) that have experienced sign
degradation efficiency of lignocellulose in T. dichotomus" (lines 24-28).

(23) L29. Need a comma before respectively.
Authors response: The comma has been added before "respectively", i.e., "…… being intersected with chromosomes 2 and

(24) Keywords. Put in alphabetical order.
Authors response: These keywords have been rearranged in alphabetical order, i.e., "Chromosome rearrangement, gene f
(lines 38-39).

(25) L63. "receiving preeminent research achievements" is very odd phrasing.
Authors response: Yes, the original statement is improper. The original sentence has been amended as "Among them, xyl
digesters, having been studied in detail including in terms of functional genomics and symbiotic intestinal microorganisms

(26) L73. Can, not could. It still has this ability as a species.
Authors response: Yes, it refers to the larval ability of Trypoxylus dichotomus. The word "could" has been replaced by "ca

(27) L113. Where did the sawdust and mushrooms come from? What was the temperature? Far more detail is needed her
Authors response: Yes, the rearing details of T. dichotomus larvae are needed for repeatability. The original description ha
"The similar-sized larvae were divided into two groups and reared with high-temperature sterilized sawdust and mushroor
60% humidity for 2 months, separately" (lines 115-118).

(28) L114. Is this needed? Was this in doubt?
Authors response: Yes, the figure 1 reflects the wood fiber structure after digestion by this beetle, which provides the visu
statement more accurate and detailed, the original statement has been amended as "After intake and digestion by the 3rd
larval excrement samples were randomly selected and photographed by an environment scanning electron microscope (ES
1b, taken by ESEM), the wood fiber structures of sawdust were degraded into similar fragments after digestion by the larv

(29) L130. What quality filer was used? What program? What parameters? Or were these just the QC of the Illumina mach
Authors response: Quality control of raw reads was performed using fastp (v.0.20.0) preprocessor. The detailed descriptio
reads were filtered using the fastp (v.0.20.0) preprocessor [31] (set to default parameters) to remove low quality reads, a

(30) L136. What chemistry and chip?
Authors response: The chemistry and chip of PromethION is "FLO-PRO002", which has been added in the sentence "After
DNA library was transferred to a flow cell of PromethION (ONT, Oxford Nanopore Technologies, FLO-PRO002 chip) for who

(31) L137-142. 1) How were the sample homogenized? 2) What version of the kits for TruSeq? 3) What version of chemis
you QC the RNA and the gDNA? Many needed details are missing to make the work reasonably reproducible.
Nothing is mentioned out the Hi-C sequencing mentioned in the previous paragraph.
Authors response:
1) Total RNA was extracted from the male thoracic muscle, "……, and then a male thorax was dissected for Hi-C and RNA s
NanoDrop 2000 and Agilent 2100 system. The sentence "It was qualified and quantified as follows: 1) RNA purity and con
quantity were measured using the Agilent 2100 system" has been added in the revised manuscript (lines 152-154).
2) The version of the kits for TruSeq is TruSeq RNA Library Preparation Kit v2 (line 155).
3) The library preparations were sequenced on an Illumina Novaseq platform (Illumina) according to the manufacturer's in
4) The target insert size was 350 bp in RNA sequencing library (line 156).
According to the above two questions, the original statement has been modified as "The library preparations (350 bp targ
San Diego, CA, USA) to generate 150 bp paired-end reads, according to the manufacturer's instructions" (lines 156-158).
5) The quality of raw reads of RNA and gDNA were controlled by fastp to remove reads containing adapters and low qualit
containing adapters and low quality bases ("N">10%, Q-value≤20) were removed using fastp" has been added in the revi

(32) L145. I assume that all other parameters were default, but do state that.
Authors response: Yes, the other parameters were default for ONT Guppy. The updated statement is "The quality of reads
mean_qscore_template ≥ 7, with the other parameters left at the defaults" (lines 163-164).

(33) L165. 1) How was cross-linking started? 2) Using what basic protocol? Provide the citation. 3) What library construct
Authors response:
1) The cross-linking was started by the process, "quick-freezing tissues of T. dichotomus were vacuum infiltrated in nuclei
2) The protocol was cited from the reference [39] "Belton J-M, McCord RP, Gibcus JH, Naumova N, Zhan Y and Dekker J. H
Methods. 2012;58 3:268-76". The sentence "The Hi-C library was prepared followed by a procedure [39] with improveme
3) The Hi-C library construction kit wasn't used in this study, but the detailed process of library preparation has been prov
by a procedure [39] with improvement modifications. ……. Finally, the Hi-C libraries were quantified and sequenced using
(lines 183-195).
4) The Hi-C libraries were quantified and sequenced on an Illumina Novaseq platform according to the manufacturer's inst

(34) L230. Where all these program used with default settings where no mentioned otherwise? This paragraph has a good
methods.
Authors response: Thanks for the reviewer's recognition for this paragraph (lines 253-268). The reviewer's recommendati
the revised manuscript.

(35) L250. I do not understand what "Natural selection" means here.
Authors response: This is an improper statement. The original sentence has been modified as "Forty-five rapidly expanded
evolution of expanded gene families" (lines 273-275).

(36) L259. Collinearity seems an odd word to me. I have never seen it used in this context. I am more familiar with this c
Authors response: The reviewer's view is showed to be reasonable. Compared to the word "Collinearity", "Synteny" is mor
been replaced by the word "synteny" in the revised manuscript (lines 281, 287, 289, 467-468, 580 and 977).

(37) L270. What quality threshold were used?
Authors response: The detailed parameter "N">10%, Q-value≤20" is added at the end of this sentence. The updated sent
low quality bases ("N">10%, Q-value≤20)" (lines 292-293).

(38) L271. What rRNA database? Please provide the citation.
Authors response: The rRNA database refers to the rRNAs annotated in the genome of this beetle, thus the original staten
mapping short reads to the rRNA database of T. dichotomus with Bowtie2 (version 2.2.8) [91]" (lines 293-294).

(39) L275. How is a transcription region defined? Are those supposed to be genes? What gene set was used. The one from
Authors response: The transcription region refers to the gene assembled by rRNA mapped reads. For ease of understandir
StringTie in a reference-based approach" has been added at the beginning of this paragraph (lines 296-297).

(40) L279. 1) What was the model for differential expression? Tissue by diet, I assume? Please tell the reader. 2) Why is e
for DESeq2? 4) Why were DEGs filtered for KEGG and GO terms? What does that actually mean? 5) Was GO or KEGG term
Authors response:
1) The original statement has been amended as "Differential gene expression analysis was performed with DESeq2 [95] se
tissues from the same diet, or the same gut tissues from different diets" (lines 304-307).
2) There was an error in the original statement. The software "edgeR" was not used in this study, thus it has been deleted
3) The shrinkage estimator for dispersion was used for DESeq2, which has been added in the updated sentence "…… DESe
4) As the mentioned in the sentence "All differentially expressed digestion-related genes were further annotated with KEG
filter out the digestion-related genes based on KEGG pathways and GO terms.
4) The intestinal transcriptome analysis aimed to understand the influences of gut segments and diets on the digestion-re
analysis was considered to be nonessential for this study.

(41) L295. Ratio? Ratio to what?
Authors response: The ratio refers to the repeat ratio in the genome of Trypoxylus dichotomus, which has been mentioned
dichotomus were estimated to be ……" (lines 322-323).

(42) Table 1. Is that actually NextDeNovo in the first line?
Authors response: Yes, it is actually NextDeNovo, the misspelling has been corrected in the table 1 (line 332).

(43) L323. What is sex determination system of this beele? XO, XY?
Authors response: According to the previous chromosomal studies on this species and its closely related beetles, the sex d
This information has been added in the revised manuscript, i.e., "……, 1) the sex determination system of T. dichotomus is
[30]; ……" (lines 108-110). Furthermore, it has the same number of chromosomes with another related species Tribolium
10 (TdChr10) in the sequenced female individual is considered to be X chromosome in the later section of "Synteny".

(44) L332. Clearest example of what?

Authors response: The original statement has been amended as "The clearest example of genomic difference was found in
with a much smaller genome size of 267.08 Mb (Bioproject: PRJNA419349)" (lines 368-370).

(45) L351. MAKER produced a consensus set. Not really predicted.
Authors response: Yes, it was an incorrect statement. The original statement has been modified as "To predict the genes i
among which the average length of genes, CDS and transcripts was 15,150, 1,743, and 2,355 bp, respectively" (lines 387

(46) L352. What is a peptide chain?
Authors response: It is not proper here, which has been deleted in the updated sentence "……, among which the average l
respectively" (lines 388-389).

(47) L393. I assume the families that the GO analyses are to are the ones specific to Td?
Authors response: Yes, GO analyses were targeted at these rapidly expanded gene families. The original statement has be
evolving gene families in T. dichotomus, we used gene family evolution analysis and revealed that 610 and 1,405 gene fa
433-435).

(48) L417. This paragraph is discussion, not results.
Authors response: Yes, the results and discussion are put together in this manuscript. Thus, it was not a problem.
Yes, this is a discussion. In this manuscript, the contents of results and discussion are together in the "Results and Discus

(49) L449. Discussion, not results. Same for L473, 480.
Authors response: This is the similar issue as the former comment. These statements are put in the section of "Results an

(50) L486. Methods, not results.
Authors response: Yes, the original sentence did describe the methods here. Thus, this sentence has been moved into the
modification, the updated statement is "Digestion-related genes were then filtered to exclude those with mean gene count

Response 2 to the manuscript GIGA-D-21-00415

Editor: For the response letter, I hope you can response them point by point and put all detailed changes in the answer (r
were made in the manuscript (highlight them), and I also require you to re-write the first round of response letter in detai
unhappy with your response, then we'll consider rejecting it.
Authors response: Yes, all the comments have been responded with the detailed changes point by point in the first and se
and highlighted with blue green (except for the changes of language editing in English grammar and sentence structure) i
re-written in detail, which has been uploaded to the submission system together with this response.

Reviewer #1: The authors have addressed many of the bigger picture concerns brought up by myself and reviewer 2, and
responded to all of the reviewer comments positively, in many cases they simply replied to our comments in their respons
comments below, line2 104-105 and 110-112, reviewer 2 comments for original line 377). The point of making these com
either reproducible or accurate or would benefit from having additional information; they weren't simply because the revie
authors revisit the original reviewer comments and critically evaluate whether or not they have been substantively addres
Authors response: Yes, there were some important and necessary details without modification in the first revised manuscr
changes answered in the first response. All the changes have been made and highlighted with blue green (except for the c
the second revised manuscript. For the examples the reviewer mentioned in this comment, "line 104-105 and 110-112" in
reviewer 2 comments have been responded with detailed changes under the corresponding comments. The corresponding
108-113, 119-124 and 414-417). However, the example "line 2" is not mentioned in the original and present reviewer's co

While the English has definitely been improved from the original version, the manuscript still needs more editing for gram
abstract only, but throughout the manuscript there are many sentences which are not clear due to ambiguous wording or
company to bring the writing up to publication quality for this journal.
Authors response: The second revised manuscript has been edited by another paid-for-service editing company (Charlesw
structure have been corrected in the revised manuscript.

SPECIFIC COMMENTS:
Line 15. "Improper handling" is ambiguous here since the last sentence is talking about biological mechanisms. i.e., Is it i
to human issues more explicit.

Authors response: Yes, the statement of “Improper handling” is ambiguous. The reviewer’s suggestion that “make that lin [illegible]
been amended as “The traditional management of plant waste including landfill and incineration usually causes serious en [illegible]
sentence “The traditional management of plant waste is usually done via landfill or incineration, which causes serious envi [illegible]
first paragraph of “Introduction” (lines 46-48). Moreover, the corresponding reference [4] “Shah SN, Mo KH, Yap SP, Yang [illegible]
incorporating waste materials: A review. Resources, Conservation and Recycling. 2021;164:105103” has also been added [illegible]

Line 19. How else would a genome be assembled if not by bioinformatic analyses? I'd simplify this sentence by removing t [illegible]
Authors response: Yes, the statement of “by bioinformatic analysis” is unnecessary. Thus, it has been removed in the upd [illegible]
that the draft genome size of T. dichotomus is 636.27 Mb, with 95.37% scaffolds anchored onto 10 chromosomes” (lines [illegible]

Line 23. "was occurred" is grammatically incorrect.
Authors response: Yes, the phrase of “was occurred” is grammatically incorrect. It has been modified as “occurred” in the [illegible]
phylogeny” (line 431). Moreover, the phrase “were evolutionarily occurred” has been modified as “evolutionarily occurred” [illegible]

Line 31-32. Reword to "comparative transcriptome analyses of larval gut" or something similar.
Authors response: Yes, the original statement “larval intestinal transcriptome comparative analyses” has been reworded t [illegible]

Line 34. The subject of "susceptible to different food habits" is unclear in this sentence, grammatically.
Authors response: Yes, the original statement is hard to understand. Thus, the original description has been modified as “ [illegible]
that the digestion-related genes were more commonly expressed in the midgut or mushroom-residue group than the hind [illegible]

Line 64. Move authority to previous line, where species is first mentioned.
Authors response: The authority of Trypoxylus dichotomus has been moved to the previous line where this species is first [illegible]
(Linnaeus, 1771), which has a similar diet to xylophagous termites” (lines 66-67) and “The rhinoceros beetle, T. dichotom [illegible]

Line 104-105. The authors responded to my question about why male vs. female tissue was used different library prep str [illegible]
this biologically relevant information that needs to be included in the manuscript.
Authors response: Yes, the biologically relevant information has been added in the revised manuscript. The updated state [illegible]
system of T. dichotomus is XY, in which the Y chromosome is much smaller than the X chromosome [30]; 2) there was or [illegible]
sequencing; thus, to meet the sequencing requirements, muscle of a female thorax was prepared for Illumina and Nanopo [illegible]
sequencing (Table S1)” (lines 108-113).

Lines 110-112. Again, while the authors responded to my original comment about the rationale for including these data in [illegible]
bringing up the question of "what's the importance of including this in the current study?" my original comment was mean [illegible]
manuscript. i.e., explicitly state that this is the first demonstration that this beetle can digest lignocellulose. Additionally, i [illegible]
to my original comment.
Authors response: Yes, the detailed information of lignocellulose digestion by the larvae of Trypoxylus dichotomus is a use [illegible]
digestion by the T. dichotomus larvae has been added to the second revised manuscript. Thus, the original statement has [illegible]
1a, taken by a digital single lens reflex camera), six larval excrement samples were randomly selected and photographed [illegible]
intact wood fiber of sawdust before digestion (Fig. 1b, taken by ESEM), the wood fiber structures of sawdust were degrade [illegible]
1c)” (lines 119-124).

Line 165. The authors state they clarified this sentence according to my original comment (although I'm still unclear on wh [illegible]
include the addition they reference in their response to my comment.
Authors response: The first response to the reviewer’s original comment is indeed problematic. So, we have rewrote to the [illegible]
1) In the line 150 of the first manuscript, the phrase “Next-Generation Sequencing (NGS) data” refers to the Illumina DNA [illegible]
assembly. The original statement is easy to be misunderstood, so the original description has been amended as “The Illum [illegible]
parameters. The corrected genomic data were polished with the filtered DNA data of the genome survey using Nextpolish [illegible]
DNA data” has been replaced by “Illumina DNA data of the genome survey” in the lines 178-179 or “Illumina DNA data fro [illegible]
data” has been replaced by “Illumina RNA data” in the lines 328-329.
2) The original statement “As a reference genome, ……GC depth was analyzed using Minimap2 and Samtools.” is verified t [illegible]
“Moreover, to verify utilization of raw data and the completeness of genome assembly, the Illumina DNA data of the geno [illegible]
mapped to the genome assembly using Minimap2. Then, the mapping rates were calculated using SAMtools v1.9 [38]”, ha [illegible]
original corresponding results of this analysis “The mapping rates of NGS (GSS, RNA-seq) and ONT reads onto our draft g [illegible]
modified as “The mapping rates of Illumina DNA data from the genome survey, Illumina RNA data of the male thorax and [illegible]
99.60%, respectively” (lines 373-376).

Lines 574-577. Although the authors toned down the conclusions about the relative strength of digestive ability in midgut [illegible]
have been edited to match.
Authors response: Yes, the original statement in the conclusion doesn’t match the relative strength of digestive ability in m [illegible]
amended as “Through a comparative analysis of intestinal transcriptome of larvae feeding on sawdust and mushroom-resi [illegible]
affected by food habits in T. dichotomus. Digestion-related genes were more commonly expressed in the midgut or mushr [illegible]

treatments or gut tissue treatments" (lines 590-595).

Reviewer #2: I think the authors did an acceptable job revising the manuscript and appreciate the effort they put into the

I have only a few further comments about the responses that I will leave to the editor to handle at his/her discretion.

8) So, then, no QC of the MAKER predictions was done? I have used and understand how MAKER works, which is why I as
Authors response: Yes, QC of the MAKER predictions was not done. There might be some false positives in the predicted g
values, the real genes of Trypoxylus dichotomus might also be removed under the condition of insufficient transcriptome d
predicted genes might be real genes even though having AED values greater than 0.5 or 0.75.
https://weatherby.genetics.utah.edu/MAKER/wiki/index.php/MAKER_Tutorial_for_WGS_Assembly_and_Annotation_Winte
Furthermore, the gene prediction of MAKER shows that there are only 94 genes (0.77%) with AED values ≥ 0.75 and 571
Thus, we recommend that all the predicted genes by MAKER should be remained with AED values indicating the quality of

14) 96% is not relatively high. Compare that to Tribolium, Dendroctonus, Nicrhophorus, or Onthophagus. I am not saying
genome. One was to demonstrate its completeness is to have a full or near full (98% or better)gene compliment.
Authors response: Yes, it is not relatively high compared to Tribolium, Dendroctonus, Nicrhophorus, or Onthophagus. The
quality of our genome assembly. During the process of gene prediction, ab initio gene prediction was made by the BRAKEI
GeneMark, which predicted 96.2% (S: 85.7%, D: 10.5%) complete genes. On the basis of ab initio gene prediction, the M
1% fragmented genes. Although the MAKER prediction can generate much more genes by setting the parameters "est2ge
large numbers of false positive genes. The quality control of gene prediction can be performed with AED values, but lots o
transcriptome data and the equal-weights of transcriptome and protein in MAKER.

17) This does not address the question at all. Why is immunity important within this context? I do believe that you found
discussion?
Authors response: The reviewer's comment reminds us that the immunity is not important to the manuscript. This manusc
immunity is not our concern even though the involved gene families are enriched in GO and KEGG analyses. Thus, the dis
original statement "……; meanwhile gene family concerning immunity was also affected by positive selection during evolut

19.1) What was the comparison group, i.e., the group that DE was measured from? That still has not been stated.
Authors response: The differential gene expression of different comparison groups is mentioned in the second paragraph o
analysis was performed with DESeq2 [95] software, with a shrinkage estimator for dispersion between different gut tissue
genes associated with a false discovery rate (FDR) below 0.05 and absolute fold change ≥ 2 (|FC|≥2) were considered as
refers to "different gut tissues from the same diet, or the same gut tissues from different diets", i.e. midgut and hindgut t
sawdust and mushroom-residue treatments from the same gut tissue (SM vs MM and SH vs MH) which are listed in the "T
paragraph of "2.1 Sampling and sequencing", i.e. "All of the samples were divided into four groups: 1) midgut from sawdu
hindgut of larva feeding sawdust), 3) midgut from mushroom-residue (MM, midgut of larva feeding mushroom-residue), 4
residue). Each group consisted of six replicates" (lines 130-135). In order to clarify the comparison groups, the detailed in
statement is "To understand the digestive ability of T. dichotomus larvae on different gut tissues and food habits, digestio
were further compared within four different treatment groups, i.e., SM vs SH, MM vs MH, SM vs MM and SH vs MH (Table
groups (SM vs SH, MM vs MH, SM vs MM and SH vs MH) has also been added in the results of corresponding comparison g

Best wishes,
Junhao

Close
