# Supplementary material for: A chromosome-level genome assembly and intestinal transcriptome of Trypoxylus dichotomus (Coleoptera: Scarabaeidae) to understand its lignocellulose digestion ability
Source: Gigascience. 2022 Jun 28;11:giac059. doi: 10.1093/gigascience/giac059 (PMC9239855; doi:10.1093/gigascience/giac059)
Supplement: giac059_Reviewer_1_Report_Original_Submission — Julian Dupuis -- 1/21/2022 Reviewed [file giac059_reviewer_1_report_original_submission.pdf]

Reviewer Report

Title: A chromosome-level genome assembly and intestinal transcriptome of Trypoxylus dichotomus (Coleoptera: Scarabaeidae) to understand its lignocellulose digestion ability

Version: Original Submission **Date:** 1/21/2022

Reviewer name: Julian Dupuis

Reviewer Comments to Author:

Here, Wang et al. present a chromosome-scale assembly and associated transcriptome experiments for a charismatic species of rhinoceros beetle. Using differential expression analysis, they explore differences between beetle larvae feeding on wood vs. mushrooms and evaluated mid vs. hindgut digestive ability. Overall, most of the analyses are conducted with proper rigor, and this will be a valuable resource for future researchers. However, I have many specific comments on areas where more clarity is needed or additional details and a few larger concerns/suggestions for the study, which I feel need to be addressed before it is ready for publication.

Given the recent release of another assembly of this species (mentioned lines 328-329) which was generated using PacBio reads, the authors are missing out on an interesting comparison between data types (and as-is, I can't evaluate the truth of lines 526-527 that this is the first chromosome-scale assembly for the species). I think some comparative analyses would be quite interesting of these 2 raw assemblies, or at the very least more than a one-line mention that another assembly exists.

Overall, I am not convinced by the mid vs. hindgut and mushroom vs. wood fiber differential expression analysis. This is both for lack of some methodological detail (pertaining the diets that larvae were reared on) and interpretation and resulting conclusion statements about particular parts of the gut having more digestive ability for wood vs. fungi. The latter could probably be addressed by toning down some of the statements of those results or at least more accurately describing patterns in the data.

Finally, I have made several comments in the first few paragraphs concerning grammatical flow, sentence structure, and English considerations, but not throughout the rest of the study (although there were many English errors in the rest of the manuscript). The entire manuscript requires editing from a native English speaker before being fit for publication, as there are many unclear and incorrect statements stemming from grammatical/English mistakes.

SPECIFIC COMMENTS

Line 45. "consists in almost all kinds" is a bit awkward. Perhaps "lignocellulosic biomass is found in all kinds"?

Line 48. "protecting the" should be "protects".

Lines 49-51. The "Thus" beginning of this sentence isn't really following the point of the preceding sentence. Perhaps reordering the sentence to something like: "Recycling plant waste produced by human production is a noteworthy environmental issue."

Line 56. I suggest rewording this sentence to make the subject of the latter half of the sentence clearer (the subject of the first half is "lignocellulose decomposition", but in the second half it is just "lignocellulose").

Line 59. Should be "highly efficient".
Line 60. Should be present tense. Same for the next sentence as well.
Lines 63-64. The termites did not receive research achievements. They were used as study systems. Additionally, while comparison to termites makes biological sense, there is no mention of microbial-aided digestion in the manuscript. Is anything known about the microbiome of this beetle species as compared to termites? Or is their digestion ability fully the beetle's doing and not their gut microbiome?
Line 67. I don't think "biodegradability" really fits here if the authors are referring to the ability to break down the substances from the point of view of the insects.
Line 73. It "can", not "could".
Lines 109-110. Was there rationale for choosing female tissue for some data and male tissue for others?
Line 113. What is the methodology for these rearing medias? Is there control to make sure they have similar microbial contents despite their different composition? Is the mushroom residue just pure fungus? Or does that include wood in the media that has been decomposed by mycelia? More description of these media would greatly improve what conclusions could be made for these samples. Additionally, would these beetles be found feeding solely in fungi in the wild? Or would they always be consuming a mix of wood and fungi (along with other decomposing matter)? Detailed description of the biology of this species' feeding would help for readers who are not familiar with this beetle in the wild (such as myself, as a North American).
Line 115/Figure 1. While it's nice to see that the wood fiber was digested, what's the rationale for providing these images as in-text figures as opposed to supplementary figures (as they don't seem terribly important to me). Also, what was the methodology of taking these photos? Is this wood fiber from the guts of the larvae, or extra-orally digested fiber? What type of image are these (I assume scanning electron microscopy)? Were there strategies employed to make sure there was no bias in selecting what tissue was imaged? (i.e., are these images fully representative of the entire amount of diet tissue, and not just exemplars representing that some of the wood fiber was digested but not necessarily all of it?) No details are provided.
Line 127. What does "gene library construction" refer to?
Line 135. What was this "certain concentration and volume" used?
Line 155. What is meant by "double check"? Do you mean using NCBI's EGAP? Or just their standard quality control?
Line 150-163. I am unclear on what the authors mean by "NGS data" here. The genome assembly of the ONT data seem appropriate, but then some additional NGS data was aligned to the reference around like 158? I am not following these methods. I assume this is just the TruSeq libraries used for polishing the ONT assembly, but this is never specified (or is it just used for the kmer genome size estimation but not for anything else?).
Line 173. How were the libraries quantified?
Line 232. "unreliable" is not a term used by BMGE. What exact BMGE commands were used?
Table 1. Should read "NextDenovo".
Line 387. What does "commendably recovered" mean?
Line 417. This sentence does not make sense grammatically. Additionally, based on the beetle genomes available, I would argue that this analysis doesn't address how "most beetles" operate (since you only sample 9 beetle species).

Figure 6a. Which samples in the PCA are SM2 and SM6? Additionally, I am red-green colorblind and have a somewhat tough time seeing the different colors in this plot.
Line 480. What is the rationale for this statement? I can see that perhaps there is more separation in ordination space of SM vs MM samples (excluding the outliers), but that separation is along PC2 (8%) compared to separation of arguably similar numbers of SH vs MH along PC1 (86%), so I would almost put more stock into slight differentiation along PC1 vs. broad differentiation along PC2. Overall, I am not convinced by statements throughout the study about midgut having stronger digestion ability than hindgut and the comparisons of wood vs. fungi as food material.
Lines 507-511. I am not following the logic here 100%. Presumably mushroom residue would be already broken down compared to wood fiber itself and require less digestion on the part of the beetle, but these results indicate more expression of digestion-related genes in the mushroom treatment. Perhaps the digestion of mushroom components themselves require the extra digestive ability, not just the lignocellulose?
Lines 536-538. Not technically true since those are not sister taxa. Their ancestors diverged from each other at that time.

Methods

Are the methods appropriate to the aims of the study, are they well described, and are necessary controls included? Choose an item.

Conclusions

Are the conclusions adequately supported by the data shown? Choose an item.

Reporting Standards

Does the manuscript adhere to the journal's guidelines on minimum standards of reporting? Choose an item.

Choose an item.

Statistics

Are you able to assess all statistics in the manuscript, including the appropriateness of statistical tests used? Choose an item.

Quality of Written English

Please indicate the quality of language in the manuscript: Choose an item.

Declaration of Competing Interests

Please complete a declaration of competing interests, considering the following questions:

- Have you in the past five years received reimbursements, fees, funding, or salary from an organisation that may in any way gain or lose financially from the publication of this manuscript, either now or in the future?
- Do you hold any stocks or shares in an organisation that may in any way gain or lose financially from the publication of this manuscript, either now or in the future?
- Do you hold or are you currently applying for any patents relating to the content of the manuscript?
- Have you received reimbursements, fees, funding, or salary from an organization that holds or has applied for patents relating to the content of the manuscript?
- Do you have any other financial competing interests?
- Do you have any non-financial competing interests in relation to this paper?

If you can answer no to all of the above, write 'I declare that I have no competing interests' below. If your reply is yes to any, please give details below.

I declare that I have no competing interests.

I agree to the open peer review policy of the journal. I understand that my name will be included on my report to the authors and, if the manuscript is accepted for publication, my named report including any attachments I upload will be posted on the website along with the authors' responses. I agree for my report to be made available under an Open Access Creative Commons CC-BY license (http://creativecommons.org/licenses/by/4.0/). I understand that any comments which I do not wish to be included in my named report can be included as confidential comments to the editors, which will not be published.

Choose an item.

To further support our reviewers, we have joined with Publons, where you can gain additional credit to further highlight your hard work (see: https://publons.com/journal/530/gigascience). On publication of this paper, your review will be automatically added to Publons, you can then choose whether or not to claim your Publons credit. I understand this statement.

Yes Choose an item.
