# Supplementary material for: A chromosome-level genome assembly and intestinal transcriptome of Trypoxylus dichotomus (Coleoptera: Scarabaeidae) to understand its lignocellulose digestion ability
Source: Gigascience. 2022 Jun 28;11:giac059. doi: 10.1093/gigascience/giac059 (PMC9239855; doi:10.1093/gigascience/giac059)
Supplement: giac059_Reviewer_1_Report_Revision_1 — Julian Dupuis -- 4/12/2022 Reviewed [file giac059_reviewer_1_report_revision_1.pdf]

Reviewer Report

Title: A chromosome-level genome assembly and intestinal transcriptome of Trypoxylus dichotomus (Coleoptera: Scarabaeidae) to understand its lignocellulose digestion ability

Version: Revision 1 **Date:** 4/12/2022

Reviewer name: Julian Dupuis

Reviewer Comments to Author:

The authors have addressed many of the bigger picture concerns brought up by myself and reviewer 2, and the manuscript has definitely been improved. However, while the authors responded to all of the reviewer comments positively, in many cases they simply replied to our comments in their response but did not actually address them in the revised manuscript (e.g. my comments below, line2 104-105 and 110-112, reviewer 2 comments for original line 377). The point of making these comments was to show areas of the manuscript that need more detail to be either reproducible or accurate or would benefit from having additional information; they weren't simply because the reviewers were curious about some aspect of the study. I'd suggest that the authors revisit the original reviewer comments and critically evaluate whether or not they have been substantively addressed in another revision of the manuscript.

While the English has definitely been improved from the original version, the manuscript still needs more editing for grammar, sentence structure, etc. Again, I have made some suggestions for the abstract only, but throughout the manuscript there are many sentences which are not clear due to ambiguous wording or sentence structure. I'd suggest the authors use a paid-for-service editing company to bring the writing up to publication quality for this journal.

SPECIFIC COMMENTS:

Line 15. "Improper handling" is ambiguous here since the last sentence is talking about biological mechanisms. i.e., Is it improper handling from the perspective of cells? I'd suggest making that link to human issues more explicit.

Line 19. How else would a genome be assembled if not by bioinformatic analyses? I'd simplify this sentence by removing that descriptor.

Line 23. "was occurred" is grammatically incorrect.

Line 31-32. Reword to "comparative transcriptome analyses of larval gut" or something similar.

Line 34. The subject of "susceptible to different food habits" is unclear in this sentence, grammatically.

Line 64. Move authority to previous line, where species is first mentioned.

Line 104-105. The authors responded to my question about why male vs. female tissue was used different library prep strategies. However, the point of my asking that question was to point out that this biologically relevant information that needs to be included in the manuscript.

Lines 110-112. Again, while the authors responded to my original comment about the rationale for including these data in the manuscript, they didn't include any of that detail in the revised text. By bringing up the question of "what's the importance of including this in the current study?" my original comment was meant to indicate that that information would be a useful addition to the manuscript. i.e.,

explicitly state that this is the first demonstration that this beetle can digest lignocellulose. Additionally, include all the methodological details here that were included in the response to my original comment.
Line 165. The authors state they clarified this sentence according to my original comment (although I'm still unclear on what is meant here), however the text in the revised manuscript does not include the addition they reference in their response to my comment.
Lines 574-577. Although the authors toned down the conclusions about the relative strength of digestive ability in midgut vs. hindgut in the discussion, the conclusion paragraph does not seem to have been edited to match.

Methods

Are the methods appropriate to the aims of the study, are they well described, and are necessary controls included? Choose an item.

Conclusions

Are the conclusions adequately supported by the data shown? Choose an item.

Reporting Standards

Does the manuscript adhere to the journal's guidelines on minimum standards of reporting? Choose an item.

Choose an item.

Statistics

Are you able to assess all statistics in the manuscript, including the appropriateness of statistical tests used? Choose an item.

Quality of Written English

Please indicate the quality of language in the manuscript: Choose an item.

Declaration of Competing Interests

Please complete a declaration of competing interests, considering the following questions:

- Have you in the past five years received reimbursements, fees, funding, or salary from an organisation that may in any way gain or lose financially from the publication of this manuscript, either now or in the future?
- Do you hold any stocks or shares in an organisation that may in any way gain or lose financially from the publication of this manuscript, either now or in the future?

- Do you hold or are you currently applying for any patents relating to the content of the manuscript?
- Have you received reimbursements, fees, funding, or salary from an organization that holds or has applied for patents relating to the content of the manuscript?
- Do you have any other financial competing interests?
- Do you have any non-financial competing interests in relation to this paper?

If you can answer no to all of the above, write 'I declare that I have no competing interests' below. If your reply is yes to any, please give details below.

I declare that I have no competing interests

I agree to the open peer review policy of the journal. I understand that my name will be included on my report to the authors and, if the manuscript is accepted for publication, my named report including any attachments I upload will be posted on the website along with the authors' responses. I agree for my report to be made available under an Open Access Creative Commons CC-BY license (http://creativecommons.org/licenses/by/4.0/). I understand that any comments which I do not wish to be included in my named report can be included as confidential comments to the editors, which will not be published.

Choose an item.

To further support our reviewers, we have joined with Publons, where you can gain additional credit to further highlight your hard work (see: https://publons.com/journal/530/gigascience). On publication of this paper, your review will be automatically added to Publons, you can then choose whether or not to claim your Publons credit. I understand this statement.

Yes Choose an item.
