# Supplementary material for: A chromosome-level genome assembly and intestinal transcriptome of Trypoxylus dichotomus (Coleoptera: Scarabaeidae) to understand its lignocellulose digestion ability
Source: Gigascience. 2022 Jun 28;11:giac059. doi: 10.1093/gigascience/giac059 (PMC9239855; doi:10.1093/gigascience/giac059)
Supplement: giac059_Reviewer_2_Report_Original_Submission — Christopher Cunningham -- 1/23/2022 Reviewed [file giac059_reviewer_2_report_original_submission.pdf]

Reviewer Report

Title: A chromosome-level genome assembly and intestinal transcriptome of Trypoxylus dichotomus (Coleoptera: Scarabaeidae) to understand its lignocellulose digestion ability

Version: Original Submission **Date:** 1/23/2022

Reviewer name: Christopher Cunningham

Reviewer Comments to Author:

I find the paper has lots of merit. Fig 3 is fantastic.
However, I think many needed details are missing from the methods for reasonable reproducibility. I find some of the conclusions poorly justified.
Major Comments
Title. The genome is estimated here as 599 MB, but the assembly is 636 MB. That needs to be resolved before claiming a chromosome level assembly.
Abstract. How is the timing of the split between Td and O taurus is needed in the abstract.
L39. Beetles are not mentioned in the first paragraph. I realize that lignocellulose digestion might be an important aspect to this work, but the paper reports a beetle genome assembly. That should be the main focus. The start of the first paragraph is much better for a biological, not geological, audience.
L222 - I was going to put this in the minor comments, but then I realized that it is actually a serious comment. All of the used genomic resources need citations. I know off the top of my head at least nine do. If we as a field do not cite reference for the resources that we use, how can we expect people in other fields to cite them. I assume the authors here want people to cite this work when using the resource here, so they should do the same for others.
L127. I assume this was done according to the manufacturer's instructions, but state that so the reader does not have to wonder.
L163. What any type of haplotype reduction used or tried?
L194. HISAT2 - what paramters were used? What any QC done of the RNA-seq reads or the alignments? Again, more details are needed in this section to make it reasonably reproducible.
L200. What QC of the gene models from MAKER was done?
L209. Is eggNOG not a third method to assign gene functional information. How was the information QC and integrated?
L262. That is definitely not the proper citation for the Tc genome. That paper does not report a single nucleotide sequence.
L298. How does this suggest good or bad sample quality? Heterozygosity is a biological property, as is genome size.
L298. Sequencing results need to be separated from genome size estimate results.
L321. But 599 Mb was the consensus estimate you gave.
L356. 4% of the BUSCO genes are not represented in the gene set? What number is duplicated or fragmented? All of those should have been in each proteome used to annotate. Odd to lose so much in what seems such a complete assembly.

L359. How many of the hits were to other scarabs? To other beetles? To other insects? The reader needs more information here better assess the quality of the annotation.
L377. How many beetle only orthogroups were there?
L413. Why is immunity being seen with the GO analysis for the gene families important? What were the contracted families and their enriched GO terms, if any?
L466. I fundamentally disagree with that statement. There a large scatter in hind and mid-gut samples along the two axes. Hind and mid definitely separate, but little consistent pattern after that.
Section 3.6. I have great difficulty understanding this section. What was the comparison treatment? So there are 423 DEGs in total? That seems like very few genes given the complete shift of diet, esp from native to a manipulated diet. That from two separate tissues too. These sections are very, very difficult to get through and interpret.
L500. Much of this is discussion, not results. However, DEGs being different between sawdust and mushrooms is not direct evidence the beetles are better at digesting mushrooms than sawdust.
L512-522. This is not a result.
Minor Comments
L25. There is no evidence provided that supports statement directly. I think it is likely, but the language needs to be moderated.
L29. Need a comma before respectively.
Keywords. Put in alphabetical order.
L63. "receiving preeminent research achievements" is very odd phrasing.
L73. Can, not could. It still has this ability as a species.
L113. Where did the sawdust and mushrooms come from? What was the temperature? Far more detail is needed here for repeatability.
L114. Is this needed? Was this in doubt?
L130. What quality filer was used? What program? What parameters? Or were these just the QC of the Illumina machine. What machine was used?
L136. What chemistry and chip?
L137-142. How were the sample homogenized? What version of the kits for TruSeq? What version of chemisty for the Novaseq? Where? What was the target insert size? How did you QC the RNA and the gDNA? Many needed details are missing to make the work reasonably reproducible.
Nothing is mentioned out the Hi-C sequencing mentioned in the previous paragraph.
L145. I assume that all other parameters were default, but do state that.
L165. How was cross-linking started? Using what basic protocol? Provide the citation. What library construction kit was used? What chemistry for the NovaSeq?
L230. Where all these program used with default settings where no mentioned otherwise? This paragraph has a good level of detail. That level needs to be replicated for each section in the methods.
L250. I do not understand what "Natural selection" means here.
L259. Collinearity seems an odd word to me. I have never seen it used in this context. I am more familiar with this concept as "synteny."
L270. What quality threshold were used?
L271. What rRNA database? Please provide the citation.
L275. How is a transcription region defined? Are those supposed to be genes? What gene set was used.

The one from the genome annotation above?
L279. What was the model for differential expression? Tissue by diet, I assume? Please tell the reader. Why is edgeR used? What what to samples? What dispersion estimator was used for DESeq2? Why were DEGs filtered for KEGG and GO terms? What does that actually mean? Was GO or KEGG term enrichment analysis not done?
L295. Ratio? Ratio to what?
Table 1. Is that actually NextDeNovo in the first line?
L323. What is sex determination system of this beele? XO, XY?
L332. Clearest example of what?
L351. MAKER produced a consensus set. Not really predicted.
L352. What is a peptide chain?
L393. I assume the families that the GO analyses are to are the ones specific to Td?
L417. This paragraph is discussion, not results.
L449. Discussion, not results. Same for L473, 480.
L486. Methods, not results.

Methods

Are the methods appropriate to the aims of the study, are they well described, and are necessary controls included? Choose an item.

Conclusions

Are the conclusions adequately supported by the data shown? Choose an item.

Reporting Standards

Does the manuscript adhere to the journal's guidelines on minimum standards of reporting? Choose an item.

Choose an item.

Statistics

Are you able to assess all statistics in the manuscript, including the appropriateness of statistical tests used? Choose an item.

Quality of Written English

Please indicate the quality of language in the manuscript: Choose an item.

Declaration of Competing Interests

Please complete a declaration of competing interests, considering the following questions:

- Have you in the past five years received reimbursements, fees, funding, or salary from an organisation that may in any way gain or lose financially from the publication of this manuscript, either now or in the future?
- Do you hold any stocks or shares in an organisation that may in any way gain or lose financially from the publication of this manuscript, either now or in the future?
- Do you hold or are you currently applying for any patents relating to the content of the manuscript?
- Have you received reimbursements, fees, funding, or salary from an organization that holds or has applied for patents relating to the content of the manuscript?
- Do you have any other financial competing interests?
- Do you have any non-financial competing interests in relation to this paper?

If you can answer no to all of the above, write 'I declare that I have no competing interests' below. If your reply is yes to any, please give details below.

I declare that I have no competing interests.

I agree to the open peer review policy of the journal. I understand that my name will be included on my report to the authors and, if the manuscript is accepted for publication, my named report including any attachments I upload will be posted on the website along with the authors' responses. I agree for my report to be made available under an Open Access Creative Commons CC-BY license (http://creativecommons.org/licenses/by/4.0/). I understand that any comments which I do not wish to be included in my named report can be included as confidential comments to the editors, which will not be published.

Choose an item.

To further support our reviewers, we have joined with Publons, where you can gain additional credit to further highlight your hard work (see: https://publons.com/journal/530/gigascience). On publication of this paper, your review will be automatically added to Publons, you can then choose whether or not to claim your Publons credit. I understand this statement.

Yes Choose an item.
